# Natural and after colon washing fecal samples: the two sides of the coin for investigating the human gut microbiome

**DOI:** 10.1038/s41598-022-20888-z

**Published:** 2022-10-25

**Authors:** Elisabetta Piancone, Bruno Fosso, Marinella Marzano, Mariangela De Robertis, Elisabetta Notario, Annarita Oranger, Caterina Manzari, Silvia Bruno, Grazia Visci, Giuseppe Defazio, Anna Maria D’Erchia, Ermes Filomena, Dominga Maio, Martina Minelli, Ilaria Vergallo, Mauro Minelli, Graziano Pesole

**Affiliations:** 1 Department of Biosciences, Biotechnology and Biopharmaceutics, University of Bari ‘Aldo Moro’, 70126 Bari, Italy; 2 Institute of Biomembranes, Bioenergetics and Molecular Biotechnologies, Consiglio Nazionale delle Ricerche, 70126 Bari, Italy; 3 Specialistic Allergic Unit & Immunological Pathologies, PoliSmail Network, 73100 Lecce, Italy; 4 Centro Direzionale Isola F2, Pegaso Online University, 80132 Naples, Italy; 5 Consorzio Interuniversitario Biotecnologie, 34100 Trieste, Italy

**Keywords:** Metagenomics, Classification and taxonomy

## Abstract

To date several studies address the important role of gut microbiome and its interplay with the human host in the health and disease status. However, the selection of a universal sampling matrix representative of the microbial biodiversity associated with the gastrointestinal (GI) tract, is still challenging. Here we present a study in which, through a deep metabarcoding analysis of the 16S rRNA gene, we compared two sampling matrices, feces (F) and colon washing feces (CWF), in order to evaluate their relative effectiveness and accuracy in representing the complexity of the human gut microbiome. A cohort of 30 volunteers was recruited and paired F and CWF samples were collected from each subject. Alpha diversity analysis confirmed a slightly higher biodiversity of CWF compared to F matched samples. Likewise, beta diversity analysis proved that paired F and CWF microbiomes were quite similar in the same individual, but remarkable inter-individual variability occurred among the microbiomes of all participants. Taxonomic analysis in matched samples was carried out to investigate the intra and inter individual/s variability. Firmicutes, Bacteroidota, Proteobacteria and Actinobacteriota were the main phyla in both F and CWF samples. At genus level, *Bacteirodetes* was the most abundant in F and CWF samples, followed by *Faecalibacterium*, *Blautia* and *Escherichia-Shigella*. Our study highlights an inter-individual variability greater than intra-individual variability for paired F and CWF samples. Indeed, an overall higher similarity was observed across matched F and CWF samples, suggesting, as expected, a remarkable overlap between the microbiomes inferred using the matched F and CWF samples. Notably, absolute quantification of total 16S rDNA by droplet digital PCR (ddPCR) revealed comparable overall microbial load between paired F and CWF samples. We report here the first comparative study on fecal and colon washing fecal samples for investigating the human gut microbiome and show that both types of samples may be used equally for the study of the gut microbiome. The presented results suggest that the combined use of both types of sampling matrices could represent a suitable choice to obtain a more complete overview of the human gut microbiota for addressing different biological and clinical questions.

## Introduction

The human gastrointestinal (GI) tract harbors an intricate and dynamic population of microorganisms termed gut microbiota. It includes bacteria, archaea, viruses and protists, which co-evolve and live in mutualistic cooperation with the host. About 10^14^ microbial cells colonize the human gut and outnumber by an order of magnitude the total number of human cells also providing a much higher gene complement compared to the number of human genes^1,2^. Recent advances in microbiome research highlight that the human gut microbiome begins to develop in utero^3,4^ and delivery mode (vaginal birth vs. Cesarean section), breastmilk and the cessation of breastfeeding are considered to be essential for shaping adult-like gut microbiota^5^. During the first years of life, the intestinal microbiota gradually changes over time, whereas in adulthood the composition of the gut microbiota remains relatively stable^6^.

Gut microbiota composition is shaped by gender and age, diet, antibiotic usage, lifestyle, ethnicity, living environment and host genetics^7–13^. Recently, various studies demonstrated the importance of the gut microbiota for human health providing many benefits to the host, through a spectrum of physiological functions such as protection against pathogens and immune-system development, formation of intestinal epithelium and maintenance of intestinal integrity^14–16^.

The alteration of the stable composition of the gut microbiota, a condition known as dysbiosis, leads to a disruption of homeostasis with the host. Different types of dysbiosis are associated with various harmful effects on human health and long-term consequences may induce a wide range of diseases including inflammatory bowel disease (IBD), irritable bowel syndrome (IBS), diabetes mellitus, obesity, and colorectal cancer^17–22^. Investigating the composition of the gut microbiota, throughout the gastrointestinal tract and understanding the role that microbial populations play in human intestinal health and diseases can be relevant for achieving a possible early and appropriate diagnosis and, consequently, for the eventual development of appropriate therapeutic approaches based on gut microbiota manipulation (e.g. prebiotics, probiotics, fecal transplants).

Further complication in setting the appropriate therapeutic approach is given by the remarkable inter-individual variation of the gut microbiota^23,24^. In fact, despite the composition of the human intestinal microbiota is preserved at high taxonomic levels, it varies enormously from individual to individual^25–27^.

In the past two decades, a multitude of studies were designed to understand and define the composition of a "normal" and "perturbed" microbiota. To date, this goal has still not been achieved also due to the difficulty of setting a universal sampling method capable of representing the entire and reliable composition of the gut microbiota.

Current sampling methods used to investigate the complexity of the gut microbiota are mainly based on feces and tissue samples, obtained by endoscopy or colonoscopy such as biopsy, microdissection and luminal brush^28^. Fecal samples represent by far the most used specimen to investigate the complexity of the human microbiome, since they represent a convenient, repeatable and non-invasive sampling method for screening a large cohort of subjects. Moreover, fecal samples are able to provide suitable biomass for gut microbiome analysis. Most of the knowledge about bacterial diversity in the human GI tract has been achieved by metagenomics analysis from fecal samples, as also reported in international projects^29,30^. Indeed, it has been proven that a more reliable and comprehensive representation of the human gut microbiota composition, in particular that associated with the small and large intestine, both at mucosal and luminal level can be obtained through the direct analysis of the relevant tissues^31–34^. However, the sampling methods to be adopted, based on endoscopy or colonoscopy, show multiple flaws.

First, these sampling methods are invasive procedures, not patient-friendly (unfeasible for healthy controls) and can induce drastic alterations in the intestinal microbiota due to the preliminary bowel preparation and extensive use of laxatives^28,35,36^. Furthermore, these methods suffer from unavoidable contamination and insufficient biomass yield, are expensive, time-consuming and require expertise and specific equipment^37,38^. Interestingly, recent findings demonstrate the potential of endoscopic colonic lavage to assess gut microbial diversity. It consists in the injection of a large amount of fluid into the colon during colonoscopy. Although endoscopic colonic lavage also requires the use of laxatives prior to sampling collection, some studies suggested that it could be an appropriate sampling method ensuring at the same time a less invasive procedure and significantly higher biomass yield than the corresponding colon biopsies^37^. An additional sampling protocol is colon washing or colonic hydrotherapy, which is similar to the endoscopic colonic lavage but is not performed with a preliminary bowel preparation and laxatives that could induce long-term and substantial change in the intestinal microbiota^13,36,39^. The latter protocol is adopted in some clinical centers and may represent another valid alternative approach for the microbiome analysis without the limitations previously mentioned of colon biopsies and of endoscopic colonic lavage (Patera S., Gennaro A., Minelli M.P.M. 2021. Metodica di prelievo enterico di biomassa microbica dopo Idrocolonterapia (ICT). Patent n. IT102019000017459).

Based on these premises, in this study we aim to evaluate the effectiveness of the colon washing (CWF), compared to feces (F), which actually represent the standard choice for the microbiome analysis for taking into account some fundamental aspects for a clinical routine analysis, such as biodiversity representativeness of the human gut microbiota, reduced costs and invasiveness.

Therefore, we performed a deep metabarcoding analysis of the 16S rRNA gene (V4 region) of feces (F) and colon washing feces (CWF) of the same subjects, in order to: i) taxonomically characterize the microbiome associated with both selected matrices; ii) investigate the potential intra and inter-individual variability related to F and CFW samples; iii) evaluate their accuracy in representing the complexity of human gut microbiome.

## Results

### Assessment of microbial load in feces and colon washing fecal samples

Paired samples (feces, F, and colon washing feces, CWF) were collected from 30 subjects (Supplementary Table S1). All subjects underwent a similar colon washing procedure performed the day after collecting fecal samples.

Total DNA extracted from the paired samples, F and CWF, was qualitatively analyzed, resulting intact. By droplet digital PCR (ddPCR) we assessed the microbial load of all samples, by measuring the number of 16S rRNA gene copies.

**Figure 1:**
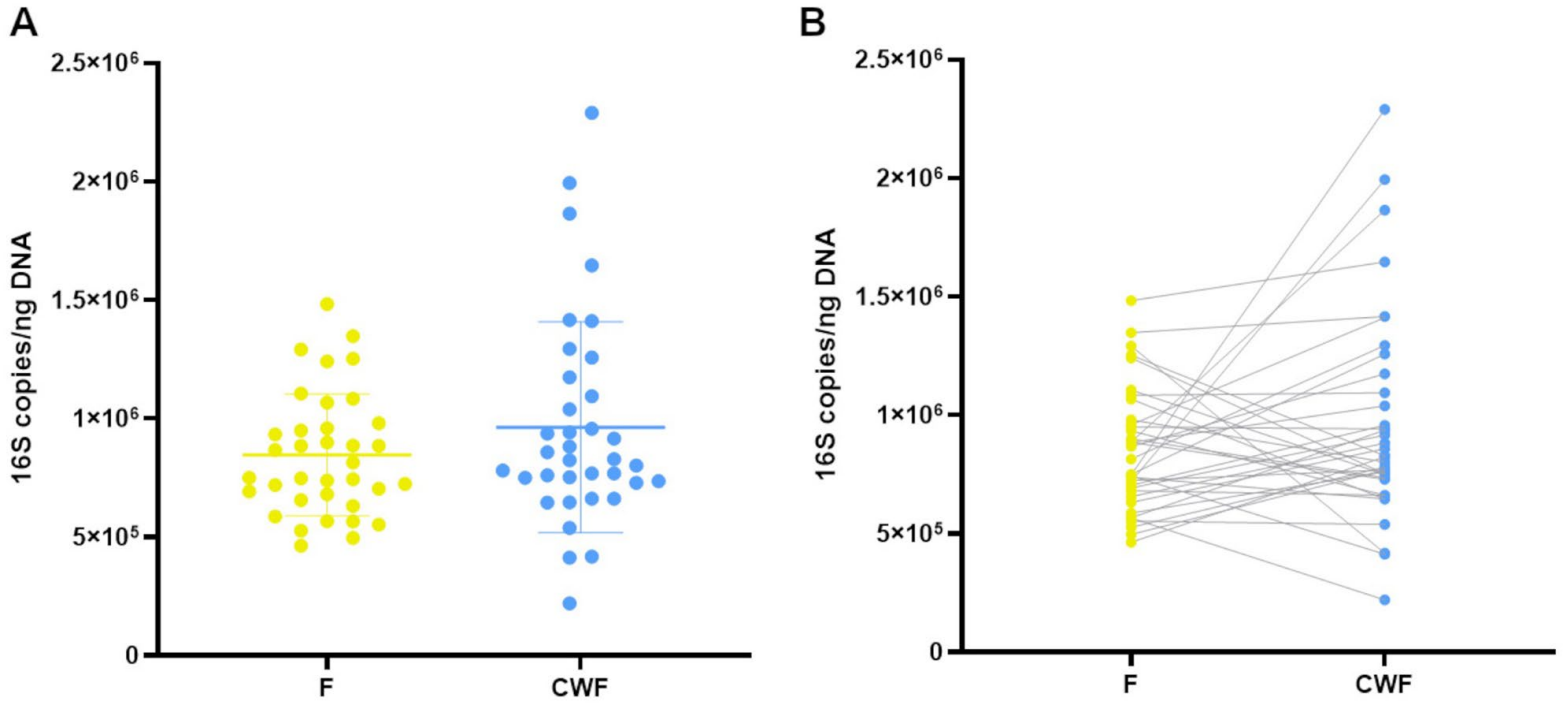
16S absolute quantification of DNA extracted from F and CWF sample matrices. Data are reported as the mean of triplicate ddPCR experiments and expressed as 16S copies/ng of total DNA (**A** dot-plot; **B** dumbbell plot).

We found that the total 16S copies per ng of DNA resulted comparable between F and CWF samples (F median = 782.7; min = 464.0; max = 1482.9; IQR = 280.0; CWF median = 826.6; min = 219.7; max = 2290.4; IQR = 414.7; Fig. 1) and no relevant statistical differences were observed (two-tailed t-test p = 0.15). This result indicates no difference in the total bacterial load between F and CWF samples.

### DNA metabarcoding analysis of F and CWF microbiomes

In order to compare the microbiome composition of F and CWF samples in the same individual we analyzed paired samples by a deep metabarcoding sequencing analysis of a specific hypervariable region (V4 region) within the bacterial 16S rRNA gene. About 9.7 million of paired-end reads (mean/sample = 162,146 ± 59,226) were generated across all samples, and following the trimming, merging and denoising procedures we retained about 97% of initial sequences. Overall, we obtained 16,105 Amplicon Sequencing Variants (ASVs) assessing their taxonomic classification as described in the Methods section. We removed from further analyses 61 chloroplast, mitochondrial and unclassified sequences as well as 131 additional contaminant ASVs.

**Figure 2:**
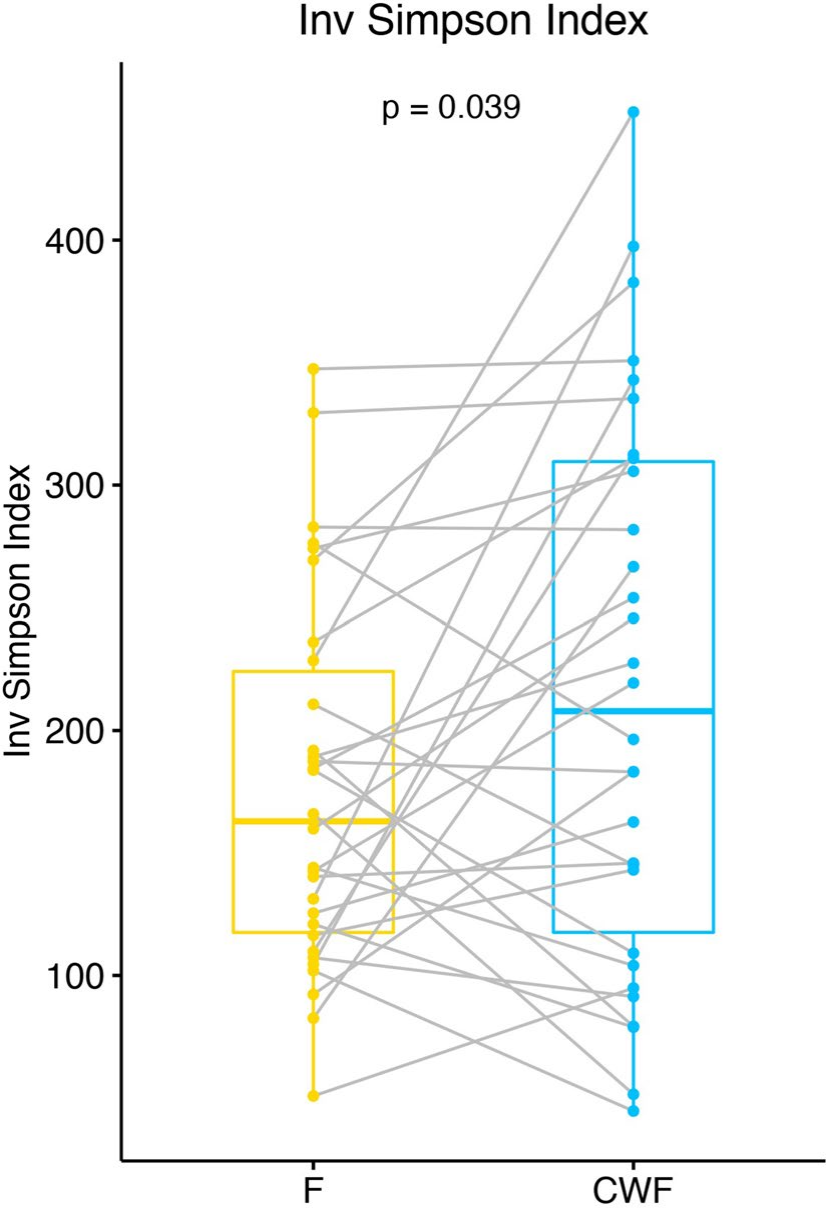
Dumbbell plot of alpha diversity, measured using the Inverse Simpson’s richness index, for matched F and CWF samples. Alpha diversity scores were calculated by using rarefied data at an equal sampling depth of 68,000 sequences. Box-plot and points represent the overall data distribution and samples, respectively. Samples belonging to the same enrolled subject were connected by using a grey line. The group mean comparison was performed by using the Student’s t-test.

The alpha (intra-sample) diversity was investigated by using the Inverse Simpson index and compared in matched F and CWF samples by using the paired two-tailed Student’s t-test, resulting in a marginally significant higher diversity in CWF than in F samples (p-value 0.039, Fig. 2). In order to account for the effect of confounding factors (i.e. age and gender) and the non-independency of paired measures (i.e. F and CWF samples are two measures of the same instance), we built a regression model based on GLM (General Linear Models). In particular, we used the inferred Inverse Simpson scores as the response variable and the age, gender and samples matrix as explanatory variables and subjects as random effect (to address the hierarchical sampling nature). Overall, the model was able to explain about 46% of the conditional variance (R^2^), with a higher contribution of random effects (i.e. subjects) than fixed (gender and matrices), 45% and 0.08%, respectively. No statistically relevant association with gender and age was identified, whereas negative association among fecal sampling and alpha diversity was confirmed (p = 0.032, Supplementary Table S2). A similar trend, even if not significant, in alpha diversity distributions was observed using the number of observed ASVs and the Pielou Evenness indexes (Supplementary Figure S1).

**Figure 3:**
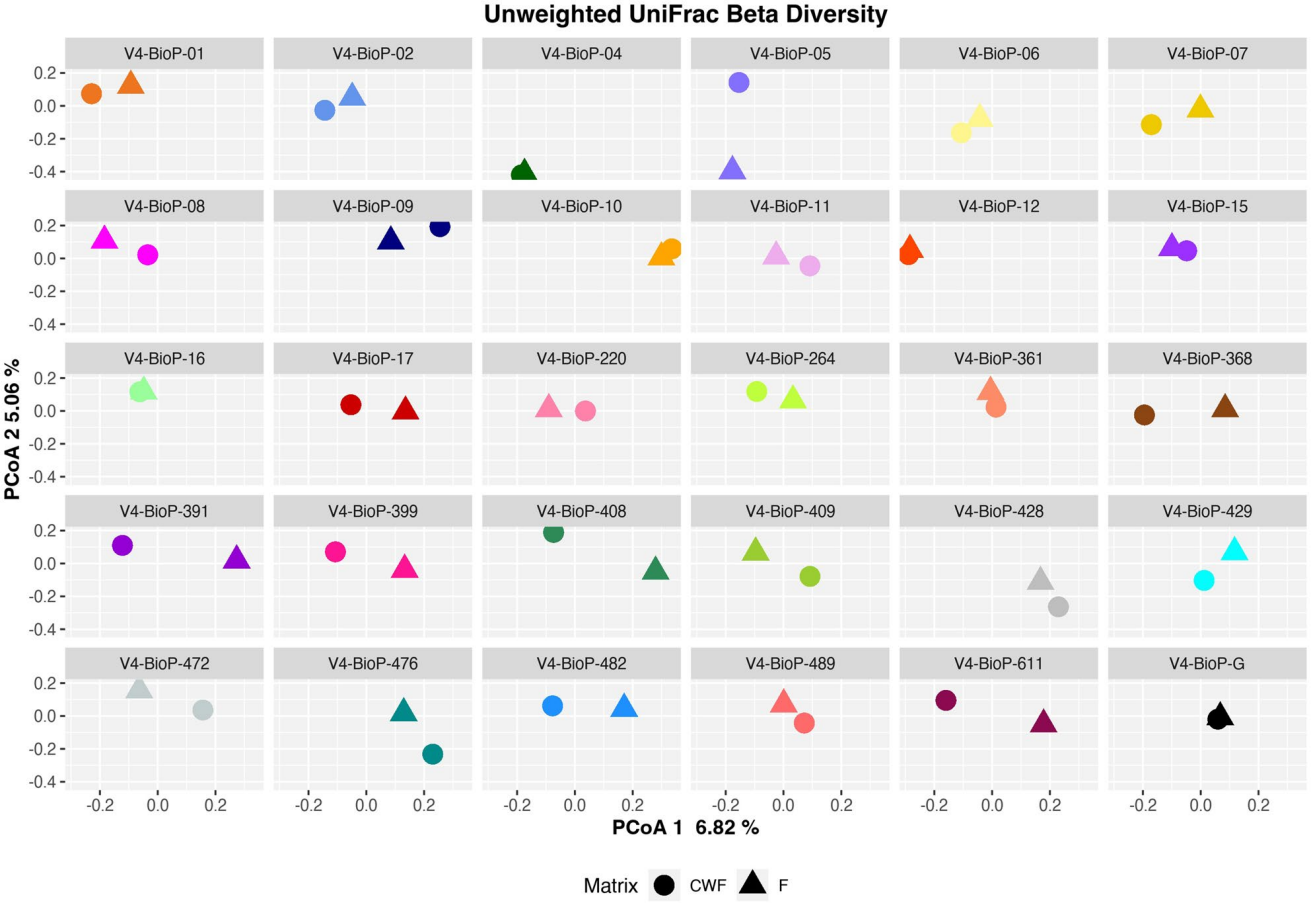
PCoA of unweighted UniFrac distances between paired F and CWF samples for the subjects dataset. Point shape and colors represent the sampling matrix and the enrolled subject, respectively.

Following, we inspected the inter-sample diversity (i.e. Beta diversity) by using the weighted and unweighted UniFrac based PCoA (Principal Coordinates Analysis) plots. Overall, in both weighted and unweighted UniFrac PCoA (Supplementary Figures S2 and S3) we observed that the paired F and CWF microbiomes tend to cluster together. In particular, in Supplementary Figure S2  it is possible to note that the first component (~ 29% of the observed variability) is driven by the most dissimilar paired samples (e.g. V4-BioP-17) while on the left side of the plot it is possible to note the closest paired samples (e.g. V4-BioP-12 and V4-BioP-16). Regarding the PCoA plot based on unweighted UniFrac dissimilarity matrix, the first component (~ 7% of the observed variability) is driven by subjects variability, as most of the paired samples are closed one to each other (e.g. V4-BioP-12, V4-BioP-16, V4-BioP-04 and V4-BioP-G). The second component (~ 5%) discriminates most divergent pairs (e.g. V4-BioP-05, V4-BioP-06 and V4-BioP-07). To better evaluate the similarity between microbial communities of paired F and CWF samples, we fractioned the Unweighted UniFrac based PCoA according to the enrolled subjects (Fig. 3). We observed that F microbiomes clustered with the matched CWFs for most of the enrolled subjects, suggesting that the microbial composition of F and CWF samples from the same individual was similar.

Consistent with these findings, the PERMANOVA analysis on weighted and unweighted UniFrac dissimilarity matrices confirmed that paired F and CWF microbiome did not significantly differ from each other and about 49% of the observed variability was related to inter-individual variability (Supplementary Table S3).

**Figure 4:**
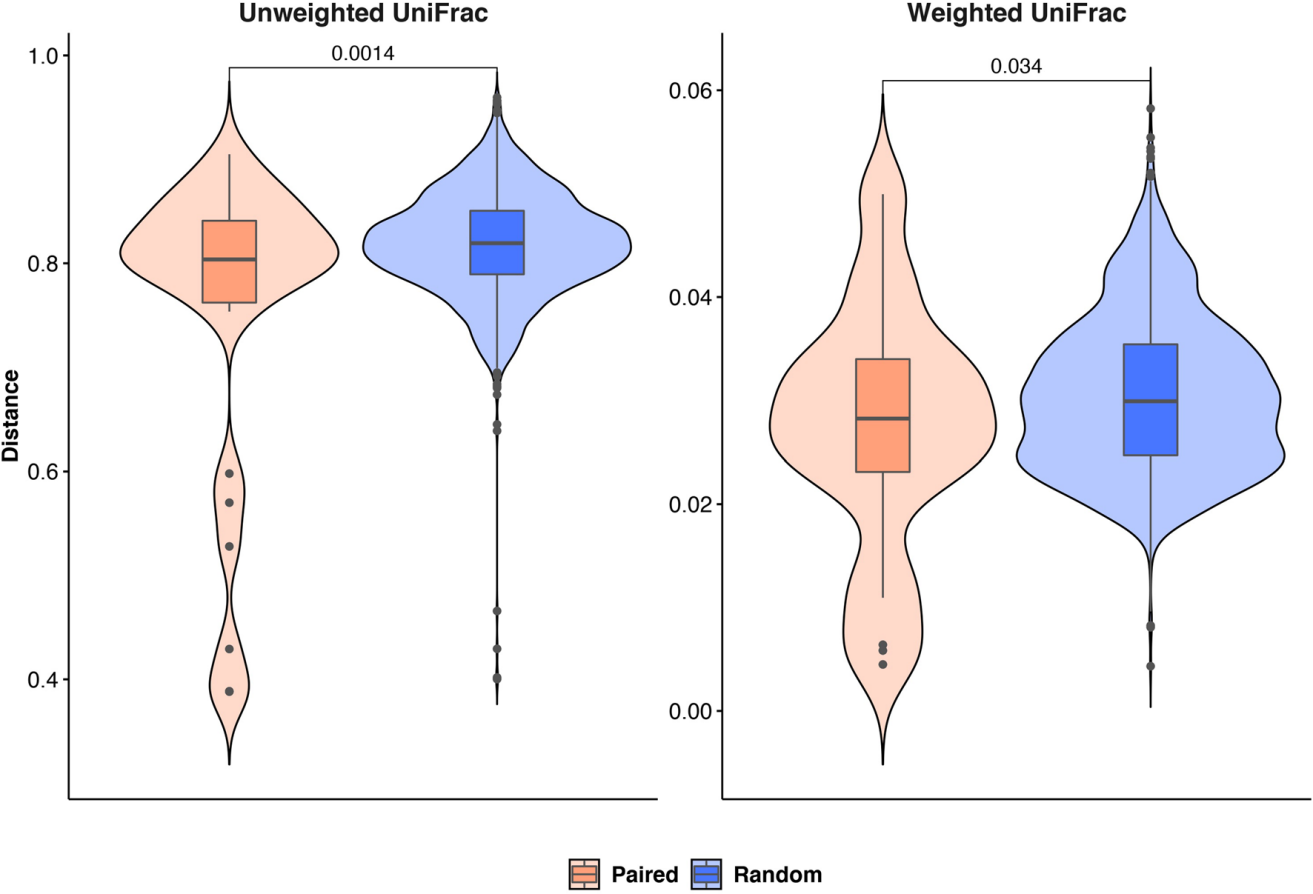
Violin plot showing the observed weighted and unweighted UniFrac distances among paired (i.e. samples belonging to the same individual but from different matrices) and random pairs. The resulting p-value is shown for both comparisons.

Moreover, we compared the observed unweighted and weighted UniFrac distances among matched samples and random pairs (i.e. samples from two randomly extracted subjects) by using the two-tailed Student’s t-test and showing the values distribution as a violin plot (Fig. 4). A statistically significant difference among the distribution of paired and random distances was observed for both the applied metrics.

Taking into account all these results, three major findings emerged: (1) CWF samples were characterized by a higher alpha-diversity compared to F samples; (2) matched samples (i.e. samples from the same subject) were generally more similar (i.e. less distant) compared to random pairs; (3) data variability was mostly driven by individual specificities.

The observed ASVs were taxonomically annotated by using BioMaS and the release 138 of the SILVA database as reference collection and taxonomy (Supplementary Table S4).

**Figure 5:**
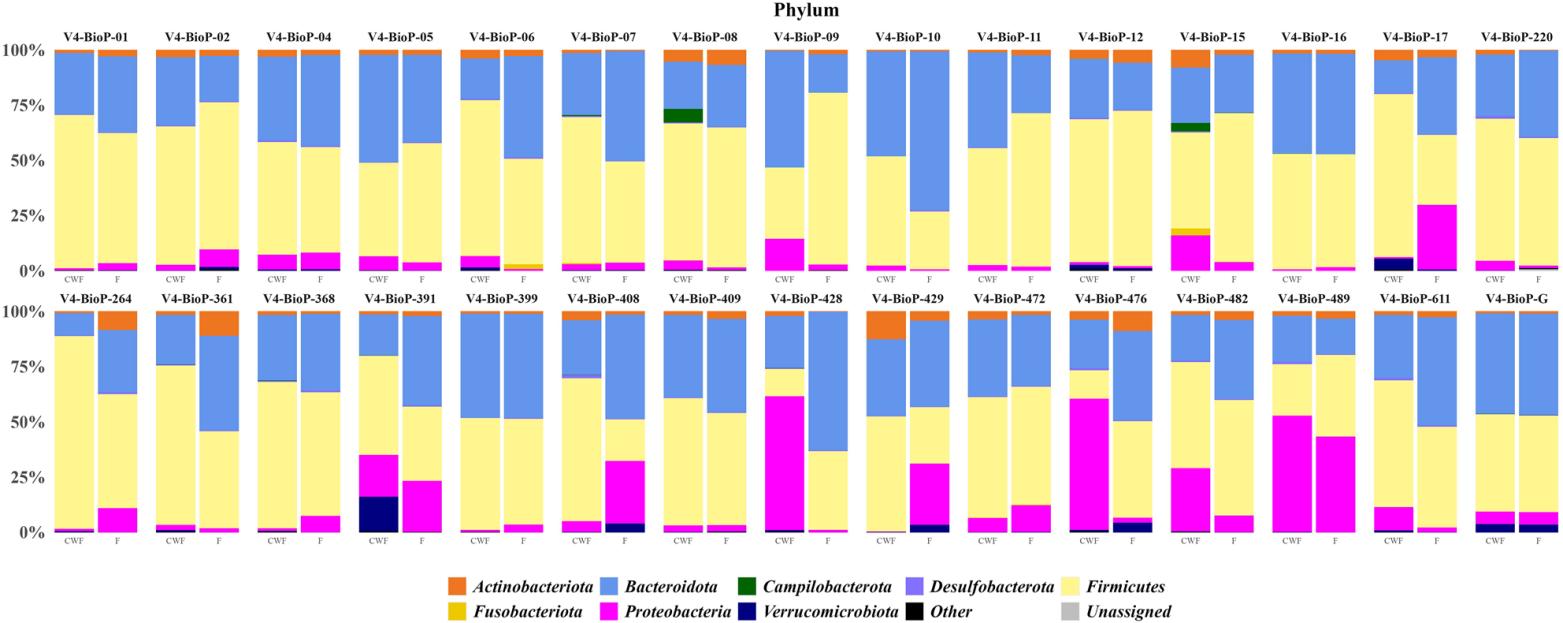
Bacteria relative abundances at the phylum level. Only taxa with a relative abundance equal or higher to 1% were shown. Rare taxa were collapsed as “Other”.

The observed main phyla were Firmicutes (F = 49.2% ± 14.1% vs CWF = 53.5% ± 17.1%), Bacteroidota (F = 38.3% ± 12.4% vs CWF = 30.7% ± 11.2%), Proteobacteria (F = 8.4% ± 10.8% vs CWF = 11.1% ± 17.1%) and Actinobacteriota (F = 3.0% ± 2.4% vs CWF = 2.7% ± 2.5%; Fig. 5).

The phylum Firmicutes was the most abundant in both F and CWF samples (predominantly represented by the Clostridia class) and the main families observed in both F and CWF samples were represented by *Lachnospiraceae* (F = 21.5% ± 8.5% vs CWF = 21.29% ± 9.3%) and *Ruminococcaceae* (F = 16.5% ± 7.1% vs CWF = 17.4% ± 9.3%), in which *Blautia* (F = 3.9% ± 3.2% vs CWF = 4.4% ± 4.0%) and *Faecalibacterium* (F = 8.3% ± 5.1% vs CWF = 9.5% ± 7.2%) were the most represented genera, respectively. Regardless of the sampling method, Bacteroidota was the second most abundant phylum (Bacteroidia was the only observed class) mainly composed of *Bacteroidaceae* (F = 30.7% ± 12.6% vs CWF = 21.0% ± 12.0%), *Prevotellaceae* (F = 1.8% ± 5.0% vs CWF = 4.1% ± 6.6%) and *Rikenellaceae* (F = 3.1% ± 2.7% vs CWF = 2.1% ± 1.7%) families. *Bacteroides* (*Bacteroidaceae*) was the most abundant genus in F and CWF samples (F = 30.7% ± 12.6% vs CWF = 21.0% ± 12.0%). Proteobacteria, mainly constituted by the class Gammaproteobacteria, was the third most abundant phylum in both sample groups and *Escherichia-Shigella,* belonging to the family *Enterobacteriaceae,* was the most observed genus (F = 4.7% ± 8.4% vs CWF = 9.5% ± 7.2%). Lastly, Actinobacteriota phylum was predominantly represented by the family *Bifidobacteriaceae* (F = 2.1% ± 2.0% vs CWF = 1.1% ± 1.1%).

**Figure 6:**
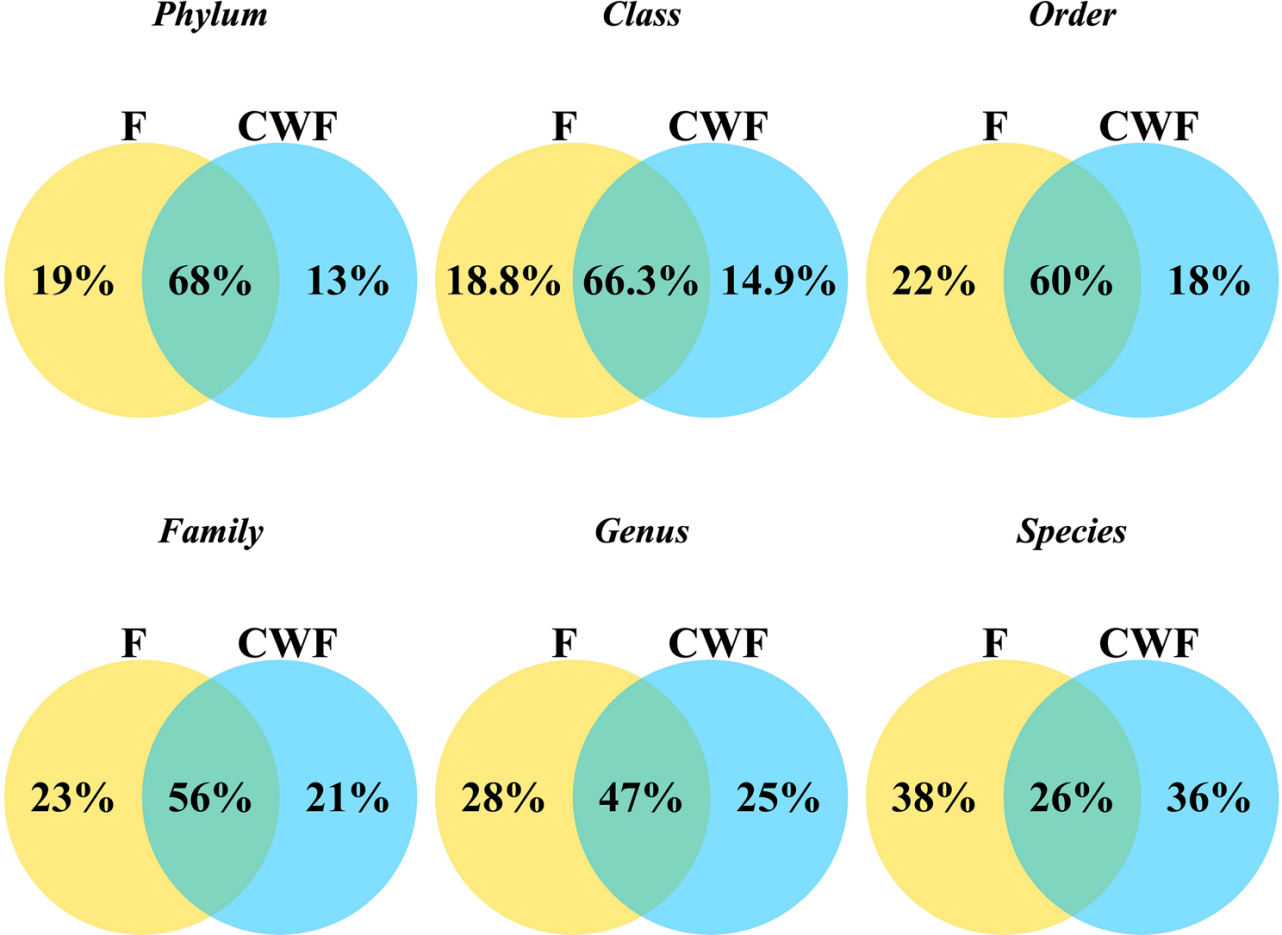
Venn diagrams depicting relative amounts of unique and shared taxa among F and CWF paired samples at phylum, class, order, family, genus and species levels.

An overall view of the core microbiome shared between F and CWF paired samples is shown in Venn diagrams on taxonomic rank from Phylum to Species (Fig. 6).

We identified a considerable common core between paired F and CWF samples at high taxonomic levels, especially at phylum, class, order, family and genus levels (68%, 67%, 60%, 56% and 47%, respectively). Only at the species level we observed a relevant decrease of shared taxa between F and CWF paired samples (26%). Nonetheless, a specific F and CWF fingerprint microbiome appeared at all the investigated levels. Moreover, according to per individual Venn diagrams (Supplementary Figures S4, S5 and S6), the number of taxa shared or exclusively observed in one of the tested matrices was peculiar to each individual, confirming a wide inter-individual variability.

In order to evaluate if there was a specific microbial signature associated with the F or CWF sampling matrix, we focused on the taxa observed in F but not in CWF samples and vice versa in at least one subject. Then we computed a Prevalence Score (Score = F prevalence – CWF prevalence, ideally ranging from −30 to + 30, Supplementary Table S5) summarizing the taxa exclusivity (i.e. taxon significantly prevalent into a specific matrix). In particular, the closer to zero the score was, the less exclusive the taxon was (i.e. its prevalence is similar among the investigated matrices), on the other hand, the higher the absolute score value was, the more exclusive the taxon was (Supplementary Figures S7 and S8). We noticed that regardless the observed taxonomic rank most of the taxa score close to zero, indicating a lack of a clear mark associated with the sampling procedure. Nonetheless, 15 taxa showed a marked prevalence imbalance among the sampling matrices (10 in F and 5 in CWF samples, Supplementary Figure S9).

**Figure 7:**
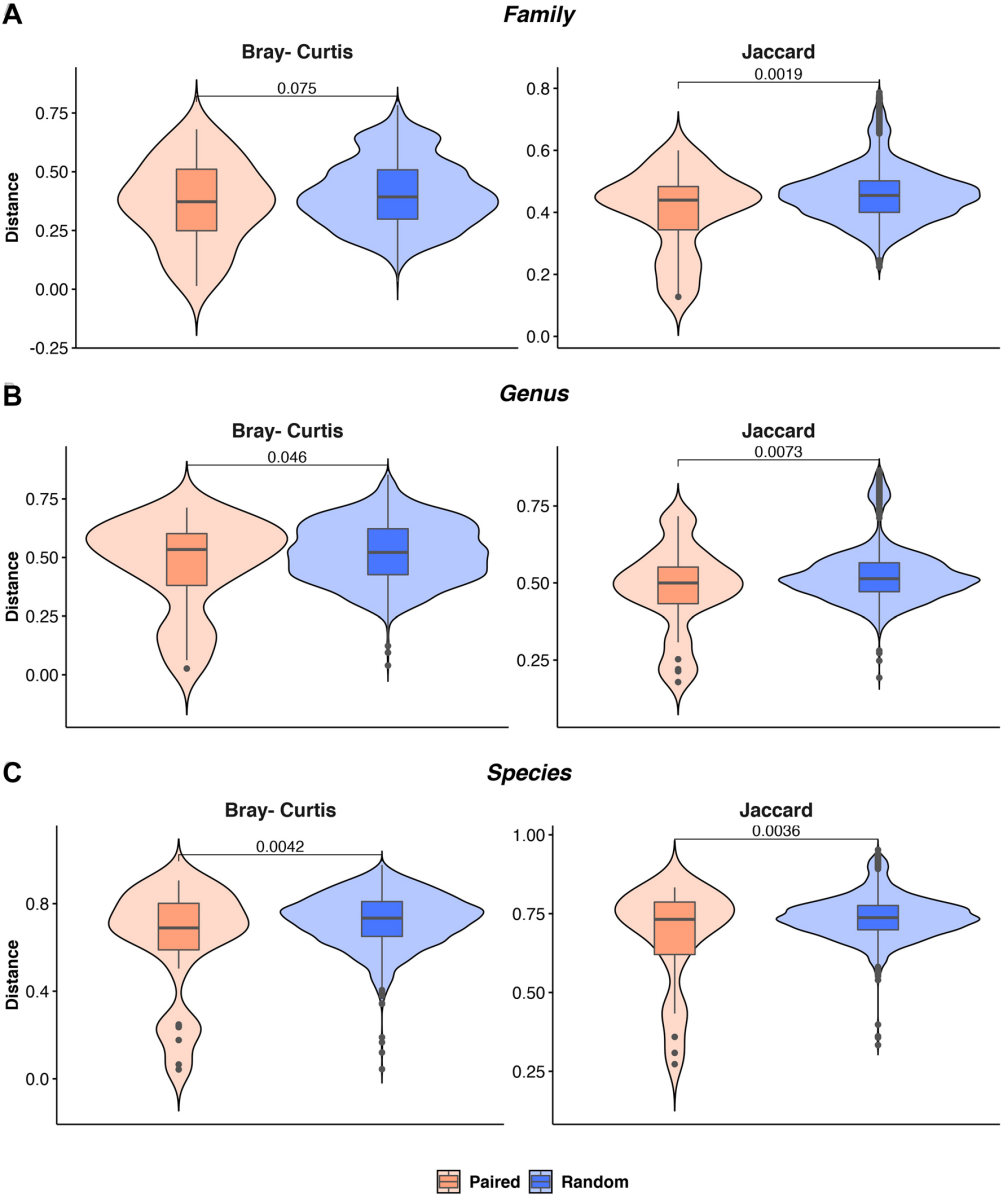
Violin plot showing the observed distances, measured by using both the Bray–Curtis and Jaccard metrics, among paired (i.e. samples belonging to the same individual but from different matrices) and random pairs at family, genus and species ranks. The resulting p-value is shown for each comparison.

Following, by using the Bray–Curtis (quantitative) and the Jaccard (qualitative) metrics, we measured the inter-sample diversity (CWF vs F) at family, genus and species rank and then compared the distance distributions in paired and random samples (Fig. 7) by using the two tailed Student’s t-test. Statistically significant differences between paired and random samples were observed at genus (p = 0.046) and species level (p = 0.0042) by using the Bray–Curtis metrics, confirming results obtained by using ASVs (Fig. 4). Regardless the analyzed taxonomic rank, distance distributions resulted statistically different by using the Jaccard distance, supporting a substantial overlap of the taxa observed in the same individual by using the two alternative sampling approaches.

### Microbial network

**Figure 8:**
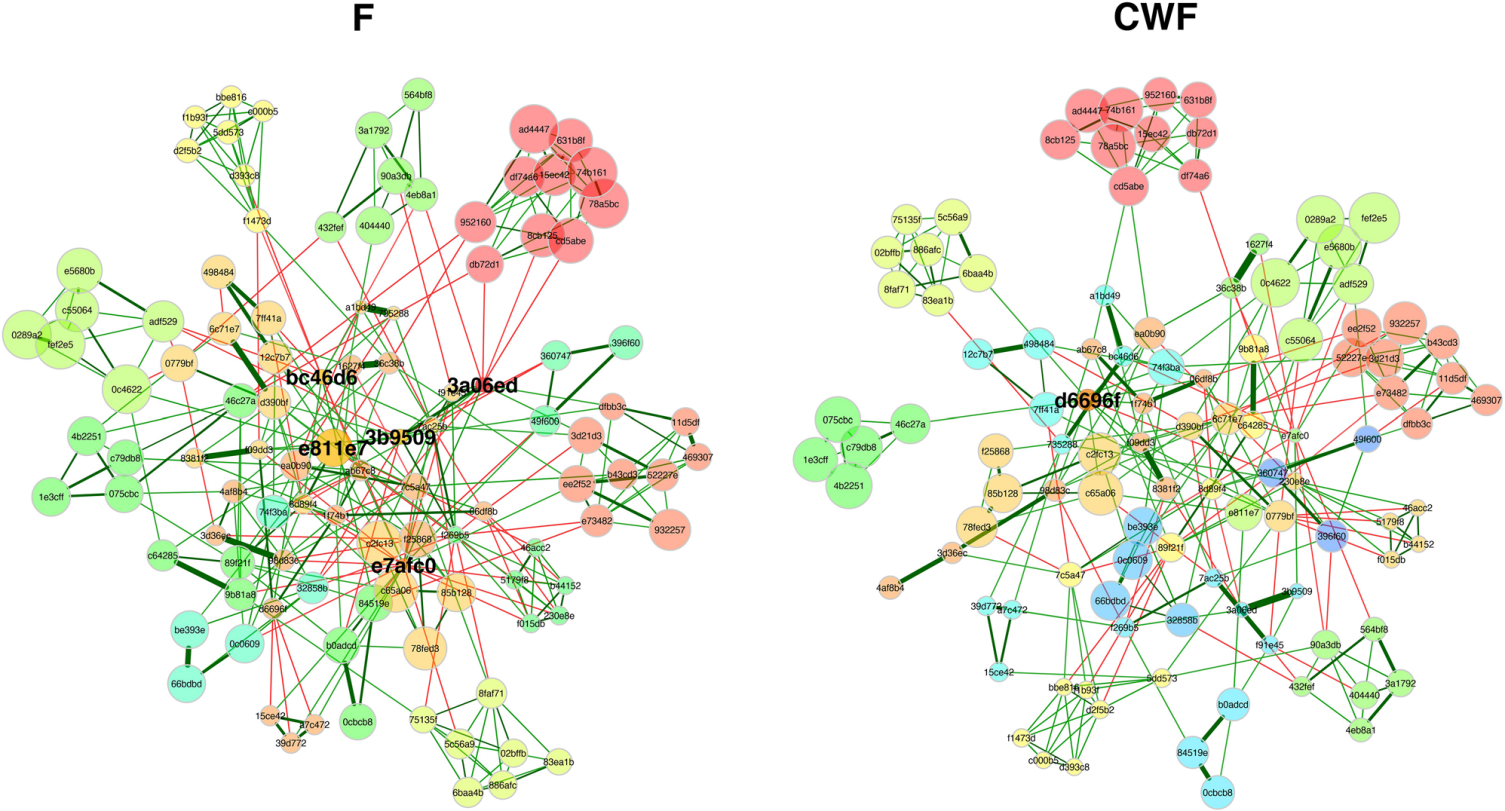
F and CWF microbial networks obtained by using the NetCoMi framework. Nodes are sized and coloured according to normalized counts and cluster membership. Edges color reflects the correlation among nodes (green and red for positive and negative correlations, respectively). In particular, cluster sharing at least 5 nodes among the two networks were plotted using the same colour. Details about node name and taxonomic classification are available in Supplementary Table S6.

By using the NetCoMi^40^ framework, two microbial networks, one per sampling matrix, were inferred and then compared (Fig. 8). In the two networks a similar structure and nodes clustering was observed. This evidence was also supported by measuring the Adjusted Rand Index (ARI), a measure of clustering agreement among networks^41^. ARI was equal to 0.481 (p = 0), supporting a compliance among the two networks clustering. Indeed, clusters were superimposable among the two networks, although node sizes were different, supporting the previous finding that most of the taxa were commonly recalled in F and CWF samples but in different relative abundances. In the F network five hub nodes were identified, whereas just one in CWF. In particular, three out of 5 hub nodes in the F networks were classified as *Citrobacter*, the remaining two as *Prevotella* and *Subdoligranulum*. The unique CWF hub was classified as *Christensenellaceae R-7 group* (Supplementary Table S6). Hub nodes were grouped in different clusters among networks, supporting the evidence that the sampling procedure influences the taxa abundance inference.

Finally, by using MaAsLin2^42^ we determined the association among taxa and the sampling matrix by controlling confounding factors such as age, gender and also taking into account the hierarchical structure of samples (i.e. non independency of paired samples). 12 out of 13 taxa that were significantly related (adjusted p ≤ 0.05) to sampling matrices (Table 1) were negatively associated with feces, indicating a lower probability to observe them in fecal samples. In particular, taxa belonging to the Campilobacterota phylum were more abundant in CWF samples (0.34% ± 1.27%) compared to feces (0.0003% ± 0.0013%). Among the MaAsLin2 returned taxa, *Prevotella* was one of the most observed in both the sampling matrices but negatively associated with feces. The genus *Granulicatella* resulted the only positively associated with feces (+ 2.72), according with the prevalence, in term of abundances, observed more frequently in feces (7 out of 30) than in CWF (3 out of 30), with a mean relative abundance around 0.01% ± 0.02%. Comparing these results with those based on Venn Diagrams and prevalence, no relevant associations were observed, supporting the evidence that the two sampling matrices were able to recall a similar microbiome, but with differences in taxa abundances.

**Table 1:** The taxa obtaining a relevant association to sampling matrix are listed. For each taxa the taxonomic rank (rank), the scientific name (feature), the reference value for association (value), the association (Association) and the obtained adjusted p-value (q-val) are listed.

Rank	Feature	Value	Association	q-val
Phylum	Campilobacterota	F	− 5.62	0.0004230
Class	Campylobacteria	F	− 5.03	0.0012278
Order	Campylobacterales	F	− 4.80	0.0017057
Family	*Campylobacteraceae*	F	− 4.87	0.0033904
Genus	*Anaerococcus*	F	− 5.11	4.72E-05
Genus	*Prevotella*	F	− 5.64	0.0001257
Genus	*Campylobacter*	F	− 5.70	0.0181270
Genus	*Granulicatella*	F	+ 2.72	0.0198773
Genus	*Peptoniphilus*	F	− 4.60	0.0446622
Genus	*Porphyromonas*	F	− 6.80	0.0446622
Species	*Peptoniphilus lacrimalis*	F	− 5.90	0.0041334
Species	*Campylobacter hominis*	F	− 6.82	0.0293966
Species	*Prevotella disiens*	F	− 7.83	0.0484327

## Discussion

Understanding the role of the gut microbiota in the well-being of the human host is a constantly moving field. Although several studies have shown that the gut microbiota offers many benefits to the host whereas dysbiosis is actively involved in the development of a wide variety of diseases, the choice of the most appropriate sampling method to properly investigate the gut microbiome is still challenging. Currently, sampling procedures for gut microbiome investigation are mainly focused on the collection of feces. Vaga et al. make it clear that stool samples provide a good approximation of gut microbiota but they cannot entirely describe in detail the complexity and the biodiversity of the different niches characterizing the GI tract^31^. Biopsy of different intestinal portions revealed specific microbial niches, exhibiting some differences between colon mucosa and stool profiles. In recent years, endoscopic colonic lavage was proposed as a new approach for gut microbiome investigation able to provide observations similar to colon biopsy. As in biopsy, the collection of endoscopic colonic lavage samples is performed in patients needing colonoscopy and requiring an appropriate bowel preparation the day before treatment including the laxatives administrations.

In our study we compared two different sampling procedures to investigate the human microbiome: feces (F) and colon washing feces (CWF). In CWF procedure, a bowel preparation with laxatives is not required, reducing the impact on microbiome analysis and making it a promising approach. Nonetheless CWF is still invasive for the patient, considering the use of a rectal catheter required to inject and collect the large amount of filtered and warm physiological solution (18–20 L) necessary to achieve a complete colon cleansing.

Obviously, taking into account the length and complexity of the CWF procedure, it cannot be proposed as a substitute of fecal sampling, but it can be used as a complementary approach to better elucidate the microbiome composition.

To the best of our knowledge, a comparative analysis between fecal and colon washing fecal samples has never been carried out, so we performed a 16S rRNA metabarcoding study on paired F and CWF samples with the purpose to verify the relative suitability of these matrices in representing the complexity of human gut microenvironment. In our study, we enrolled a cohort of 30 volunteers who were asked to first collect fecal samples and then to undergo the colon washing treatment. We investigated the gut microbiome using a deep metabarcoding approach that selectively amplifies the hypervariable region V4 of the bacterial 16S rRNA gene.

We began our investigation by evaluating whether the total microbial load observed by using the two sampling matrices could be different. Quantification of 16S rRNA gene copies using ddPCR showed no statistically relevant differences between F and CWF samples. Then, we analyzed metabarcoding data focusing on alpha and beta diversity. The intra-sample diversity survey relying on Inverse Simpson index measurement highlighted that CWF was able to catch more biodiversity than feces. On the other hand, regardless of the metric applied to inspect the beta-diversity (i.e. weighted and unweighted UniFrac), the observed variability was mainly dominated by individual peculiarities, although paired samples generally tended to co-cluster, with few exceptions. Arguably, this could be due to confounding factors, such as diet or stressful conditions, lifestyle and others, which may affect the gut microbial composition^8,9,11,43^. As a first result, our data emphasize the strong individual-specificity of the gut microbiome, independently of the type of sample analyzed. This is in line with the most recent evidence suggesting the strong uniqueness of gut microbiota, which can be affected by many factors, such as age, gender, lifestyle, and diet. For example, Wilmanski et al. identified distinct signatures in the gut microbiome of a cohort of 9000 adults of different ages, proving that age is one of the crucial factors for the specificity of the gut microbiota, and that the gut microbiome becomes increasingly divergent from others by the middle of adulthood^7^. Moreover, the comparison among the measured distance in matched (i.e. samples from the same individual) and random sample pairs, demonstrated that F and CWF samples from the same individual tended to be more similar compared to F and CWF samples from different subjects. However, F and CWF generally were able to recall the same microbiome but with differences in taxon abundances.

Next we focused on the retrieved taxonomic information. In particular, we observed that most of the detected microorganisms (> 95%) in both F and CWF sample matrices were members of the Bacteroidota*,* Firmicutes and Proteobacteria phyla, which is concordant with the literature about the human gut microbiome^30,31^. The Gram-negative bacteria belonging to the Bacteroidota phylum are common, abundant, and diverse within the human GI tract. They perform the metabolic conversions essential for the host, often related to the degradation of proteins or complex carbohydrates^44^. In particular, the majority of the GI *Bacteroidetes spp.* identified in our study belongs to the *Bacteroidaceae*, *Prevotellaceae* and *Rikenellaceae* families which produce succinic acid, acetic acid and propionic acid as the main final products^44^. Several species of *Bacteroides* are dominant bacteria which produce and offer nutrition and vitamins for both the host and other intestinal microbial residents. Depending on the location in the host, some species of *Bacteroides* may be beneficial in the intestine but pathogenic opportunistic in other locations of the body. *Bacteroides vulgatus* and *Bacteroides fragilis* were isolated from patients suffering from Crohn’s disease, in addition *Bacteroides fragilis* was associated with intra-abdominal abscesses, appendicitis, and IBD. Moreover, as thoroughly reviewed by Marzano et al., 2021^21^, the well-established interaction between some species, including those belonging to *Bacteroides* genus, and the colorectal cancer, points up the importance of identifying gut microbiota biomarkers with diagnostic, prognostic or predictive significance, as well as the possibility of intestinal microbiota modulation to prevent cancer or enhance the effect of specific therapies.

The second component of the GI microbiota that we found abundant in both F and CWF samples was the Firmicutes phylum which is known to dominate the butyrate biosynthesis (mainly due to *Faecalibacterium prausnitzii*). Within this phylum, the most abundant GI microorganisms were members of the *Ruminococcaceae* and *Lachnospiraceae* families, belonging to the Clostridia class. The prominent representation of these functional groups among the subjects analyzed was in agreement with the role of these bacteria in the maintenance and protection of normal colonic epithelium and in the production of regulatory T (Treg) cells^45,46^.

As regards bacteria belonging to the Proteobacteria phylum, they are commonly detected in the human microbiota and this group of Gram-negative bacteria is particularly large (six classes), although not very abundant in the GI tract. In fact, all Proteobacteria account for about 4% of the total gut microbiota in healthy people^47^. In our study, we found Proteobacteria as the third most abundant phylum, particularly rich in taxa belonging to the family of *Enterobacteriaceae*. Several studies showed that an increased prevalence of the Proteobacteria phylum is a marker of dysbiosis^47,48^.

The major novelty of this study is the comparative analysis of two different sampling matrices from the same individual that allowed us to highlight the variability at intra and inter individual level for the gut microbiome. Focusing on the taxonomic composition of matched F and CWF samples collected by the same individual, we generally found greater similarity between matched samples, proving that the composition of gut microbiota was similar in matched samples from the same individual. Interestingly, we observed the similarity rate widely changed across different subjects, suggesting a large inter-individual variability among all participants of our study. A relevant overlap between community composition among F and CWF samples was also revealed by Venn diagrams. Furthermore, we identified unique taxa in F and CWF samples, underlining that F samples revealed taxa that we did not observe in CWF samples and vice versa. Interestingly, we again noticed that the number of common taxa as well as the number of uncommon taxa were unique for each matched sample. This, again, highlighted the considerable inter-individual variability across all individuals. Moreover, by prevalence analysis we proved that no taxa were matrix-specific but most of them were shared. Finally, association analysis among taxa and sampling matrix by taking into account also confounding factors (i.e. gender and age) and the hierarchical structure of the data, retrieved 13 taxa with a lower probability to be observed in feces compared to CWF. In particular, we found *Prevotella* associated with CWF samples. A recent review by Gabriela Precup and Dan-Cristian Vodnar analyzed the possibility of using *Prevotella* as a potential biomarker for homeostasis or disease state through its metabolite signature^49^. Nonetheless, the main difference observed between the tested matrices remained restricted to taxa relative abundances.

In conclusion, we have generated a first overview of the microbiome composition among paired F and CWF samples. Our preliminary diversity analysis confirmed that both sampling approaches are capable of representing a good approximation of the human gut microbiome and suggest that both F and CWF samples could be independently used for the study of the human microbiome. While further studies based on larger and independent cohorts of patients and healthy subjects are needed, our encouraging results make conceivable that the use of both types of sampling matrices may represent a possible choice to obtain a more complete view of the human gut microbiota. Nowadays, new therapeutic approaches of modern medicine are particularly focused on precision medicine, and the accurate individual-specific characterization of the gut microbiome appears to be one of the most interesting aspects for future research aimed at an appropriate treatment of diseases^24,50^. In this perspective, we show here the usefulness of an in-depth analysis of gut microbiome where both F and CWF can represent two sides of the coin for investigating the human gut microbiome and unraveling individual-specific differences.

## Methods

### Subject recruitment and sampling procedures

A total of 30 subjects were recruited from PoliSmail—Integrated Biomedical Services for Allergic and Immunological Diseases (Lecce), following written informed consent. Age, gender and clinical metadata were considered as clinical characteristics of enrolled participants (Supplementary Table S1).

From each participant, two different samples were collected: feces (F) and colon washing feces (CWF). The day before the colon washing procedure, 1 g of F samples/participants was collected in a DNA/RNA Shield™ Fecal Collection tube (Zymo Research, USA) and resuspended to obtain a semi-liquid consistency (Supplementary Figure S10A). The device is prefilled with a DNA/RNA Shield solution, which allows the homogenization of the sample and the preservation of nucleic acid. The tube was stored at 4 °C until the DNA extraction.

Colon washing procedure, also called colonic hydrotherapy, involves flushing the colon from rectum to the cecum with physiological, filtered and temperature-regulated warm water (25–30 L). The procedure was performed without preliminary bowel preparation and laxatives. An anatomic single-use speculum, connected to the medical equipment through a device that allows the water flow, was inserted into the patient’s rectum. The medical equipment works at physiological pressures producing water at controlled temperature (37 °C – 38 °C). During the procedures, an abdominal massage was performed to relax contractions and reactivate peristalsis. The entire session generally takes around 30 min from start to finish. After the colonic hydrotherapy, the fecal matter and the liquid used during the procedure was flushed out of the body and, about 10 mL, were collected in a sterile tube. Finally, 1 mL of each CWF sample was inserted in a DNA/RNA Shield™ Fecal Collection tube, homogenized and stored at 4 °C until the DNA extraction (Supplementary Figure S10B).

### DNA Extraction From feces and colon washing feces

Before the DNA extraction, the devices containing resuspended F and CWF samples were mixed by gentle inversions at least 3–4 times to further homogenate the contents of the entire container. 500 uL of F and CWF samples were recovered and used for the DNA extraction carried out by the FastDNA™ Spin Kit for Soil (MP Biomedicals, Santa Ana, CA, USA), according to the manufacturer instructions. A 40 s bead-beating step at speed 6 was executed on the FastPrep Instrument (BIO 101, Carlsbad, Canada). The DNA was eluted in a 100-μL volume of sterile water and stored at -20 °C. DNA quality was evaluated using the Agilent TapeStation 2200 System (Agilent, Santa Clara, CA, USA) with Genomic DNA ScreenTape assay (Agilent, Santa Clara, CA, USA). The DNA Integrity Number (DIN) was assigned to each sample by applying Agilent 2200 TapeStation software (controller version A.01.05). Then, quantitative fluorimetric analysis was carried out using Quant-iTTM PicoGreen® dsDNA Assay Kit (Invitrogen, Carlsbad, California) on a NanoDrop 3300 Fluorospectrometer (Thermo Fisher Scientific, Waltham, MA, USA).

### ddPCR experiments

Digital PCR was performed to determine the total number of 16S copies by using universal primers targeting the V5–V6 regions of 16S rDNA (primer sequences: forward, B-V5: 5′- ATTAGATACCCYGGTAGTCC-3′; reverse, A-V6: 5′-ACGAGCTGACGACARCCATG-3′), according to established procedures^51^. Absolute quantification was performed using QuantaSoft version 7.4.1 software (Bio-Rad, Hercules, CA, USA,) and the negative/positive thresholds were set manually, excluding samples with a number of droplets < 10 000. Output results were expressed in 16S copies µl^−1^.

### 16S rRNA Amplicon Sequencing

The V4 hypervariable region of the bacterial 16S rRNA gene was chosen as the target for prokaryotic identification and the amplicon library was achieved according to established procedures^52–54^. The V4 region was amplified using 0.5 ng of DNA extracted from F and CWF and the universal primer pairs 515F and 806R (underlined nucleotides in the following sequences) designed to contain from 5′ to 3′ ends the transposon Nextera’s sequences (Nextera DNA sample preparation guide, Illumina): 515F: 5′- TCGTCGGCAGCGTCAGATGTGTATAAGAGACAG/GTGCCAGCMGCCGCGGTAA-3′, and 806R: 5′- GTCTCGTGGGCTCGGAGATGTGTATAAGAGACAG/GGACTACHVGGGTWTCTAAT-3′.

All PCRs were done in the presence of a non-template-control PCR reaction (negative control). The PCR products were purified using the AMPure XP Beads at a concentration of 0.8 vol/vol (Agencourt Bioscience Corporation, Beverly, Massachusetts) employing the Hamilton Microlab STAR Liquid Handling System (Hamilton Company, Reno, NV, USA). Obtained amplicon libraries (around 420 bp long) were then quantified by the Quant-iTTM PicoGreen® dsDNA Assay Kit (Invitrogen, Carlsbad, CA, USA) using the NanoDrop™ 3300 Fluorospectrometer (Thermo Fisher Scientific, Waltham, MA, USA).

Equimolar ratios of the purified amplicons were pooled and subjected to 2 × 250 bp paired-end sequencing on the MiSeq platform (Illumina, San Diego, CA, USA). To increase the genetic diversity, as required by the MiSeq platform, 25% of phage PhiX genomic DNA library was added to the mix and co-sequenced.

### 16S rRNA Sequencing processing

Raw 16S rRNA sequences data were quality checked by using FastQC (Available online: https://www.bioinformatics.babraham.ac.uk/projects/fastqc/) and multiQC^55^. Illumina adapters and PCR primers were removed from raw reads by applying cutadapt^56^ and the retained PE reads were merged by using PEAR^57^. Then, the obtained merged amplicons were denoised into ASVs (Amplicon Sequence Variants)^58^ by applying DADA2 (version 1.10.1)^59^. ASVs were taxonomically annotated by using BioMaS (Bioinformatic analysis of Metagenomic amplicons)^60^ and using the release 138 of the SILVA database^61^. Contaminant ASVs were identified by using decontam^62^ and those assigned to chloroplast or mitochondria were removed from subsequent analysis. The retained ASVs were multi-aligned by applying MAFFT^63^ and the obtained multiple sequence alignment (MSA) was masked by using DECIPHER^64,65^. A maximum-likelihood (ML) phylogenetic tree based on the masked MSA was obtained in Fasttree 2^66^. The R packages phyloseq (1.26.1)^67^ and vegan (2.5–6)^68^ were used to measure alpha and beta diversity. For this purpose, ASVs counts were normalized to an equal depth of 68,000 sequences, by using rarefaction. In particular, the rarefaction depth was chosen by considering the rarefaction curves (Supplementary Figure S11). The Inverse Simpson index was used as measures of alpha diversity (i.e. intra-sample diversity) and it is a richness measure that is not influenced by the sampling depth, such as Shannon index is. Statistical difference in Inverse Simpson Fecal and CWF distributions was measured by using the paired Student’s t-test (p < 0.05 was considered as statistically significant) after verifying that both the data distributions were normal (Shapiro–Wilk test). General Linear Model (GLM) were obtained by using the R package lmer4^69^. Also the Observed ASVs and the Pielou Evenness indexes were considered as alpha diversity metrics and compared by using the paired t-test.

The unweighted and weighted UniFrac^70^ dissimilarity matrices were used to measure the beta diversity (i.e. inter-sample diversity). The weighted UniFrac is a quantitative metric taking also into account the relative frequencies of the observed ASVs, whereas the unweighted UniFrac is a qualitative measure which considers just ASVs presence or absence. The PERMANOVA (Permutational analysis of variance) was measured to infer the explained variability in beta diversity data by applying 999 permutations. Beta diversity inference on data aggregated according to the inferred taxonomy were obtained by using the Bray–Curtis and the Jaccard metrics. Comparison of inter-sample distances among paired and random samples was performed by using two tailed Student’s t-test.

Microbial networks were inferred using the NetCoMi^40^ framework. The ASVs counts were normalized by mclr (modified centered log-ratio transformation) and the networks were estimated using SPRING^71^, which converts the estimated partial correlations into dissimilarities via a “signed” distance metric. The inferred similarities were used as edge weights. Node clusters were inferred using the fast-greedy algorithm. Hub nodes were identified taking into account the following centrality properties: degree (number of adjacent nodes), betweenness (ability of the network to connect sub-networks), closeness (a measure of how close a node is to other nodes) and Eigenvector (a node is central if the connected nodes are also central). Nodes obtaining the highest centrality values were considered hubs. Networks comparison was performed in NetCoMi by using 1000 permutations. Clustering agreement was measured by using the Adjusted Rand Index (ARI), ranging between -1 (clustering disagreement) and 1 (clustering agreement).

Association among microbial taxa and sampling matrix were obtained by using the MaAsLin2 (Microbiome Multivariable Association with Linear Models) R package^42^. Microbial counts were normalized by CSS (cumulative sum squared) and used as a response variable in the regression model generated by the “NEGBIN” approach. Sampling Matrices were used as explanatory variable and adjusted for age, gender (fixed effect) and subject (random effect). The obtained p-values were adjusted by using the Benjamini & Hochberg method.

### Ethical statements

All experimental protocols were approved by the Research Committee of the Department of Biosciences, Biotechnology and Biopharmaceutics of the University of Bari “A. Moro”, and all methods were carried out in accordance with relevant guidelines and regulations.

## Supplementary Information

Supplementary Information.

## Data Availability

Sequencing raw data and supporting metadata are available on SRA under the BioProject PRJNA792991.
