# Supplementary material for: Natural and after colon washing fecal samples: the two sides of the coin for investigating the human gut microbiome
Source: Sci Rep. 2022 Oct 25;12:17909. doi: 10.1038/s41598-022-20888-z (PMC9596478; doi:10.1038/s41598-022-20888-z)
Supplement: Supplementary file 1 — Supplementary Information. [file 41598_2022_20888_MOESM1_ESM.pdf]

SUPPLEMENTARY MATERIALS

Natural and after colon washing fecal samples: the two sides of the coin for investigating the human gut microbiome

Elisabetta Piancone[1], Bruno Fosso[2], Marinella Marzano[2], Mariangela De Robertis[1], Elisabetta Notario[1], Annarita Oranger[1], Caterina Manzari[1], Silvia Bruno[1], Grazia Visci[2], Giuseppe Defazio[1], Anna Maria D'Erchia[1,5], Ermes Filomena[1], Dominga Maio[3], Martina Minelli[3], Ilaria Vergallo[3], Mauro Minelli[3,4] and Graziano Pesole[1,2,5*]

[1]Department of Biosciences, Biotechnology and Biopharmaceutics, University of Bari 'Aldo Moro', 70126 Bari, Italy
[2]Institute of Biomembranes, Bioenergetics and Molecular Biotechnologies, Consiglio Nazionale delle Ricerche, 70126 Bari, Italy
[3]Specialistic Allergic Unit & Immunological Pathologies, PoliSmail Network, 73100 Lecce, Italy
[4]Centro Direzionale Isola F2, Pegaso Online University, 80132 Naples, Italy
[5]Consorzio Interuniversitario Biotecnologie, 34100 Trieste, Italy

[*]Corresponding author: graziano.pesole@uniba.it

List of Supplementary Materials

Figures

Figure S1. Dumbbell plot of alpha diversity, measured using the Observed ASVs (A) and Pielou evenness index (B), for matched F and CWF samples.

Figure S2. PCoA plot based on Weighted UniFrac dissimilarity matrices. F samples points are depicted as circles, CWF samples are triangles. Each subject is depicted in a single colour.

Figure S3. PCoA plot based on Unweighted UniFrac dissimilarity matrices. F samples points are depicted as *circles*, CWF samples are triangles. Each subject is depicted in a single colour.

Figure S4. Per individual Venn Diagram for Families.

Figure S5. Per individual Venn Diagram for Genera.

Figure S6. Per individual Venn Diagram for Species.

Figure S7. Scatterplot representing the taxa prevalence in F samples (x-axis) against the number of times the taxa are exclusively observed in F (y-axis). Dot colour is equal to the difference among F and CWF prevalence (score).

Figure S8. Scatterplot representing the taxa prevalence in CWF samples (x-axis) against the number of times the taxa are exclusively observed in CWF (y-axis). Point color is equal to the difference among F and CWF prevalence (score).

Figure S9. Taxa prevalence in F and CWF samples at order, family, genus and species level.

Figure S10. Graphic description of the fecal (A) and colon washing feces (B) sampling procedures adopted in the study.

Figure S11. Rarefaction curves for F and CWF samples. The red line corresponds to the chosen rarefaction normalisation value.

Tables

Table S1. Clinical characteristics of enrolled participants.

Table S2. Summary of the GLM model used to estimate the Inverse Simpson alpha diversity as a function of age, gender (fixed effect), sampling matrix (fixed effect) and subject (random effect). For each explanatory variable the effects on the response variable were estimated with the Confidence Interval (CI) and the associated p-value. Finally, the estimation of the explained variability is available (R2).

Table S3. Summary of the PERMANOVA model applied on weighted and unweighted UniFrac dissimilarities as function of sampling matrix, gender, age and subject (random effect). For each the estimation of the explained variability (R2) and the associated p-values are shown.

Table S4. From phylum to species are shown the relative abundances of most abundant taxa (i.e. taxa with a relative abundance ≥ 1% in at least one sample). In particular, for each taxa the scientific name, the mean and standard deviation in both F and CWF are reported.

Table S5. Prevalence table. Taxa observed in CWF sample but not in the paired F one and *vice versa*, in at least one individual. For each taxa are listed the taxonomic path (Taxa), the number of times it is observed exclusively in F samples (Exclusively in F), the number of times it is observed exclusively in CWF samples (Exclusively in CWF), the number of times it is observed in F samples (Raw F count), the number of times it is not observed in F samples (Not Observed in F), the number of times it is observed in CWF samples (Raw CWF count), the number of times it is not observed in CWF samples (Not Observed in CWF), the taxonomic rank (rank), the prevalence score (Score), the F prevalence (F prev perc), the CWF prevalence (CWF prev perc) and the absolute score value (Abn Score).

Table S6. List of nodes represented in the F and CWF microbial networks and their attributes. For each node the following attributes are listed: 1) ASV: asv name; 2) F Network: cluster colour in F network; 3) CWF Network: cluster colour in CWF network: 4) Same Cluster: logical attribute indicating if the nodes are grouped in the same clusters in the two networks; 5) Taxonomy: ASV taxonomic classification; 6) Label: ASV label shortcut; 7) Hub: attribute indicating if the ASV is an hub (YES) or not (NO).

Figure S1

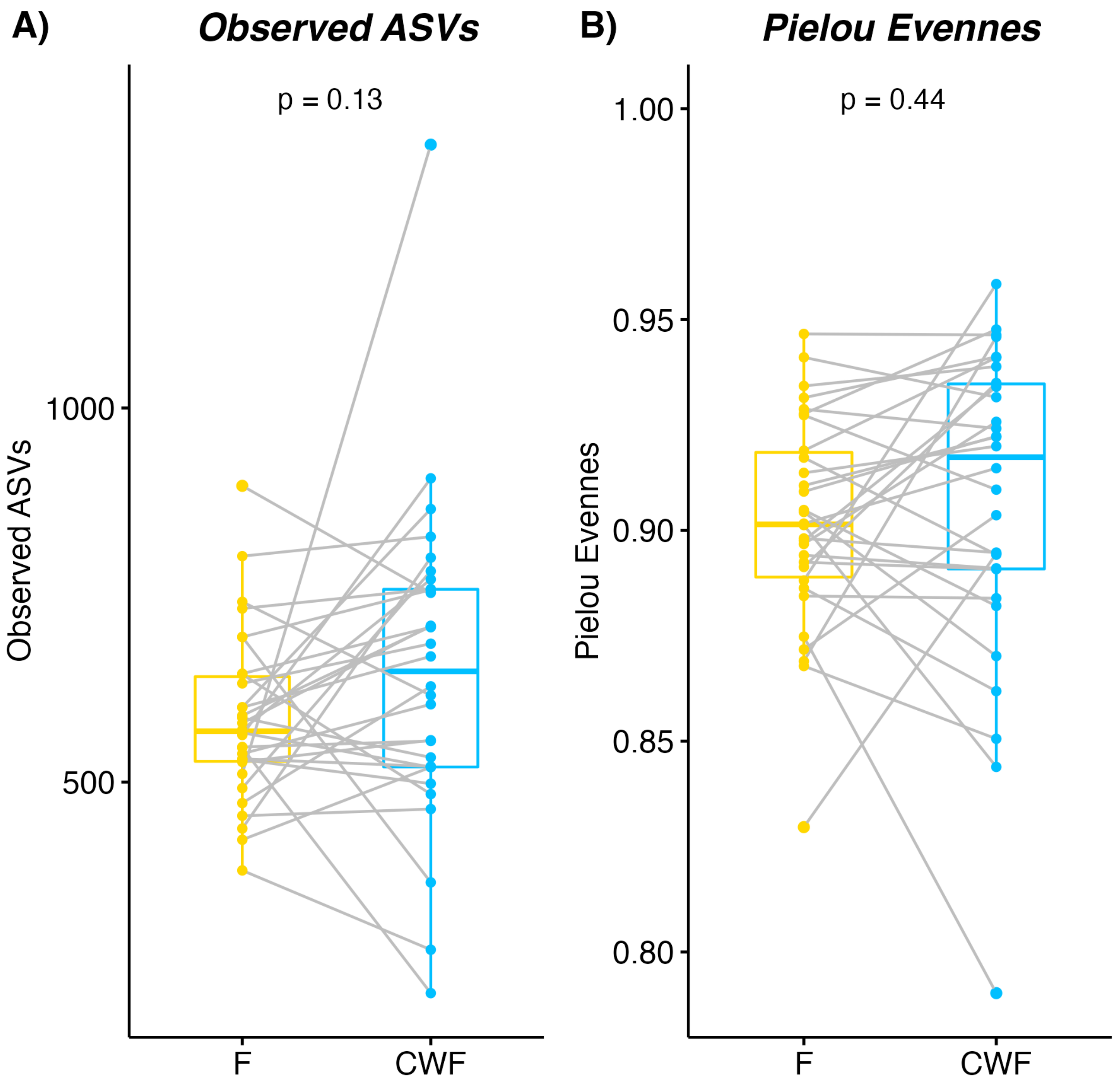

Figure S1. Dumbbell plot of alpha diversity, measured using the Observed ASVs (A) and Pielou evenness index (B), for matched F and CWF samples.

Figure S2

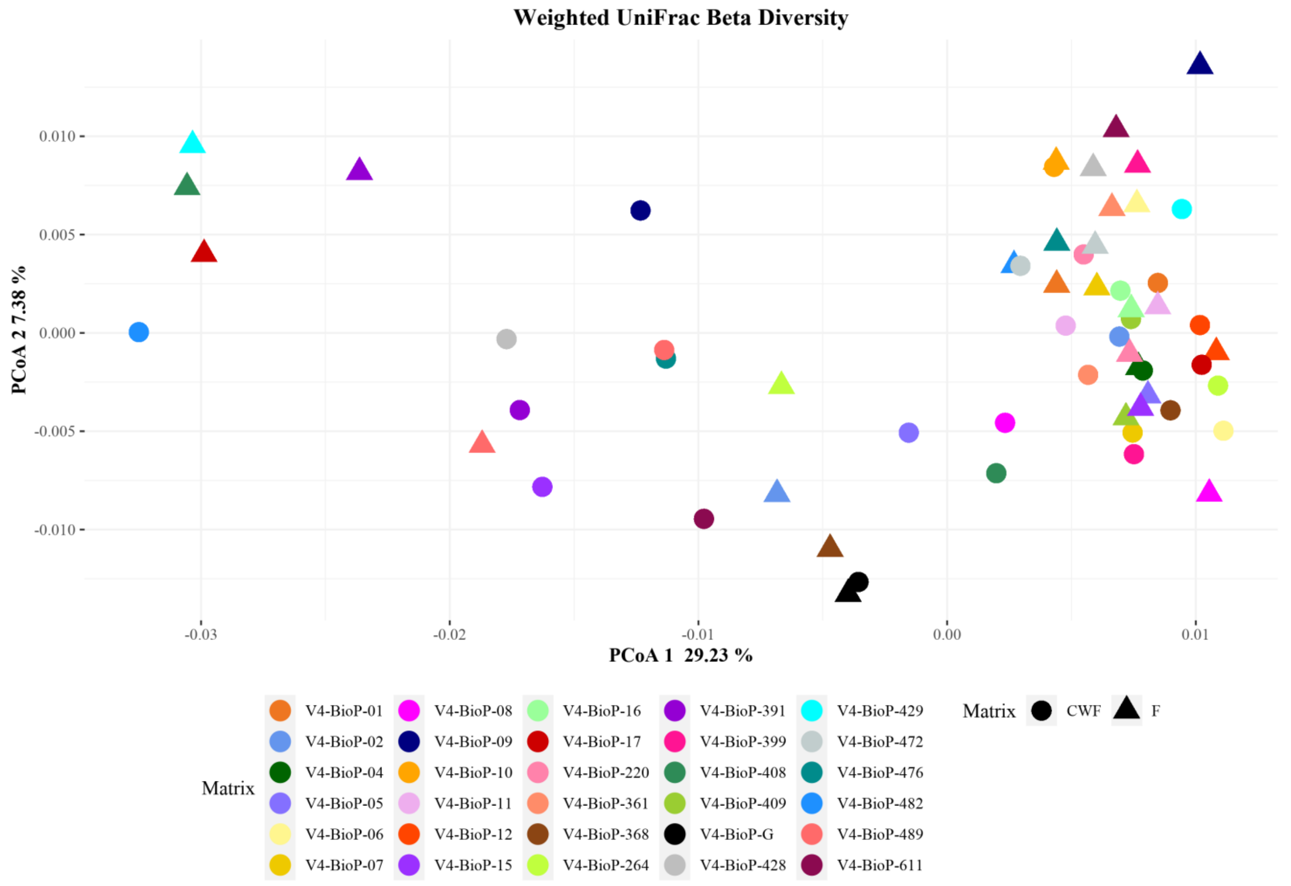

Figure S2. PCoA plot based on Weighted UniFrac dissimilarity matrices. F samples points are depicted as circles, CWF samples are triangles. Each subject is depicted in a single colour.

Figure S3

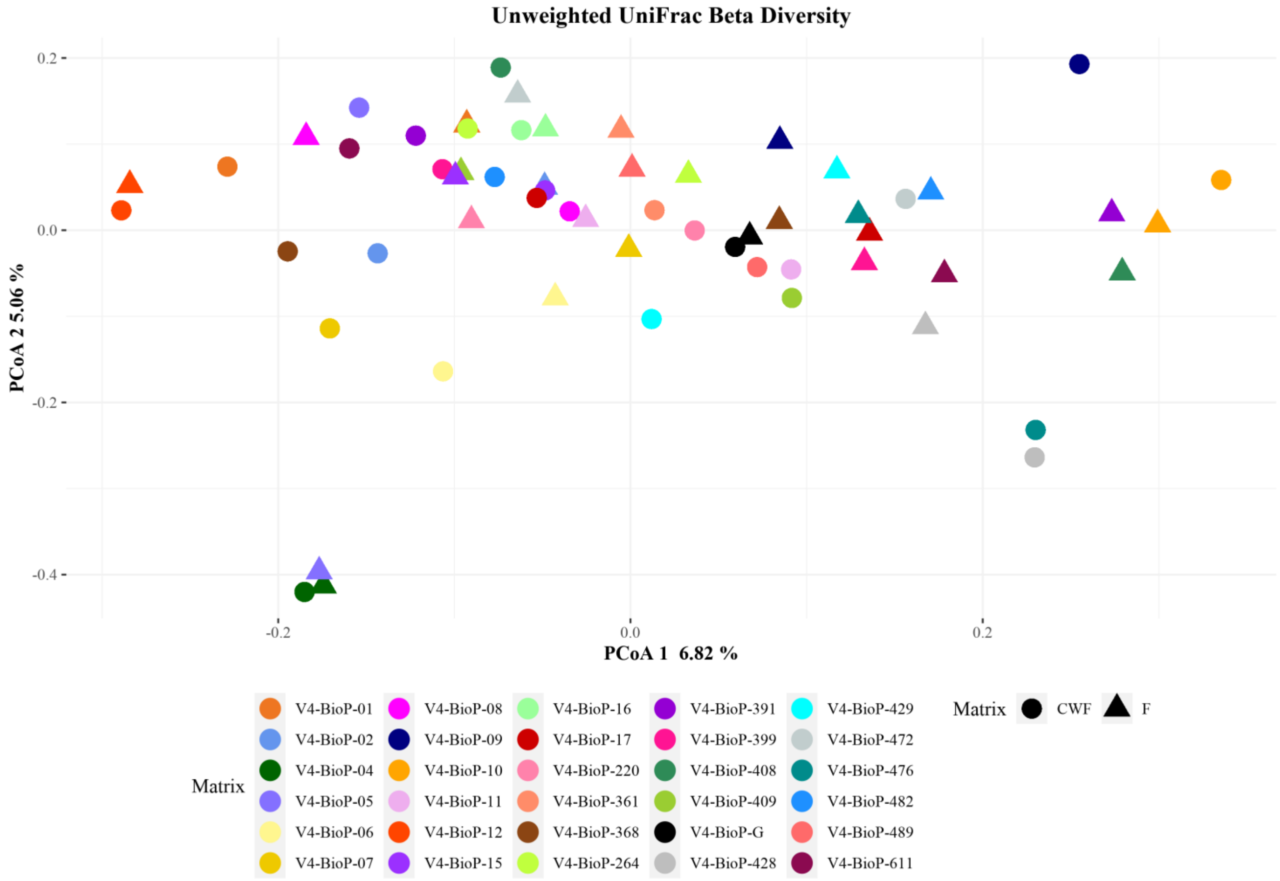

Figure S3. PCoA plot based on Unweighted UniFrac dissimilarity matrices. F samples points are depicted as *circles*, CWF samples are *triangles.* Each subject is depicted in a single colour.

Figure S4

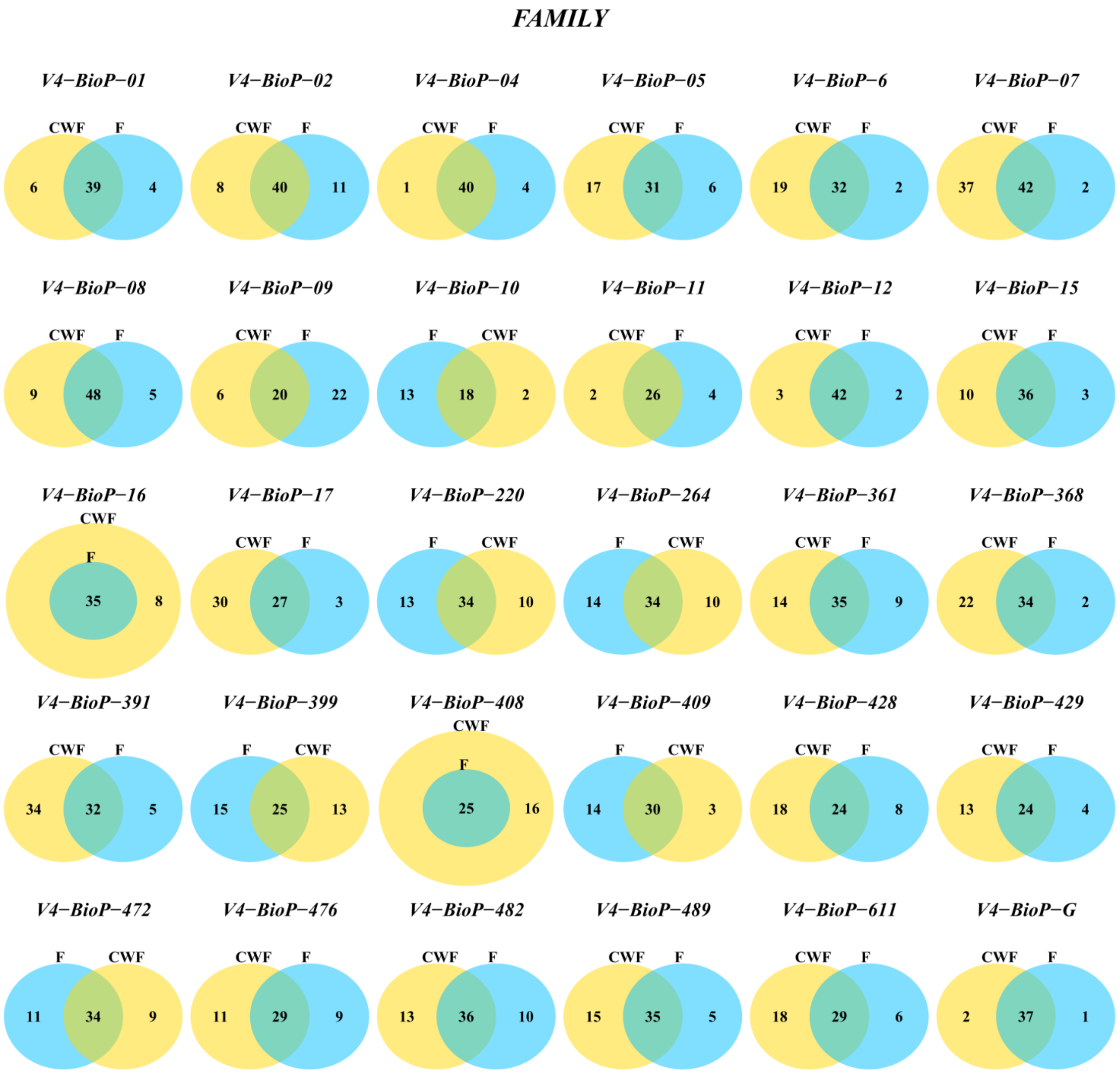

Figure S4. Per individual Venn Diagram for Families.

Figure S5

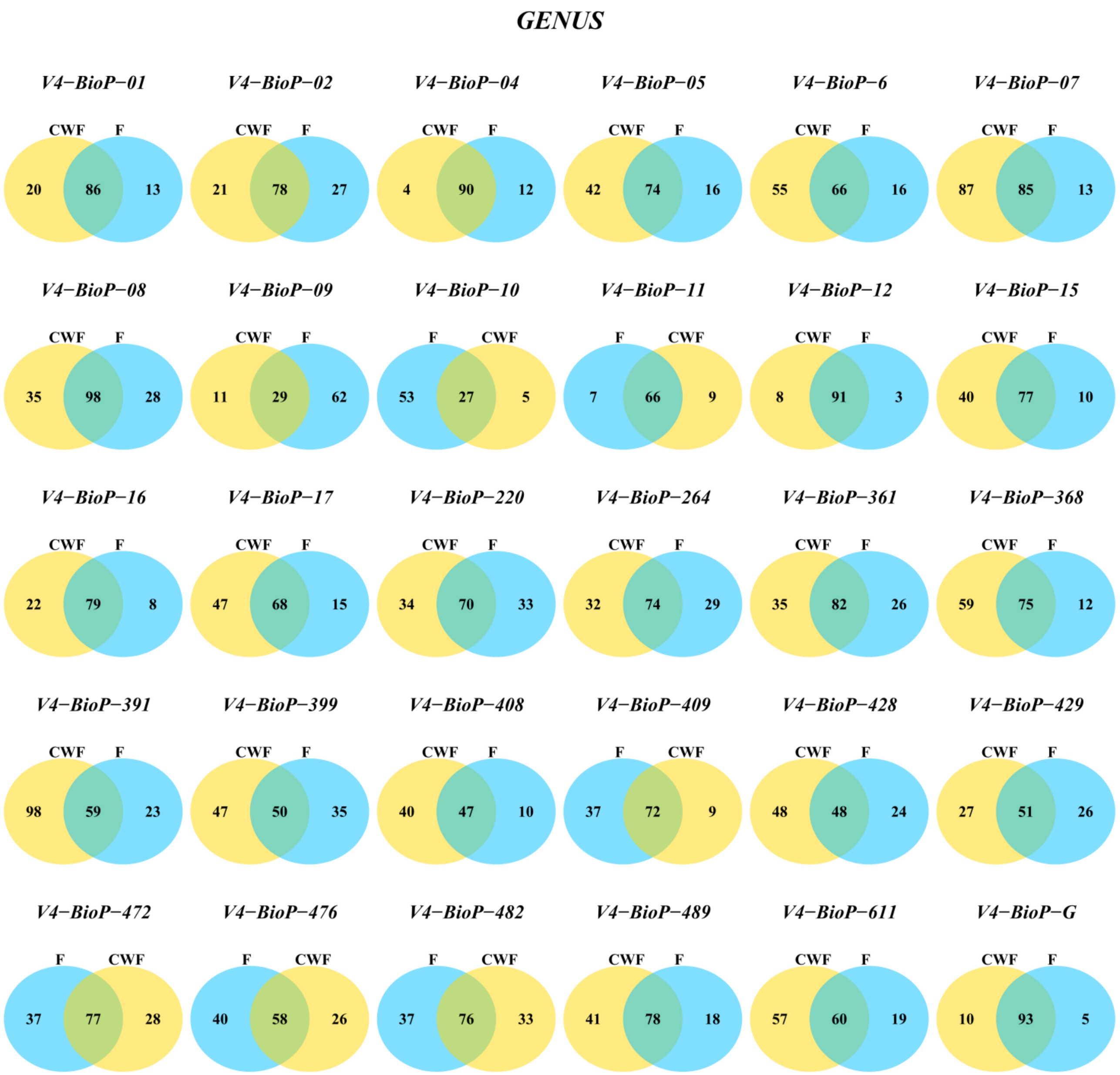

Figure S5. Per individual Venn Diagram for Genera.

Figure S6

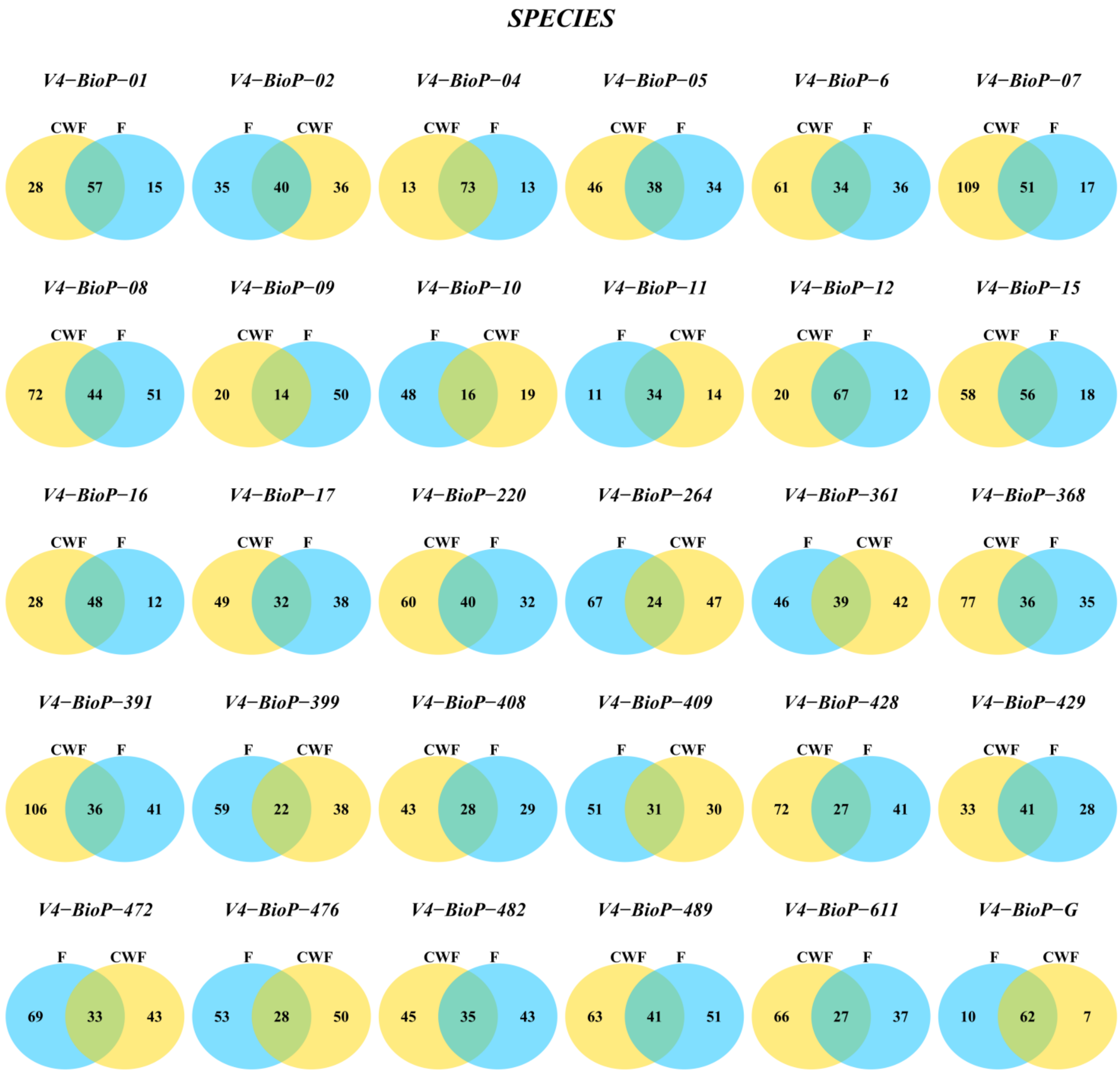

Figure S6. Per individual Venn Diagram for Species.

Figure S7

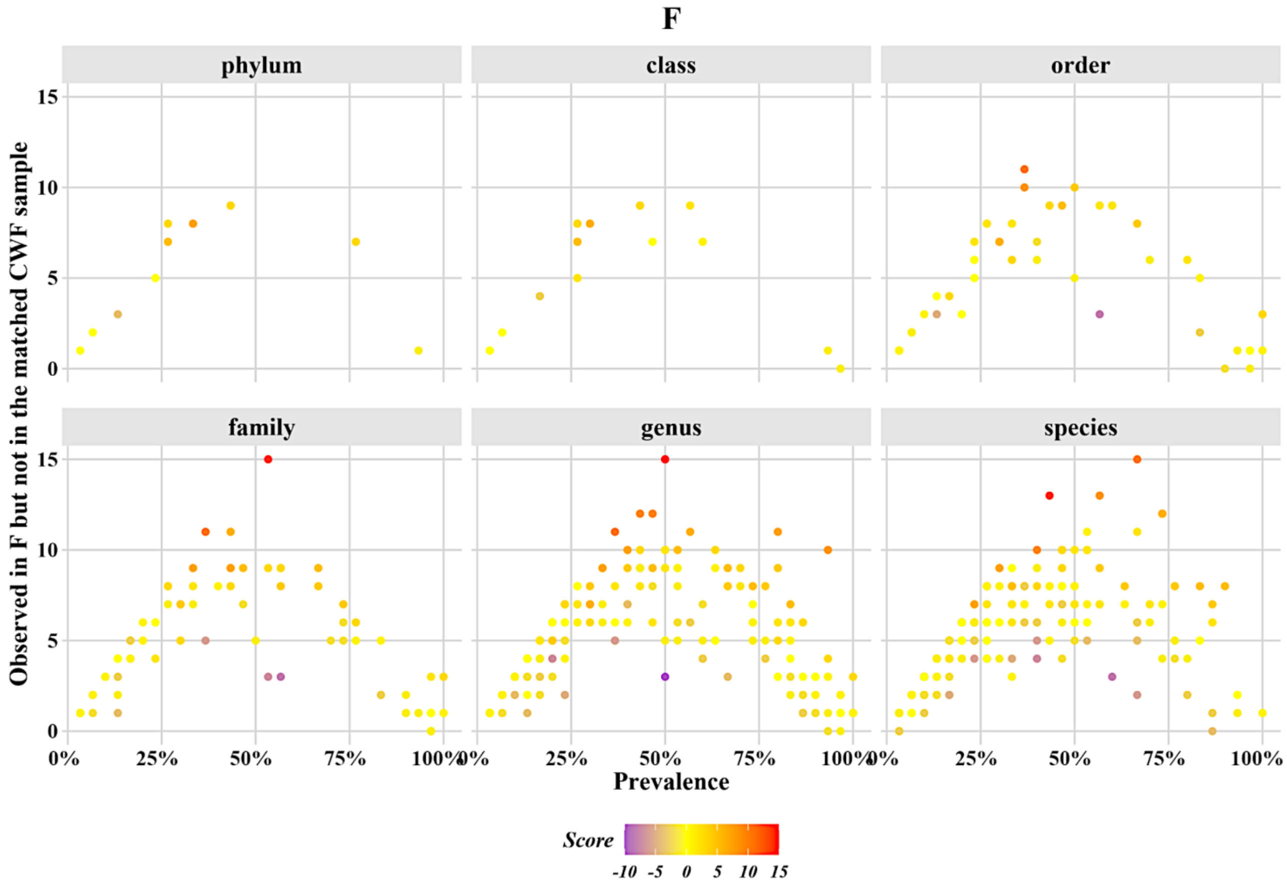

Figure S7. Scatterplot representing the taxa prevalence in F samples (x-axis) against the number of times the taxa are exclusively observed in F (y-axis). Dot colour is equal to the difference among F and CWF prevalence (score).

Figure S8

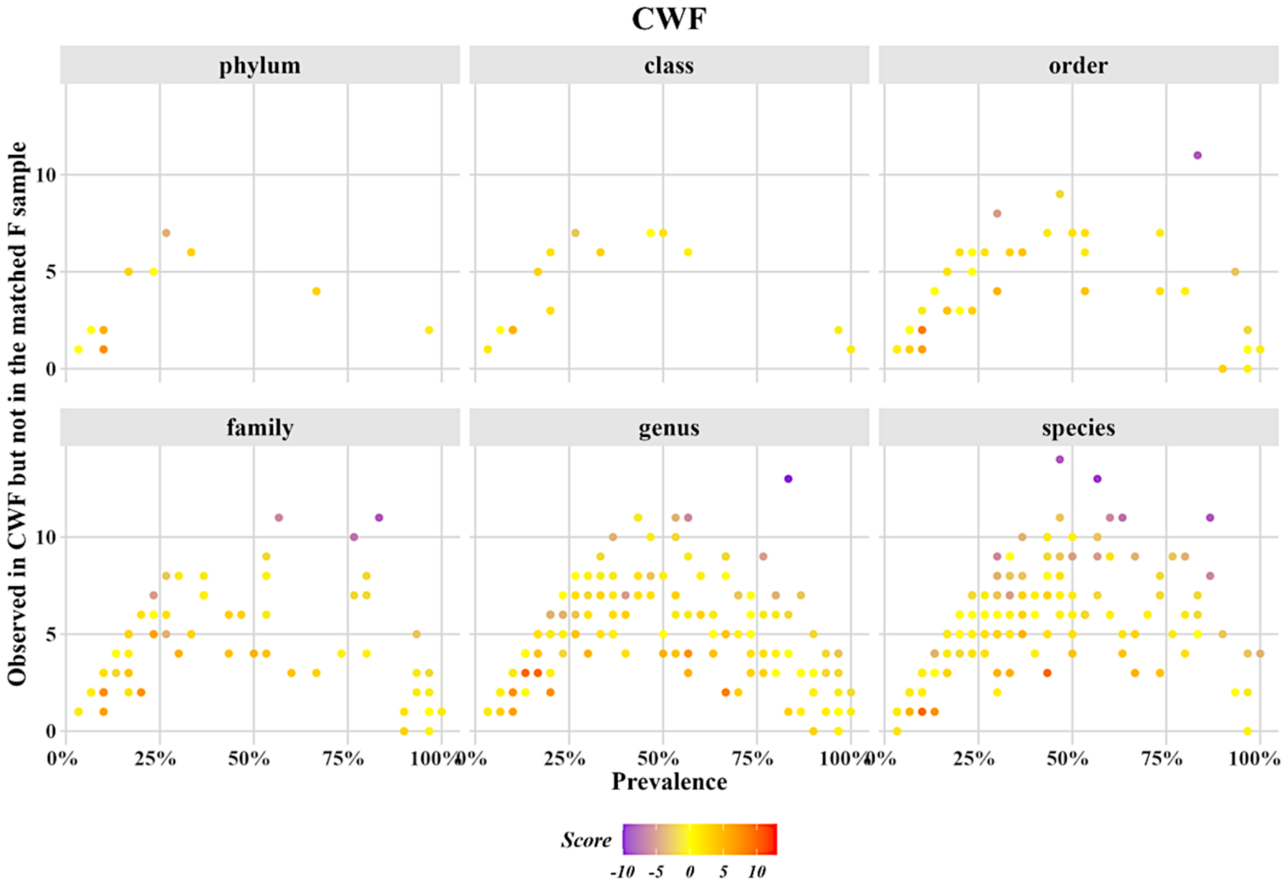

Figure S8. Scatterplot representing the taxa prevalence in CWF samples (x-axis) against the number of times the taxa are exclusively observed in CWF (y-axis). Point color is equal to the difference among F and CWF prevalence (score).

Figure S9

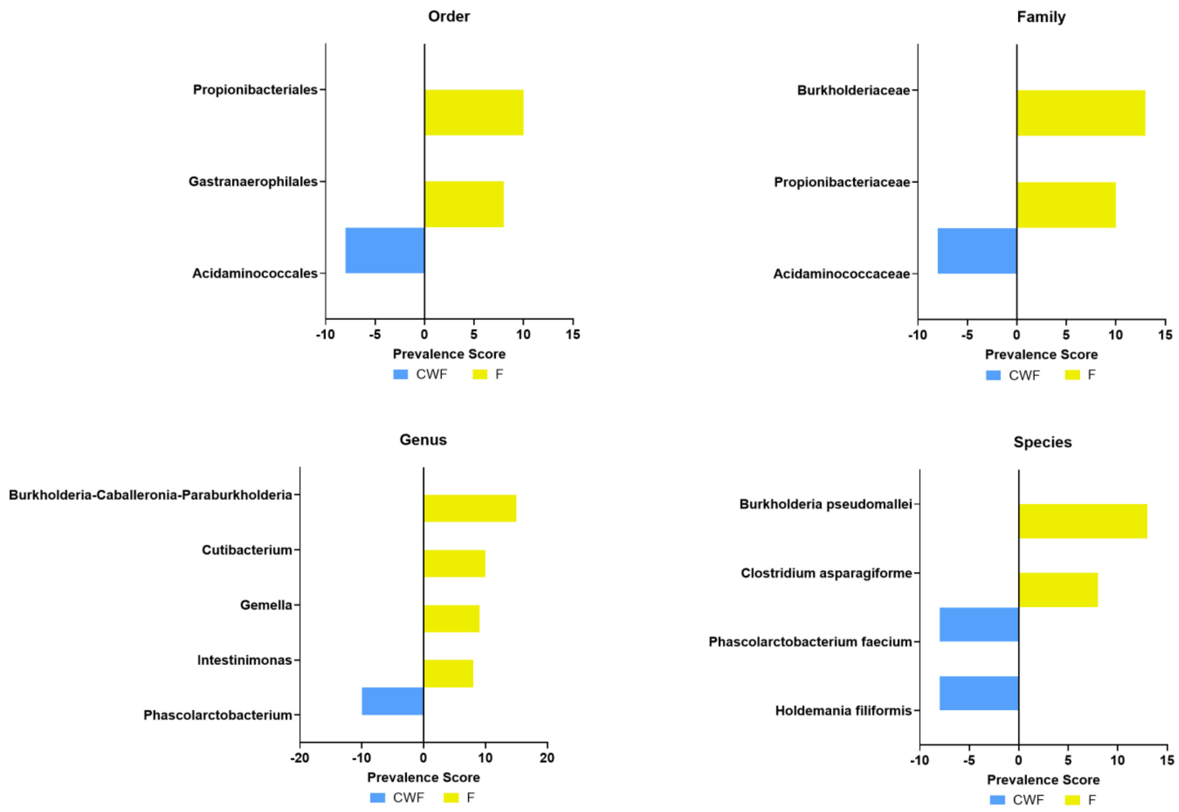

Figure S9. Taxa prevalence in F and CWF samples at order, family, genus and species level.

Figure S10

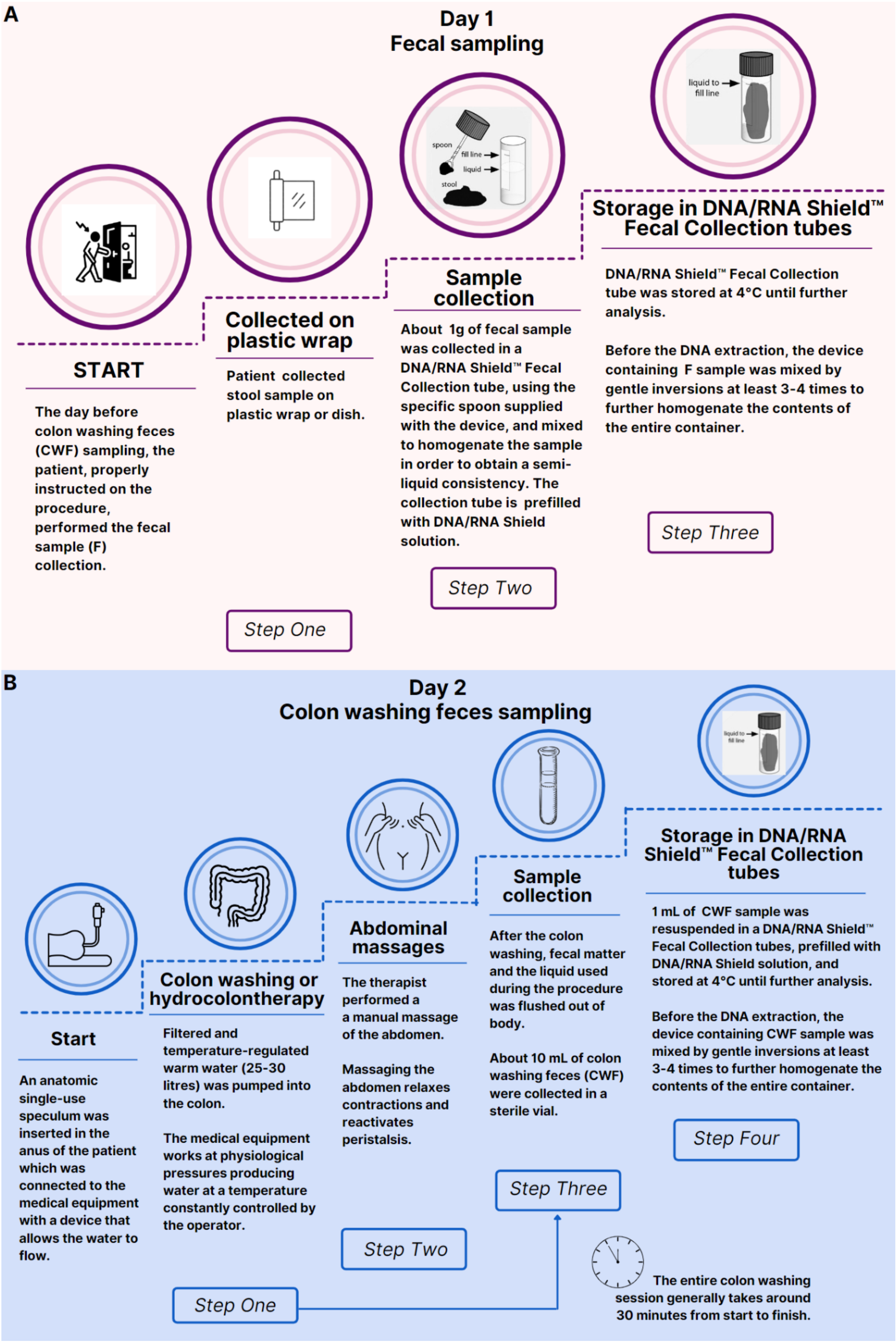

A
Day 1
Fecal sampling
START
The day before colon washing feces (CWF) sampling, the patient, properly instructed on the procedure, performed the fecal sample (F) collection.
Collected on plastic wrap
Patient collected stool sample on plastic wrap or dish.
Step One
spoon
fill line
liquid
stool
Sample collection
About 1g of fecal sample was collected in a DNA/RNA Shield™ Fecal Collection tube, using the specific spoon supplied with the device, and mixed to homogenate the sample in order to obtain a semi-liquid consistency. The collection tube is prefilled with DNA/RNA Shield solution.
Step Two
liquid to fill line
Storage in DNA/RNA Shield™ Fecal Collection tubes
DNA/RNA Shield™ Fecal Collection tube was stored at 4°C until further analysis.
Before the DNA extraction, the device containing F sample was mixed by gentle inversions at least 3-4 times to further homogenate the contents of the entire container.
Step Three
B
Day 2
Colon washing feces sampling
Start
An anatomic single-use speculum was inserted in the anus of the patient which was connected to the medical equipment with a device that allows the water to flow.
Colon washing or hydrocolontherapy
Filtered and temperature-regulated warm water (25-30 litres) was pumped into the colon.
The medical equipment works at physiological pressures producing water at a temperature constantly controlled by the operator.
Step One
Abdominal massages
The therapist performed a a manual massage of the abdomen.
Massaging the abdomen relaxes contractions and reactivates peristalsis.
Step Two
Sample collection
After the colon washing, fecal matter and the liquid used during the procedure was flushed out of body.
About 10 mL of colon washing feces (CWF) were collected in a sterile vial.
Step Three
Storage in DNA/RNA Shield™ Fecal Collection tubes
1 mL of CWF sample was resuspended in a DNA/RNA Shield™ Fecal Collection tubes, prefilled with DNA/RNA Shield solution, and stored at 4°C until further analysis.
Before the DNA extraction, the device containing CWF sample was mixed by gentle inversions at least 3-4 times to further homogenate the contents of the entire container.
Step Four
The entire colon washing session generally takes around 30 minutes from start to finish.

Figure S11

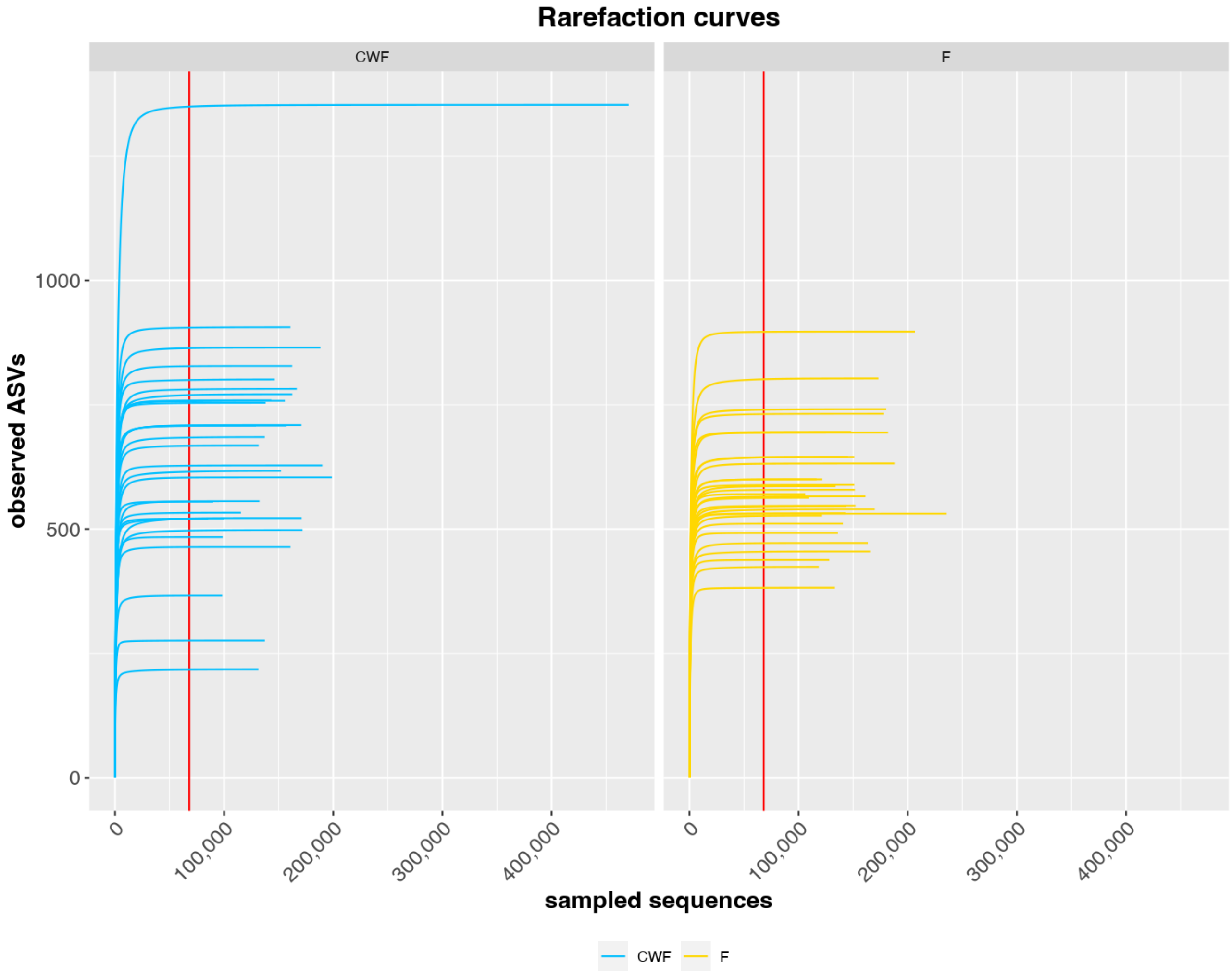

Figure S11. Rarefaction curves for F and CWF samples. The red line corresponds to the chosen rarefaction normalisation value.

Table S1

Subject (N=30)	Characteristics			
	**Sample-ID**	**Gender**	**Range of Age (yrs)**	**Clinical Metadata**
S1	V4-BioP-01	F	41-60	SNAS (Systemic Nickel Allergic Syndrome)
S2	V4-BioP-02	F	15-25	Vasculitis
S3	V4-BioP-04	M	15-25	Acute Hives; behavior Disorders
S4	V4-BioP-05	F	41-60	Mucosal hyperreactivity; poliabortivity
S5	V4-BioP-06	F	41-60	Mucosal hyperreactivity; fibromyalgia
S6	V4-BioP-07	F	41-60	Syncopal episodes
S7	V4-BioP-08	F	26-40	Chronic thyroid disease; migraine
S8	V4-BioP-09	M	41-60	Gastroesophagitis, dysmnesia and asthenia\adynamia
S9	V4-BioP-10	F	15-25	Vaginitis\recurrent cystitis; gastroesophagitis
S10	V4-BioP-11	M	15-25	Alopecia areata and metabolic syndrome
S11	V4-BioP-12	F	41-60	Chronic Hives and autoimmune thyroiditis
S12	V4-BioP-15	F	41-60	Non-alcoholic steatohepatitis (NASH)
S13	V4-BioP-16	F	26-40	Mucosal hyperreactivity and polyarthralgic syndrome
S14	V4-BioP-17	M	41-60	Metabolic syndrome
S15	V4-BioP-220	F	26-40	Acne and atopy
S16	V4-BioP-264	F	41-60	Irritable bowel syndrome (IBS); Anxiety-depressive syndrome
S17	V4-BioP-361	M	41-60	Irritable bowel syndrome (IBS); plantar fasciitis
S18	V4-BioP-368	M	26-40	COVID-19 and bronchial asthma
S19	V4-BioP-391	M	26-40	Mucosal hyperreactivity; Sjögren syndrome
S20	V4-BioP-399	M	15-25	Insulin-dependent diabetes
S21	V4-BioP-408	M	15-25	Alopecia and atopic dermatitis
S22	V4-BioP-409	F	41-60	Migraine; metabolic syndrome
S23	V4-BioP-428	F	41-60	Bronchial asthma; thyroiditis
S24	V4-BioP-429	F	41-60	SNAS-urticaria-angioedema
S25	V4-BioP-472	F	41-60	SNAS and recurrent stomatitis
S26	V4-BioP-476	F	15-25	Alopecia and chronic thyroiditis; dyslexia and dyscalculia
S27	V4-BioP-482	F	26-40	Celiac disease and SNAS
S28	V4-BioP-489	M	26-40	Atopic dermatitis
S29	V4-BioP-611	F	41-60	Recurrent Transient ischemic attack (TIA) in Rheumatoid Arthritis
S30	V4-BioP-G	F	15-25	No condition

Table S1. Clinical characteristics of enrolled participants.

Table S2

Predictors	*Estimates*	Inv Simpson *CI*	*p*
**(Intercept)**	161.30	62.28 – 260.32	0.002
**Age**	1.37	-0.72 – 3.46	0.195
**Gender [M]**	6.14	-60.53 – 72.80	0.854
**Matrix [F]**	-40.36	-77.22 – -3.50	0.032
**Random Effects**			
σ^2	5070.71		
$\tau_{00\ Subject}$	3491.60		
**ICC**	0.41		
$N_{Subject}$	30		
**Observations**	60		
**Marginal R2/Conditional R2**	0.086/0.458		

Table S2. Summary of the GLM model used to estimate the Inverse Simpson alpha diversity as a function of age, gender (fixed effect), sampling matrix (fixed effect) and subject (random effect). For each explanatory variable the effects on the response variable were estimated with the Confidence Interval (CI) and the associated p-value. Finally, the estimation of the explained variability is available (R^2).

Table S3

Weighted UniFrac			Unweighted uniFrac		
Factor	R2	p-value	Factor	R2	p-value
Matrix	0.016	0.300	Matrix	0.018	0.066
Gender	0.020	0.150	Gender	0.016	0.236
Age Group	0.033	0.280	Age Group	0.044	0.001 ***
Subject	0.494	0.024 *	Subject	0.492	0.001 ***
Residuals	0.436		Residuals	0.430	

Table S3. Summary of the PERMANOVA model applied on weighted and unweighted UniFrac dissimilarities as function of sampling matrix, gender, age and subject (random effect). For each the estimation of the explained variability (R2) and the associated p-values are shown.

Table S4

**Phylum**	**F**	**F sd**	**CWF**	**CWF sd**
d__Bacteria;p__Bacteroidota	38.27%	12.40%	30.66%	11.20%
d__Bacteria;p__Proteobacteria	8.42%	10.84%	11.05%	17.07%
d__Bacteria;p__Firmicutes	49.20%	14.14%	53.52%	17.11%
d__Bacteria;p__Verrucomicrobiota	0.63%	1.27%	1.08%	2.96%
d__Bacteria;p__Actinobacteriota	3.03%	2.64%	2.69%	2.50%
d__Bacteria;p__Desulfobacterota	0.30%	0.21%	0.42%	0.34%
d__Bacteria;p__Campilobacterota	0.00%	0.00%	0.34%	1.27%
d__Bacteria;p__Fusobacteriota	0.07%	0.37%	0.12%	0.55%
Other	0.06%	0.13%	0.11%	0.17%
Unassigned	0.02%	0.12%	0.00%	0.00%

**Class**	**F**	**F sd**	**CWF**	**CWF sd**
d__Bacteria;p__Bacteroidota;c__Bacteroidia	38.27%	12.40%	30.66%	11.20%
d__Bacteria;p__Proteobacteria;c__Gammaproteobacteria	8.07%	10.97%	10.66%	17.06%
d__Bacteria;p__Firmicutes;c__Clostridia	45.89%	13.69%	49.12%	17.35%
d__Bacteria;p__Verrucomicrobiota;c__Verrucomicrobiae	0.58%	1.28%	1.02%	2.94%
d__Bacteria;p__Actinobacteriota;c__Coriobacteriia	0.91%	0.89%	1.32%	1.77%
d__Bacteria;p__Actinobacteriota;c__Actinobacteria	2.12%	2.03%	1.37%	1.38%
d__Bacteria;p__Firmicutes;c__Negativicutes	1.46%	0.98%	1.68%	0.95%
d__Bacteria;p__Firmicutes;c__Bacilli	1.85%	1.50%	2.72%	2.94%
d__Bacteria;p__Proteobacteria;c__Alphaproteobacteria	0.35%	1.09%	0.39%	1.07%
d__Bacteria;p__Desulfobacterota;c__Desulfovibrionia	0.30%	0.21%	0.42%	0.34%
d__Bacteria;p__Campilobacterota;c__Campylobacteria	0.00%	0.00%	0.34%	1.27%
d__Bacteria;p__Fusobacteriota;c__Fusobacteriia	0.07%	0.37%	0.12%	0.55%
Other	0.11%	0.19%	0.18%	0.23%
Unassigned	0.02%	0.12%	0.00%	0.00%

**Order**	**F**	**F sd**	**CWF**	**CWF sd**

d__Bacteria;p__Bacteroidota;c__Bacteroidia;o__Bacteroidales	38.26%	12.42%	30.65%	11.21%
d__Bacteria;p__Proteobacteria;c__Gammaproteobacteria;o__Enterobacterales	6.06%	11.06%	7.52%	12.79%
d__Bacteria;p__Firmicutes;c__Clostridia;o__Oscillospirales	20.03%	7.95%	22.25%	11.07%
d__Bacteria;p__Firmicutes;c__Clostridia;o__Lachnospirales	21.50%	8.48%	21.30%	9.27%
d__Bacteria;p__Verrucomicrobiota;c__Verrucomicrobiae;o__Verrucomicrobiales	0.56%	1.28%	1.00%	2.94%
d__Bacteria;p__Actinobacteriota;c__Coriobacteriia;o__Coriobacteriales	0.91%	0.89%	1.32%	1.77%
d__Bacteria;p__Actinobacteriota;c__Actinobacteria;o__Bifidobacteriales	2.05%	1.97%	1.14%	1.10%
d__Bacteria;p__Firmicutes;c__Clostridia;o__Peptostreptococcales-Tissierellales	1.27%	3.11%	1.86%	2.82%
d__Bacteria;p__Proteobacteria;c__Gammaproteobacteria;o__Burkholderiales	1.47%	1.62%	2.71%	6.20%
d__Bacteria;p__Firmicutes;c__Clostridia;o__Christensenellales	1.54%	3.26%	1.75%	2.56%
d__Bacteria;p__Firmicutes;c__Negativicutes;o__Veillonellales-Selenomonadales	0.87%	1.09%	0.96%	1.10%
d__Bacteria;p__Firmicutes;c__Bacilli;o__Erysipelotrichales	1.11%	1.02%	1.28%	1.43%
d__Bacteria;p__Firmicutes;c__Clostridia;o__Clostridiales	0.69%	1.89%	0.77%	1.93%
d__Bacteria;p__Proteobacteria;c__Gammaproteobacteria;o__Pseudomonadales	0.21%	0.80%	0.19%	0.85%
d__Bacteria;p__Firmicutes;c__Clostridia;o__Monoglobales	0.31%	0.38%	0.29%	0.34%
d__Bacteria;p__Proteobacteria;c__Alphaproteobacteria;o__Rhodospirillales	0.34%	1.09%	0.39%	1.07%
d__Bacteria;p__Desulfobacterota;c__Desulfovibrionia;o__Desulfovibrionales	0.30%	0.21%	0.42%	0.34%
d__Bacteria;p__Firmicutes;c__Bacilli;o__Lactobacillales	0.55%	1.05%	1.05%	2.47%
d__Bacteria;p__Firmicutes;c__Negativicutes;o__Acidaminococcales	0.59%	0.73%	0.72%	0.62%
d__Bacteria;p__Proteobacteria;c__Gammaproteobacteria;o__Pasteurellales	0.22%	0.52%	0.23%	0.98%
d__Bacteria;p__Firmicutes;c__Bacilli;o__Bacillales	0.08%	0.31%	0.28%	1.53%
d__Bacteria;p__Campilobacterota;c__Campylobacteria;o__Campylobacterales	0.00%	0.00%	0.34%	1.27%
d__Bacteria;p__Firmicutes;c__Clostridia;o__Clostridia_UCG-014	0.41%	0.99%	0.61%	1.28%
d__Bacteria;p__Proteobacteria;c__Gammaproteobacteria;o__Xanthomonadales	0.11%	0.58%	0.00%	0.00%
d__Bacteria;p__Firmicutes;c__Clostridia;o__Clostridia_vadinBB60_group	0.12%	0.28%	0.25%	0.98%
d__Bacteria;p__Actinobacteriota;c__Actinobacteria;o__Actinomycetales	0.02%	0.06%	0.22%	0.91%
d__Bacteria;p__Firmicutes;c__Bacilli;o__RF39	0.06%	0.31%	0.06%	0.11%
d__Bacteria;p__Fusobacteriota;c__Fusobacteriia;o__Fusobacteriales	0.07%	0.37%	0.12%	0.55%
Other	0.29%	0.44%	0.32%	0.39%
Unassigned	0.02%	0.12%	0.00%	0.00%

Family	F	F sd	CWF	CWF sd
d__Bacteria;p__Bacteroidota;c__Bacteroidia;o__Bacteroidales;f__Bacteroidaceae	30.71%	12.60%	21.04%	12.02%
d__Bacteria;p__Proteobacteria;c__Gammaproteobacteria;o__Enterobacterales;f__Enterobacteriaceae	5.96%	11.05%	6.71%	10.68%
d__Bacteria;p__Firmicutes;c__Clostridia;o__Oscillospirales;f__Ruminococcaceae	16.51%	7.09%	17.41%	9.25%
d__Bacteria;p__Bacteroidota;c__Bacteroidia;o__Bacteroidales;f__Prevotellaceae	1.78%	4.96%	4.06%	6.58%
d__Bacteria;p__Firmicutes;c__Clostridia;o__Lachnospirales;f__Lachnospiraceae	21.48%	8.51%	21.29%	9.28%
d__Bacteria;p__Bacteroidota;c__Bacteroidia;o__Bacteroidales;f__Rikenellaceae	3.10%	2.66%	2.06%	1.74%
d__Bacteria;p__Verrucomicrobiota;c__Verrucomicrobiae;o__Verrucomicrobiales;f__Akkermansiaceae	0.56%	1.28%	1.00%	2.94%
d__Bacteria;p__Actinobacteriota;c__Coriobacteriia;o__Coriobacteriales;f__Coriobacteriaceae	0.63%	0.83%	0.89%	1.58%
d__Bacteria;p__Actinobacteriota;c__Actinobacteria;o__Bifidobacteriales;f__Bifidobacteriaceae	2.05%	1.97%	1.14%	1.10%
d__Bacteria;p__Bacteroidota;c__Bacteroidia;o__Bacteroidales;f__Tannerellaceae	1.60%	1.24%	1.93%	1.36%
d__Bacteria;p__Firmicutes;c__Clostridia;o__Peptostreptococcales-Tissierellales;f__Peptostreptococcaceae	1.16%	3.12%	0.93%	1.63%
d__Bacteria;p__Proteobacteria;c__Gammaproteobacteria;o__Burkholderiales;f__Comamonadaceae	0.00%	0.00%	1.71%	6.39%
d__Bacteria;p__Firmicutes;c__Clostridia;o__Oscillospirales;f__Oscillospiraceae	2.77%	1.84%	3.87%	3.34%
d__Bacteria;p__Firmicutes;c__Clostridia;o__Christensenellales;f__Christensenellaceae	1.54%	3.26%	1.75%	2.56%
d__Bacteria;p__Firmicutes;c__Negativicutes;o__Veillonellales-Selenomonadales;f__Veillonellaceae	0.86%	1.08%	0.96%	1.09%
d__Bacteria;p__Firmicutes;c__Bacilli;o__Erysipelotrichales;f__Erysipelatoclostridiaceae	0.52%	0.48%	0.70%	1.05%
d__Bacteria;p__Firmicutes;c__Clostridia;o__Clostridiales;f__Clostridiaceae	0.69%	1.89%	0.77%	1.93%
d__Bacteria;p__Proteobacteria;c__Gammaproteobacteria;o__Enterobacterales;f__Erwiniaceae	0.02%	0.06%	0.63%	2.90%
d__Bacteria;p__Proteobacteria;c__Gammaproteobacteria;o__Burkholderiales;f__Sutterellaceae	1.46%	1.62%	0.98%	0.81%
d__Bacteria;p__Bacteroidota;c__Bacteroidia;o__Bacteroidales;f__Marinifilaceae	0.38%	0.28%	0.38%	0.31%
d__Bacteria;p__Proteobacteria;c__Gammaproteobacteria;o__Pseudomonadales;f__Pseudomonadaceae	0.21%	0.80%	0.08%	0.37%
d__Bacteria;p__Firmicutes;c__Clostridia;o__Monoglobales;f__Monoglobaceae	0.31%	0.38%	0.29%	0.34%
d__Bacteria;p__Proteobacteria;c__Alphaproteobacteria;o__Rhodospirillales;f__uncultured	0.34%	1.09%	0.39%	1.07%
d__Bacteria;p__Desulfobacterota;c__Desulfovibrionia;o__Desulfovibrionales;f__Desulfovibrionaceae	0.30%	0.21%	0.42%	0.34%
d__Bacteria;p__Firmicutes;c__Bacilli;o__Lactobacillales;f__Streptococcaceae	0.42%	0.97%	0.32%	0.58%
d__Bacteria;p__Firmicutes;c__Negativicutes;o__Acidaminococcales;f__Acidaminococcaceae	0.59%	0.73%	0.72%	0.62%
d__Bacteria;p__Firmicutes;c__Bacilli;o__Erysipelotrichales;f__Erysipelotrichaceae	0.60%	0.94%	0.58%	0.59%
d__Bacteria;p__Proteobacteria;c__Gammaproteobacteria;o__Pseudomonadales;f__Moraxellaceae	0.00%	0.00%	0.12%	0.49%
d__Bacteria;p__Bacteroidota;c__Bacteroidia;o__Bacteroidales;f__Barnesiellaceae	0.63%	0.94%	0.69%	0.88%
d__Bacteria;p__Firmicutes;c__Clostridia;o__Oscillospirales;f__Butyricicoccaceae	0.27%	0.24%	0.39%	0.32%

d__Bacteria;p__Firmicutes;c__Bacilli;o__Lactobacillales;f__Lactobacillaceae	0.03%	0.08%	0.44%	2.39%
d__Bacteria;p__Proteobacteria;c__Gammaproteobacteria;o__Pasteurellales;f__Pasteurellaceae	0.22%	0.52%	0.23%	0.98%
d__Bacteria;p__Firmicutes;c__Clostridia;o__Oscillospirales;f__[Eubacterium]_coprostanoligenes_group	0.36%	0.35%	0.42%	0.43%
d__Bacteria;p__Firmicutes;c__Bacilli;o__Lactobacillales;f__Enterococcaceae	0.08%	0.22%	0.28%	0.61%
d__Bacteria;p__Firmicutes;c__Bacilli;o__Bacillales;f__Planococcaceae	0.02%	0.12%	0.28%	1.53%
d__Bacteria;p__Firmicutes;c__Clostridia;o__Peptostreptococcales-Tissierellales;f__Peptostreptococcales-Tissierellales	0.02%	0.07%	0.77%	2.45%
d__Bacteria;p__Campilobacterota;c__Campylobacteria;o__Campylobacterales;f__Campylobacteraceae	0.00%	0.00%	0.34%	1.27%
d__Bacteria;p__Firmicutes;c__Clostridia;o__Clostridia_UCG-014;f__Clostridia_UCG-014	0.41%	0.99%	0.61%	1.28%
d__Bacteria;p__Proteobacteria;c__Gammaproteobacteria;o__Xanthomonadales;f__Xanthomonadaceae	0.11%	0.58%	0.00%	0.00%
d__Bacteria;p__Firmicutes;c__Clostridia;o__Clostridia_vadinBB60_group;f__Clostridia_vadinBB60_group	0.12%	0.28%	0.25%	0.98%
d__Bacteria;p__Bacteroidota;c__Bacteroidia;o__Bacteroidales;f__Muribaculaceae	0.05%	0.15%	0.36%	0.68%
d__Bacteria;p__Actinobacteriota;c__Actinobacteria;o__Actinomycetales;f__Actinomycetaceae	0.02%	0.06%	0.22%	0.91%
d__Bacteria;p__Proteobacteria;c__Gammaproteobacteria;o__Enterobacterales;f__Yersiniaceae	0.06%	0.31%	0.06%	0.28%
d__Bacteria;p__Firmicutes;c__Bacilli;o__RF39;f__RF39	0.06%	0.31%	0.06%	0.11%
d__Bacteria;p__Fusobacteriota;c__Fusobacteriia;o__Fusobacteriales;f__Fusobacteriaceae	0.07%	0.37%	0.12%	0.55%
d__Bacteria;p__Proteobacteria;c__Gammaproteobacteria;o__Enterobacterales;f__Morganellaceae	0.00%	0.00%	0.09%	0.35%
d__Bacteria;p__Actinobacteriota;c__Coriobacteriia;o__Coriobacteriales;f__Atopobiaceae	0.08%	0.27%	0.12%	0.47%
d__Bacteria;p__Bacteroidota;c__Bacteroidia;o__Bacteroidales;f__Porphyromonadaceae	0.00%	0.00%	0.13%	0.39%
Other	0.84%	0.69%	1.02%	0.80%
Unassigned	0.03%	0.14%	0.01%	0.04%

**Genus**	**F**	**F sd**	**CWF**	**CWF sd**
d__Bacteria;p__Bacteroidota;c__Bacteroidia;o__Bacteroidales;f__Bacteroidaceae;g__Bacteroides	30.71%	12.60%	21.04%	12.02%
d__Bacteria;p__Proteobacteria;c__Gammaproteobacteria;o__Enterobacterales;f__Enterobacteriaceae;g__Escherichia-Shigella	4.65%	8.36%	4.06%	5.73%
d__Bacteria;p__Firmicutes;c__Clostridia;o__Oscillospirales;f__Ruminococcaceae;g__Faecalibacterium	8.29%	5.06%	9.45%	7.16%
d__Bacteria;p__Bacteroidota;c__Bacteroidia;o__Bacteroidales;f__Prevotellaceae;g__Prevotella	0.57%	3.12%	2.72%	5.22%
d__Bacteria;p__Firmicutes;c__Clostridia;o__Lachnospirales;f__Lachnospiraceae;g__Blautia	3.92%	3.15%	4.36%	4.00%
d__Bacteria;p__Firmicutes;c__Clostridia;o__Oscillospirales;f__Ruminococcaceae;g__Ruminococcus	3.45%	2.58%	2.88%	2.61%
d__Bacteria;p__Firmicutes;c__Clostridia;o__Lachnospirales;f__Lachnospiraceae;g__Agathobacter	2.74%	2.57%	2.08%	2.45%
d__Bacteria;p__Bacteroidota;c__Bacteroidia;o__Bacteroidales;f__Rikenellaceae;g__Alistipes	3.09%	2.67%	2.02%	1.76%
d__Bacteria;p__Firmicutes;c__Clostridia;o__Oscillospirales;f__Ruminococcaceae;g__Subdoligranulum	2.17%	1.83%	2.32%	1.80%

d__Bacteria;p__Verrucomicrobiota;c__Verrucomicrobiae;o__Verrucomicrobiales;f__Akkermansiaceae;g__Akkermansia	0.56%	1.28%	1.00%	2.94%
d__Bacteria;p__Firmicutes;c__Clostridia;o__Oscillospirales;f__Ruminococcaceae;g__CAG-352	1.06%	2.56%	0.59%	1.54%
d__Bacteria;p__Firmicutes;c__Clostridia;o__Lachnospirales;f__Lachnospiraceae;g__Roseburia	2.67%	3.46%	1.67%	1.76%
d__Bacteria;p__Firmicutes;c__Clostridia;o__Lachnospirales;f__Lachnospiraceae;g__Fusicatenibacter	0.70%	0.54%	0.80%	0.68%
d__Bacteria;p__Proteobacteria;c__Gammaproteobacteria;o__Enterobacterales;f__Enterobacteriaceae;g__Enterobacter	0.03%	0.11%	0.90%	4.46%
d__Bacteria;p__Proteobacteria;c__Gammaproteobacteria;o__Enterobacterales;f__Enterobacteriaceae;g__Citrobacter	0.92%	4.87%	0.98%	3.40%
d__Bacteria;p__Actinobacteriota;c__Coriobacteriia;o__Coriobacteriales;f__Coriobacteriaceae;g__Collinsella	0.63%	0.83%	0.88%	1.59%
d__Bacteria;p__Proteobacteria;c__Gammaproteobacteria;o__Enterobacterales;f__Enterobacteriaceae;g__Salmonella	0.28%	0.54%	0.26%	0.41%
d__Bacteria;p__Firmicutes;c__Clostridia;o__Lachnospirales;f__Lachnospiraceae;g__Lachnospira	0.98%	1.20%	1.05%	2.01%
d__Bacteria;p__Firmicutes;c__Clostridia;o__Lachnospirales;f__Lachnospiraceae;g__Lachnospiraceae_NK4A136_group	0.99%	1.34%	0.94%	0.95%
d__Bacteria;p__Actinobacteriota;c__Actinobacteria;o__Bifidobacteriales;f__Bifidobacteriaceae;g__Bifidobacterium	2.05%	1.97%	1.05%	0.98%
d__Bacteria;p__Bacteroidota;c__Bacteroidia;o__Bacteroidales;f__Tannerellaceae;g__Parabacteroides	1.60%	1.24%	1.93%	1.36%
d__Bacteria;p__Firmicutes;c__Clostridia;o__Peptostreptococcales-Tissierellales;f__Peptostreptococcaceae;g__Romboutsia	0.47%	0.80%	0.69%	1.52%
d__Bacteria;p__Firmicutes;c__Clostridia;o__Lachnospirales;f__Lachnospiraceae;g__Anaerostipes	0.74%	0.91%	0.67%	1.38%
d__Bacteria;p__Proteobacteria;c__Gammaproteobacteria;o__Burkholderiales;f__Comamonadaceae;g__Comamonas	0.00%	0.00%	1.70%	6.39%
d__Bacteria;p__Firmicutes;c__Clostridia;o__Oscillospirales;f__Oscillospiraceae;g__UCG-002	0.90%	1.05%	1.83%	2.13%
d__Bacteria;p__Firmicutes;c__Clostridia;o__Lachnospirales;f__Lachnospiraceae;g__Dorea	0.58%	0.51%	0.84%	0.52%
d__Bacteria;p__Bacteroidota;c__Bacteroidia;o__Bacteroidales;f__Prevotellaceae;g__Prevotellaceae_NK3B31_group	0.42%	1.10%	0.55%	1.20%
d__Bacteria;p__Firmicutes;c__Clostridia;o__Lachnospirales;f__Lachnospiraceae;g__[Eubacterium]_hallii_group	0.86%	0.82%	0.69%	0.65%
d__Bacteria;p__Firmicutes;c__Clostridia;o__Lachnospirales;f__Lachnospiraceae;g__[Ruminococcus]_torques_group	1.86%	1.72%	1.65%	1.28%
d__Bacteria;p__Firmicutes;c__Clostridia;o__Lachnospirales;f__Lachnospiraceae;g__Coprococcus	0.69%	0.83%	1.21%	1.29%
d__Bacteria;p__Firmicutes;c__Clostridia;o__Christensenellales;f__Christensenellaceae;g__Christensenellaceae_R-7_group	1.53%	3.27%	1.74%	2.56%
d__Bacteria;p__Firmicutes;c__Clostridia;o__Lachnospirales;f__Lachnospiraceae;g__[Ruminococcus]_gnavus_group	0.15%	0.26%	0.52%	2.17%
d__Bacteria;p__Firmicutes;c__Clostridia;o__Oscillospirales;f__Ruminococcaceae;g__[Eubacterium]_siraeum_group	0.52%	1.53%	0.69%	1.82%
d__Bacteria;p__Firmicutes;c__Negativicutes;o__Veillonellales-Selenomonadales;f__Veillonellaceae;g__Dialister	0.81%	1.08%	0.77%	1.02%
d__Bacteria;p__Firmicutes;c__Clostridia;o__Lachnospirales;f__Lachnospiraceae;g__Lachnospiraceae_UCG-008	0.47%	0.46%	0.40%	0.33%
d__Bacteria;p__Firmicutes;c__Clostridia;o__Lachnospirales;f__Lachnospiraceae;g__[Eubacterium]_eligens_group	0.89%	1.15%	0.68%	0.89%
d__Bacteria;p__Firmicutes;c__Bacilli;o__Erysipelotrichales;f__Erysipelatoclostridiaceae;g__Erysipelotrichaceae_UCG-003	0.36%	0.49%	0.30%	0.40%
d__Bacteria;p__Firmicutes;c__Clostridia;o__Clostridiales;f__Clostridiaceae;g__Clostridium_sensu_stricto_1	0.69%	1.89%	0.77%	1.93%
d__Bacteria;p__Firmicutes;c__Clostridia;o__Peptostreptococcales-Tissierellales;f__Peptostreptococcaceae;g__Intestinibacter	0.51%	1.82%	0.16%	0.33%
d__Bacteria;p__Bacteroidota;c__Bacteroidia;o__Bacteroidales;f__Prevotellaceae;g__Alloprevotella	0.28%	1.07%	0.43%	1.68%

d__Bacteria;p__Proteobacteria;c__Gammaproteobacteria;o__Enterobacterales;f__Erwiniaceae;g__Pantoea	0.02%	0.05%	0.63%	2.90%
d__Bacteria;p__Proteobacteria;c__Gammaproteobacteria;o__Burkholderiales;f__Sutterellaceae;g__Parasutterella	0.56%	0.72%	0.31%	0.62%
d__Bacteria;p__Firmicutes;c__Clostridia;o__Lachnospirales;f__Lachnospiraceae;g__Lachnoclostridium	0.74%	0.59%	0.76%	0.70%
d__Bacteria;p__Proteobacteria;c__Gammaproteobacteria;o__Pseudomonadales;f__Pseudomonadaceae;g__Pseudomonas	0.21%	0.80%	0.08%	0.37%
d__Bacteria;p__Firmicutes;c__Clostridia;o__Lachnospirales;f__Lachnospiraceae;g__Lachnospiraceae_ND3007_group	0.17%	0.19%	0.27%	0.30%
d__Bacteria;p__Firmicutes;c__Clostridia;o__Monoglobales;f__Monoglobaceae;g__Monoglobus	0.31%	0.38%	0.29%	0.34%
d__Bacteria;p__Firmicutes;c__Clostridia;o__Oscillospirales;f__Ruminococcaceae;g__Incertae_Sedis	0.33%	0.23%	0.68%	1.23%
d__Bacteria;p__Proteobacteria;c__Alphaproteobacteria;o__Rhodospirillales;f__uncultured;g__uncultured	0.34%	1.09%	0.39%	1.07%
d__Bacteria;p__Firmicutes;c__Clostridia;o__Lachnospirales;f__Lachnospiraceae;g__CAG-56	0.12%	0.20%	0.31%	0.66%
d__Bacteria;p__Firmicutes;c__Bacilli;o__Lactobacillales;f__Streptococcaceae;g__Streptococcus	0.42%	0.97%	0.32%	0.58%
d__Bacteria;p__Firmicutes;c__Clostridia;o__Lachnospirales;f__Lachnospiraceae;g__[Eubacterium]_ruminantium_group	0.26%	0.68%	0.33%	0.97%
d__Bacteria;p__Proteobacteria;c__Gammaproteobacteria;o__Enterobacterales;f__Enterobacteriaceae;g__Klebsiella	0.05%	0.10%	0.28%	0.73%
d__Bacteria;p__Firmicutes;c__Clostridia;o__Oscillospirales;f__Oscillospiraceae;g__UCG-005	0.38%	0.50%	0.62%	0.66%
d__Bacteria;p__Firmicutes;c__Negativicutes;o__Acidaminococcales;f__Acidaminococcaceae;g__Phascolarctobacterium	0.47%	0.64%	0.63%	0.55%
d__Bacteria;p__Firmicutes;c__Bacilli;o__Erysipelotrichales;f__Erysipelotrichaceae;g__Turicibacter	0.40%	0.92%	0.22%	0.40%
d__Bacteria;p__Firmicutes;c__Clostridia;o__Oscillospirales;f__Oscillospiraceae;g__Colidextribacter	0.30%	0.26%	0.35%	0.47%
d__Bacteria;p__Proteobacteria;c__Gammaproteobacteria;o__Pseudomonadales;f__Moraxellaceae;g__Acinetobacter	0.00%	0.00%	0.12%	0.49%
d__Bacteria;p__Firmicutes;c__Clostridia;o__Oscillospirales;f__Oscillospiraceae;g__Oscillibacter	0.28%	0.24%	0.16%	0.13%
d__Bacteria;p__Firmicutes;c__Clostridia;o__Oscillospirales;f__Oscillospiraceae;g__UCG-003	0.17%	0.33%	0.22%	0.28%
d__Bacteria;p__Bacteroidota;c__Bacteroidia;o__Bacteroidales;f__Prevotellaceae;g__Paraprevotella	0.48%	0.97%	0.23%	0.39%
d__Bacteria;p__Firmicutes;c__Clostridia;o__Lachnospirales;f__Lachnospiraceae;g__[Ruminococcus]_gauvreauii_group	0.15%	0.22%	0.21%	0.32%
d__Bacteria;p__Proteobacteria;c__Gammaproteobacteria;o__Burkholderiales;f__Sutterellaceae;g__Sutterella	0.90%	1.73%	0.68%	0.75%
d__Bacteria;p__Bacteroidota;c__Bacteroidia;o__Bacteroidales;f__Barnesiellaceae;g__Barnesiella	0.60%	0.94%	0.62%	0.87%
d__Bacteria;p__Firmicutes;c__Clostridia;o__Oscillospirales;f__Butyricicoccaceae;g__Butyricicoccus	0.26%	0.24%	0.35%	0.29%
d__Bacteria;p__Firmicutes;c__Bacilli;o__Lactobacillales;f__Lactobacillaceae;g__Lactobacillus	0.03%	0.08%	0.44%	2.39%
d__Bacteria;p__Firmicutes;c__Clostridia;o__Oscillospirales;f__Oscillospiraceae;g__NK4A214_group	0.20%	0.40%	0.30%	0.63%
d__Bacteria;p__Proteobacteria;c__Gammaproteobacteria;o__Pasteurellales;f__Pasteurellaceae;g__Haemophilus	0.22%	0.52%	0.23%	0.96%
d__Bacteria;p__Firmicutes;c__Clostridia;o__Oscillospirales;f__Oscillospiraceae;g__Flavonifractor	0.19%	0.37%	0.07%	0.09%
d__Bacteria;p__Firmicutes;c__Clostridia;o__Oscillospirales;f__Ruminococcaceae;g__UBA1819	0.09%	0.10%	0.13%	0.26%
d__Bacteria;p__Firmicutes;c__Clostridia;o__Peptostreptococcales-Tissierellales;f__Peptostreptococcaceae;g__Terrisporobacter	0.16%	0.56%	0.06%	0.14%
d__Bacteria;p__Firmicutes;c__Clostridia;o__Oscillospirales;f__[Eubacterium]_coprostanoligenes_group;g__[Eubacterium]_coprostanoligenes_group	0.36%	0.35%	0.42%	0.43%

d__Bacteria;p__Firmicutes;c__Bacilli;o__Lactobacillales;f__Enterococcaceae;g__Enterococcus	0.08%	0.22%	0.28%	0.61%
d__Bacteria;p__Firmicutes;c__Bacilli;o__Bacillales;f__Planococcaceae;g__Lysinibacillus	0.02%	0.11%	0.28%	1.53%
d__Bacteria;p__Firmicutes;c__Clostridia;o__Lachnospirales;f__Lachnospiraceae;g__uncultured	0.45%	0.25%	0.45%	0.28%
d__Bacteria;p__Firmicutes;c__Clostridia;o__Peptostreptococcales-Tissierellales;f__Peptostreptococcales-Tissierellales;g__Ezakiella	0.00%	0.00%	0.15%	0.45%
d__Bacteria;p__Campilobacterota;c__Campylobacteria;o__Campylobacterales;f__Campylobacteraceae;g__Campylobacter	0.00%	0.00%	0.34%	1.27%
d__Bacteria;p__Firmicutes;c__Negativicutes;o__Acidaminococcales;f__Acidaminococcaceae;g__Acidaminococcus	0.12%	0.31%	0.09%	0.21%
d__Bacteria;p__Firmicutes;c__Clostridia;o__Oscillospirales;f__Ruminococcaceae;g__uncultured	0.51%	1.00%	0.53%	0.88%
d__Bacteria;p__Firmicutes;c__Clostridia;o__Clostridia_UCG-014;f__Clostridia_UCG-014;g__Clostridia_UCG-014	0.41%	0.99%	0.61%	1.28%
d__Bacteria;p__Proteobacteria;c__Gammaproteobacteria;o__Enterobacterales;f__Enterobacteriaceae;g__Raoultella	0.02%	0.11%	0.22%	0.81%
d__Bacteria;p__Firmicutes;c__Bacilli;o__Erysipelotrichales;f__Erysipelatoclostridiaceae;g__Catenibacterium	0.04%	0.16%	0.20%	0.54%
d__Bacteria;p__Firmicutes;c__Bacilli;o__Erysipelotrichales;f__Erysipelotrichaceae;g__Holdemanella	0.05%	0.17%	0.20%	0.41%
d__Bacteria;p__Proteobacteria;c__Gammaproteobacteria;o__Xanthomonadales;f__Xanthomonadaceae;g__Stenotrophomonas	0.11%	0.58%	0.00%	0.00%
d__Bacteria;p__Firmicutes;c__Clostridia;o__Lachnospirales;f__Lachnospiraceae;g__Lachnospiraceae_NC2004_group	0.04%	0.11%	0.10%	0.32%
d__Bacteria;p__Firmicutes;c__Bacilli;o__Erysipelotrichales;f__Erysipelatoclostridiaceae;g__Coprobacillus	0.01%	0.02%	0.13%	0.67%
d__Bacteria;p__Firmicutes;c__Clostridia;o__Clostridia_vadinBB60_group;f__Clostridia_vadinBB60_group;g__Clostridia_vadinBB60_group	0.12%	0.28%	0.25%	0.98%
d__Bacteria;p__Firmicutes;c__Bacilli;o__Erysipelotrichales;f__Erysipelatoclostridiaceae;g__Erysipelatoclostridium	0.10%	0.23%	0.05%	0.12%
d__Bacteria;p__Bacteroidota;c__Bacteroidia;o__Bacteroidales;f__Muribaculaceae;g__Muribaculaceae	0.05%	0.15%	0.36%	0.68%
d__Bacteria;p__Actinobacteriota;c__Actinobacteria;o__Actinomycetales;f__Actinomycetaceae;g__Mobiluncus	0.00%	0.00%	0.16%	0.78%
d__Bacteria;p__Firmicutes;c__Clostridia;o__Lachnospirales;f__Lachnospiraceae;g__Hungatella	0.13%	0.62%	0.04%	0.15%
d__Bacteria;p__Firmicutes;c__Clostridia;o__Peptostreptococcales-Tissierellales;f__Peptostreptococcales-Tissierellales;g__Fenollaria	0.00%	0.00%	0.16%	0.87%
d__Bacteria;p__Firmicutes;c__Clostridia;o__Peptostreptococcales-Tissierellales;f__Peptostreptococcales-Tissierellales;g__Peptoniphilus	0.00%	0.02%	0.20%	0.67%
d__Bacteria;p__Firmicutes;c__Clostridia;o__Peptostreptococcales-Tissierellales;f__Peptostreptococcales-Tissierellales;g__Finegoldia	0.01%	0.04%	0.08%	0.30%
d__Bacteria;p__Firmicutes;c__Clostridia;o__Peptostreptococcales-Tissierellales;f__Peptostreptococcales-Tissierellales;g__Murdochiella	0.00%	0.00%	0.07%	0.24%
d__Bacteria;p__Firmicutes;c__Negativicutes;o__Veillonellales-Selenomonadales;f__Veillonellaceae;g__Veillonella	0.04%	0.08%	0.15%	0.58%
d__Bacteria;p__Firmicutes;c__Bacilli;o__RF39;f__RF39;g__RF39	0.06%	0.31%	0.06%	0.11%
d__Bacteria;p__Firmicutes;c__Clostridia;o__Lachnospirales;f__Lachnospiraceae;g__Anaerosporobacter	0.06%	0.30%	0.06%	0.21%
d__Bacteria;p__Fusobacteriota;c__Fusobacteriia;o__Fusobacteriales;f__Fusobacteriaceae;g__Fusobacterium	0.07%	0.37%	0.12%	0.55%
d__Bacteria;p__Proteobacteria;c__Gammaproteobacteria;o__Enterobacterales;f__Morganellaceae;g__Providencia	0.00%	0.00%	0.09%	0.35%
d__Bacteria;p__Firmicutes;c__Bacilli;o__Erysipelotrichales;f__Erysipelotrichaceae;g__[Clostridium]_innocuum_group	0.06%	0.26%	0.01%	0.03%
d__Bacteria;p__Actinobacteriota;c__Coriobacteriia;o__Coriobacteriales;f__Atopobiaceae;g__Olsenella	0.08%	0.27%	0.01%	0.03%
d__Bacteria;p__Firmicutes;c__Clostridia;o__Peptostreptococcales-Tissierellales;f__Peptostreptococcales-Tissierellales;g__Anaerococcus	0.00%	0.00%	0.09%	0.32%

d__Bacteria;p__Desulfobacterota;c__Desulfovibrionia;o__Desulfovibrionales;f__Desulfovibrionaceae;g__Desulfovibrio	0.02%	0.09%	0.18%	0.35%
d__Bacteria;p__Actinobacteriota;c__Actinobacteria;o__Bifidobacteriales;f__Bifidobacteriaceae;g__Gardnerella	0.00%	0.00%	0.10%	0.52%
d__Bacteria;p__Bacteroidota;c__Bacteroidia;o__Bacteroidales;f__Porphyromonadaceae;g__Porphyromonas	0.00%	0.00%	0.13%	0.39%
d__Bacteria;p__Actinobacteriota;c__Coriobacteriia;o__Coriobacteriales;f__Atopobiaceae;g__Atopobium	0.00%	0.00%	0.08%	0.45%
d__Bacteria;p__Bacteroidota;c__Bacteroidia;o__Bacteroidales;f__Prevotellaceae;g__Prevotellaceae_Ga6A1_group	0.00%	0.00%	0.07%	0.40%
Other	3.28%	1.24%	3.89%	1.82%
Unassigned	0.08%	0.19%	0.07%	0.18%

**Species**	**F**	**F sd**	**CWF**	**CWF sd**
d__Bacteria;p__Bacteroidota;c__Bacteroidia;o__Bacteroidales;f__Bacteroidaceae;g__Bacteroides;s__Bacteroides_vulgatus	5.28%	4.77%	3.09%	4.24%
d__Bacteria;p__Proteobacteria;c__Gammaproteobacteria;o__Enterobacterales;f__Enterobacteriaceae;g__Escherichia-Shigella;s__Escherichia_coli	1.43%	2.55%	1.25%	1.78%
d__Bacteria;p__Proteobacteria;c__Gammaproteobacteria;o__Enterobacterales;f__Enterobacteriaceae;g__Escherichia-Shigella;s__Shigella_boydii	1.54%	2.73%	1.33%	1.85%
d__Bacteria;p__Firmicutes;c__Clostridia;o__Oscillospirales;f__Ruminococcaceae;g__Faecalibacterium;s__uncultured_organism	1.88%	1.47%	1.82%	1.40%
d__Bacteria;p__Bacteroidota;c__Bacteroidia;o__Bacteroidales;f__Bacteroidaceae;g__Bacteroides;s__bacterium_NLAE-zl-P803	4.17%	2.70%	3.42%	2.97%
d__Bacteria;p__Bacteroidota;c__Bacteroidia;o__Bacteroidales;f__Bacteroidaceae;g__Bacteroides;s__Bacteroides_dorei	1.54%	2.41%	2.35%	3.79%
d__Bacteria;p__Proteobacteria;c__Gammaproteobacteria;o__Enterobacterales;f__Enterobacteriaceae;g__Escherichia-Shigella;s__uncultured_organism	0.49%	0.90%	0.43%	0.60%
d__Bacteria;p__Firmicutes;c__Clostridia;o__Oscillospirales;f__Ruminococcaceae;g__Faecalibacterium;s__uncultured_bacterium	3.04%	2.54%	3.75%	3.67%
d__Bacteria;p__Proteobacteria;c__Gammaproteobacteria;o__Enterobacterales;f__Enterobacteriaceae;g__Escherichia-Shigella;s__uncultured_bacterium	0.68%	1.21%	0.58%	0.80%
d__Bacteria;p__Bacteroidota;c__Bacteroidia;o__Bacteroidales;f__Bacteroidaceae;g__Bacteroides;s__Bacteroides_stercoris	2.21%	4.72%	0.77%	1.73%
d__Bacteria;p__Bacteroidota;c__Bacteroidia;o__Bacteroidales;f__Prevotellaceae;g__Prevotella;s__uncultured_bacterium	0.43%	2.35%	1.78%	3.91%
d__Bacteria;p__Firmicutes;c__Clostridia;o__Oscillospirales;f__Ruminococcaceae;g__Faecalibacterium;s__uncultured_Faecalibacterium	0.68%	0.51%	0.65%	0.47%
d__Bacteria;p__Firmicutes;c__Clostridia;o__Lachnospirales;f__Lachnospiraceae;g__Blautia;s__uncultured_bacterium	2.82%	2.87%	2.81%	2.73%
d__Bacteria;p__Bacteroidota;c__Bacteroidia;o__Bacteroidales;f__Bacteroidaceae;g__Bacteroides;s__Bacteroides_plebeius	1.17%	2.15%	0.68%	1.63%
d__Bacteria;p__Bacteroidota;c__Bacteroidia;o__Bacteroidales;f__Bacteroidaceae;g__Bacteroides;s__uncultured_bacterium	3.61%	4.09%	1.83%	1.99%
d__Bacteria;p__Firmicutes;c__Clostridia;o__Oscillospirales;f__Ruminococcaceae;g__Ruminococcus;s__uncultured_bacterium	2.08%	1.77%	1.69%	1.84%
d__Bacteria;p__Firmicutes;c__Clostridia;o__Lachnospirales;f__Lachnospiraceae;g__Agathobacter;s__uncultured_bacterium	0.99%	0.88%	0.83%	0.92%
d__Bacteria;p__Proteobacteria;c__Gammaproteobacteria;o__Enterobacterales;f__Enterobacteriaceae;g__Escherichia-Shigella;s__uncultured_Shigella	0.21%	0.37%	0.19%	0.26%
d__Bacteria;p__Bacteroidota;c__Bacteroidia;o__Bacteroidales;f__Rikenellaceae;g__Alistipes;s__uncultured_organism	1.24%	0.98%	0.99%	0.81%
d__Bacteria;p__Bacteroidota;c__Bacteroidia;o__Bacteroidales;f__Bacteroidaceae;g__Bacteroides;s__Bacteroides_uniformis	1.00%	0.72%	0.71%	1.21%
d__Bacteria;p__Bacteroidota;c__Bacteroidia;o__Bacteroidales;f__Bacteroidaceae;g__Bacteroides;s__Bacteroides_coprocola	0.92%	2.59%	0.58%	1.89%
d__Bacteria;p__Bacteroidota;c__Bacteroidia;o__Bacteroidales;f__Bacteroidaceae;g__Bacteroides;s__Bacteroides_caccae	0.72%	1.40%	0.39%	0.55%

d__Bacteria;p__Bacteroidota;c__Bacteroidia;o__Bacteroidales;f__Bacteroidaceae;g__Bacteroides;s__uncultured_organism	4.69%	4.12%	2.65%	2.83%
d__Bacteria;p__Verrucomicrobiota;c__Verrucomicrobiae;o__Verrucomicrobiales;f__Akkermansiaceae;g__Akkermansia;s__Akkermansia_muciniphila	0.28%	0.64%	0.49%	1.50%
d__Bacteria;p__Firmicutes;c__Clostridia;o__Oscillospirales;f__Ruminococcaceae;g__Subdoligranulum;s__uncultured_bacterium	1.63%	1.36%	1.73%	1.25%
d__Bacteria;p__Bacteroidota;c__Bacteroidia;o__Bacteroidales;f__Prevotellaceae;g__Prevotella;s__metagenome	0.14%	0.77%	0.49%	1.34%
d__Bacteria;p__Firmicutes;c__Clostridia;o__Oscillospirales;f__Ruminococcaceae;g__CAG-352;s__uncultured_bacterium	0.38%	1.43%	0.51%	1.54%
d__Bacteria;p__Proteobacteria;c__Gammaproteobacteria;o__Enterobacterales;f__Enterobacteriaceae;g__Enterobacter;s__Klebsiella_oxytoca	0.02%	0.10%	0.82%	4.47%
d__Bacteria;p__Proteobacteria;c__Gammaproteobacteria;o__Enterobacterales;f__Enterobacteriaceae;g__Citrobacter;s__Citrobacter_freundii	0.55%	2.85%	0.62%	2.10%
d__Bacteria;p__Firmicutes;c__Clostridia;o__Oscillospirales;f__Ruminococcaceae;g__Faecalibacterium;s__metagenome	0.24%	0.19%	0.58%	1.13%
d__Bacteria;p__Firmicutes;c__Clostridia;o__Oscillospirales;f__Ruminococcaceae;g__Faecalibacterium;s__Faecalibacterium_prausnitzii	0.77%	0.94%	0.91%	1.85%
d__Bacteria;p__Verrucomicrobiota;c__Verrucomicrobiae;o__Verrucomicrobiales;f__Akkermansiaceae;g__Akkermansia;s__uncultured_bacterium	0.28%	0.64%	0.49%	1.44%
d__Bacteria;p__Firmicutes;c__Clostridia;o__Lachnospirales;f__Lachnospiraceae;g__Blautia;s__uncultured_Blautia	0.35%	0.22%	0.40%	0.39%
d__Bacteria;p__Proteobacteria;c__Gammaproteobacteria;o__Enterobacterales;f__Enterobacteriaceae;g__Salmonella;s__Salmonella_enterica	0.28%	0.54%	0.26%	0.41%
d__Bacteria;p__Firmicutes;c__Clostridia;o__Lachnospirales;f__Lachnospiraceae;g__Lachnospira;s__uncultured_bacterium	0.84%	1.12%	0.99%	2.02%
d__Bacteria;p__Firmicutes;c__Clostridia;o__Lachnospirales;f__Lachnospiraceae;g__Roseburia;s__uncultured_organism	1.68%	3.15%	0.97%	1.42%
d__Bacteria;p__Bacteroidota;c__Bacteroidia;o__Bacteroidales;f__Bacteroidaceae;g__Bacteroides;s__Bacteroides_massiliensis	0.60%	1.14%	0.33%	0.57%
d__Bacteria;p__Firmicutes;c__Clostridia;o__Lachnospirales;f__Lachnospiraceae;g__Lachnospiraceae_NK4A136_group;s__uncultured_bacterium	0.43%	0.82%	0.36%	0.50%
d__Bacteria;p__Bacteroidota;c__Bacteroidia;o__Bacteroidales;f__Bacteroidaceae;g__Bacteroides;s__Bacteroides_fragilis	0.21%	0.52%	0.81%	2.22%
d__Bacteria;p__Actinobacteriota;c__Actinobacteria;o__Bifidobacteriales;f__Bifidobacteriaceae;g__Bifidobacterium;s__Bifidobacterium_breve	0.66%	0.88%	0.35%	0.47%
d__Bacteria;p__Bacteroidota;c__Bacteroidia;o__Bacteroidales;f__Bacteroidaceae;g__Bacteroides;s__Bacteroides_xylanisolvens	0.51%	0.90%	0.22%	0.35%
d__Bacteria;p__Firmicutes;c__Clostridia;o__Lachnospirales;f__Lachnospiraceae;g__Blautia;s__uncultured_organism	0.63%	0.48%	0.84%	0.82%
d__Bacteria;p__Bacteroidota;c__Bacteroidia;o__Bacteroidales;f__Bacteroidaceae;g__Bacteroides;s__Bacteroides_thetaiotaomicron	0.49%	0.64%	0.36%	0.41%
d__Bacteria;p__Bacteroidota;c__Bacteroidia;o__Bacteroidales;f__Bacteroidaceae;g__Bacteroides;s__Bacteroides_caecimuris	0.20%	0.28%	0.09%	0.13%
d__Bacteria;p__Bacteroidota;c__Bacteroidia;o__Bacteroidales;f__Tannerellaceae;g__Parabacteroides;s__Parabacteroides_merdae	0.43%	0.75%	0.30%	0.40%
d__Bacteria;p__Firmicutes;c__Clostridia;o__Oscillospirales;f__Ruminococcaceae;g__Ruminococcus;s__uncultured_organism	0.32%	0.36%	0.24%	0.32%
d__Bacteria;p__Firmicutes;c__Clostridia;o__Lachnospirales;f__Lachnospiraceae;g__Agathobacter;s__uncultured_Clostridiales	1.09%	1.04%	0.67%	0.98%
d__Bacteria;p__Bacteroidota;c__Bacteroidia;o__Bacteroidales;f__Bacteroidaceae;g__Bacteroides;s__Bacteroides_ovatus	0.47%	0.55%	0.64%	1.50%
d__Bacteria;p__Bacteroidota;c__Bacteroidia;o__Bacteroidales;f__Rikenellaceae;g__Alistipes;s__uncultured_bacterium	0.56%	0.51%	0.37%	0.32%
d__Bacteria;p__Firmicutes;c__Clostridia;o__Lachnospirales;f__Lachnospiraceae;g__Lachnospiraceae_NK4A136_group;s__uncultured_organism	0.55%	0.76%	0.56%	0.57%
d__Bacteria;p__Firmicutes;c__Clostridia;o__Peptostreptococcales-Tissierellales;f__Peptostreptococcaceae;g__Romboutsia;s__uncultured_bacterium	0.43%	0.72%	0.66%	1.52%
d__Bacteria;p__Proteobacteria;c__Gammaproteobacteria;o__Burkholderiales;f__Comamonadaceae;g__Comamonas;s__Comamonas_kerstersii	0.00%	0.00%	0.80%	3.06%
d__Bacteria;p__Bacteroidota;c__Bacteroidia;o__Bacteroidales;f__Rikenellaceae;g__Alistipes;s__Alistipes_onderdonkii	0.56%	1.07%	0.18%	0.21%

d__Bacteria;p__Firmicutes;c__Clostridia;o__Oscillospirales;f__Oscillospiraceae;g__UCG-002;s__uncultured_organism	0.42%	0.59%	0.73%	0.95%
d__Bacteria;p__Firmicutes;c__Clostridia;o__Lachnospirales;f__Lachnospiraceae;g__Dorea;s__uncultured_bacterium	0.45%	0.45%	0.68%	0.49%
d__Bacteria;p__Firmicutes;c__Clostridia;o__Oscillospirales;f__Ruminococcaceae;g__Ruminococcus;s__Ruminococcus_bicirculans	0.50%	0.82%	0.25%	0.49%
d__Bacteria;p__Bacteroidota;c__Bacteroidia;o__Bacteroidales;f__Prevotellaceae;g__Prevotellaceae_NK3B31_group;s__uncultured_organism	0.42%	1.10%	0.55%	1.20%
d__Bacteria;p__Bacteroidota;c__Bacteroidia;o__Bacteroidales;f__Bacteroidaceae;g__Bacteroides;s__Bacteroides_cellulosilyticus	0.32%	0.62%	0.45%	0.61%
d__Bacteria;p__Firmicutes;c__Clostridia;o__Lachnospirales;f__Lachnospiraceae;g__[Eubacterium]_hallii_group;s__uncultured_bacterium	0.66%	0.68%	0.56%	0.56%
d__Bacteria;p__Firmicutes;c__Clostridia;o__Lachnospirales;f__Lachnospiraceae;g__[Ruminococcus]_torques_group;s__uncultured_bacterium	1.34%	1.41%	1.15%	0.88%
d__Bacteria;p__Actinobacteriota;c__Actinobacteria;o__Bifidobacteriales;f__Bifidobacteriaceae;g__Bifidobacterium;s__Bifidobacterium_adolescentis	0.42%	0.62%	0.18%	0.25%
d__Bacteria;p__Bacteroidota;c__Bacteroidia;o__Bacteroidales;f__Tannerellaceae;g__Parabacteroides;s__Parabacteroides_distasonis	0.22%	0.34%	0.30%	0.47%
d__Bacteria;p__Bacteroidota;c__Bacteroidia;o__Bacteroidales;f__Bacteroidaceae;g__Bacteroides;s__Bacteroides_kribbi	0.31%	0.49%	0.20%	0.36%
d__Bacteria;p__Firmicutes;c__Clostridia;o__Lachnospirales;f__Lachnospiraceae;g__Coprococcus;s__Coprococcus_sp.	0.14%	0.47%	0.32%	0.84%
d__Bacteria;p__Proteobacteria;c__Gammaproteobacteria;o__Burkholderiales;f__Comamonadaceae;g__Comamonas;s__Comamonas_aquatica	0.00%	0.00%	0.25%	0.97%
d__Bacteria;p__Firmicutes;c__Clostridia;o__Christensenellales;f__Christensenellaceae;g__Christensenellaceae_R-7_group;s__uncultured_bacterium	1.14%	2.60%	1.28%	1.90%
d__Bacteria;p__Bacteroidota;c__Bacteroidia;o__Bacteroidales;f__Tannerellaceae;g__Parabacteroides;s__uncultured_organism	0.34%	0.30%	0.47%	0.51%
d__Bacteria;p__Firmicutes;c__Clostridia;o__Lachnospirales;f__Lachnospiraceae;g__[Ruminococcus]_torques_group;s__uncultured_organism	0.36%	0.53%	0.26%	0.39%
d__Bacteria;p__Firmicutes;c__Clostridia;o__Oscillospirales;f__Oscillospiraceae;g__UCG-002;s__uncultured_bacterium	0.36%	0.41%	0.88%	1.07%
d__Bacteria;p__Firmicutes;c__Clostridia;o__Lachnospirales;f__Lachnospiraceae;g__Lachnospiraceae_UCG-008;s__uncultured_bacterium	0.47%	0.46%	0.40%	0.33%
d__Bacteria;p__Firmicutes;c__Clostridia;o__Lachnospirales;f__Lachnospiraceae;g__[Eubacterium]_eligens_group;s__[Eubacterium]_eligens	0.20%	0.29%	0.19%	0.31%
d__Bacteria;p__Proteobacteria;c__Gammaproteobacteria;o__Enterobacterales;f__Enterobacteriaceae;g__Citrobacter;s__bacterium_enrichment	0.23%	1.25%	0.21%	0.76%
d__Bacteria;p__Firmicutes;c__Clostridia;o__Clostridiales;f__Clostridiaceae;g__Clostridium_sensu_stricto_1;s__uncultured_bacterium	0.42%	1.01%	0.46%	1.03%
d__Bacteria;p__Bacteroidota;c__Bacteroidia;o__Bacteroidales;f__Prevotellaceae;g__Alloprevotella;s__uncultured_organism	0.28%	1.07%	0.42%	1.68%
d__Bacteria;p__Firmicutes;c__Clostridia;o__Lachnospirales;f__Lachnospiraceae;g__[Eubacterium]_eligens_group;s__uncultured_bacterium	0.25%	0.34%	0.14%	0.22%
d__Bacteria;p__Proteobacteria;c__Gammaproteobacteria;o__Enterobacterales;f__Erwiniaceae;g__Pantoea;s__uncultured_Erwinia	0.02%	0.05%	0.63%	2.90%
d__Bacteria;p__Firmicutes;c__Clostridia;o__Lachnospirales;f__Lachnospiraceae;g__Coprococcus;s__uncultured_bacterium	0.21%	0.40%	0.34%	0.45%
d__Bacteria;p__Actinobacteriota;c__Coriobacteriia;o__Coriobacteriales;f__Coriobacteriaceae;g__Collinsella;s__uncultured_bacterium	0.20%	0.29%	0.27%	0.60%
d__Bacteria;p__Proteobacteria;c__Gammaproteobacteria;o__Enterobacterales;f__Enterobacteriaceae;g__Escherichia-Shigella;s__Shigella_dysenteriae	0.18%	0.38%	0.16%	0.29%
d__Bacteria;p__Firmicutes;c__Clostridia;o__Lachnospirales;f__Lachnospiraceae;g__Lachnoclostridium;s__uncultured_organism	0.48%	0.41%	0.52%	0.57%
d__Bacteria;p__Firmicutes;c__Clostridia;o__Christensenellales;f__Christensenellaceae;g__Christensenellaceae_R-7_group;s__uncultured_organism	0.14%	0.53%	0.16%	0.35%
d__Bacteria;p__Proteobacteria;c__Gammaproteobacteria;o__Pseudomonadales;f__Pseudomonadaceae;g__Pseudomonas;s__Pseudomonas_aeruginosa	0.10%	0.54%	0.00%	0.00%
d__Bacteria;p__Proteobacteria;c__Gammaproteobacteria;o__Enterobacterales;f__Enterobacteriaceae;g__Citrobacter;s__Enterobacter_sp.	0.14%	0.77%	0.15%	0.54%
d__Bacteria;p__Bacteroidota;c__Bacteroidia;o__Bacteroidales;f__Bacteroidaceae;g__Bacteroides;s__Bacteroides_eggerthii	0.41%	0.95%	0.10%	0.20%

d__Bacteria;p__Firmicutes;c__Clostridia;o__Monoglobales;f__Monoglobaceae;g__Monoglobus;s__uncultured_organism	0.26%	0.35%	0.27%	0.33%
d__Bacteria;p__Firmicutes;c__Clostridia;o__Lachnospirales;f__Lachnospiraceae;g__[Ruminococcus]_torques_group;s__Ruminococcus_lactaris	0.16%	0.28%	0.24%	0.36%
d__Bacteria;p__Firmicutes;c__Clostridia;o__Oscillospirales;f__Ruminococcaceae;g__Incertae_Sedis;s__uncultured_bacterium	0.24%	0.20%	0.54%	1.24%
d__Bacteria;p__Firmicutes;c__Clostridia;o__Oscillospirales;f__Ruminococcaceae;g__Ruminococcus;s__metagenome	0.28%	0.42%	0.25%	0.49%
d__Bacteria;p__Proteobacteria;c__Alphaproteobacteria;o__Rhodospirillales;f__uncultured;g__uncultured;s__gut_metagenome	0.34%	1.08%	0.34%	1.03%
d__Bacteria;p__Bacteroidota;c__Bacteroidia;o__Bacteroidales;f__Bacteroidaceae;g__Bacteroides;s__bacterium_NLAE-zl-C233	0.36%	0.71%	0.11%	0.28%
d__Bacteria;p__Actinobacteriota;c__Actinobacteria;o__Bifidobacteriales;f__Bifidobacteriaceae;g__Bifidobacterium;s__metagenome	0.51%	0.67%	0.19%	0.30%
d__Bacteria;p__Firmicutes;c__Clostridia;o__Oscillospirales;f__Ruminococcaceae;g__CAG-352;s__uncultured_organism	0.68%	2.13%	0.08%	0.31%
d__Bacteria;p__Firmicutes;c__Clostridia;o__Oscillospirales;f__Ruminococcaceae;g__Ruminococcus;s__human_gut	0.11%	0.21%	0.23%	0.51%
d__Bacteria;p__Bacteroidota;c__Bacteroidia;o__Bacteroidales;f__Bacteroidaceae;g__Bacteroides;s__bacterium_NLAE-zl-P297	0.21%	0.36%	0.13%	0.25%
d__Bacteria;p__Firmicutes;c__Clostridia;o__Oscillospirales;f__Ruminococcaceae;g__Subdoligranulum;s__uncultured_organism	0.31%	0.34%	0.33%	0.38%
d__Bacteria;p__Firmicutes;c__Bacilli;o__Lactobacillales;f__Streptococcaceae;g__Streptococcus;s__Streptococcus_salivarius	0.18%	0.38%	0.12%	0.26%
d__Bacteria;p__Bacteroidota;c__Bacteroidia;o__Bacteroidales;f__Tannerellaceae;g__Parabacteroides;s__uncultured_bacterium	0.17%	0.24%	0.21%	0.39%
d__Bacteria;p__Firmicutes;c__Clostridia;o__Lachnospirales;f__Lachnospiraceae;g__[Eubacterium]_ruminantium_group;s__uncultured_bacterium	0.26%	0.68%	0.33%	0.97%
d__Bacteria;p__Bacteroidota;c__Bacteroidia;o__Bacteroidales;f__Rikenellaceae;g__Alistipes;s__gut_metagenome	0.20%	0.80%	0.14%	0.73%
d__Bacteria;p__Proteobacteria;c__Gammaproteobacteria;o__Enterobacterales;f__Enterobacteriaceae;g__Klebsiella;s__Klebsiella_oxytoca	0.00%	0.01%	0.08%	0.46%
d__Bacteria;p__Firmicutes;c__Clostridia;o__Oscillospirales;f__Oscillospiraceae;g__UCG-005;s__uncultured_organism	0.19%	0.34%	0.38%	0.46%
d__Bacteria;p__Bacteroidota;c__Bacteroidia;o__Bacteroidales;f__Bacteroidaceae;g__Bacteroides;s__Bacteroides_coprophilus	0.41%	1.25%	0.34%	0.87%
d__Bacteria;p__Firmicutes;c__Negativicutes;o__Acidaminococcales;f__Acidaminococcaceae;g__Phascolarctobacterium;s__Phascolarctobacterium_faecium	0.19%	0.39%	0.21%	0.39%
d__Bacteria;p__Proteobacteria;c__Gammaproteobacteria;o__Burkholderiales;f__Comamonadaceae;g__Comamonas;s__Comamonas_terrae	0.00%	0.00%	0.62%	2.37%
d__Bacteria;p__Firmicutes;c__Bacilli;o__Erysipelotrichales;f__Erysipelotrichaceae;g__Turicibacter;s__Turicibacter_sp.	0.15%	0.43%	0.07%	0.14%
d__Bacteria;p__Firmicutes;c__Clostridia;o__Oscillospirales;f__Oscillospiraceae;g__Colidextribacter;s__uncultured_bacterium	0.21%	0.25%	0.29%	0.46%
d__Bacteria;p__Bacteroidota;c__Bacteroidia;o__Bacteroidales;f__Rikenellaceae;g__Alistipes;s__unidentified	0.19%	0.40%	0.05%	0.07%
d__Bacteria;p__Actinobacteriota;c__Actinobacteria;o__Bifidobacteriales;f__Bifidobacteriaceae;g__Bifidobacterium;s__Bifidobacterium_pseudocatenulatum	0.27%	0.59%	0.25%	0.56%
d__Bacteria;p__Bacteroidota;c__Bacteroidia;o__Bacteroidales;f__Tannerellaceae;g__Parabacteroides;s__Parabacteroides_johnsonii	0.26%	0.43%	0.32%	0.75%
d__Bacteria;p__Bacteroidota;c__Bacteroidia;o__Bacteroidales;f__Tannerellaceae;g__Parabacteroides;s__Parabacteroides_sp.	0.07%	0.13%	0.18%	0.35%
d__Bacteria;p__Proteobacteria;c__Gammaproteobacteria;o__Enterobacterales;f__Enterobacteriaceae;g__Klebsiella;s__Klebsiella_variicola	0.03%	0.07%	0.13%	0.37%
d__Bacteria;p__Bacteroidota;c__Bacteroidia;o__Bacteroidales;f__Prevotellaceae;g__Paraprevotella;s__uncultured_bacterium	0.48%	0.97%	0.23%	0.39%
d__Bacteria;p__Firmicutes;c__Negativicutes;o__Veillonellales-Selenomonadales;f__Veillonellaceae;g__Dialister;s__uncultured_organism	0.17%	0.31%	0.11%	0.25%
d__Bacteria;p__Firmicutes;c__Clostridia;o__Lachnospirales;f__Lachnospiraceae;g__[Ruminococcus]_gauvreauii_group;s__uncultured_bacterium	0.15%	0.22%	0.20%	0.32%
d__Bacteria;p__Proteobacteria;c__Gammaproteobacteria;o__Pseudomonadales;f__Moraxellaceae;g__Acinetobacter;s__Acinetobacter_johnsonii	0.00%	0.00%	0.07%	0.27%

d__Bacteria;p__Bacteroidota;c__Bacteroidia;o__Bacteroidales;f__Rikenellaceae;g__Alistipes;s__Alistipes_finegoldii	0.12%	0.28%	0.10%	0.19%
d__Bacteria;p__Firmicutes;c__Bacilli;o__Lactobacillales;f__Lactobacillaceae;g__Lactobacillus;s__Lactobacillus_sakei	0.00%	0.02%	0.33%	1.82%
d__Bacteria;p__Firmicutes;c__Clostridia;o__Oscillospirales;f__Oscillospiraceae;g__UCG-005;s__uncultured_bacterium	0.17%	0.25%	0.18%	0.19%
d__Bacteria;p__Firmicutes;c__Clostridia;o__Oscillospirales;f__Oscillospiraceae;g__NK4A214_group;s__metagenome	0.10%	0.24%	0.14%	0.37%
d__Bacteria;p__Firmicutes;c__Clostridia;o__Lachnospirales;f__Lachnospiraceae;g__[Eubacterium]_eligens_group;s__uncultured_organism	0.25%	0.41%	0.20%	0.31%
d__Bacteria;p__Proteobacteria;c__Gammaproteobacteria;o__Burkholderiales;f__Sutterellaceae;g__Sutterella;s__uncultured_bacterium	0.51%	1.10%	0.38%	0.45%
d__Bacteria;p__Firmicutes;c__Clostridia;o__Clostridiales;f__Clostridiaceae;g__Clostridium_sensu_stricto_1;s__uncultured_Clostridium	0.09%	0.31%	0.09%	0.34%
d__Bacteria;p__Proteobacteria;c__Gammaproteobacteria;o__Pasteurellales;f__Pasteurellaceae;g__Haemophilus;s__uncultured_bacterium	0.14%	0.27%	0.15%	0.59%
d__Bacteria;p__Bacteroidota;c__Bacteroidia;o__Bacteroidales;f__Bacteroidaceae;g__Bacteroides;s__bacterium_New	0.08%	0.41%	0.12%	0.53%
d__Bacteria;p__Firmicutes;c__Clostridia;o__Peptostreptococcales-Tissierellales;f__Peptostreptococcaceae;g__Terrisporobacter;s__uncultured_bacterium	0.09%	0.28%	0.04%	0.08%
d__Bacteria;p__Firmicutes;c__Clostridia;o__Oscillospirales;f__[Eubacterium]_coprostanoligenes_group;g__[Eubacterium]_coprostanoligenes_group;s__uncultured_organism	0.07%	0.15%	0.19%	0.31%
d__Bacteria;p__Firmicutes;c__Clostridia;o__Oscillospirales;f__Oscillospiraceae;g__UCG-002;s__gut_metagenome	0.10%	0.23%	0.12%	0.30%
d__Bacteria;p__Firmicutes;c__Clostridia;o__Lachnospirales;f__Lachnospiraceae;g__Anaerostipes;s__Anaerostipes_hadrus	0.06%	0.12%	0.08%	0.20%
d__Bacteria;p__Firmicutes;c__Negativicutes;o__Veillonellales-Selenomonadales;f__Veillonellaceae;g__Dialister;s__Dialister_sp.	0.17%	0.61%	0.15%	0.49%
d__Bacteria;p__Firmicutes;c__Clostridia;o__Clostridiales;f__Clostridiaceae;g__Clostridium_sensu_stricto_1;s__uncultured_organism	0.06%	0.21%	0.05%	0.17%
d__Bacteria;p__Firmicutes;c__Clostridia;o__Peptostreptococcales-Tissierellales;f__Peptostreptococcaceae;g__Intestinibacter;s__Intestinibacter_bartlettii	0.12%	0.46%	0.03%	0.08%
d__Bacteria;p__Firmicutes;c__Negativicutes;o__Veillonellales-Selenomonadales;f__Veillonellaceae;g__Dialister;s__uncultured_bacterium	0.12%	0.47%	0.14%	0.49%
d__Bacteria;p__Firmicutes;c__Bacilli;o__Lactobacillales;f__Streptococcaceae;g__Streptococcus;s__uncultured_bacterium	0.07%	0.22%	0.05%	0.12%
d__Bacteria;p__Firmicutes;c__Bacilli;o__Lactobacillales;f__Enterococcaceae;g__Enterococcus;s__Enterococcus_faecium	0.06%	0.15%	0.11%	0.32%
d__Bacteria;p__Firmicutes;c__Bacilli;o__Bacillales;f__Planococcaceae;g__Lysinibacillus;s__Lysinibacillus_sphaericus	0.00%	0.00%	0.20%	1.09%
d__Bacteria;p__Campilobacterota;c__Campylobacteria;o__Campylobacterales;f__Campylobacteraceae;g__Campylobacter;s__Campylobacter_hominis	0.00%	0.00%	0.33%	1.21%
d__Bacteria;p__Firmicutes;c__Clostridia;o__Oscillospirales;f__Ruminococcaceae;g__Ruminococcus;s__uncultured_Ruminococcaceae	0.10%	0.31%	0.04%	0.20%
d__Bacteria;p__Proteobacteria;c__Gammaproteobacteria;o__Burkholderiales;f__Sutterellaceae;g__Sutterella;s__uncultured_organism	0.16%	0.64%	0.03%	0.09%
d__Bacteria;p__Firmicutes;c__Clostridia;o__Oscillospirales;f__Ruminococcaceae;g__uncultured;s__gut_metagenome	0.07%	0.18%	0.10%	0.25%
d__Bacteria;p__Firmicutes;c__Bacilli;o__Erysipelotrichales;f__Erysipelotrichaceae;g__Turicibacter;s__uncultured_bacterium	0.17%	0.37%	0.12%	0.25%
d__Bacteria;p__Firmicutes;c__Clostridia;o__Clostridia_UCG-014;f__Clostridia_UCG-014;g__Clostridia_UCG-014;s__gut_metagenome	0.12%	0.28%	0.22%	0.38%
d__Bacteria;p__Proteobacteria;c__Gammaproteobacteria;o__Enterobacterales;f__Enterobacteriaceae;g__Raoultella;s__Raoultella_ornithinolytica	0.00%	0.01%	0.15%	0.54%
d__Bacteria;p__Firmicutes;c__Bacilli;o__Erysipelotrichales;f__Erysipelatoclostridiaceae;g__Catenibacterium;s__uncultured_bacterium	0.04%	0.16%	0.20%	0.54%
d__Bacteria;p__Firmicutes;c__Clostridia;o__Oscillospirales;f__Oscillospiraceae;g__NK4A214_group;s__uncultured_organism	0.04%	0.16%	0.09%	0.33%
d__Bacteria;p__Firmicutes;c__Clostridia;o__Oscillospirales;f__[Eubacterium]_coprostanoligenes_group;g__[Eubacterium]_coprostanoligenes_group;s__gut_metagenome	0.13%	0.31%	0.12%	0.23%
d__Bacteria;p__Firmicutes;c__Clostridia;o__Christensenellales;f__Christensenellaceae;g__Christensenellaceae_R-7_group;s__gut_metagenome	0.08%	0.29%	0.09%	0.32%

d__Bacteria;p__Bacteroidota;c__Bacteroidia;o__Bacteroidales;f__Barnesiellaceae;g__Barnesiella;s__uncultured_organism	0.09%	0.47%	0.12%	0.51%
d__Bacteria;p__Firmicutes;c__Negativicutes;o__Acidaminococcales;f__Acidaminococcaceae;g__Phascolarctobacterium;s__uncultured_Firmicutes	0.10%	0.39%	0.09%	0.22%
d__Bacteria;p__Firmicutes;c__Clostridia;o__Christensenellales;f__Christensenellaceae;g__Christensenellaceae_R-7_group;s__metagenome	0.15%	0.19%	0.15%	0.24%
d__Bacteria;p__Bacteroidota;c__Bacteroidia;o__Bacteroidales;f__Bacteroidaceae;g__Bacteroides;s__metagenome	0.21%	1.11%	0.00%	0.00%
d__Bacteria;p__Bacteroidota;c__Bacteroidia;o__Bacteroidales;f__Bacteroidaceae;g__Bacteroides;s__uncultured_Bacteroidales	0.17%	0.76%	0.16%	0.51%
d__Bacteria;p__Proteobacteria;c__Gammaproteobacteria;o__Xanthomonadales;f__Xanthomonadaceae;g__Stenotrophomonas;s__Stenotrophomonas_acidaminiphila	0.07%	0.36%	0.00%	0.00%
d__Bacteria;p__Firmicutes;c__Clostridia;o__Oscillospirales;f__[Eubacterium]_coprostanoligenes_group;g__[Eubacterium]_coprostanoligenes_group;s__unidentified	0.11%	0.24%	0.04%	0.14%
d__Bacteria;p__Bacteroidota;c__Bacteroidia;o__Bacteroidales;f__Bacteroidaceae;g__Bacteroides;s__Bacteroides_sp.	0.18%	0.54%	0.09%	0.38%
d__Bacteria;p__Bacteroidota;c__Bacteroidia;o__Bacteroidales;f__Prevotellaceae;g__Prevotella;s__Prevotella_disiens	0.00%	0.00%	0.19%	0.67%
d__Bacteria;p__Firmicutes;c__Clostridia;o__Clostridiales;f__Clostridiaceae;g__Clostridium_sensu_stricto_1;s__Ruminococcus_sp.	0.05%	0.22%	0.07%	0.39%
d__Bacteria;p__Firmicutes;c__Clostridia;o__Lachnospirales;f__Lachnospiraceae;g__Lachnospiraceae_NC2004_group;s__uncultured_bacterium	0.03%	0.08%	0.07%	0.22%
d__Bacteria;p__Proteobacteria;c__Gammaproteobacteria;o__Burkholderiales;f__Sutterellaceae;g__Sutterella;s__Sutterellaceae_bacterium	0.05%	0.16%	0.07%	0.22%
d__Bacteria;p__Firmicutes;c__Clostridia;o__Lachnospirales;f__Lachnospiraceae;g__Agathobacter;s__uncultured_organism	0.07%	0.20%	0.06%	0.14%
d__Bacteria;p__Firmicutes;c__Clostridia;o__Lachnospirales;f__Lachnospiraceae;g__Anaerostipes;s__uncultured_organism	0.09%	0.19%	0.09%	0.33%
d__Bacteria;p__Firmicutes;c__Clostridia;o__Lachnospirales;f__Lachnospiraceae;g__Blautia;s__Blautia_glucerasea	0.00%	0.00%	0.05%	0.24%
d__Bacteria;p__Firmicutes;c__Clostridia;o__Clostridia_UCG-014;f__Clostridia_UCG-014;g__Clostridia_UCG-014;s__uncultured_organism	0.05%	0.12%	0.07%	0.22%
d__Bacteria;p__Proteobacteria;c__Gammaproteobacteria;o__Pasteurellales;f__Pasteurellaceae;g__Haemophilus;s__uncultured_organism	0.06%	0.17%	0.07%	0.34%
d__Bacteria;p__Firmicutes;c__Bacilli;o__Lactobacillales;f__Lactobacillaceae;g__Lactobacillus;s__uncultured_bacterium	0.00%	0.00%	0.10%	0.56%
d__Bacteria;p__Bacteroidota;c__Bacteroidia;o__Bacteroidales;f__Bacteroidaceae;g__Bacteroides;s__uncultured_Bacteroides	0.03%	0.14%	0.15%	0.64%
d__Bacteria;p__Firmicutes;c__Clostridia;o__Clostridia_vadinBB60_group;f__Clostridia_vadinBB60_group;g__Clostridia_vadinBB60_group;s__uncultured_organism	0.05%	0.14%	0.15%	0.75%
d__Bacteria;p__Bacteroidota;c__Bacteroidia;o__Bacteroidales;f__Muribaculaceae;g__Muribaculaceae;s__uncultured_bacterium	0.05%	0.15%	0.32%	0.63%
d__Bacteria;p__Bacteroidota;c__Bacteroidia;o__Bacteroidales;f__Prevotellaceae;g__Prevotella;s__Prevotella_timonensis	0.00%	0.00%	0.09%	0.28%
d__Bacteria;p__Firmicutes;c__Clostridia;o__Clostridia_UCG-014;f__Clostridia_UCG-014;g__Clostridia_UCG-014;s__uncultured_bacterium	0.23%	0.66%	0.20%	0.42%
d__Bacteria;p__Firmicutes;c__Clostridia;o__Oscillospirales;f__Ruminococcaceae;g__[Eubacterium]_siraeum_group;s__[Eubacterium]_siraeum	0.02%	0.09%	0.09%	0.28%
d__Bacteria;p__Firmicutes;c__Clostridia;o__Oscillospirales;f__Ruminococcaceae;g__uncultured;s__uncultured_bacterium	0.35%	0.93%	0.34%	0.78%
d__Bacteria;p__Firmicutes;c__Bacilli;o__Bacillales;f__Planococcaceae;g__Lysinibacillus;s__Bacillus_sp.	0.00%	0.00%	0.08%	0.44%
d__Bacteria;p__Firmicutes;c__Clostridia;o__Lachnospirales;f__Lachnospiraceae;g__Hungatella;s__uncultured_organism	0.12%	0.62%	0.02%	0.06%
d__Bacteria;p__Firmicutes;c__Clostridia;o__Peptostreptococcales-Tissierellales;f__Peptostreptococcaceae;g__Terrisporobacter;s__uncultured_organism	0.05%	0.21%	0.01%	0.03%
d__Bacteria;p__Proteobacteria;c__Gammaproteobacteria;o__Enterobacterales;f__Enterobacteriaceae;g__Raoultella;s__bacterium_E2-20	0.00%	0.00%	0.07%	0.27%
d__Bacteria;p__Firmicutes;c__Clostridia;o__Clostridiales;f__Clostridiaceae;g__Clostridium_sensu_stricto_1;s__human_gut	0.00%	0.02%	0.07%	0.22%
d__Bacteria;p__Firmicutes;c__Clostridia;o__Clostridia_UCG-014;f__Clostridia_UCG-014;g__Clostridia_UCG-014;s__uncultured_Clostridiales	0.00%	0.00%	0.10%	0.52%

d__Bacteria;p__Firmicutes;c__Clostridia;o__Peptostreptococcales-Tissierellales;f__Peptostreptococcaceae;g__Intestinibacter;s__uncultured_organism	0.06%	0.27%	0.01%	0.03%
d__Bacteria;p__Proteobacteria;c__Gammaproteobacteria;o__Xanthomonadales;f__Xanthomonadaceae;g__Stenotrophomonas;s__uncultured_Xanthomonadales	0.03%	0.19%	0.00%	0.00%
d__Bacteria;p__Firmicutes;c__Clostridia;o__Peptostreptococcales-Tissierellales;f__Peptostreptococcales-Tissierellales;g__Peptoniphilus;s__Peptoniphilus_lacrimalis	0.00%	0.00%	0.10%	0.35%
d__Bacteria;p__Firmicutes;c__Clostridia;o__Oscillospirales;f__Ruminococcaceae;g__Incertae_Sedis;s__[Clostridium]_leptum	0.02%	0.03%	0.07%	0.26%
d__Bacteria;p__Firmicutes;c__Clostridia;o__Peptostreptococcales-Tissierellales;f__Peptostreptococcales-Tissierellales;g__Murdochiella;s__Murdochiella_asaccharolytica	0.00%	0.00%	0.07%	0.24%
d__Bacteria;p__Firmicutes;c__Negativicutes;o__Veillonellales-Selenomonadales;f__Veillonellaceae;g__Veillonella;s__uncultured_bacterium	0.03%	0.06%	0.13%	0.58%
d__Bacteria;p__Firmicutes;c__Bacilli;o__RF39;f__RF39;g__RF39;s__uncultured_bacterium	0.06%	0.29%	0.05%	0.09%
d__Bacteria;p__Proteobacteria;c__Gammaproteobacteria;o__Pseudomonadales;f__Pseudomonadaceae;g__Pseudomonas;s__Pseudomonas_mosselii	0.00%	0.01%	0.04%	0.19%
d__Bacteria;p__Firmicutes;c__Clostridia;o__Lachnospirales;f__Lachnospiraceae;g__Anaerosporobacter;s__uncultured_organism	0.00%	0.00%	0.06%	0.21%
d__Bacteria;p__Bacteroidota;c__Bacteroidia;o__Bacteroidales;f__Bacteroidaceae;g__Bacteroides;s__Bacteroides_intestinalis	0.04%	0.16%	0.05%	0.21%
d__Bacteria;p__Fusobacteriota;c__Fusobacteriia;o__Fusobacteriales;f__Fusobacteriaceae;g__Fusobacterium;s__uncultured_bacterium	0.04%	0.20%	0.09%	0.38%
d__Bacteria;p__Bacteroidota;c__Bacteroidia;o__Bacteroidales;f__Prevotellaceae;g__Prevotella;s__Prevotella_colorans	0.00%	0.00%	0.07%	0.23%
d__Bacteria;p__Firmicutes;c__Clostridia;o__Peptostreptococcales-Tissierellales;f__Peptostreptococcales-Tissierellales;g__Anaerococcus;s__uncultured_organism	0.00%	0.00%	0.07%	0.28%
d__Bacteria;p__Firmicutes;c__Clostridia;o__Oscillospirales;f__Ruminococcaceae;g__Ruminococcus;s__gut_metagenome	0.00%	0.01%	0.12%	0.61%
d__Bacteria;p__Desulfobacterota;c__Desulfovibrionia;o__Desulfovibrionales;f__Desulfovibrionaceae;g__Desulfovibrio;s__Desulfovibrio_desulfuricans	0.01%	0.02%	0.04%	0.21%
d__Bacteria;p__Bacteroidota;c__Bacteroidia;o__Bacteroidales;f__Bacteroidaceae;g__Bacteroides;s__Bacteroides_salyersiae	0.06%	0.23%	0.04%	0.12%
d__Bacteria;p__Desulfobacterota;c__Desulfovibrionia;o__Desulfovibrionales;f__Desulfovibrionaceae;g__Desulfovibrio;s__uncultured_bacterium	0.00%	0.02%	0.04%	0.21%
d__Bacteria;p__Firmicutes;c__Clostridia;o__Clostridia_vadinBB60_group;f__Clostridia_vadinBB60_group;g__Clostridia_vadinBB60_group;s__uncultured_bacterium	0.03%	0.09%	0.07%	0.24%
d__Bacteria;p__Actinobacteriota;c__Coriobacteriia;o__Coriobacteriales;f__Atopobiaceae;g__Atopobium;s__Atopobium_vaginae	0.00%	0.00%	0.08%	0.42%
d__Bacteria;p__Firmicutes;c__Bacilli;o__Erysipelotrichales;f__Erysipelotrichaceae;g__Holdemanella;s__uncultured_bacterium	0.00%	0.01%	0.06%	0.24%
d__Bacteria;p__Bacteroidota;c__Bacteroidia;o__Bacteroidales;f__Bacteroidaceae;g__Bacteroides;s__Mogibacterium_sp.	0.00%	0.00%	0.06%	0.33%
d__Bacteria;p__Firmicutes;c__Clostridia;o__Lachnospirales;f__Lachnospiraceae;g__Anaerosporobacter;s__uncultured_bacterium	0.06%	0.30%	0.00%	0.00%
d__Bacteria;p__Bacteroidota;c__Bacteroidia;o__Bacteroidales;f__Prevotellaceae;g__Prevotellaceae_Ga6A1_group;s__uncultured_bacterium	0.00%	0.00%	0.04%	0.20%
d__Bacteria;p__Bacteroidota;c__Bacteroidia;o__Bacteroidales;f__Prevotellaceae;g__Prevotella;s__uncultured_organism	0.00%	0.00%	0.04%	0.24%
Other	7.75%	1.99%	8.91%	3.22%
Unassigned	9.57%	3.31%	10.84%	4.33%

Table S4. From phylum to species are shown the relative abundances of most abundant taxa (i.e. taxa with a relative abundance ≥ 1% in at least one sample). In particular, for each taxa the scientific name**,** the mean and standard deviation in both F and CWF are reported.

Table S5

Taxa	Exclusively in F	Exclusively in CWF	Raw F count	Not Observed in F	Raw CWF prev	Not Observed in CWF	Rank	Score	F prev perc	CWF prev perc	Abn Score
d__Bacteria;p__Proteobacteria;c__Gammaproteobacteria;o__Burkholderiales;f__Burkholderiaceae;g__Burkholderia-Caballeronia-Paraburkholderia	15	0	15	15	0	30	genus	15	50.00%	0.00%	15
d__Bacteria;p__Proteobacteria;c__Gammaproteobacteria;o__Burkholderiales;f__Burkholderiaceae	15	2	16	14	3	27	family	13	53.33%	10.00%	13
d__Bacteria;p__Proteobacteria;c__Gammaproteobacteria;o__Burkholderiales;f__Burkholderiaceae;g__Burkholderia-Caballeronia-Paraburkholderia;s__Burkholderia_pseudomallei	13	0	13	17	0	30	species	13	43.33%	0.00%	13
d__Bacteria;p__Actinobacteriota;c__Actinobacteria;o__Propionibacteriales	11	1	11	19	1	29	order	10	36.67%	3.33%	10
d__Bacteria;p__Actinobacteriota;c__Actinobacteria;o__Propionibacteriales;f__Propionibacteriaceae	11	1	11	19	1	29	family	10	36.67%	3.33%	10
d__Bacteria;p__Firmicutes;c__Negativicutes;o__Acidaminococcales;f__Acidaminococcaceae;g__Phascolarctobacterium	3	13	15	15	25	5	genus	-10	50.00%	83.33%	10
d__Bacteria;p__Actinobacteriota;c__Actinobacteria;o__Propionibacteriales;f__Propionibacteriaceae;g__Cutibacterium	11	1	11	19	1	29	genus	10	36.67%	3.33%	10
d__Bacteria;p__Firmicutes;c__Clostridia;o__Oscillospirales;f__Oscillospiraceae;g__UCG-002;s__gut_metagenome	15	5	20	10	10	20	species	10	66.67%	33.33%	10
d__Bacteria;p__Firmicutes;c__Bacilli;o__Staphylococcales;f__Gemellaceae;g__Gemella	12	3	14	16	5	25	genus	9	46.67%	16.67%	9
d__Bacteria;p__Firmicutes;c__Clostridia;o__Lachnospirales;f__Lachnospiraceae;g__[Eubacterium]_fissicatena_group	12	3	13	17	4	26	genus	9	43.33%	13.33%	9
d__Bacteria;p__Firmicutes;c__Clostridia;o__Oscillospirales;f__[Eubacterium]_coprostanoligenes_group;g__[Eubacterium]_coprostanoligenes_group;s__gut_metagenome	4	13	8	22	17	13	species	-9	26.67%	56.67%	9
d__Bacteria;p__Bacteroidota;c__Bacteroidia;o__Bacteroidales;f__Rikenellaceae;g__Alistipes;s__unidentified	12	3	22	8	13	17	species	9	73.33%	43.33%	9
d__Bacteria;p__Firmicutes;c__Clostridia;o__Lachnospirales;f__Lachnospiraceae;g__Coprococcus;s__Coprococcus_sp.	10	1	12	18	3	27	species	9	40.00%	10.00%	9
d__Bacteria;p__Firmicutes;c__Negativicutes;o__Acidaminococcales	3	11	17	13	25	5	order	-8	56.67%	83.33%	8
d__Bacteria;p__Cyanobacteria;c__Vampirivibrionia;o__Gastranaerophilales	10	2	11	19	3	27	order	8	36.67%	10.00%	8
d__Bacteria;p__Firmicutes;c__Negativicutes;o__Acidaminococcales;f__Acidaminococcaceae	3	11	17	13	25	5	family	-8	56.67%	83.33%	8
d__Bacteria;p__Firmicutes;c__Clostridia;o__Oscillospirales;f__Oscillospiraceae;g__Intestinimonas	10	2	28	2	20	10	genus	8	93.33%	66.67%	8
d__Bacteria;p__Firmicutes;c__Bacilli;o__Erysipelotrichales;f__Erysipelotrichaceae;g__Holdemania;s__Holdemania_filiformis	3	11	18	12	26	4	species	-8	60.00%	86.67%	8
d__Bacteria;p__Firmicutes;c__Negativicutes;o__Acidaminococcales;f__Acidaminococcaceae;g__Phascolarctobacterium;s__Phascolarctobacterium_faecium	6	14	6	24	14	16	species	-8	20.00%	46.67%	8
d__Bacteria;p__Firmicutes;c__Clostridia;o__Lachnospirales;f__Lachnospiraceae;g__Lachnoclostridium;s__[Clostridium_asparagiforme]	13	5	17	13	9	21	species	8	56.67%	30.00%	8
d__Bacteria;p__Fusobacteriota	8	1	10	20	3	27	phylum	7	33.33%	10.00%	7
d__Bacteria;p__Actinobacteriota;c__Coriobacteriia;o__Coriobacteriales;f__Coriobacteriales_Incertae_Sedis	3	10	16	14	23	7	family	-7	53.33%	76.67%	7
d__Bacteria;p__Cyanobacteria;c__Vampirivibrionia;o__Gastranaerophilales;f__Gastranaerophilales	9	2	10	20	3	27	family	7	33.33%	10.00%	7
d__Bacteria;p__Firmicutes;c__Bacilli;o__Lactobacillales;f__Carnobacteriaceae	9	2	13	17	6	24	family	7	43.33%	20.00%	7
d__Bacteria;p__Firmicutes;c__Clostridia;o__Oscillospirales;f__Ruminococcaceae;g__[Eubacterium]_siraeum_group	11	4	24	6	17	13	genus	7	80.00%	56.67%	7
d__Bacteria;p__Firmicutes;c__Bacilli;o__Lactobacillales;f__Carnobacteriaceae;g__Granulicatella	9	2	13	17	6	24	genus	7	43.33%	20.00%	7

d__Bacteria;p__Firmicutes;c__Clostridia;o__Lachnospirales;f__Lachnospiraceae;g__[Eubacterium]_ruminantium_group	4	11	6	24	13	17	genus	-7	20.00%	43.33%	7
d__Bacteria;p__Cyanobacteria;c__Vampirivibrionia;o__Gastranaerophilales;f__Gastranaerophilales;g__Gastranaerophilales	9	2	10	20	3	27	genus	7	33.33%	10.00%	7
d__Bacteria;p__Firmicutes;c__Clostridia;o__Lachnospirales;f__Lachnospiraceae;g__[Eubacterium]_oxidoreducens_group	10	3	12	18	5	25	genus	7	40.00%	16.67%	7
d__Bacteria;p__Firmicutes;c__Clostridia;o__Oscillospirales;f__Ruminococcaceae;s__gut_metagenome	8	1	11	19	4	26	species	7	36.67%	13.33%	7
d__Bacteria;p__Firmicutes;c__Bacilli;o__Lactobacillales;f__Streptococcaceae;g__Streptococcus;s__Streptococcus_oralis	9	2	9	21	2	28	species	7	30.00%	6.67%	7
d__Bacteria;p__Firmicutes;c__Clostridia;o__Oscillospirales;f__Oscillospiraceae;g__NK4A214_group;s__metagenome	4	11	12	18	19	11	species	-7	40.00%	63.33%	7
d__Bacteria;p__Actinobacteriota;c__Actinobacteria;o__Propionibacteriales;f__Propionibacteriaceae;g__Cutibacterium;s__Cutibacterium_acnes	7	0	7	23	0	30	species	7	23.33%	0.00%	7
d__Bacteria;p__Fusobacteriota;c__Fusobacteriia	8	2	9	21	3	27	class	6	30.00%	10.00%	6
d__Bacteria;p__Fusobacteriota;c__Fusobacteriia;o__Fusobacteriales	7	1	9	21	3	27	order	6	30.00%	10.00%	6
d__Bacteria;p__Fusobacteriota;c__Fusobacteriia;o__Fusobacteriales;f__Fusobacteriaceae	7	1	9	21	3	27	family	6	30.00%	10.00%	6
d__Bacteria;p__Firmicutes;c__Bacilli;o__Lactobacillales;f__Enterococcaceae	5	11	11	19	17	13	family	-6	36.67%	56.67%	6
d__Bacteria;p__Firmicutes;c__Bacilli;o__Staphylococcales;f__Gemellaceae	11	5	13	17	7	23	family	6	43.33%	23.33%	6
d__Bacteria;p__Synergistota;c__Synergistia;o__Synergistales;f__Synergistaceae;g__Pyramidobacter	7	1	7	23	1	29	genus	6	23.33%	3.33%	6
d__Bacteria;p__Firmicutes;c__Bacilli;o__Lactobacillales;f__Enterococcaceae;g__Enterococcus	5	11	11	19	17	13	genus	-6	36.67%	56.67%	6
d__Bacteria;p__Bacteroidota;c__Bacteroidia;o__Bacteroidales;f__Prevotellaceae;g__Prevotella	11	5	17	13	11	19	genus	6	56.67%	36.67%	6
d__Bacteria;p__Fusobacteriota;c__Fusobacteriia;o__Fusobacteriales;f__Fusobacteriaceae;g__Fusobacterium	7	1	9	21	3	27	genus	6	30.00%	10.00%	6
d__Bacteria;p__Firmicutes;c__Clostridia;o__Lachnospirales;f__Lachnospiraceae;g__Lachnoclostridium;s__bacterium_NLAE-zl-P872	10	4	14	16	8	22	species	6	46.67%	26.67%	6
d__Bacteria;p__Synergistota;c__Synergistia;o__Synergistales;f__Synergistaceae;g__Pyramidobacter;s__Pyramidobacter_piscolens	6	0	6	24	0	30	species	6	20.00%	0.00%	6
d__Bacteria;p__Firmicutes;c__Clostridia;o__Lachnospirales;f__Lachnospiraceae;g__Dorea;s__Dorea_formicigenerans	2	8	20	10	26	4	species	-6	66.67%	86.67%	6
d__Bacteria;p__Firmicutes;c__Clostridia;o__Oscillospirales;f__Oscillospiraceae;s__Pseudoflavonifractor_sp.	6	0	6	24	0	30	species	6	20.00%	0.00%	6
d__Bacteria;p__Firmicutes;c__Bacilli;o__Lactobacillales;f__Enterococcaceae;g__Enterococcus;s__Enterococcus_faecium	4	10	7	23	13	17	species	-6	23.33%	43.33%	6
d__Bacteria;p__Firmicutes;c__Clostridia;o__Lachnospirales;f__Lachnospiraceae;g__Agathobacter;s__Eubacterium_ramulus	5	11	12	18	18	12	species	-6	40.00%	60.00%	6
d__Bacteria;p__Bacteroidota;c__Bacteroidia;o__Bacteroidales;f__Tannerellaceae;g__Parabacteroides;s__Parabacteroides_johnsonii	12	6	22	8	16	14	species	6	73.33%	53.33%	6
d__Bacteria;p__Firmicutes;c__Bacilli;o__Lactobacillales;f__Streptococcaceae;g__Streptococcus;s__bacterium_enrichment	8	2	8	22	2	28	species	6	26.67%	6.67%	6
d__Bacteria;p__Bacteroidota;c__Bacteroidia;o__Bacteroidales;f__Rikenellaceae;g__Alistipes;s__Bacteroides_sp.	3	9	3	27	9	21	species	-6	10.00%	30.00%	6
d__Bacteria;p__Cyanobacteria	7	2	8	22	3	27	phylum	5	26.67%	10.00%	5
d__Bacteria;p__Cyanobacteria;c__Vampirivibrionia	7	2	8	22	3	27	class	5	26.67%	10.00%	5
d__Bacteria;p__Firmicutes;c__Bacilli;o__RF39	9	4	14	16	9	21	order	5	46.67%	30.00%	5
d__Bacteria;p__Campilobacterota;c__Campylobacteria;o__Campylobacterales	3	8	4	26	9	21	order	-5	13.33%	30.00%	5
d__Bacteria;p__Firmicutes;c__Bacilli;o__RF39;f__RF39	9	4	14	16	9	21	family	5	46.67%	30.00%	5
d__Bacteria;p__Firmicutes;c__Clostridia;o__Oscillospirales;f__UCG-010	9	4	20	10	15	15	family	5	66.67%	50.00%	5

d__Bacteria;p__Firmicutes;c__Negativicutes;o__Veillonellales-Selenomonadales;f__Selenomonadaceae	2	7	2	28	7	23	family	-5	6.67%	23.33%	5
d__Bacteria;p__Firmicutes;c__Clostridia;o__Peptostreptococcales-Tissierellales;f__Anaerovoracaceae;g__Family_XIII_UCG-001	7	2	25	5	20	10	genus	5	83.33%	66.67%	5
d__Bacteria;p__Firmicutes;c__Clostridia;o__Lachnospirales;f__Defluviitaleaceae;g__Defluviitaleaceae_UCG-011	8	3	22	8	17	13	genus	5	73.33%	56.67%	5
d__Bacteria;p__Proteobacteria;c__Gammaproteobacteria;o__Enterobacterales;f__Enterobacteriaceae;g__Klebsiella	2	7	7	23	12	18	genus	-5	23.33%	40.00%	5
d__Bacteria;p__Actinobacteriota;c__Coriobacteriia;o__Coriobacteriales;f__Eggerthellaceae;g__Adlercreutzia	4	9	18	12	23	7	genus	-5	60.00%	76.67%	5
d__Bacteria;p__Firmicutes;c__Clostridia;o__Oscillospirales;f__UCG-010;g__UCG-010	9	4	20	10	15	15	genus	5	66.67%	50.00%	5
d__Bacteria;p__Firmicutes;c__Bacilli;o__RF39;f__RF39;g__RF39	9	4	14	16	9	21	genus	5	46.67%	30.00%	5
d__Bacteria;p__Firmicutes;c__Clostridia;o__Oscillospirales;f__Oscillospirales;g__Hydrogenoanaerobacterium	10	5	16	14	11	19	genus	5	53.33%	36.67%	5
d__Bacteria;p__Proteobacteria;c__Gammaproteobacteria;o__Enterobacterales;f__Enterobacteriaceae;g__Raoultella	1	6	1	29	6	24	genus	-5	3.33%	20.00%	5
d__Bacteria;p__Proteobacteria;c__Gammaproteobacteria;o__Burkholderiales;f__Sutterellaceae;g__Sutterella;s__Sutterellaceae_bacterium	6	1	6	24	1	29	species	5	20.00%	3.33%	5
d__Bacteria;p__Firmicutes;c__Bacilli;o__Lactobacillales;f__Streptococcaceae;g__Streptococcus;s__Streptococcus_mutans	5	0	5	25	0	30	species	5	16.67%	0.00%	5
d__Bacteria;p__Firmicutes;c__Clostridia;o__Oscillospirales;f__Butyricicoccaceae;g__Butyricicoccus;s__bacterium_NLAE-zl-H60	5	0	5	25	0	30	species	5	16.67%	0.00%	5
d__Bacteria;p__Firmicutes;c__Clostridia;o__Lachnospirales;f__Lachnospiraceae;g__Anaerostipes;s__Anaerostipes_caccae	5	0	5	25	0	30	species	5	16.67%	0.00%	5
d__Bacteria;p__Firmicutes;c__Clostridia;o__Lachnospirales;f__Lachnospiraceae;g__Blautia;s__metagenome	4	9	12	18	17	13	species	-5	40.00%	56.67%	5
d__Bacteria;p__Firmicutes;c__Clostridia;o__Oscillospirales;f__Oscillospiraceae;g__Intestinimonas;s__Intestinimonas_sp.	10	5	16	14	11	19	species	5	53.33%	36.67%	5
d__Bacteria;p__Firmicutes;c__Bacilli;o__Erysipelotrichales;f__Erysipelotrichaceae;g__Holdemania;s__Holdemania_massiliensis	8	3	8	22	3	27	species	5	26.67%	10.00%	5
d__Bacteria;p__Bacteroidota;c__Bacteroidia;o__Bacteroidales;f__Bacteroidaceae;g__Bacteroides;s__Bacteroides_vulgatus	8	3	27	3	22	8	species	5	90.00%	73.33%	5
d__Bacteria;p__Firmicutes;c__Clostridia;o__Oscillospirales;f__UCG-010;g__UCG-010;s__gut_metagenome	8	3	15	15	10	20	species	5	50.00%	33.33%	5
d__Bacteria;p__Firmicutes;c__Clostridia;o__Peptostreptococcales-Tissierellales;f__Peptostreptococcaceae;g__Intestinibacter;s__Intestinibacter_bartlettii	8	3	14	16	9	21	species	5	46.67%	30.00%	5
d__Bacteria;p__Firmicutes;c__Clostridia;o__Lachnospirales;f__Lachnospiraceae;g__Lachnoclostridium;s__[Clostridium]_glycyrrhizinilyticum	8	3	10	20	5	25	species	5	33.33%	16.67%	5
d__Bacteria;p__Proteobacteria;c__Gammaproteobacteria;o__Enterobacterales;f__Enterobacteriaceae;g__Klebsiella;s__Klebsiella_variicola	2	7	5	25	10	20	species	-5	16.67%	33.33%	5
d__Bacteria;p__Bacteroidota;c__Bacteroidia;o__Bacteroidales;f__Rikenellaceae;g__Alistipes;s__Alistipes_onderdonkii	8	3	25	5	20	10	species	5	83.33%	66.67%	5
d__Bacteria;p__Firmicutes;c__Clostridia;o__Lachnospirales;f__Lachnospiraceae;g__[Eubacterium]_fissicatena_group;s__Faecalicatena_contorta	6	1	7	23	2	28	species	5	23.33%	6.67%	5
d__Bacteria;p__Firmicutes;c__Clostridia;o__Oscillospirales;f__Ruminococcaceae;g__Ruminococcus;s__human_gut	4	9	10	20	15	15	species	-5	33.33%	50.00%	5
d__Bacteria;p__Firmicutes;c__Clostridia;o__Clostridiales;f__Clostridiaceae;g__Clostridium_sensu_stricto_1;s__Clostridium_perfringens	6	1	7	23	2	28	species	5	23.33%	6.67%	5
d__Bacteria;p__Proteobacteria;c__Gammaproteobacteria;o__Enterobacterales;f__Enterobacteriaceae;g__Raoultella;s__Raoultella_ornithinolytica	1	6	1	29	6	24	species	-5	3.33%	20.00%	5
d__Bacteria;p__Campilobacterota	3	7	4	26	8	22	phylum	-4	13.33%	26.67%	4
d__Bacteria;p__Firmicutes;c__Clostridia;o__Clostridia_UCG-014	8	4	20	10	16	14	order	4	66.67%	53.33%	4
d__Bacteria;p__Firmicutes;c__Bacilli;o__Staphylococcales	10	6	15	15	11	19	order	4	50.00%	36.67%	4
d__Bacteria;p__Firmicutes;c__Bacilli;o__Izemoplasmatales	7	3	9	21	5	25	order	4	30.00%	16.67%	4
d__Bacteria;p__Actinobacteriota;c__Coriobacteriia;o__Coriobacteriales;f__Atopobiaceae	8	4	17	13	13	17	family	4	56.67%	43.33%	4

d__Bacteria;p__Proteobacteria;c__Gammaproteobacteria;o__Burkholderiales;f__Comamonadaceae	1	5	1	29	5	25	family	-4	3.33%	16.67%	4
d__Bacteria;p__Firmicutes;c__Clostridia;o__Lachnospirales;f__Defluviitaleaceae	7	3	22	8	18	12	family	4	73.33%	60.00%	4
d__Bacteria;p__Proteobacteria;c__Gammaproteobacteria;o__Enterobacterales;f__Erwiniaceae	1	5	4	26	8	22	family	-4	13.33%	26.67%	4
d__Bacteria;p__Firmicutes;c__Bacilli;o__Izemoplasmatales;f__Izemoplasmatales	7	3	9	21	5	25	family	4	30.00%	16.67%	4
d__Bacteria;p__Firmicutes;c__Clostridia;o__Clostridia_UCG-014;f__Clostridia_UCG-014	8	4	20	10	16	14	family	4	66.67%	53.33%	4
d__Bacteria;p__Bacteroidota;c__Bacteroidia;o__Bacteroidales;f__Barnesiellaceae;g__Coprobacter	7	11	12	18	16	14	genus	-4	40.00%	53.33%	4
d__Bacteria;p__Firmicutes;c__Clostridia;o__Lachnospirales;f__Lachnospiraceae;g__Lachnospiraceae	10	6	15	15	11	19	genus	4	50.00%	36.67%	4
d__Bacteria;p__Actinobacteriota;c__Actinobacteria;o__Actinomycetales;f__Actinomycetaceae;g__Actinomyces	8	4	23	7	19	11	genus	4	76.67%	63.33%	4
d__Bacteria;p__Actinobacteriota;c__Coriobacteriia;o__Coriobacteriales;f__Coriobacteriaceae;g__Enorma	1	5	1	29	5	25	genus	-4	3.33%	16.67%	4
d__Bacteria;p__Firmicutes;c__Clostridia;o__Peptostreptococcales-Tissierellales;f__Anaerovoracaceae;g__S5-A14a	5	1	5	25	1	29	genus	4	16.67%	3.33%	4
d__Bacteria;p__Proteobacteria;c__Gammaproteobacteria;o__Enterobacterales;f__Erwiniaceae;g__Pantoea	1	5	4	26	8	22	genus	-4	13.33%	26.67%	4
d__Bacteria;p__Firmicutes;c__Clostridia;o__Oscillospirales;f__Ruminococcaceae;g__Candidatus_Soleaferrea	9	5	24	6	20	10	genus	4	80.00%	66.67%	4
d__Bacteria;p__Firmicutes;c__Bacilli;o__Erysipelotrichales;f__Erysipelotrichaceae;g__Solobacterium	5	1	6	24	2	28	genus	4	20.00%	6.67%	4
d__Bacteria;p__Proteobacteria;c__Gammaproteobacteria;o__Enterobacterales;f__Enterobacteriaceae;g__Citrobacter	6	10	7	23	11	19	genus	-4	23.33%	36.67%	4
d__Bacteria;p__Firmicutes;c__Bacilli;o__Izemoplasmatales;f__Izemoplasmatales;g__Izemoplasmatales	7	3	9	21	5	25	genus	4	30.00%	16.67%	4
d__Bacteria;p__Firmicutes;c__Clostridia;o__Clostridia_UCG-014;f__Clostridia_UCG-014;g__Clostridia_UCG-014	8	4	20	10	16	14	genus	4	66.67%	53.33%	4
d__Bacteria;p__Firmicutes;c__Clostridia;o__Lachnospirales;f__Lachnospiraceae;g__Frisingicoccus	8	4	9	21	5	25	genus	4	30.00%	16.67%	4
d__Bacteria;p__Firmicutes;c__Clostridia;o__Oscillospirales;f__[Clostridium]_methylpentosum_group;g__[Clostridium]_methylpentosum_group	9	5	12	18	8	22	genus	4	40.00%	26.67%	4
d__Bacteria;p__Firmicutes;c__Clostridia;o__Lachnospirales;f__Lachnospiraceae;g__Marvinbryantia	3	7	20	10	24	6	genus	-4	66.67%	80.00%	4
d__Bacteria;p__Firmicutes;c__Bacilli;o__Erysipelotrichales;f__Erysipelatoclostridiaceae;g__Catenibacterium	2	6	2	28	6	24	genus	-4	6.67%	20.00%	4
d__Bacteria;p__Firmicutes;c__Clostridia;o__Lachnospirales;f__Lachnospiraceae;g__UC5-1-2E3	2	6	3	27	7	23	genus	-4	10.00%	23.33%	4
d__Bacteria;p__Firmicutes;c__Clostridia;o__Christensenellales;f__Christensenellaceae;g__Christensenella;s__Christensenella_massiliensis	0	4	0	30	4	26	species	-4	0.00%	13.33%	4
d__Bacteria;p__Synergistota;c__Synergistia;o__Synergistales;f__Synergistaceae;g__Cloacibacillus;s__Cloacibacillus_evryensis	0	4	0	30	4	26	species	-4	0.00%	13.33%	4
d__Bacteria;p__Bacteroidota;c__Bacteroidia;o__Bacteroidales;f__Bacteroidaceae;g__Bacteroides;s__Bacteroides_thetaiotaomicron	0	4	26	4	30	0	species	-4	86.67%	100.0%	4
d__Bacteria;p__Firmicutes;c__Bacilli;o__Erysipelotrichales;f__Erysipelotrichaceae;g__Turicibacter;s__Turicibacter_sp.	9	5	17	13	13	17	species	4	56.67%	43.33%	4
d__Bacteria;p__Firmicutes;c__Bacilli;o__Staphylococcales;f__Staphylococcaceae;g__Staphylococcus;s__Staphylococcus_aureus	5	1	5	25	1	29	species	4	16.67%	3.33%	4
d__Bacteria;p__Firmicutes;c__Clostridia;o__Clostridiales;f__Clostridiaceae;g__Clostridium_sensu_stricto_1;s__Clostridium_sp.	4	0	4	26	0	30	species	4	13.33%	0.00%	4
d__Bacteria;p__Firmicutes;c__Clostridia;o__Peptostreptococcales-Tissierellales;f__Anaerovoracaceae;g__Family_XIII_AD3011_group;s__metagenome	1	5	1	29	5	25	species	-4	3.33%	16.67%	4
d__Bacteria;p__Bacteroidota;c__Bacteroidia;o__Bacteroidales;f__Bacteroidaceae;g__Bacteroides;s__Bacteroides_coprophilus	3	7	3	27	7	23	species	-4	10.00%	23.33%	4
d__Bacteria;p__Firmicutes;c__Clostridia;o__Lachnospirales;f__Lachnospiraceae;g__Coprococcus;s__Clostridiaceae_bacterium	5	9	16	14	20	10	species	-4	53.33%	66.67%	4
d__Bacteria;p__Firmicutes;c__Clostridia;o__Clostridiales;f__Clostridiaceae;g__Clostridium_sensu_stricto_1;s__Clostridium_paraputrificum	6	2	7	23	3	27	species	4	23.33%	10.00%	4

d__Bacteria;p__Actinobacteriota;c__Actinobacteria;o__Bifidobacteriales;f__Bifidobacteriaceae;g__Bifidobacterium;s__Bifidobacterium_adolescentis	8	4	19	11	15	15	species	4	63.33%	50.00%	4
d__Bacteria;p__Proteobacteria;c__Gammaproteobacteria;o__Enterobacterales;f__Enterobacteriaceae;g__Citrobacter;s__Citrobacter_freundii	6	10	7	23	11	19	species	-4	23.33%	36.67%	4
d__Bacteria;p__Proteobacteria;c__Gammaproteobacteria;o__Enterobacterales;f__Enterobacteriaceae;g__Citrobacter;s__Enterobacter_sp.	1	5	1	29	5	25	species	-4	3.33%	16.67%	4
d__Bacteria;p__Firmicutes;c__Clostridia;o__Lachnospirales;f__Lachnospiraceae;g__Tyzzerella;s__unidentified	4	8	5	25	9	21	species	-4	16.67%	30.00%	4
d__Bacteria;p__Firmicutes;c__Clostridia;o__Oscillospirales;f__Ruminococcaceae;g__Incertae_Sedis;s__[Clostridium]_leptum	5	9	20	10	24	6	species	-4	66.67%	80.00%	4
d__Bacteria;p__Firmicutes;c__Bacilli;o__Lactobacillales;f__Streptococcaceae;g__Streptococcus;s__Streptococcus_salivarius	7	3	26	4	22	8	species	4	86.67%	73.33%	4
d__Bacteria;p__Firmicutes;c__Clostridia;o__Lachnospirales;f__Lachnospiraceae;g__Lachnoclostridium;s__Lachnoclostridium_urinimassiliense	0	4	0	30	4	26	species	-4	0.00%	13.33%	4
d__Bacteria;p__Proteobacteria;c__Gammaproteobacteria;o__Enterobacterales;f__Enterobacteriaceae;g__Escherichia-Shigella;s__Shigella_boydii	8	4	23	7	19	11	species	4	76.67%	63.33%	4
d__Bacteria;p__Verrucomicrobiota	7	4	23	7	20	10	phylum	3	76.67%	66.67%	3
d__Bacteria;p__Synergistota	8	5	8	22	5	25	phylum	3	26.67%	16.67%	3
d__Archaea;p__Euryarchaeota	9	6	13	17	10	20	phylum	3	43.33%	33.33%	3
d__Bacteria;p__Campilobacterota;c__Campylobacteria	4	7	5	25	8	22	class	-3	16.67%	26.67%	3
d__Bacteria;p__Synergistota;c__Synergistia	8	5	8	22	5	25	class	3	26.67%	16.67%	3
d__Archaea;p__Euryarchaeota;c__Methanobacteria	9	6	13	17	10	20	class	3	43.33%	33.33%	3
d__Bacteria;p__Firmicutes;c__Bacilli;o__Bacillales	4	1	5	25	2	28	order	3	16.67%	6.67%	3
d__Bacteria;p__Firmicutes;c__Clostridia;o__Clostridiales	2	5	25	5	28	2	order	-3	83.33%	93.33%	3
d__Bacteria;p__Firmicutes;c__Clostridia;o__Clostridia	6	3	10	20	7	23	order	3	33.33%	23.33%	3
d__Archaea;p__Euryarchaeota;c__Methanobacteria;o__Methanobacteriales	9	6	13	17	10	20	order	3	43.33%	33.33%	3
d__Bacteria;p__Synergistota;c__Synergistia;o__Synergistales	8	5	8	22	5	25	order	3	26.67%	16.67%	3
d__Bacteria;p__Firmicutes;c__Clostridia;o__Monoglobales	3	0	30	0	27	3	order	3	100.0%	90.00%	3
d__Bacteria;p__Firmicutes;c__Clostridia;o__Monoglobales;f__Monoglobaceae	3	0	30	0	27	3	family	3	100.0%	90.00%	3
d__Bacteria;p__Firmicutes;c__Clostridia;o__Clostridiales;f__Clostridiaceae	2	5	25	5	28	2	family	-3	83.33%	93.33%	3
d__Bacteria;p__Bacteroidota;c__Bacteroidia;o__Bacteroidales;f__Prevotellaceae	6	3	23	7	20	10	family	3	76.67%	66.67%	3
d__Bacteria;p__Campilobacterota;c__Campylobacteria;o__Campylobacterales;f__Campylobacteraceae	3	6	4	26	7	23	family	-3	13.33%	23.33%	3
d__Bacteria;p__Proteobacteria;c__Gammaproteobacteria;o__Burkholderiales;f__Oxalobacteraceae	8	5	13	17	10	20	family	3	43.33%	33.33%	3
d__Bacteria;p__Firmicutes;c__Clostridia;o__Oscillospirales;f__Oscillospirales	9	6	16	14	13	17	family	3	53.33%	43.33%	3
d__Bacteria;p__Firmicutes;c__Clostridia;o__Clostridia;f__Hungateiclostridiaceae	5	2	9	21	6	24	family	3	30.00%	20.00%	3
d__Bacteria;p__Verrucomicrobiota;c__Lentisphaeria;o__Victivallales;f__Victivallaceae	9	6	17	13	14	16	family	3	56.67%	46.67%	3
d__Bacteria;p__Proteobacteria;c__Gammaproteobacteria;o__Burkholderiales;f__Neisseriaceae	3	0	3	27	0	30	family	3	10.00%	0.00%	3
d__Bacteria;p__Firmicutes;c__Clostridia;o__Oscillospirales;f__Ethanoligenenaceae	5	8	5	25	8	22	family	-3	16.67%	26.67%	3
d__Bacteria;p__Synergistota;c__Synergistia;o__Synergistales;f__Synergistaceae	8	5	8	22	5	25	family	3	26.67%	16.67%	3

d__Bacteria;p__Actinobacteriota;c__Actinobacteria;o__Corynebacteriales;f__Corynebacteriaceae;g__Lawsonella	5	2	5	25	2	28	genus	3	16.67%	6.67%	3
d__Bacteria;p__Bacteroidota;c__Bacteroidia;o__Bacteroidales;f__Marinifilaceae;g__Butyricimonas	6	9	17	13	20	10	genus	-3	56.67%	66.67%	3
d__Bacteria;p__Bacteroidota;c__Bacteroidia;o__Bacteroidales;f__Marinifilaceae;g__Odoribacter	1	4	26	4	29	1	genus	-3	86.67%	96.67%	3
d__Bacteria;p__Bacteroidota;c__Bacteroidia;o__Bacteroidales;f__Marinifilaceae;g__Sanguibacteroides	6	3	6	24	3	27	genus	3	20.00%	10.00%	3
d__Bacteria;p__Firmicutes;c__Clostridia;o__Lachnospirales;f__Lachnospiraceae;g__Tyzzerella	5	8	11	19	14	16	genus	-3	36.67%	46.67%	3
d__Bacteria;p__Firmicutes;c__Clostridia;o__Oscillospirales;f__Ruminococcaceae;g__Harryflintia	1	4	2	28	5	25	genus	-3	6.67%	16.67%	3
d__Bacteria;p__Firmicutes;c__Bacilli;o__Bacillales;f__Bacillaceae;g__Bacillus	4	1	4	26	1	29	genus	3	13.33%	3.33%	3
d__Bacteria;p__Campilobacterota;c__Campylobacteria;o__Campylobacterales;f__Campylobacteraceae;g__Campylobacter	3	6	4	26	7	23	genus	-3	13.33%	23.33%	3
d__Bacteria;p__Firmicutes;c__Negativicutes;o__Veillonellales-Selenomonadales;f__Veillonellaceae;g__Veillonella	10	7	19	11	16	14	genus	3	63.33%	53.33%	3
d__Bacteria;p__Firmicutes;c__Clostridia;o__Lachnospirales;f__Lachnospiraceae;g__Sellimonas	9	6	15	15	12	18	genus	3	50.00%	40.00%	3
d__Bacteria;p__Firmicutes;c__Clostridia;o__Lachnospirales;f__Lachnospiraceae;g__Lachnospiraceae_UCG-006	5	2	5	25	2	28	genus	3	16.67%	6.67%	3
d__Bacteria;p__Synergistota;c__Synergistia;o__Synergistales;f__Synergistaceae;g__Cloacibacillus	1	4	1	29	4	26	genus	-3	3.33%	13.33%	3
d__Bacteria;p__Firmicutes;c__Clostridia;o__Oscillospirales;f__Ruminococcaceae;g__Anaerotruncus	6	3	25	5	22	8	genus	3	83.33%	73.33%	3
d__Bacteria;p__Firmicutes;c__Bacilli;o__Erysipelotrichales;f__Erysipelotrichaceae;g__Faecalitalea	6	3	9	21	6	24	genus	3	30.00%	20.00%	3
d__Bacteria;p__Firmicutes;c__Clostridia;o__Lachnospirales;f__Lachnospiraceae;g__Oribacterium	4	1	4	26	1	29	genus	3	13.33%	3.33%	3
d__Archaea;p__Euryarchaeota;c__Methanobacteria;o__Methanobacteriales;f__Methanobacteriaceae;g__Methanobrevibacter	10	7	13	17	10	20	genus	3	43.33%	33.33%	3
d__Bacteria;p__Firmicutes;c__Clostridia;o__Christensenellales;f__Christensenellaceae;g__Christensenella	3	6	3	27	6	24	genus	-3	10.00%	20.00%	3
d__Bacteria;p__Firmicutes;c__Bacilli;o__Erysipelotrichales;f__Erysipelotrichaceae;g__Erysipelotrichaceae	4	1	4	26	1	29	genus	3	13.33%	3.33%	3
d__Bacteria;p__Proteobacteria;c__Gammaproteobacteria;o__Burkholderiales;f__Comamonadaceae;g__Comamonas	1	4	1	29	4	26	genus	-3	3.33%	13.33%	3
d__Bacteria;p__Firmicutes;c__Clostridia;o__Lachnospirales;f__Lachnospiraceae;g__Lachnospiraceae_UCG-010	9	6	21	9	18	12	genus	3	70.00%	60.00%	3
d__Bacteria;p__Bacteroidota;c__Bacteroidia;o__Bacteroidales;f__Prevotellaceae;g__Alloprevotella	4	1	5	25	2	28	genus	3	16.67%	6.67%	3
d__Bacteria;p__Firmicutes;c__Clostridia;o__Oscillospirales;f__Ruminococcaceae;g__Ruminococcaceae	7	4	7	23	4	26	genus	3	23.33%	13.33%	3
d__Bacteria;p__Firmicutes;c__Clostridia;o__Oscillospirales;f__Ruminococcaceae;g__UBA1819	6	3	26	4	23	7	genus	3	86.67%	76.67%	3
d__Bacteria;p__Actinobacteriota;c__Coriobacteriia;o__Coriobacteriales;f__Atopobiaceae;g__Atopobium	5	2	5	25	2	28	genus	3	16.67%	6.67%	3
d__Bacteria;p__Firmicutes;c__Clostridia;o__Lachnospirales;f__Lachnospiraceae;g__GCA-900066755	7	4	7	23	4	26	genus	3	23.33%	13.33%	3
d__Bacteria;p__Firmicutes;c__Clostridia;o__Peptostreptococcales-Tissierellales;f__Peptostreptococcaceae;g__Romboutsia	1	4	26	4	29	1	genus	-3	86.67%	96.67%	3
d__Bacteria;p__Firmicutes;c__Clostridia;o__Peptostreptococcales-Tissierellales;f__Peptostreptococcaceae;g__Peptostreptococcus	3	6	3	27	6	24	genus	-3	10.00%	20.00%	3
d__Bacteria;p__Proteobacteria;c__Gammaproteobacteria;o__Burkholderiales;f__Sutterellaceae;g__Parasutterella	5	2	24	6	21	9	genus	3	80.00%	70.00%	3
d__Bacteria;p__Firmicutes;c__Negativicutes;o__Veillonellales-Selenomonadales;f__Selenomonadaceae;g__Mitsuokella	2	5	2	28	5	25	genus	-3	6.67%	16.67%	3
d__Bacteria;p__Proteobacteria;c__Gammaproteobacteria;o__Enterobacterales;f__Enterobacteriaceae;g__Enterobacter	3	6	5	25	8	22	genus	-3	16.67%	26.67%	3
d__Bacteria;p__Firmicutes;c__Bacilli;o__Staphylococcales;f__Staphylococcaceae;g__Staphylococcus	6	3	6	24	3	27	genus	3	20.00%	10.00%	3

d__Bacteria;p__Firmicutes;c__Clostridia;o__Monoglobales;f__Monoglobaceae;g__Monoglobus	3	0	30	0	27	3	genus	3	100.0%	90.00%	3
d__Bacteria;p__Desulfobacterota;c__Desulfovibrionia;o__Desulfovibrionales;f__Desulfovibrionaceae;g__Bilophila	4	1	28	2	25	5	genus	3	93.33%	83.33%	3
d__Bacteria;p__Proteobacteria;c__Gammaproteobacteria;o__Enterobacterales;f__Enterobacteriaceae;g__Escherichia-Shigella	1	4	26	4	29	1	genus	-3	86.67%	96.67%	3
d__Bacteria;p__Firmicutes;c__Bacilli;o__Erysipelotrichales;f__Erysipelatoclostridiaceae;g__Erysipelatoclostridium	4	7	18	12	21	9	genus	-3	60.00%	70.00%	3
d__Bacteria;p__Firmicutes;c__Clostridia;o__Oscillospirales;f__Oscillospiraceae;g__Oscillospira	7	4	7	23	4	26	genus	3	23.33%	13.33%	3
d__Bacteria;p__Firmicutes;c__Clostridia;o__Lachnospirales;f__Lachnospiraceae;g__Coprococcus	4	7	23	7	26	4	genus	-3	76.67%	86.67%	3
d__Bacteria;p__Firmicutes;c__Clostridia;o__Peptostreptococcales-Tissierellales;f__Anaerovoracaceae;g__[Eubacterium]_nodatum_group;s__unidentified	3	0	3	27	0	30	species	3	10.00%	0.00%	3
d__Bacteria;p__Firmicutes;c__Clostridia;o__Peptostreptococcales-Tissierellales;f__Peptostreptococcales-Tissierellales;g__Peptoniphilus;s__Peptoniphilus_duerdenii	3	0	3	27	0	30	species	3	10.00%	0.00%	3
d__Bacteria;p__Firmicutes;c__Clostridia;o__Oscillospirales;s__metagenome	1	4	1	29	4	26	species	-3	3.33%	13.33%	3
d__Bacteria;p__Proteobacteria;c__Gammaproteobacteria;o__Burkholderiales;f__Comamonadaceae;g__Comamonas;s__Comamonas_terrae	0	3	0	30	3	27	species	-3	0.00%	10.00%	3
d__Bacteria;p__Firmicutes;c__Clostridia;o__Lachnospirales;f__Lachnospiraceae;g__Anaerostipes;s__Anaerostipes_hadrus	6	9	11	19	14	16	species	-3	36.67%	46.67%	3
d__Bacteria;p__Proteobacteria;c__Gammaproteobacteria;o__Enterobacterales;f__Enterobacteriaceae;g__Klebsiella;s__Klebsiella_pneumoniae	1	4	3	27	6	24	species	-3	10.00%	20.00%	3
d__Bacteria;p__Proteobacteria;c__Gammaproteobacteria;o__Enterobacterales;f__Enterobacteriaceae;g__Enterobacter;s__Enterobacteriaceae_bacterium	0	3	0	30	3	27	species	-3	0.00%	10.00%	3
d__Bacteria;p__Firmicutes;c__Bacilli;o__Erysipelotrichales;f__Erysipelotrichaceae;g__Erysipelotrichaceae;s__Faecalitalea_sp.	4	1	4	26	1	29	species	3	13.33%	3.33%	3
d__Bacteria;p__Firmicutes;c__Clostridia;o__Oscillospirales;f__Oscillospiraceae;g__Intestinimonas;s__Intestinimonas_butyriciproducens	8	5	10	20	7	23	species	3	33.33%	23.33%	3
d__Bacteria;p__Proteobacteria;c__Gammaproteobacteria;o__Enterobacterales;f__Enterobacteriaceae;g__Citrobacter;s__bacterium_enrichment	2	5	2	28	5	25	species	-3	6.67%	16.67%	3
d__Bacteria;p__Firmicutes;c__Clostridia;o__Oscillospirales;f__Oscillospiraceae;s__Intestinimonas_sp.	5	8	8	22	11	19	species	-3	26.67%	36.67%	3
d__Bacteria;p__Firmicutes;c__Clostridia;o__Oscillospirales;f__Ruminococcaceae;g__Faecalibacterium;s__metagenome	2	5	24	6	27	3	species	-3	80.00%	90.00%	3
d__Bacteria;p__Firmicutes;c__Clostridia;o__Oscillospirales;f__Ruminococcaceae;g__Ruminococcus;s__Ruminococcus_sp.	6	3	8	22	5	25	species	3	26.67%	16.67%	3
d__Bacteria;p__Firmicutes;c__Bacilli;o__Lactobacillales;f__Enterococcaceae;g__Enterococcus;s__Enterococcus_gilvus	0	3	0	30	3	27	species	-3	0.00%	10.00%	3
d__Bacteria;p__Bacteroidota;c__Bacteroidia;o__Bacteroidales;f__Rikenellaceae;g__Alistipes;s__Alistipes_finegoldii	10	7	14	16	11	19	species	3	46.67%	36.67%	3
d__Bacteria;p__Bacteroidota;c__Bacteroidia;o__Bacteroidales;f__Prevotellaceae;s__Prevotella_sp.	1	4	1	29	4	26	species	-3	3.33%	13.33%	3
d__Bacteria;p__Bacteroidota;c__Bacteroidia;o__Bacteroidales;f__Bacteroidaceae;g__Bacteroides;s__Bacteroides_ovatus	8	5	23	7	20	10	species	3	76.67%	66.67%	3
d__Bacteria;p__Firmicutes;c__Bacilli;o__RF39;f__RF39;g__RF39;s__gut_metagenome	4	1	4	26	1	29	species	3	13.33%	3.33%	3
d__Bacteria;p__Cyanobacteria;c__Vampirivibrionia;o__Gastranaerophilales;f__Gastranaerophilales;g__Gastranaerophilales;s__Clostridium_sp.	3	0	3	27	0	30	species	3	10.00%	0.00%	3
d__Bacteria;p__Bacteroidota;c__Bacteroidia;o__Bacteroidales;f__Rikenellaceae;g__Alistipes;s__Alistipes_inops	4	7	6	24	9	21	species	-3	20.00%	30.00%	3
d__Bacteria;p__Firmicutes;c__Bacilli;o__Lactobacillales;f__Enterococcaceae;g__Enterococcus;s__Enterococcus_durans	1	4	1	29	4	26	species	-3	3.33%	13.33%	3
d__Bacteria;p__Firmicutes;c__Bacilli;o__Erysipelotrichales;f__Erysipelatoclostridiaceae;g__Erysipelatoclostridium;s__[Clostridium]_spiroforme	7	4	10	20	7	23	species	3	33.33%	23.33%	3
d__Bacteria;p__Proteobacteria;c__Gammaproteobacteria;o__Pasteurellales;f__Pasteurellaceae;g__Haemophilus;s__Haemophilus_pittmaniae	4	1	4	26	1	29	species	3	13.33%	3.33%	3
d__Bacteria;p__Actinobacteriota;c__Actinobacteria;o__Bifidobacteriales;f__Bifidobacteriaceae;g__Bifidobacterium;s__Bifidobacterium_pseudocatenulatum	5	8	7	23	10	20	species	-3	23.33%	33.33%	3
d__Bacteria;p__Proteobacteria;c__Gammaproteobacteria;o__Burkholderiales;f__Sutterellaceae;g__Sutterella;s__Sutterella_wadsworthensis	8	5	11	19	8	22	species	3	36.67%	26.67%	3

d__Bacteria;p__Bacteroidota;c__Bacteroidia;o__Bacteroidales;f__Tannerellaceae;g__Parabacteroides;s__Parabacteroides_gordonii	1	4	1	29	4	26	species	-3	3.33%	13.33%	3
d__Bacteria;p__Firmicutes;c__Clostridia;o__Oscillospirales;f__Ruminococcaceae;g__Harryflintia;s__Harryflintia_acetispora	1	4	1	29	4	26	species	-3	3.33%	13.33%	3
d__Bacteria;p__Actinobacteriota;c__Coriobacteriia;o__Coriobacteriales;f__Eggerthellaceae;g__Gordonibacter;s__Gordonibacter_pamelaeae	8	11	11	19	14	16	species	-3	36.67%	46.67%	3
d__Bacteria;p__Firmicutes;c__Bacilli;o__Lactobacillales;f__Enterococcaceae;g__Enterococcus;s__Enterococcus_casseliflavus	0	3	0	30	3	27	species	-3	0.00%	10.00%	3
d__Bacteria;p__Bacteroidota;c__Bacteroidia;o__Bacteroidales;f__Tannerellaceae;g__Parabacteroides;s__gut_metagenome	2	5	2	28	5	25	species	-3	6.67%	16.67%	3
d__Bacteria;p__Proteobacteria;c__Gammaproteobacteria;o__Burkholderiales;f__Oxalobacteraceae;g__Oxalobacter;s__Oxalobacter_formigenes	8	5	12	18	9	21	species	3	40.00%	30.00%	3
d__Bacteria;p__Proteobacteria;c__Gammaproteobacteria;o__Enterobacterales;f__Enterobacteriaceae;g__Enterobacter;s__Klebsiella_oxytoca	0	3	1	29	4	26	species	-3	3.33%	13.33%	3
d__Bacteria;p__Firmicutes;c__Clostridia;o__Lachnospirales;f__Lachnospiraceae;g__Sellimonas;s__Lachnoclostridium_phocaeense	6	3	8	22	5	25	species	3	26.67%	16.67%	3
d__Bacteria;p__Bacteroidota;c__Bacteroidia;o__Bacteroidales;f__Tannerellaceae;g__Parabacteroides;s__Parabacteroides_distasonis	7	10	14	16	17	13	species	-3	46.67%	56.67%	3
d__Bacteria;p__Firmicutes;c__Clostridia;o__Lachnospirales;f__Lachnospiraceae;g__Catenibacillus;s__Catenibacillus_scindens	2	5	5	25	8	22	species	-3	16.67%	26.67%	3
d__Bacteria;p__Verrucomicrobiota;c__Lentisphaeria;o__Victivallales;f__Victivallaceae;g__Victivallis;s__Victivallis_vadensis	4	7	4	26	7	23	species	-3	13.33%	23.33%	3
d__Bacteria;p__Firmicutes;c__Clostridia;o__Oscillospirales;f__Ruminococcaceae;g__Anaerotruncus;s__Anaerotruncus_sp.	10	7	16	14	13	17	species	3	53.33%	43.33%	3
d__Bacteria;p__Bacteroidota;c__Bacteroidia;o__Bacteroidales;f__Marinifilaceae;g__Odoribacter;s__Odoribacter_splanchnicus	1	4	26	4	29	1	species	-3	86.67%	96.67%	3
d__Bacteria;p__Firmicutes;c__Clostridia;o__Oscillospirales;f__Ruminococcaceae;g__Phocea;s__Anaerotruncus_sp.	9	6	14	16	11	19	species	3	46.67%	36.67%	3
d__Bacteria;p__Bacteroidota;c__Bacteroidia;o__Bacteroidales;f__Marinifilaceae;g__Sanguibacteroides;s__Sanguibacteroides_justesenii	4	1	4	26	1	29	species	3	13.33%	3.33%	3
d__Bacteria;p__Bacteroidota;c__Bacteroidia;o__Bacteroidales;f__Tannerellaceae;g__Parabacteroides;s__Parabacteroides_goldsteinii	6	3	8	22	5	25	species	3	26.67%	16.67%	3
d__Bacteria;p__Firmicutes;c__Clostridia;o__Oscillospirales;f__Ruminococcaceae;g__Ruminococcus;s__Ruminococcus_bicirculans	7	10	14	16	17	13	species	-3	46.67%	56.67%	3
d__Bacteria;p__Campilobacterota;c__Campylobacteria;o__Campylobacterales;f__Campylobacteraceae;g__Campylobacter;s__Campylobacter_hominis	2	5	3	27	6	24	species	-3	10.00%	20.00%	3
d__Bacteria;p__Firmicutes;c__Clostridia;o__Lachnospirales;f__Lachnospiraceae;g__Marvinbryantia;s__metagenome	6	9	12	18	15	15	species	-3	40.00%	50.00%	3
d__Bacteria;p__Firmicutes;c__Bacilli;o__Erysipelotrichales;f__Erysipelotrichaceae;g__Dielma;s__Dielma_sp.	2	5	2	28	5	25	species	-3	6.67%	16.67%	3
d__Bacteria;p__Actinobacteriota;c__Actinobacteria;o__Corynebacteriales;f__Corynebacteriaceae;g__Corynebacterium;s__Corynebacterium_aurimucosum	4	1	4	26	1	29	species	3	13.33%	3.33%	3
d__Bacteria;p__Bacteroidota;c__Bacteroidia;o__Bacteroidales;f__Prevotellaceae;g__Prevotella;s__metagenome	6	3	6	24	3	27	species	3	20.00%	10.00%	3
d__Bacteria;p__Firmicutes;c__Clostridia;o__Christensenellales;f__Christensenellaceae;g__Christensenellaceae_R-7_group;s__metagenome	6	9	20	10	23	7	species	-3	66.67%	76.67%	3
d__Bacteria;p__Patescibacteria;c__Saccharimonadia	5	3	8	22	6	24	class	2	26.67%	20.00%	2
d__Bacteria;p__Verrucomicrobiota;c__Lentisphaeria	9	7	17	13	15	15	class	2	56.67%	50.00%	2
d__Bacteria;p__Firmicutes;c__Incertae_Sedis	8	6	8	22	6	24	class	2	26.67%	20.00%	2
d__Bacteria;p__Proteobacteria;c__Alphaproteobacteria;o__Sphingomonadales	2	4	2	28	4	26	order	-2	6.67%	13.33%	2
d__Bacteria;p__Actinobacteriota;c__Actinobacteria;o__Actinomycetales	6	4	24	6	22	8	order	2	80.00%	73.33%	2
d__Bacteria;p__Actinobacteriota;c__Actinobacteria;o__Corynebacteriales	8	6	10	20	8	22	order	2	33.33%	26.67%	2
d__Bacteria;p__Firmicutes;c__Bacilli;o__Acholeplasmatales	0	2	0	30	2	28	order	-2	0.00%	6.67%	2
d__Bacteria;p__Verrucomicrobiota;c__Lentisphaeria;o__Victivallales	9	7	17	13	15	15	order	2	56.67%	50.00%	2

d__Bacteria;p__Proteobacteria;c__Gammaproteobacteria;o__Pseudomonadales	7	9	12	18	14	16	order	-2	40.00%	46.67%	2
d__Bacteria;p__Firmicutes;c__Negativicutes;o__Veillonellales-Selenomonadales	0	2	27	3	29	1	order	-2	90.00%	96.67%	2
d__Bacteria;p__Verrucomicrobiota;c__Verrucomicrobiae;o__Opitutales	7	5	7	23	5	25	order	2	23.33%	16.67%	2
d__Bacteria;p__Firmicutes;c__Clostridia;o__Peptococcales	9	7	18	12	16	14	order	2	60.00%	53.33%	2
d__Bacteria;p__Firmicutes;c__Incertae_Sedis;o__DTU014	8	6	8	22	6	24	order	2	26.67%	20.00%	2
d__Bacteria;p__Proteobacteria;c__Gammaproteobacteria;o__Pseudomonadales;f__Moraxellaceae	4	6	5	25	7	23	family	-2	16.67%	23.33%	2
d__Bacteria;p__Firmicutes;c__Incertae_Sedis;o__DTU014;f__DTU014	8	6	8	22	6	24	family	2	26.67%	20.00%	2
d__Bacteria;p__Proteobacteria;c__Gammaproteobacteria;o__Pasteurellales;f__Pasteurellaceae	5	7	21	9	23	7	family	-2	70.00%	76.67%	2
d__Bacteria;p__Firmicutes;c__Bacilli;o__Acholeplasmatales;f__Acholeplasmataceae	0	2	0	30	2	28	family	-2	0.00%	6.67%	2
d__Bacteria;p__Actinobacteriota;c__Coriobacteriia;o__Coriobacteriales;f__Coriobacteriaceae	6	8	22	8	24	6	family	-2	73.33%	80.00%	2
d__Bacteria;p__Firmicutes;c__Clostridia;o__Peptostreptococcales-Tissierellales;f__Anaerovoracaceae	3	1	29	1	27	3	family	2	96.67%	90.00%	2
d__Bacteria;p__Actinobacteriota;c__Actinobacteria;o__Corynebacteriales;f__Corynebacteriaceae	8	6	10	20	8	22	family	2	33.33%	26.67%	2
d__Bacteria;p__Bacteroidota;c__Bacteroidia;o__Bacteroidales;f__Marinifilaceae	1	3	27	3	29	1	family	-2	90.00%	96.67%	2
d__Archaea;p__Euryarchaeota;c__Methanobacteria;o__Methanobacteriales;f__Methanobacteriaceae	9	7	13	17	11	19	family	2	43.33%	36.67%	2
d__Bacteria;p__Proteobacteria;c__Gammaproteobacteria;o__Pseudomonadales;f__Pseudomonadaceae	6	8	7	23	9	21	family	-2	23.33%	30.00%	2
d__Bacteria;p__Firmicutes;c__Bacilli;o__Bacillales;f__Bacillaceae	4	2	4	26	2	28	family	2	13.33%	6.67%	2
d__Bacteria;p__Firmicutes;c__Bacilli;o__Staphylococcales;f__Staphylococcaceae	5	3	5	25	3	27	family	2	16.67%	10.00%	2
d__Bacteria;p__Actinobacteriota;c__Actinobacteria;o__Actinomycetales;f__Actinomycetales_Incertae_Sedis	0	2	0	30	2	28	family	-2	0.00%	6.67%	2
d__Bacteria;p__Bacteroidota;c__Bacteroidia;o__Bacteroidales;f__Barnesiellaceae	5	7	22	8	24	6	family	-2	73.33%	80.00%	2
d__Bacteria;p__Proteobacteria;c__Gammaproteobacteria;o__Enterobacterales;f__Morganellaceae	1	3	2	28	4	26	family	-2	6.67%	13.33%	2
d__Bacteria;p__Firmicutes;c__Clostridia;o__Peptostreptococcales-Tissierellales;f__Peptostreptococcales-Tissierellales	7	9	14	16	16	14	family	-2	46.67%	53.33%	2
d__Bacteria;p__Patescibacteria;c__Saccharimonadia;o__Saccharimonadales;f__Saccharimonadaceae	4	2	7	23	5	25	family	2	23.33%	16.67%	2
d__Bacteria;p__Proteobacteria;c__Gammaproteobacteria;o__Burkholderiales;f__Oxalobacteraceae;g__Oxalobacter	7	5	12	18	10	20	genus	2	40.00%	33.33%	2
d__Bacteria;p__Firmicutes;c__Clostridia;o__Oscillospirales;f__Ruminococcaceae;g__Negativibacillus	4	6	23	7	25	5	genus	-2	76.67%	83.33%	2
d__Bacteria;p__Firmicutes;c__Clostridia;o__Lachnospirales;f__Lachnospiraceae;g__Cellulosilyticum	0	2	0	30	2	28	genus	-2	0.00%	6.67%	2
d__Bacteria;p__Firmicutes;c__Incertae_Sedis;o__DTU014;f__DTU014;g__DTU014	8	6	8	22	6	24	genus	2	26.67%	20.00%	2
d__Bacteria;p__Verrucomicrobiota;c__Lentisphaeria;o__Victivallales;f__Victivallaceae;g__Victivallaceae	4	2	5	25	3	27	genus	2	16.67%	10.00%	2
d__Bacteria;p__Bacteroidota;c__Bacteroidia;o__Bacteroidales;f__Prevotellaceae;g__Paraprevotella	5	7	11	19	13	17	genus	-2	36.67%	43.33%	2
d__Bacteria;p__Firmicutes;c__Clostridia;o__Oscillospirales;f__Oscillospiraceae;g__Pseudoflavonifractor	3	5	4	26	6	24	genus	-2	13.33%	20.00%	2
d__Bacteria;p__Firmicutes;c__Clostridia;o__Oscillospirales;f__UCG-011;g__UCG-011	5	3	6	24	4	26	genus	2	20.00%	13.33%	2
d__Bacteria;p__Firmicutes;c__Clostridia;o__Lachnospirales;f__Lachnospiraceae;g__Lachnospiraceae_UCG-004	3	1	27	3	25	5	genus	2	90.00%	83.33%	2

d__Bacteria;p__Firmicutes;c__Bacilli;o__Erysipelotrichales;f__Erysipelotrichaceae;g__Holdemania	1	3	27	3	29	1	genus	-2	90.00%	96.67%	2
d__Bacteria;p__Firmicutes;c__Clostridia;o__Lachnospirales;f__Lachnospiraceae;g__Hungatella	6	4	14	16	12	18	genus	2	46.67%	40.00%	2
d__Bacteria;p__Firmicutes;c__Clostridia;o__Clostridiales;f__Clostridiaceae;g__Sarcina	2	0	2	28	0	30	genus	2	6.67%	0.00%	2
d__Bacteria;p__Proteobacteria;c__Gammaproteobacteria;o__Enterobacterales;f__Yersiniaceae;g__Serratia	5	3	5	25	3	27	genus	2	16.67%	10.00%	2
d__Bacteria;p__Firmicutes;c__Clostridia;o__Peptostreptococcales-Tissierellales;f__Peptostreptococcales-Tissierellales;g__Gallicola	2	0	2	28	0	30	genus	2	6.67%	0.00%	2
d__Bacteria;p__Firmicutes;c__Bacilli;o__Acholeplasmatales;f__Acholeplasmataceae;g__Anaeroplasma	0	2	0	30	2	28	genus	-2	0.00%	6.67%	2
d__Bacteria;p__Verrucomicrobiota;c__Lentisphaeria;o__Victivallales;f__vadinBE97;g__vadinBE97	6	4	6	24	4	26	genus	2	20.00%	13.33%	2
d__Bacteria;p__Firmicutes;c__Clostridia;o__Peptostreptococcales-Tissierellales;f__Anaerovoracaceae;g__[Eubacterium]_nodatum_group	9	7	10	20	8	22	genus	2	33.33%	26.67%	2
d__Bacteria;p__Firmicutes;c__Clostridia;o__Peptostreptococcales-Tissierellales;f__Peptostreptococcaceae;g__Terrisporobacter	6	8	16	14	18	12	genus	-2	53.33%	60.00%	2
d__Bacteria;p__Firmicutes;c__Clostridia;o__Oscillospirales;f__Ethanoligenenaceae;g__Acetanaerobacterium	3	5	3	27	5	25	genus	-2	10.00%	16.67%	2
d__Bacteria;p__Firmicutes;c__Clostridia;o__Clostridiales;f__Clostridiaceae;g__Clostridium_sensu_stricto_1	3	5	25	5	27	3	genus	-2	83.33%	90.00%	2
d__Bacteria;p__Bacteroidota;c__Bacteroidia;o__Bacteroidales;f__Prevotellaceae;g__Prevotellaceae_UCG-001	2	4	2	28	4	26	genus	-2	6.67%	13.33%	2
d__Bacteria;p__Firmicutes;c__Bacilli;o__Erysipelotrichales;f__Erysipelotrichaceae;g__Holdemanella	5	3	7	23	5	25	genus	2	23.33%	16.67%	2
d__Bacteria;p__Proteobacteria;c__Gammaproteobacteria;o__Enterobacterales;f__Enterobacteriaceae;g__Salmonella	10	8	15	15	13	17	genus	2	50.00%	43.33%	2
d__Bacteria;p__Firmicutes;c__Bacilli;o__Erysipelotrichales;f__Erysipelotrichaceae;g__Merdibacter	8	6	13	17	11	19	genus	2	43.33%	36.67%	2
d__Bacteria;p__Actinobacteriota;c__Coriobacteriia;o__Coriobacteriales;f__Eggerthellaceae;g__Eggerthella	7	9	18	12	20	10	genus	-2	60.00%	66.67%	2
d__Bacteria;p__Actinobacteriota;c__Coriobacteriia;o__Coriobacteriales;f__Eggerthellaceae;g__Gordonibacter	8	10	14	16	16	14	genus	-2	46.67%	53.33%	2
d__Bacteria;p__Patescibacteria;c__Saccharimonadia;o__Saccharimonadales;f__Saccharimonadaceae;g__TM7x	4	2	6	24	4	26	genus	2	20.00%	13.33%	2
d__Bacteria;p__Firmicutes;c__Clostridia;o__Oscillospirales;f__Ruminococcaceae;g__CAG-352	8	6	11	19	9	21	genus	2	36.67%	30.00%	2
d__Bacteria;p__Firmicutes;c__Clostridia;o__Oscillospirales;f__Oscillospiraceae;g__UCG-003	5	7	19	11	21	9	genus	-2	63.33%	70.00%	2
d__Bacteria;p__Firmicutes;c__Clostridia;o__Oscillospirales;f__[Eubacterium]_coprostanoligenes_group;g__[Eubacterium]_coprostanoligenes_group	2	4	26	4	28	2	genus	-2	86.67%	93.33%	2
d__Bacteria;p__Bacteroidota;c__Bacteroidia;o__Bacteroidales;f__Barnesiellaceae;g__Barnesiella	8	6	21	9	19	11	genus	2	70.00%	63.33%	2
d__Bacteria;p__Firmicutes;c__Clostridia;o__Lachnospirales;f__Lachnospiraceae;g__[Ruminococcus]_gauvreauii_group	6	4	24	6	22	8	genus	2	80.00%	73.33%	2
d__Bacteria;p__Firmicutes;c__Negativicutes;o__Veillonellales-Selenomonadales;f__Veillonellaceae;g__Megasphaera	4	2	4	26	2	28	genus	2	13.33%	6.67%	2
d__Bacteria;p__Firmicutes;c__Clostridia;o__Lachnospirales;f__Lachnospiraceae;g__Lachnospiraceae_NC2004_group	9	7	15	15	13	17	genus	2	50.00%	43.33%	2
d__Bacteria;p__Actinobacteriota;c__Coriobacteriia;o__Coriobacteriales;f__Eggerthellaceae;g__Slackia	5	7	7	23	9	21	genus	-2	23.33%	30.00%	2
d__Bacteria;p__Firmicutes;c__Clostridia;o__Lachnospirales;f__Lachnospiraceae;g__Lactonifactor	3	1	3	27	1	29	genus	2	10.00%	3.33%	2
d__Bacteria;p__Firmicutes;c__Clostridia;o__Lachnospirales;f__Lachnospiraceae;g__Lachnospira	1	3	26	4	28	2	genus	-2	86.67%	93.33%	2
d__Bacteria;p__Firmicutes;c__Clostridia;o__Oscillospirales;f__Butyricicoccaceae;g__UCG-009	9	7	16	14	14	16	genus	2	53.33%	46.67%	2
d__Bacteria;p__Proteobacteria;c__Gammaproteobacteria;o__Pseudomonadales;f__Moraxellaceae;g__Acinetobacter	4	6	5	25	7	23	genus	-2	16.67%	23.33%	2
d__Bacteria;p__Firmicutes;c__Bacilli;o__Erysipelotrichales;f__Erysipelotrichaceae;g__Dielma	7	9	8	22	10	20	genus	-2	26.67%	33.33%	2

d__Bacteria;p__Firmicutes;c__Clostridia;o__Lachnospirales;f__Lachnospiraceae;g__Catenibacillus	3	5	6	24	8	22	genus	-2	20.00%	26.67%	2
d__Bacteria;p__Firmicutes;c__Bacilli;o__Lactobacillales;f__Streptococcaceae;g__Lactococcus	1	3	2	28	4	26	genus	-2	6.67%	13.33%	2
d__Bacteria;p__Firmicutes;c__Clostridia;o__Oscillospirales;f__Ruminococcaceae;g__Paludicola	2	4	5	25	7	23	genus	-2	16.67%	23.33%	2
d__Bacteria;p__Actinobacteriota;c__Actinobacteria;o__Bifidobacteriales;f__Bifidobacteriaceae;g__Alloscardovia	0	2	0	30	2	28	genus	-2	0.00%	6.67%	2
d__Bacteria;p__Actinobacteriota;c__Coriobacteriia;o__Coriobacteriales;f__Eggerthellaceae;g__CHKCI002	2	0	2	28	0	30	genus	2	6.67%	0.00%	2
d__Bacteria;p__Firmicutes;c__Clostridia;o__Peptostreptococcales-Tissierellales;f__Peptostreptococcales-Tissierellales;g__Fenollaria	5	3	5	25	3	27	genus	2	16.67%	10.00%	2
d__Bacteria;p__Actinobacteriota;c__Actinobacteria;o__Bifidobacteriales;f__Bifidobacteriaceae;g__Gardnerella	0	2	0	30	2	28	genus	-2	0.00%	6.67%	2
d__Bacteria;p__Firmicutes;c__Negativicutes;o__Acidaminococcales;f__Acidaminococcaceae;g__Acidaminococcus	7	5	8	22	6	24	genus	2	26.67%	20.00%	2
d__Bacteria;p__Proteobacteria;c__Gammaproteobacteria;o__Burkholderiales;f__Comamonadaceae;g__Extensimonas	0	2	0	30	2	28	genus	-2	0.00%	6.67%	2
d__Bacteria;p__Proteobacteria;c__Gammaproteobacteria;o__Enterobacterales;f__Morganellaceae;g__Providencia	0	2	0	30	2	28	genus	-2	0.00%	6.67%	2
d__Bacteria;p__Firmicutes;c__Clostridia;o__Oscillospirales;f__Oscillospiraceae;g__UCG-005	1	3	27	3	29	1	genus	-2	90.00%	96.67%	2
d__Bacteria;p__Firmicutes;c__Bacilli;o__Erysipelotrichales;f__Erysipelatoclostridiaceae;g__Asteroleplasma	4	2	4	26	2	28	genus	2	13.33%	6.67%	2
d__Bacteria;p__Firmicutes;c__Clostridia;o__Oscillospirales;f__Butyricicoccaceae;g__Butyricicoccus	0	2	28	2	30	0	genus	-2	93.33%	100.0%	2
d__Bacteria;p__Firmicutes;c__Clostridia;o__Lachnospirales;f__Lachnospiraceae;g__Anaerosporobacter	7	5	7	23	5	25	genus	2	23.33%	16.67%	2
d__Bacteria;p__Firmicutes;c__Clostridia;o__Clostridia_vadinBB60_group;f__Clostridia_vadinBB60_group;g__Clostridia_vadinBB60_group	6	4	25	5	23	7	genus	2	83.33%	76.67%	2
d__Bacteria;p__Proteobacteria;c__Gammaproteobacteria;o__Enterobacterales;f__Enterobacteriaceae;g__Escherichia-Shigella;s__Escherichia_coli	5	7	23	7	25	5	species	-2	76.67%	83.33%	2
d__Bacteria;p__Bacteroidota;c__Bacteroidia;o__Bacteroidales;f__Rikenellaceae;g__Alistipes;s__gut_metagenome	3	5	3	27	5	25	species	-2	10.00%	16.67%	2
d__Bacteria;p__Proteobacteria;c__Gammaproteobacteria;o__Enterobacterales;f__Yersiniaceae;g__Yersinia;s__Yersinia_ruckeri	1	3	1	29	3	27	species	-2	3.33%	10.00%	2
d__Bacteria;p__Proteobacteria;c__Gammaproteobacteria;o__Burkholderiales;f__Comamonadaceae;g__Comamonas;s__Comamonas_aquatica	0	2	0	30	2	28	species	-2	0.00%	6.67%	2
d__Bacteria;p__Firmicutes;c__Clostridia;o__Peptostreptococcales-Tissierellales;f__Peptostreptococcales-Tissierellales;g__Peptoniphilus;s__Peptoniphilus_coxii	0	2	0	30	2	28	species	-2	0.00%	6.67%	2
d__Bacteria;p__Bacteroidota;c__Bacteroidia;o__Bacteroidales;f__Tannerellaceae;g__Parabacteroides;s__Parabacteroides_sp.	8	6	10	20	8	22	species	2	33.33%	26.67%	2
d__Bacteria;p__Firmicutes;c__Clostridia;o__Oscillospirales;f__[Clostridium]_methylpentosum_group;g__[Clostridium]_methylpentosum_group;s__[Clostridium]_methylpentosum	2	0	2	28	0	30	species	2	6.67%	0.00%	2
d__Bacteria;p__Proteobacteria;c__Gammaproteobacteria;o__Enterobacterales;f__Morganellaceae;g__Morganella;s__Morganella_morganii	0	2	0	30	2	28	species	-2	0.00%	6.67%	2
d__Bacteria;p__Firmicutes;c__Clostridia;o__Lachnospirales;f__Lachnospiraceae;g__[Eubacterium]_hallii_group;s__[Eubacterium]_hallii	6	8	20	10	22	8	species	-2	66.67%	73.33%	2
d__Bacteria;p__Firmicutes;c__Clostridia;o__Oscillospirales;f__Oscillospiraceae;g__Colidextribacter;s__Colidextribacter_massiliensis	1	3	1	29	3	27	species	-2	3.33%	10.00%	2
d__Bacteria;p__Proteobacteria;c__Gammaproteobacteria;o__Enterobacterales;f__Enterobacteriaceae;g__Raoultella;s__bacterium_E2-20	0	2	0	30	2	28	species	-2	0.00%	6.67%	2
d__Bacteria;p__Actinobacteriota;c__Actinobacteria;o__Bifidobacteriales;f__Bifidobacteriaceae;g__Bifidobacterium;s__Bifidobacterium_catenulatum	5	7	7	23	9	21	species	-2	23.33%	30.00%	2
d__Bacteria;p__Bacteroidota;c__Bacteroidia;o__Bacteroidales;f__Bacteroidaceae;g__Bacteroides;s__Bacteroides_kribbi	7	5	17	13	15	15	species	2	56.67%	50.00%	2
d__Bacteria;p__Firmicutes;c__Bacilli;o__Lactobacillales;f__Leuconostocaceae;g__Leuconostoc;s__Leuconostoc_mesenteroides	0	2	0	30	2	28	species	-2	0.00%	6.67%	2
d__Bacteria;p__Firmicutes;c__Clostridia;o__Lachnospirales;f__Lachnospiraceae;g__Lachnospiraceae_UCG-010;s__metagenome	3	1	3	27	1	29	species	2	10.00%	3.33%	2
d__Bacteria;p__Firmicutes;c__Clostridia;o__Oscillospirales;f__Oscillospirales;g__Hydrogenoanaerobacterium;s__Selenomonadales_bacterium	7	5	13	17	11	19	species	2	43.33%	36.67%	2

d__Bacteria;p__Proteobacteria;c__Gammaproteobacteria;o__Burkholderiales;f__Neisseriaceae;s__unidentified_marine	2	0	2	28	0	30	species	2	6.67%	0.00%	2
d__Bacteria;p__Firmicutes;c__Clostridia;o__Lachnospirales;f__Lachnospiraceae;g__Blautia;s__Blautia_hydrogenotrophica	4	2	4	26	2	28	species	2	13.33%	6.67%	2
d__Bacteria;p__Bacteroidota;c__Bacteroidia;o__Bacteroidales;f__Bacteroidaceae;g__Bacteroides;s__Bacteroides_stercoris	11	9	20	10	18	12	species	2	66.67%	60.00%	2
d__Bacteria;p__Proteobacteria;c__Gammaproteobacteria;o__Pseudomonadales;f__Pseudomonadaceae;g__Pseudomonas;s__Pseudomonas_plecoglossicida	0	2	0	30	2	28	species	-2	0.00%	6.67%	2
d__Bacteria;p__Firmicutes;c__Clostridia;o__Oscillospirales;f__Ruminococcaceae;g__Candidatus_Soleaferrea;s__Ruminococcaceae_bacterium	9	7	12	18	10	20	species	2	40.00%	33.33%	2
d__Bacteria;p__Firmicutes;c__Bacilli;o__Erysipelotrichales;f__Erysipelatoclostridiaceae;g__Erysipelatoclostridium;s__bacterium_NLAE-zl-C597	2	0	2	28	0	30	species	2	6.67%	0.00%	2
d__Bacteria;p__Bacteroidota;c__Bacteroidia;o__Bacteroidales;f__Bacteroidaceae;g__Bacteroides;s__Bacteroides_xylanisolvens	5	7	20	10	22	8	species	-2	66.67%	73.33%	2
d__Bacteria;p__Desulfobacterota;c__Desulfovibrionia;o__Desulfovibrionales;f__Desulfovibrionaceae;g__Desulfovibrio;s__metagenome	1	3	1	29	3	27	species	-2	3.33%	10.00%	2
d__Bacteria;p__Bacteroidota;c__Bacteroidia;o__Bacteroidales;f__Bacteroidaceae;g__Bacteroides;s__Bacteroides_caccae	4	6	23	7	25	5	species	-2	76.67%	83.33%	2
d__Bacteria;p__Proteobacteria;c__Gammaproteobacteria;o__Enterobacterales;f__Enterobacteriaceae;g__Salmonella;s__Salmonella_enterica	10	8	15	15	13	17	species	2	50.00%	43.33%	2
d__Bacteria;p__Firmicutes;c__Clostridia;o__Oscillospirales;f__Ruminococcaceae;g__Subdoligranulum;s__Subdoligranulum_variabile	2	0	2	28	0	30	species	2	6.67%	0.00%	2
d__Bacteria;p__Firmicutes;c__Clostridia;o__Oscillospirales;f__Oscillospiraceae;g__Oscillibacter;s__metagenome	4	2	5	25	3	27	species	2	16.67%	10.00%	2
d__Bacteria;p__Firmicutes;c__Bacilli;o__Lactobacillales;f__Streptococcaceae;g__Streptococcus;s__Streptococcus_mitis	5	3	5	25	3	27	species	2	16.67%	10.00%	2
d__Bacteria;p__Firmicutes;c__Clostridia;o__Peptostreptococcales-Tissierellales;f__Peptostreptococcaceae;g__Peptostreptococcus;s__Peptostreptococcus_anaerobius	2	0	3	27	1	29	species	2	10.00%	3.33%	2
d__Bacteria;p__Bacteroidota;c__Bacteroidia;o__Bacteroidales;f__Prevotellaceae;g__Prevotella;s__Prevotella_bivia	4	2	5	25	3	27	species	2	16.67%	10.00%	2
d__Bacteria;p__Firmicutes;c__Clostridia;o__Oscillospirales;f__Butyricicoccaceae;g__Butyricicoccus;s__Butyricicoccus_pullicaecorum	0	2	0	30	2	28	species	-2	0.00%	6.67%	2
d__Bacteria;p__Proteobacteria;c__Gammaproteobacteria;o__Pseudomonadales;f__Moraxellaceae;g__Acinetobacter;s__Acinetobacter_pittii	0	2	0	30	2	28	species	-2	0.00%	6.67%	2
d__Bacteria;p__Firmicutes;c__Clostridia;o__Oscillospirales;f__[Eubacterium]_coprostanoligenes_group;g__[Eubacterium]_coprostanoligenes_group;s__Eubacteriaceae_bacterium	1	3	1	29	3	27	species	-2	3.33%	10.00%	2
d__Bacteria;p__Firmicutes;c__Clostridia;o__Lachnospirales;f__Lachnospiraceae;g__Coprococcus;s__Coprococcus_eutactus	2	0	2	28	0	30	species	2	6.67%	0.00%	2
d__Bacteria;p__Firmicutes;c__Clostridia;o__Lachnospirales;f__Lachnospiraceae;g__Lachnoclostridium;s__[Clostridium]_scindens	5	3	7	23	5	25	species	2	23.33%	16.67%	2
d__Bacteria;p__Actinobacteriota;c__Actinobacteria;o__Bifidobacteriales;f__Bifidobacteriaceae;g__Bifidobacterium;s__Bifidobacterium_animalis	3	1	3	27	1	29	species	2	10.00%	3.33%	2
d__Bacteria;p__Bacteroidota;c__Bacteroidia;o__Bacteroidales;f__Prevotellaceae;g__Prevotellaceae_UCG-001;s__Marseilla_massiliensis	2	4	2	28	4	26	species	-2	6.67%	13.33%	2
d__Bacteria;p__Bacteroidota;c__Bacteroidia;o__Bacteroidales;f__Bacteroidaceae;g__Bacteroides;s__Bacteroides_clarus	3	5	4	26	6	24	species	-2	13.33%	20.00%	2
d__Bacteria;p__Proteobacteria;c__Gammaproteobacteria;o__Pseudomonadales;f__Pseudomonadaceae;g__Pseudomonas;s__Pseudomonas_yamanorum	0	2	0	30	2	28	species	-2	0.00%	6.67%	2
d__Bacteria;p__Bacteroidota;c__Bacteroidia;o__Bacteroidales;f__Bacteroidaceae;g__Bacteroides;s__Bacteroides_intestinalis	4	2	4	26	2	28	species	2	13.33%	6.67%	2
d__Bacteria;p__Bacteroidota;c__Bacteroidia;o__Bacteroidales;f__Bacteroidaceae;g__Bacteroides;s__Bacteroides_dorei	5	7	15	15	17	13	species	-2	50.00%	56.67%	2
d__Bacteria;p__Desulfobacterota;c__Desulfovibrionia;o__Desulfovibrionales;f__Desulfovibrionaceae;g__Desulfovibrio;s__Desulfovibrio_desulfuricans	4	2	5	25	3	27	species	2	16.67%	10.00%	2
d__Bacteria;p__Bacteroidota;c__Bacteroidia;o__Bacteroidales;f__Bacteroidaceae;g__Bacteroides;s__Bacteroides_plebeius	7	9	11	19	13	17	species	-2	36.67%	43.33%	2
d__Bacteria;p__Firmicutes;c__Bacilli;o__RF39;f__RF39;g__RF39;s__human_gut	3	1	3	27	1	29	species	2	10.00%	3.33%	2
d__Bacteria;p__Firmicutes;c__Clostridia;o__Lachnospirales;f__Lachnospiraceae;g__Lachnoclostridium;s__Faecalicatena_orotica	2	0	2	28	0	30	species	2	6.67%	0.00%	2
d__Bacteria;p__Firmicutes;c__Bacilli;o__Lactobacillales;f__Streptococcaceae;g__Streptococcus;s__Streptococcus_constellatus	2	0	2	28	0	30	species	2	6.67%	0.00%	2

d__Bacteria;p__Firmicutes;c__Bacilli;o__Lactobacillales;f__Lactobacillaceae;g__Lactobacillus;s__Lactobacillus_delbrueckii	2	0	2	28	0	30	species	2	6.67%	0.00%	2
d__Bacteria;p__Bacteroidota;c__Bacteroidia;o__Bacteroidales;f__Marinifilaceae;g__Butyricimonas;s__Butyricimonas_virosa	6	4	10	20	8	22	species	2	33.33%	26.67%	2
d__Bacteria;p__Bacteroidota;c__Bacteroidia;o__Bacteroidales;f__Prevotellaceae;g__Prevotella;s__Prevotella_colorans	3	1	3	27	1	29	species	2	10.00%	3.33%	2
d__Bacteria;p__Actinobacteriota;c__Actinobacteria;o__Bifidobacteriales;f__Bifidobacteriaceae;g__Bifidobacterium;s__Bifidobacterium_dentium	4	2	4	26	2	28	species	2	13.33%	6.67%	2
d__Bacteria;p__Proteobacteria;c__Gammaproteobacteria;o__Xanthomonadales;f__Xanthomonadaceae;g__Stenotrophomonas;s__Stenotrophomonas_maltophilia	2	0	2	28	0	30	species	2	6.67%	0.00%	2
d__Bacteria;p__Fusobacteriota;c__Fusobacteriia;o__Fusobacteriales;f__Fusobacteriaceae;g__Fusobacterium;s__Fusobacterium_nucleatum	3	1	3	27	1	29	species	2	10.00%	3.33%	2
d__Bacteria;p__Proteobacteria;c__Gammaproteobacteria;o__Burkholderiales;f__Comamonadaceae;g__Comamonas;s__Comamonas_testosteroni	0	2	0	30	2	28	species	-2	0.00%	6.67%	2
d__Bacteria;p__Firmicutes;c__Clostridia;o__Lachnospirales;f__Lachnospiraceae;g__Lachnoclostridium;s__[Clostridium]_citroniae	4	2	5	25	3	27	species	2	16.67%	10.00%	2
d__Bacteria;p__Firmicutes;c__Clostridia;o__Lachnospirales;f__Lachnospiraceae;g__Anaerosporobacter;s__bacterium_NLAE-zl-C571	4	2	4	26	2	28	species	2	13.33%	6.67%	2
d__Bacteria;p__Bacteroidota;c__Bacteroidia;o__Bacteroidales;f__Tannerellaceae;g__Parabacteroides;s__Parabacteroides_merdae	7	5	21	9	19	11	species	2	70.00%	63.33%	2
d__Bacteria;p__Bacteroidota;c__Bacteroidia;o__Bacteroidales;f__Prevotellaceae;g__Prevotella;s__Prevotella_buccalis	2	4	2	28	4	26	species	-2	6.67%	13.33%	2
d__Bacteria;p__Firmicutes;c__Bacilli;o__Lactobacillales;f__Streptococcaceae;g__Streptococcus;s__Streptococcus_parasanguinis	4	6	14	16	16	14	species	-2	46.67%	53.33%	2
d__Bacteria;p__Bacteroidota;c__Bacteroidia;o__Bacteroidales;f__Rikenellaceae;g__Alistipes;s__Alistipes_timonensis	4	6	6	24	8	22	species	-2	20.00%	26.67%	2
d__Bacteria;p__Firmicutes;c__Clostridia;o__Peptococcales;f__Peptococcaceae;s__metagenome	2	0	2	28	0	30	species	2	6.67%	0.00%	2
d__Bacteria;p__Firmicutes;c__Bacilli;o__Erysipelotrichales;f__Erysipelatoclostridiaceae;g__Erysipelotrichaceae_UCG-003;s__Erysipelotrichaceae_bacterium	6	4	9	21	7	23	species	2	30.00%	23.33%	2
d__Bacteria;p__Firmicutes;c__Bacilli;o__Lactobacillales;f__Streptococcaceae;g__Streptococcus;s__Streptococcus_sp.	0	2	0	30	2	28	species	-2	0.00%	6.67%	2
d__Bacteria;p__Firmicutes;c__Clostridia;o__Oscillospirales;f__Oscillospiraceae;g__Oscillibacter;s__human_gut	6	4	26	4	24	6	species	2	86.67%	80.00%	2
d__Bacteria;p__Firmicutes;c__Bacilli;o__Erysipelotrichales;f__Erysipelotrichaceae;g__Dielma;s__Dielma_fastidiosa	7	5	8	22	6	24	species	2	26.67%	20.00%	2
d__Bacteria;p__Actinobacteriota;c__Actinobacteria;o__Bifidobacteriales;f__Bifidobacteriaceae;g__Bifidobacterium;s__Bifidobacterium_bifidum	3	5	5	25	7	23	species	-2	16.67%	23.33%	2
d__Bacteria;p__Firmicutes;c__Clostridia;o__Oscillospirales;f__Butyricicoccaceae;g__Butyricicoccus;s__Butyricicoccus_sp.	5	3	5	25	3	27	species	2	16.67%	10.00%	2
d__Bacteria;p__Firmicutes;c__Incertae_Sedis;o__DTU014;f__DTU014;g__DTU014;s__unidentified	8	6	8	22	6	24	species	2	26.67%	20.00%	2
d__Bacteria;p__Firmicutes;c__Clostridia;o__Peptostreptococcales-Tissierellales;f__Peptostreptococcales-Tissierellales;g__Peptoniphilus;s__Peptoniphilus_urinimassiliensis	3	1	3	27	1	29	species	2	10.00%	3.33%	2
d__Bacteria;p__Actinobacteriota;c__Actinobacteria;o__Corynebacteriales;f__Corynebacteriaceae;g__Corynebacterium;s__Corynebacterium_xerosis	2	0	2	28	0	30	species	2	6.67%	0.00%	2
d__Bacteria;p__Firmicutes;c__Bacilli;o__Lactobacillales;f__Lactobacillaceae;g__Lactobacillus;s__Lactobacillus_acidophilus	0	2	0	30	2	28	species	-2	0.00%	6.67%	2
d__Bacteria;p__Firmicutes;c__Negativicutes;o__Veillonellales-Selenomonadales;f__Veillonellaceae;g__Dialister;s__Dialister_propionicifaciens	3	1	3	27	1	29	species	2	10.00%	3.33%	2
d__Bacteria;p__Firmicutes;c__Clostridia;o__Lachnospirales;f__Lachnospiraceae;g__Lachnospiraceae;s__Cuneatibacter_caecimuris	3	1	3	27	1	29	species	2	10.00%	3.33%	2
d__Bacteria;p__Firmicutes;c__Clostridia;o__Peptostreptococcales-Tissierellales;f__Peptostreptococcaceae;g__Peptostreptococcus;s__Peptostreptococcus_stomatis	2	4	2	28	4	26	species	-2	6.67%	13.33%	2
d__Bacteria;p__Firmicutes;c__Negativicutes;o__Veillonellales-Selenomonadales;f__Veillonellaceae;g__Megasphaera;s__Megasphaera_elsdenii	3	1	3	27	1	29	species	2	10.00%	3.33%	2
d__Bacteria;p__Firmicutes;c__Clostridia;o__Oscillospirales;f__Butyricicoccaceae;g__Butyricicoccus;s__Agathobaculum_sp.	6	8	7	23	9	21	species	-2	23.33%	30.00%	2
d__Bacteria;p__Firmicutes;c__Bacilli;o__Erysipelotrichales;f__Erysipelatoclostridiaceae;g__Erysipelatoclostridium;s__Erysipelatoclostridium_ramosum	7	5	10	20	8	22	species	2	33.33%	26.67%	2
d__Bacteria;p__Proteobacteria;c__Gammaproteobacteria;o__Enterobacterales;f__Morganellaceae;g__Providencia;s__Providencia_sp.	0	2	0	30	2	28	species	-2	0.00%	6.67%	2

d__Bacteria;p__Firmicutes;c__Clostridia;o__Lachnospirales;f__Lachnospiraceae;g__Lachnoclostridium;s__bacterium_NLAE-zl-G277	0	2	0	30	2	28	species	-2	0.00%	6.67%	2
d__Bacteria;p__Bacteroidota;c__Bacteroidia;o__Bacteroidales;f__Prevotellaceae;g__Prevotella;s__Prevotella_disiens	2	4	3	27	5	25	species	-2	10.00%	16.67%	2
d__Bacteria;p__Firmicutes;c__Clostridia;o__Oscillospirales;f__Ruminococcaceae;g__Ruminococcus;s__metagenome	10	8	16	14	14	16	species	2	53.33%	46.67%	2
d__Bacteria;p__Firmicutes;c__Clostridia;o__Clostridia_vadinBB60_group;f__Clostridia_vadinBB60_group;g__Clostridia_vadinBB60_group;s__gut_metagenome	7	5	15	15	13	17	species	2	50.00%	43.33%	2
d__Bacteria;p__Firmicutes;c__Clostridia;o__Oscillospirales;f__Oscillospiraceae;s__unidentified	4	2	5	25	3	27	species	2	16.67%	10.00%	2
d__Bacteria;p__Firmicutes;c__Clostridia;o__Oscillospirales;f__[Clostridium]_methylpentosum_group;g__[Clostridium]_methylpentosum_group;s__Ruminococcus_sp.	5	7	5	25	7	23	species	-2	16.67%	23.33%	2
d__Bacteria;p__Bacteroidota;c__Bacteroidia;o__Bacteroidales;f__Bacteroidaceae;g__Bacteroides;s__Bacteroides_salyersiae	2	4	4	26	6	24	species	-2	13.33%	20.00%	2
d__Bacteria;p__Firmicutes;c__Clostridia;o__Peptostreptococcales-Tissierellales;f__Peptostreptococcales-Tissierellales;g__Peptoniphilus;s__Peptoniphilus_obesi	2	0	2	28	0	30	species	2	6.67%	0.00%	2
d__Bacteria;p__Actinobacteriota;c__Coriobacteriia;o__Coriobacteriales;f__Atopobiaceae;g__Atopobium;s__Atopobium_vaginae	0	2	0	30	2	28	species	-2	0.00%	6.67%	2
d__Bacteria;p__Proteobacteria;c__Gammaproteobacteria;o__Pseudomonadales;f__Moraxellaceae;g__Acinetobacter;s__bacterium_28S421	0	2	0	30	2	28	species	-2	0.00%	6.67%	2
d__Bacteria;p__Desulfobacterota	1	2	28	2	29	1	phylum	-1	93.33%	96.67%	1
d__Bacteria;p__Actinobacteriota;c__Actinobacteria	0	1	29	1	30	0	class	-1	96.67%	100.0%	1
d__Bacteria;p__Firmicutes;c__Syntrophomonadia	0	1	0	30	1	29	class	-1	0.00%	3.33%	1
d__Bacteria;p__Actinobacteriota;c__Coriobacteriia	0	1	29	1	30	0	class	-1	96.67%	100.0%	1
d__Bacteria;p__Firmicutes;c__Moorellia	0	1	0	30	1	29	class	-1	0.00%	3.33%	1
d__Bacteria;p__Verrucomicrobiota;c__Verrucomicrobiae	7	6	18	12	17	13	class	1	60.00%	56.67%	1
d__Bacteria;p__Desulfobacterota;c__Desulfovibrionia	1	2	28	2	29	1	class	-1	93.33%	96.67%	1
d__Bacteria;p__Proteobacteria;c__Gammaproteobacteria;o__Alteromonadales	0	1	0	30	1	29	order	-1	0.00%	3.33%	1
d__Bacteria;p__Firmicutes;c__Moorellia;o__Desulfitibacterales	0	1	0	30	1	29	order	-1	0.00%	3.33%	1
d__Bacteria;p__Proteobacteria;c__Alphaproteobacteria;o__Acetobacterales	1	0	1	29	0	30	order	1	3.33%	0.00%	1
d__Bacteria;p__Proteobacteria;c__Gammaproteobacteria;o__Cardiobacteriales	1	0	1	29	0	30	order	1	3.33%	0.00%	1
d__Bacteria;p__Firmicutes;c__Clostridia;o__Caldicoprobacterales	0	1	0	30	1	29	order	-1	0.00%	3.33%	1
d__Bacteria;p__Actinobacteriota;c__Coriobacteriia;o__Coriobacteriales	0	1	29	1	30	0	order	-1	96.67%	100.0%	1
d__Bacteria;p__Verrucomicrobiota;c__Verrucomicrobiae;o__Verrucomicrobiales	5	6	15	15	16	14	order	-1	50.00%	53.33%	1
d__Bacteria;p__Desulfobacterota;c__Desulfovibrionia;o__Desulfovibrionales	1	2	28	2	29	1	order	-1	93.33%	96.67%	1
d__Bacteria;p__Proteobacteria;c__Alphaproteobacteria;o__Rhodospirillales	6	7	12	18	13	17	order	-1	40.00%	43.33%	1
d__Bacteria;p__Firmicutes;c__Bacilli;o__Paenibacillales	0	1	0	30	1	29	order	-1	0.00%	3.33%	1
d__Bacteria;p__Firmicutes;c__Clostridia;o__Clostridia_vadinBB60_group	5	4	25	5	24	6	order	1	83.33%	80.00%	1
d__Bacteria;p__Proteobacteria;c__Gammaproteobacteria;o__Pasteurellales	6	7	21	9	22	8	order	-1	70.00%	73.33%	1
d__Bacteria;p__Proteobacteria;c__Gammaproteobacteria;o__Enterobacterales	0	1	29	1	30	0	order	-1	96.67%	100.0%	1
d__Bacteria;p__Proteobacteria;c__Gammaproteobacteria;o__Burkholderiales	1	0	30	0	29	1	order	1	100.0%	96.67%	1

d__Bacteria;p__Proteobacteria;c__Gammaproteobacteria;o__Xanthomonadales	3	2	3	27	2	28	order	1	10.00%	6.67%	1
d__Bacteria;p__Firmicutes;c__Syntrophomonadia;o__Syntrophomonadales	0	1	0	30	1	29	order	-1	0.00%	3.33%	1
d__Bacteria;p__Proteobacteria;c__Alphaproteobacteria;o__Rhizobiales	2	1	2	28	1	29	order	1	6.67%	3.33%	1
d__Bacteria;p__Proteobacteria;c__Gammaproteobacteria;o__Aeromonadales	2	3	2	28	3	27	order	-1	6.67%	10.00%	1
d__Bacteria;p__Actinobacteriota;c__Actinobacteria;o__Streptomycetales	1	0	1	29	0	30	order	1	3.33%	0.00%	1
d__Bacteria;p__Verrucomicrobiota;c__Lentisphaeria;o__Victivallales;f__vadinBE97	5	4	6	24	5	25	family	1	20.00%	16.67%	1
d__Bacteria;p__Firmicutes;c__Bacilli;o__Lactobacillales;f__Streptococcaceae	1	0	30	0	29	1	family	1	100.0%	96.67%	1
d__Bacteria;p__Patescibacteria;c__Saccharimonadia;o__Saccharimonadales;f__Saccharimonadales	0	1	0	30	1	29	family	-1	0.00%	3.33%	1
d__Bacteria;p__Proteobacteria;c__Gammaproteobacteria;o__Enterobacterales;f__Yersiniaceae	5	4	5	25	4	26	family	1	16.67%	13.33%	1
d__Bacteria;p__Firmicutes;c__Bacilli;o__Lactobacillales;f__Lactobacillaceae	7	8	10	20	11	19	family	-1	33.33%	36.67%	1
d__Bacteria;p__Myxococcota;c__Polyangia;o__Polyangiales;f__Polyangiaceae	0	1	0	30	1	29	family	-1	0.00%	3.33%	1
d__Bacteria;p__Bacteroidota;c__Bacteroidia;o__Bacteroidales;f__Rikenellaceae	1	2	28	2	29	1	family	-1	93.33%	96.67%	1
d__Bacteria;p__Actinobacteriota;c__Coriobacteriia;o__Coriobacteriales;f__Eggerthellaceae	0	1	29	1	30	0	family	-1	96.67%	100.0%	1
d__Bacteria;p__Proteobacteria;c__Gammaproteobacteria;o__Burkholderiales;f__Nitrosomonadaceae	0	1	0	30	1	29	family	-1	0.00%	3.33%	1
d__Bacteria;p__Proteobacteria;c__Alphaproteobacteria;o__Sphingomonadales;f__Sphingomonadaceae	2	3	2	28	3	27	family	-1	6.67%	10.00%	1
d__Bacteria;p__Proteobacteria;c__Alphaproteobacteria;o__Rhizobiales;f__Beijerinckiaceae	0	1	0	30	1	29	family	-1	0.00%	3.33%	1
d__Bacteria;p__Firmicutes;c__Clostridia;o__Eubacteriales;f__Eubacteriaceae	2	3	4	26	5	25	family	-1	13.33%	16.67%	1
d__Bacteria;p__Proteobacteria;c__Gammaproteobacteria;o__Xanthomonadales;f__Xanthomonadaceae	3	2	3	27	2	28	family	1	10.00%	6.67%	1
d__Bacteria;p__Firmicutes;c__Syntrophomonadia;o__Syntrophomonadales;f__Syntrophomonadaceae	0	1	0	30	1	29	family	-1	0.00%	3.33%	1
d__Bacteria;p__Firmicutes;c__Bacilli;o__Lactobacillales;f__Leuconostocaceae	4	3	5	25	4	26	family	1	16.67%	13.33%	1
d__Bacteria;p__Firmicutes;c__Bacilli;o__Paenibacillales;f__Paenibacillaceae	0	1	0	30	1	29	family	-1	0.00%	3.33%	1
d__Bacteria;p__Firmicutes;c__Negativicutes;o__Veillonellales-Selenomonadales;f__Sporomusaceae	1	0	1	29	0	30	family	1	3.33%	0.00%	1
d__Bacteria;p__Proteobacteria;c__Gammaproteobacteria;o__Cardiobacteriales;f__Cardiobacteriaceae	1	0	1	29	0	30	family	1	3.33%	0.00%	1
d__Bacteria;p__Firmicutes;c__Clostridia;o__Oscillospirales;f__[Eubacterium]_coprostanoligenes_group	2	3	27	3	28	2	family	-1	90.00%	93.33%	1
d__Bacteria;p__Actinobacteriota;c__Actinobacteria;o__Micrococcales;f__Dermatophilaceae	1	0	1	29	0	30	family	1	3.33%	0.00%	1
d__Bacteria;p__Firmicutes;c__Clostridia;o__Oscillospirales;f__Butyricicoccaceae	0	1	29	1	30	0	family	-1	96.67%	100.0%	1
d__Bacteria;p__Firmicutes;c__Clostridia;o__Peptococcales;f__Peptococcaceae	9	8	17	13	16	14	family	1	56.67%	53.33%	1
d__Bacteria;p__Proteobacteria;c__Gammaproteobacteria;o__Burkholderiales;f__Rhodocyclaceae	0	1	0	30	1	29	family	-1	0.00%	3.33%	1
d__Bacteria;p__Firmicutes;c__Clostridia;o__Caldicoprobacterales;f__Caldicoprobacteraceae	0	1	0	30	1	29	family	-1	0.00%	3.33%	1
d__Bacteria;p__Firmicutes;c__Bacilli;o__Lactobacillales;f__Aerococcaceae	3	2	3	27	2	28	family	1	10.00%	6.67%	1
d__Bacteria;p__Proteobacteria;c__Gammaproteobacteria;o__Aeromonadales;f__Aeromonadaceae	1	2	1	29	2	28	family	-1	3.33%	6.67%	1

d__Bacteria;p__Proteobacteria;c__Gammaproteobacteria;o__Burkholderiales;f__Alcaligenaceae	1	0	1	29	0	30	family	1	3.33%	0.00%	1
d__Bacteria;p__Firmicutes;c__Bacilli;o__Erysipelotrichales;f__Erysipelotrichaceae	0	1	29	1	30	0	family	-1	96.67%	100.0%	1
d__Bacteria;p__Firmicutes;c__Clostridia;o__Oscillospirales;f__[Clostridium]_methylpentosum_group	8	7	12	18	11	19	family	1	40.00%	36.67%	1
d__Bacteria;p__Verrucomicrobiota;c__Verrucomicrobiae;o__Opitutales;f__Puniceicoccaceae	6	5	6	24	5	25	family	1	20.00%	16.67%	1
d__Bacteria;p__Proteobacteria;c__Alphaproteobacteria;o__Rhizobiales;f__Rhizobiaceae	2	1	2	28	1	29	family	1	6.67%	3.33%	1
d__Bacteria;p__Firmicutes;c__Bacilli;o__Lactobacillales;f__P5D1-392	0	1	0	30	1	29	family	-1	0.00%	3.33%	1
d__Bacteria;p__Proteobacteria;c__Gammaproteobacteria;o__Enterobacterales;f__Enterobacteriaceae	0	1	29	1	30	0	family	-1	96.67%	100.0%	1
d__Bacteria;p__Actinobacteriota;c__Actinobacteria;o__Micrococcales;f__Micrococcaceae	3	4	3	27	4	26	family	-1	10.00%	13.33%	1
d__Bacteria;p__Firmicutes;c__Clostridia;o__Peptostreptococcales-Tissierellales;f__Peptostreptococcaceae	0	1	29	1	30	0	family	-1	96.67%	100.0%	1
d__Bacteria;p__Firmicutes;c__Clostridia;o__Eubacteriales;f__Garciellaceae	0	1	0	30	1	29	family	-1	0.00%	3.33%	1
d__Bacteria;p__Firmicutes;c__Moorellia;o__Desulfitibacterales;f__Desulfitibacteraceae	0	1	0	30	1	29	family	-1	0.00%	3.33%	1
d__Bacteria;p__Firmicutes;c__Negativicutes;o__Veillonellales-Selenomonadales;f__Veillonellaceae	1	2	27	3	28	2	family	-1	90.00%	93.33%	1
d__Bacteria;p__Proteobacteria;c__Alphaproteobacteria;o__Acetobacterales;f__Acetobacteraceae	1	0	1	29	0	30	family	1	3.33%	0.00%	1
d__Bacteria;p__Desulfobacterota;c__Desulfovibrionia;o__Desulfovibrionales;f__Desulfovibrionaceae	1	2	28	2	29	1	family	-1	93.33%	96.67%	1
d__Bacteria;p__Campilobacterota;c__Campylobacteria;o__Campylobacterales;f__Arcobacteraceae	0	1	0	30	1	29	family	-1	0.00%	3.33%	1
d__Bacteria;p__Proteobacteria;c__Gammaproteobacteria;o__Alteromonadales;f__Alteromonadaceae	0	1	0	30	1	29	family	-1	0.00%	3.33%	1
d__Bacteria;p__Verrucomicrobiota;c__Verrucomicrobiae;o__Verrucomicrobiales;f__Akkermansiaceae	5	6	15	15	16	14	family	-1	50.00%	53.33%	1
d__Bacteria;p__Actinobacteriota;c__Actinobacteria;o__Streptomycetales;f__Streptomycetaceae	1	0	1	29	0	30	family	1	3.33%	0.00%	1
d__Bacteria;p__Firmicutes;c__Clostridia;o__Clostridia_vadinBB60_group;f__Clostridia_vadinBB60_group	5	4	25	5	24	6	family	1	83.33%	80.00%	1
d__Bacteria;p__Actinobacteriota;c__Actinobacteria;o__Micrococcales;f__Promicromonosporaceae	0	1	0	30	1	29	family	-1	0.00%	3.33%	1
d__Bacteria;p__Proteobacteria;c__Gammaproteobacteria;o__Enterobacterales;f__Hafniaceae	3	2	3	27	2	28	family	1	10.00%	6.67%	1
d__Bacteria;p__Actinobacteriota;c__Actinobacteria;o__Actinomycetales;f__Actinomycetaceae	5	4	23	7	22	8	family	1	76.67%	73.33%	1
d__Bacteria;p__Bacteroidota;c__Bacteroidia;o__Bacteroidales;f__Muribaculaceae	7	8	8	22	9	21	family	-1	26.67%	30.00%	1
d__Bacteria;p__Actinobacteriota;c__Coriobacteriia;o__Coriobacteriales;f__Coriobacteriaceae;g__Collinsella	8	7	22	8	21	9	genus	1	73.33%	70.00%	1
d__Bacteria;p__Proteobacteria;c__Gammaproteobacteria;o__Burkholderiales;f__Oxalobacteraceae;g__Noviherbaspirillum	1	0	1	29	0	30	genus	1	3.33%	0.00%	1
d__Bacteria;p__Proteobacteria;c__Gammaproteobacteria;o__Aeromonadales;f__Aeromonadaceae;g__Aeromonas	1	2	1	29	2	28	genus	-1	3.33%	6.67%	1
d__Bacteria;p__Firmicutes;c__Clostridia;o__Lachnospirales;f__Lachnospiraceae;g__Anaerostignum	2	3	2	28	3	27	genus	-1	6.67%	10.00%	1
d__Bacteria;p__Firmicutes;c__Clostridia;o__Lachnospirales;f__Lachnospiraceae;g__[Eubacterium]_eligens_group	5	4	25	5	24	6	genus	1	83.33%	80.00%	1
d__Bacteria;p__Firmicutes;c__Clostridia;o__Christensenellales;f__Christensenellaceae;g__Catabacter	4	3	4	26	3	27	genus	1	13.33%	10.00%	1
d__Bacteria;p__Proteobacteria;c__Gammaproteobacteria;o__Burkholderiales;f__Burkholderiaceae;g__Pandoraea	0	1	0	30	1	29	genus	-1	0.00%	3.33%	1
d__Bacteria;p__Firmicutes;c__Clostridia;o__Lachnospirales;f__Lachnospiraceae;g__CAG-56	5	6	16	14	17	13	genus	-1	53.33%	56.67%	1

d__Bacteria;p__Proteobacteria;c__Gammaproteobacteria;o__Xanthomonadales;f__Xanthomonadaceae;g__Pseudoxanthomonas	1	0	1	29	0	30	genus	1	3.33%	0.00%	1
d__Bacteria;p__Firmicutes;c__Clostridia;o__Peptostreptococcales-Tissierellales;f__Peptostreptococcales-Tissierellales;g__Parvimonas	5	6	6	24	7	23	genus	-1	20.00%	23.33%	1
d__Bacteria;p__Proteobacteria;c__Gammaproteobacteria;o__Pasteurellales;f__Pasteurellaceae;g__Actinobacillus	1	0	1	29	0	30	genus	1	3.33%	0.00%	1
d__Bacteria;p__Firmicutes;c__Clostridia;o__Peptostreptococcales-Tissierellales;f__Peptostreptococcaceae;g__Peptostreptococcaceae	0	1	0	30	1	29	genus	-1	0.00%	3.33%	1
d__Bacteria;p__Firmicutes;c__Bacilli;o__Erysipelotrichales;f__Erysipelatoclostridiaceae;g__Erysipelatoclostridiaceae	0	1	0	30	1	29	genus	-1	0.00%	3.33%	1
d__Bacteria;p__Firmicutes;c__Negativicutes;o__Veillonellales-Selenomonadales;f__Sporomusaceae;g__Anaerosporomusa	1	0	1	29	0	30	genus	1	3.33%	0.00%	1
d__Bacteria;p__Actinobacteriota;c__Actinobacteria;o__Micrococcales;f__Promicromonosporaceae;g__Cellulosimicrobium	0	1	0	30	1	29	genus	-1	0.00%	3.33%	1
d__Bacteria;p__Firmicutes;c__Clostridia;o__Lachnospirales;f__Lachnospiraceae;g__[Bacteroides]_pectinophilus_group	1	0	1	29	0	30	genus	1	3.33%	0.00%	1
d__Bacteria;p__Firmicutes;c__Bacilli;o__Erysipelotrichales;f__Erysipelotrichaceae;g__Faecalicoccus	2	1	2	28	1	29	genus	1	6.67%	3.33%	1
d__Archaea;p__Thermoplasmatota;c__Thermoplasmata;o__Methanomassiliicoccales;f__Methanomethylophilaceae;g__Candidatus_Methanomethylophilus	1	0	1	29	0	30	genus	1	3.33%	0.00%	1
d__Bacteria;p__Bacteroidota;c__Bacteroidia;o__Bacteroidales;f__Muribaculaceae;g__Muribaculaceae	9	8	10	20	9	21	genus	1	33.33%	30.00%	1
d__Bacteria;p__Firmicutes;c__Clostridia;o__Lachnospirales;f__Lachnospiraceae;g__Fusicatenibacter	2	3	27	3	28	2	genus	-1	90.00%	93.33%	1
d__Bacteria;p__Proteobacteria;c__Gammaproteobacteria;o__Burkholderiales;f__Burkholderiaceae;g__Ralstonia	1	2	1	29	2	28	genus	-1	3.33%	6.67%	1
d__Bacteria;p__Firmicutes;c__Bacilli;o__Lactobacillales;f__Aerococcaceae;g__Eremococcus	1	0	1	29	0	30	genus	1	3.33%	0.00%	1
d__Bacteria;p__Firmicutes;c__Clostridia;o__Oscillospirales;f__Oscillospiraceae;g__Oscillibacter	0	1	29	1	30	0	genus	-1	96.67%	100.0%	1
d__Bacteria;p__Proteobacteria;c__Gammaproteobacteria;o__Burkholderiales;f__Neisseriaceae;g__Neisseria	1	0	1	29	0	30	genus	1	3.33%	0.00%	1
d__Bacteria;p__Firmicutes;c__Clostridia;o__Lachnospirales;f__Lachnospiraceae;g__GCA-900066575	2	3	25	5	26	4	genus	-1	83.33%	86.67%	1
d__Archaea;p__Thermoplasmatota;c__Thermoplasmata;o__Methanomassiliicoccales;f__Methanomethylophilaceae;g__RumEn_M2	0	1	0	30	1	29	genus	-1	0.00%	3.33%	1
d__Bacteria;p__Firmicutes;c__Clostridia;o__Lachnospirales;f__Lachnospiraceae;g__Epulopiscium	4	3	4	26	3	27	genus	1	13.33%	10.00%	1
d__Bacteria;p__Firmicutes;c__Clostridia;o__Lachnospirales;f__Lachnospiraceae;g__[Eubacterium]_xylanophilum_group	6	5	22	8	21	9	genus	1	73.33%	70.00%	1
d__Bacteria;p__Firmicutes;c__Bacilli;o__Erysipelotrichales;f__Erysipelatoclostridiaceae;g__Coprobacillus	4	3	5	25	4	26	genus	1	16.67%	13.33%	1
d__Bacteria;p__Firmicutes;c__Syntrophomonadia;o__Syntrophomonadales;f__Syntrophomonadaceae;g__Syntrophomonas	0	1	0	30	1	29	genus	-1	0.00%	3.33%	1
d__Bacteria;p__Actinobacteriota;c__Actinobacteria;o__Micrococcales;f__Micrococcaceae;g__Kocuria	0	1	0	30	1	29	genus	-1	0.00%	3.33%	1
d__Bacteria;p__Firmicutes;c__Clostridia;o__Lachnospirales;f__Lachnospiraceae;g__Howardella	4	3	4	26	3	27	genus	1	13.33%	10.00%	1
d__Bacteria;p__Firmicutes;c__Clostridia;o__Peptostreptococcales-Tissierellales;f__Anaerovoracaceae;g__Mogibacterium	4	5	6	24	7	23	genus	-1	20.00%	23.33%	1
d__Bacteria;p__Proteobacteria;c__Alphaproteobacteria;o__Caulobacterales;f__Caulobacteraceae;g__Phenylobacterium	1	0	1	29	0	30	genus	1	3.33%	0.00%	1
d__Bacteria;p__Bacteroidota;c__Bacteroidia;o__Bacteroidales;f__Rikenellaceae;g__Alistipes	1	2	28	2	29	1	genus	-1	93.33%	96.67%	1
d__Bacteria;p__Actinobacteriota;c__Actinobacteria;o__Bifidobacteriales;f__Bifidobacteriaceae;g__Bifidobacterium	1	0	30	0	29	1	genus	1	100.0%	96.67%	1
d__Bacteria;p__Proteobacteria;c__Gammaproteobacteria;o__Burkholderiales;f__Comamonadaceae;g__Delftia	0	1	0	30	1	29	genus	-1	0.00%	3.33%	1
d__Bacteria;p__Firmicutes;c__Clostridia;o__Peptostreptococcales-Tissierellales;f__Peptostreptococcales-Tissierellales;g__Anaerococcus	4	5	5	25	6	24	genus	-1	16.67%	20.00%	1
d__Bacteria;p__Firmicutes;c__Clostridia;o__Clostridiales;f__Clostridiaceae;g__Clostridium_sensu_stricto_3	0	1	0	30	1	29	genus	-1	0.00%	3.33%	1

d__Bacteria;p__Firmicutes;c__Clostridia;o__Lachnospirales;f__Lachnospiraceae;g__[Eubacterium]_ventriosum_group	2	3	26	4	27	3	genus	-1	86.67%	90.00%	1
d__Bacteria;p__Firmicutes;c__Bacilli;o__Erysipelotrichales;f__Erysipelotrichaceae;g__[Clostridium]_innocuum_group	8	9	16	14	17	13	genus	-1	53.33%	56.67%	1
d__Bacteria;p__Firmicutes;c__Clostridia;o__Peptostreptococcales-Tissierellales;f__Peptostreptococcales-Tissierellales;g__W5053	1	0	1	29	0	30	genus	1	3.33%	0.00%	1
d__Bacteria;p__Firmicutes;c__Clostridia;o__Lachnospirales;f__Lachnospiraceae;g__Acetitomaculum	1	0	1	29	0	30	genus	1	3.33%	0.00%	1
d__Bacteria;p__Firmicutes;c__Clostridia;o__Oscillospirales;f__Ruminococcaceae;g__Fournierella	5	4	5	25	4	26	genus	1	16.67%	13.33%	1
d__Bacteria;p__Firmicutes;c__Clostridia;o__Oscillospirales;f__Ruminococcaceae;g__Angelakisella	2	3	2	28	3	27	genus	-1	6.67%	10.00%	1
d__Bacteria;p__Proteobacteria;c__Gammaproteobacteria;o__Enterobacterales;f__Morganellaceae;g__Proteus	1	0	1	29	0	30	genus	1	3.33%	0.00%	1
d__Bacteria;p__Firmicutes;c__Negativicutes;o__Acidaminococcales;f__Acidaminococcaceae;g__Succiniclasticum	1	0	1	29	0	30	genus	1	3.33%	0.00%	1
d__Bacteria;p__Firmicutes;c__Bacilli;o__Lactobacillales;f__Aerococcaceae;g__Aerosphaera	0	1	0	30	1	29	genus	-1	0.00%	3.33%	1
d__Bacteria;p__Firmicutes;c__Clostridia;o__Oscillospirales;f__Oscillospiraceae;g__Papillibacter	3	2	3	27	2	28	genus	1	10.00%	6.67%	1
d__Bacteria;p__Proteobacteria;c__Gammaproteobacteria;o__Burkholderiales;f__Burkholderiaceae;g__Cupriavidus	0	1	0	30	1	29	genus	-1	0.00%	3.33%	1
d__Bacteria;p__Proteobacteria;c__Gammaproteobacteria;o__Enterobacterales;f__Erwiniaceae;g__Rosenbergiella	0	1	0	30	1	29	genus	-1	0.00%	3.33%	1
d__Bacteria;p__Bacteroidota;c__Bacteroidia;o__Flavobacteriales;f__Flavobacteriaceae;g__Flavobacterium	0	1	0	30	1	29	genus	-1	0.00%	3.33%	1
d__Bacteria;p__Firmicutes;c__Bacilli;o__Bacillales;f__Planococcaceae;g__Solibacillus	1	0	1	29	0	30	genus	1	3.33%	0.00%	1
d__Bacteria;p__Actinobacteriota;c__Actinobacteria;o__Micrococcales;f__Micrococcaceae;g__Rothia	3	4	3	27	4	26	genus	-1	10.00%	13.33%	1
d__Bacteria;p__Campilobacterota;c__Campylobacteria;o__Campylobacterales;f__Arcobacteraceae;g__Arcobacter	0	1	0	30	1	29	genus	-1	0.00%	3.33%	1
d__Bacteria;p__Firmicutes;c__Clostridia;o__Peptococcales;f__Peptococcaceae;g__Peptococcus	7	8	9	21	10	20	genus	-1	30.00%	33.33%	1
d__Bacteria;p__Firmicutes;c__Clostridia;o__Oscillospirales;f__Ruminococcaceae;g__Pygmaiobacter	0	1	0	30	1	29	genus	-1	0.00%	3.33%	1
d__Bacteria;p__Proteobacteria;c__Gammaproteobacteria;o__Enterobacterales;f__Morganellaceae;g__Morganella	1	2	1	29	2	28	genus	-1	3.33%	6.67%	1
d__Bacteria;p__Firmicutes;c__Clostridia;o__Lachnospirales;f__Lachnospiraceae;g__Lachnospiraceae_AC2044_group	1	2	1	29	2	28	genus	-1	3.33%	6.67%	1
d__Bacteria;p__Firmicutes;c__Clostridia;o__Oscillospirales;f__Ruminococcaceae;g__Ruminococcus	2	1	29	1	28	2	genus	1	96.67%	93.33%	1
d__Bacteria;p__Firmicutes;c__Bacilli;o__Bacillales;f__Planococcaceae;g__Psychrobacillus	0	1	0	30	1	29	genus	-1	0.00%	3.33%	1
d__Bacteria;p__Firmicutes;c__Bacilli;o__Erysipelotrichales;f__Erysipelotrichaceae;g__Catenisphaera	2	1	2	28	1	29	genus	1	6.67%	3.33%	1
d__Bacteria;p__Firmicutes;c__Negativicutes;o__Veillonellales-Selenomonadales;f__Veillonellaceae;g__Anaeroglobus	0	1	0	30	1	29	genus	-1	0.00%	3.33%	1
d__Bacteria;p__Firmicutes;c__Clostridia;o__Peptostreptococcales-Tissierellales;f__Peptostreptococcaceae;g__Paeniclostridium	2	1	2	28	1	29	genus	1	6.67%	3.33%	1
d__Bacteria;p__Firmicutes;c__Clostridia;o__Peptostreptococcales-Tissierellales;f__Peptostreptococcales-Tissierellales;g__Finegoldia	4	5	5	25	6	24	genus	-1	16.67%	20.00%	1
d__Bacteria;p__Actinobacteriota;c__Actinobacteria;o__Actinomycetales;f__Actinomycetales_Incertae_Sedis;g__Candidatus_Ancillula	0	1	0	30	1	29	genus	-1	0.00%	3.33%	1
d__Bacteria;p__Firmicutes;c__Clostridia;o__Oscillospirales;f__Oscillospiraceae;g__UCG-002	3	2	28	2	27	3	genus	1	93.33%	90.00%	1
d__Bacteria;p__Firmicutes;c__Bacilli;o__Lactobacillales;f__Streptococcaceae;g__Streptococcus	1	0	30	0	29	1	genus	1	100.0%	96.67%	1
d__Bacteria;p__Myxococcota;c__Polyangia;o__Polyangiales;f__Polyangiaceae;g__Minicystis	0	1	0	30	1	29	genus	-1	0.00%	3.33%	1
d__Bacteria;p__Firmicutes;c__Clostridia;o__Lachnospirales;f__Lachnospiraceae;g__Lachnospiraceae_UCG-008	2	1	29	1	28	2	genus	1	96.67%	93.33%	1

d__Bacteria;p__Firmicutes;c__Clostridia;o__Lachnospirales;f__Lachnospiraceae;g__Roseburia	1	0	30	0	29	1	genus	1	100.0%	96.67%	1
d__Bacteria;p__Proteobacteria;c__Gammaproteobacteria;o__Aeromonadales;f__Succinivibrionaceae;g__Succinatimonas	1	0	1	29	0	30	genus	1	3.33%	0.00%	1
d__Bacteria;p__Firmicutes;c__Clostridia;o__Oscillospirales;f__Ruminococcaceae;g__Anaerofilum	9	10	13	17	14	16	genus	-1	43.33%	46.67%	1
d__Bacteria;p__Actinobacteriota;c__Coriobacteriia;o__Coriobacteriales;f__Atopobiaceae;g__Libanicoccus	0	1	0	30	1	29	genus	-1	0.00%	3.33%	1
d__Bacteria;p__Firmicutes;c__Bacilli;o__Lactobacillales;f__P5D1-392;g__P5D1-392	0	1	0	30	1	29	genus	-1	0.00%	3.33%	1
d__Bacteria;p__Firmicutes;c__Bacilli;o__Lactobacillales;f__Leuconostocaceae;g__Fructobacillus	0	1	0	30	1	29	genus	-1	0.00%	3.33%	1
d__Bacteria;p__Firmicutes;c__Clostridia;o__Oscillospirales;f__Oscillospiraceae;g__NK4A214_group	5	6	23	7	24	6	genus	-1	76.67%	80.00%	1
d__Bacteria;p__Proteobacteria;c__Gammaproteobacteria;o__Oceanospirillales;f__Pseudohongiellaceae;g__Pseudohongiella	1	0	1	29	0	30	genus	1	3.33%	0.00%	1
d__Bacteria;p__Firmicutes;c__Clostridia;o__Oscillospirales;f__Ruminococcaceae;g__Faecalibacterium	1	0	30	0	29	1	genus	1	100.0%	96.67%	1
d__Bacteria;p__Proteobacteria;c__Alphaproteobacteria;o__Acetobacterales;f__Acetobacteraceae;g__Roseomonas	1	0	1	29	0	30	genus	1	3.33%	0.00%	1
d__Bacteria;p__Firmicutes;c__Bacilli;o__Erysipelotrichales;f__Erysipelotrichaceae;g__Turicibacter	2	1	27	3	26	4	genus	1	90.00%	86.67%	1
d__Bacteria;p__Firmicutes;c__Clostridia;o__Lachnospirales;f__Lachnospiraceae;g__Stomatobaculum	1	0	1	29	0	30	genus	1	3.33%	0.00%	1
d__Bacteria;p__Firmicutes;c__Clostridia;o__Lachnospirales;f__Lachnospiraceae;g__Lachnoanaerobaculum	1	0	1	29	0	30	genus	1	3.33%	0.00%	1
d__Bacteria;p__Proteobacteria;c__Alphaproteobacteria;o__Caulobacterales;f__Caulobacteraceae;g__Brevundimonas	0	1	0	30	1	29	genus	-1	0.00%	3.33%	1
d__Bacteria;p__Firmicutes;c__Bacilli;o__Lactobacillales;f__Lactobacillaceae;g__Lactobacillus	7	8	10	20	11	19	genus	-1	33.33%	36.67%	1
d__Bacteria;p__Verrucomicrobiota;c__Lentisphaeria;o__Victivallales;f__Victivallaceae;g__Victivallis	10	11	12	18	13	17	genus	-1	40.00%	43.33%	1
d__Bacteria;p__Proteobacteria;c__Gammaproteobacteria;o__Burkholderiales;f__Nitrosomonadaceae;g__Nitrosomonas	0	1	0	30	1	29	genus	-1	0.00%	3.33%	1
d__Bacteria;p__Firmicutes;c__Clostridia;o__Caldicoprobacterales;f__Caldicoprobacteraceae;g__Caldicoprobacter	0	1	0	30	1	29	genus	-1	0.00%	3.33%	1
d__Bacteria;p__Firmicutes;c__Clostridia;o__Lachnospirales;f__Lachnospiraceae;g__Anaerostipes	1	0	30	0	29	1	genus	1	100.0%	96.67%	1
d__Bacteria;p__Actinobacteriota;c__Actinobacteria;o__Corynebacteriales;f__Corynebacteriaceae;g__Corynebacterium	6	5	8	22	7	23	genus	1	26.67%	23.33%	1
d__Bacteria;p__Firmicutes;c__Clostridia;o__Lachnospirales;f__Lachnospiraceae;g__Lachnospiraceae_FCS020_group	5	6	22	8	23	7	genus	-1	73.33%	76.67%	1
d__Bacteria;p__Firmicutes;c__Clostridia;o__Eubacteriales;f__Anaerofustaceae;g__Anaerofustis	3	2	3	27	2	28	genus	1	10.00%	6.67%	1
d__Bacteria;p__Firmicutes;c__Clostridia;o__Eubacteriales;f__Garciellaceae;g__Irregularibacter	0	1	0	30	1	29	genus	-1	0.00%	3.33%	1
d__Bacteria;p__Actinobacteriota;c__Coriobacteriia;o__Coriobacteriales;f__Eggerthellaceae;g__Enteroscipio	1	0	1	29	0	30	genus	1	3.33%	0.00%	1
d__Bacteria;p__Actinobacteriota;c__Coriobacteriia;o__Coriobacteriales;f__Atopobiaceae;g__Coriobacteriaceae_UCG-002	3	2	3	27	2	28	genus	1	10.00%	6.67%	1
d__Bacteria;p__Firmicutes;c__Bacilli;o__Bacillales;f__Planococcaceae;g__[Renibacterium]_salmoninarum_group	0	1	0	30	1	29	genus	-1	0.00%	3.33%	1
d__Bacteria;p__Firmicutes;c__Bacilli;o__Paenibacillales;f__Paenibacillaceae;g__Paenibacillus	0	1	0	30	1	29	genus	-1	0.00%	3.33%	1
d__Bacteria;p__Proteobacteria;c__Gammaproteobacteria;o__Enterobacterales;f__Hafniaceae;g__Hafnia-Obesumbacterium	3	2	3	27	2	28	genus	1	10.00%	6.67%	1
d__Bacteria;p__Firmicutes;c__Clostridia;o__Clostridia;f__Hungateiclostridiaceae;g__Ruminiclostridium	4	3	7	23	6	24	genus	1	23.33%	20.00%	1
d__Bacteria;p__Proteobacteria;c__Gammaproteobacteria;o__Enterobacterales;f__Yersiniaceae;g__Yersinia	2	3	2	28	3	27	genus	-1	6.67%	10.00%	1
d__Bacteria;p__Firmicutes;c__Bacilli;o__Lactobacillales;f__Aerococcaceae;g__Abiotrophia	1	0	1	29	0	30	genus	1	3.33%	0.00%	1

d__Bacteria;p__Firmicutes;c__Bacilli;o__Lactobacillales;f__Aerococcaceae;g__Aerococcus	0	1	0	30	1	29	genus	-1	0.00%	3.33%	1
d__Bacteria;p__Firmicutes;c__Clostridia;o__Clostridia;f__Hungateiclostridiaceae;g__Fastidiosipila	2	1	2	28	1	29	genus	1	6.67%	3.33%	1
d__Bacteria;p__Firmicutes;c__Moorellia;o__Desulfitibacterales;f__Desulfitibacteraceae;g__Desulfitibacter	0	1	0	30	1	29	genus	-1	0.00%	3.33%	1
d__Bacteria;p__Firmicutes;c__Clostridia;o__Lachnospirales;f__Lachnospiraceae;g__28-4	3	2	3	27	2	28	genus	1	10.00%	6.67%	1
d__Bacteria;p__Firmicutes;c__Bacilli;o__Lactobacillales;f__Aerococcaceae;g__Ignavigranum	1	0	1	29	0	30	genus	1	3.33%	0.00%	1
d__Bacteria;p__Firmicutes;c__Clostridia;o__Lachnospirales;f__Lachnospiraceae;g__[Ruminococcus]_gnavus_group	9	8	16	14	15	15	genus	1	53.33%	50.00%	1
d__Bacteria;p__Synergistota;c__Synergistia;o__Synergistales;f__Synergistaceae;g__Jonquetella	0	1	0	30	1	29	genus	-1	0.00%	3.33%	1
d__Bacteria;p__Actinobacteriota;c__Coriobacteriia;o__Coriobacteriales;f__Atopobiaceae;g__Coriobacteriaceae_UCG-003	1	0	1	29	0	30	genus	1	3.33%	0.00%	1
d__Bacteria;p__Actinobacteriota;c__Actinobacteria;o__Streptomycetales;f__Streptomycetaceae;g__Streptomyces	1	0	1	29	0	30	genus	1	3.33%	0.00%	1
d__Bacteria;p__Patescibacteria;c__Saccharimonadia;o__Saccharimonadales;f__Saccharimonadaceae;g__Saccharimonadaceae	1	0	1	29	0	30	genus	1	3.33%	0.00%	1
d__Bacteria;p__Proteobacteria;c__Gammaproteobacteria;o__Pasteurellales;f__Pasteurellaceae;g__Aggregatibacter	1	0	1	29	0	30	genus	1	3.33%	0.00%	1
d__Bacteria;p__Firmicutes;c__Clostridia;o__Lachnospirales;f__Lachnospiraceae;g__Eisenbergiella	5	6	18	12	19	11	genus	-1	60.00%	63.33%	1
d__Bacteria;p__Firmicutes;c__Clostridia;o__Oscillospirales;f__Butyricicoccaceae;g__UCG-008	0	1	0	30	1	29	genus	-1	0.00%	3.33%	1
d__Bacteria;p__Synergistota;c__Synergistia;o__Synergistales;f__Synergistaceae;g__Synergistes	1	0	1	29	0	30	genus	1	3.33%	0.00%	1
d__Bacteria;p__Firmicutes;c__Clostridia;o__Oscillospirales;f__Ruminococcaceae;g__Phocea	6	5	12	18	11	19	genus	1	40.00%	36.67%	1
d__Bacteria;p__Firmicutes;c__Negativicutes;o__Veillonellales-Selenomonadales;f__Veillonellaceae;g__Negativicoccus	1	2	1	29	2	28	genus	-1	3.33%	6.67%	1
d__Bacteria;p__Bacteroidota;c__Bacteroidia;o__Bacteroidales;f__Prevotellaceae;g__Prevotellaceae_Ga6A1_group	0	1	0	30	1	29	genus	-1	0.00%	3.33%	1
d__Bacteria;p__Actinobacteriota;c__Actinobacteria;o__Actinomycetales;f__Actinomycetaceae;g__Trueperella	0	1	0	30	1	29	genus	-1	0.00%	3.33%	1
d__Bacteria;p__Firmicutes;c__Bacilli;o__Erysipelotrichales;f__Erysipelatoclostridiaceae;g__UCG-004	1	2	1	29	2	28	genus	-1	3.33%	6.67%	1
d__Bacteria;p__Firmicutes;c__Clostridia;o__Lachnospirales;f__Lachnospiraceae;g__Butyrivibrio	1	0	1	29	0	30	genus	1	3.33%	0.00%	1
d__Bacteria;p__Actinobacteriota;c__Actinobacteria;o__Actinomycetales;f__Actinomycetaceae;g__Mobiluncus	2	3	2	28	3	27	genus	-1	6.67%	10.00%	1
d__Bacteria;p__Proteobacteria;c__Gammaproteobacteria;o__Alteromonadales;f__Alteromonadaceae;g__Rheinheimera	0	1	0	30	1	29	genus	-1	0.00%	3.33%	1
d__Bacteria;p__Firmicutes;c__Clostridia;o__Lachnospirales;f__Lachnospiraceae;g__Moryella	4	5	5	25	6	24	genus	-1	16.67%	20.00%	1
d__Bacteria;p__Verrucomicrobiota;c__Verrucomicrobiae;o__Verrucomicrobiales;f__Akkermansiaceae;g__Akkermansia	5	6	15	15	16	14	genus	-1	50.00%	53.33%	1
d__Bacteria;p__Bacteroidota;c__Bacteroidia;o__Bacteroidales;f__Muribaculaceae;g__CAG-873	1	0	1	29	0	30	genus	1	3.33%	0.00%	1
d__Bacteria;p__Proteobacteria;c__Gammaproteobacteria;o__Burkholderiales;f__Comamonadaceae;g__Aquabacterium	0	1	0	30	1	29	genus	-1	0.00%	3.33%	1
d__Bacteria;p__Patescibacteria;c__Saccharimonadia;o__Saccharimonadales;f__Saccharimonadales;g__Saccharimonadales	0	1	0	30	1	29	genus	-1	0.00%	3.33%	1
d__Bacteria;p__Firmicutes;c__Clostridia;o__Lachnospirales;f__Lachnospiraceae;g__Lachnospiraceae_UCG-003	1	2	1	29	2	28	genus	-1	3.33%	6.67%	1
d__Bacteria;p__Proteobacteria;c__Gammaproteobacteria;o__Burkholderiales;f__Rhodocyclaceae;g__Dechloromonas	0	1	0	30	1	29	genus	-1	0.00%	3.33%	1
d__Bacteria;p__Actinobacteriota;c__Coriobacteriia;o__Coriobacteriales;f__Eggerthellaceae;g__DNF00809	1	2	1	29	2	28	genus	-1	3.33%	6.67%	1
d__Bacteria;p__Firmicutes;c__Clostridia;o__Christensenellales;f__Christensenellaceae;g__Christensenellaceae_R-7_group	3	2	28	2	27	3	genus	1	93.33%	90.00%	1

d__Bacteria;p__Proteobacteria;c__Alphaproteobacteria;o__Sphingomonadales;f__Sphingomonadaceae;g__Sphingobium	1	2	1	29	2	28	genus	-1	3.33%	6.67%	1
d__Bacteria;p__Actinobacteriota;c__Actinobacteria;o__Actinomycetales;f__Actinomycetaceae;g__Actinotignum	2	3	2	28	3	27	genus	-1	6.67%	10.00%	1
d__Bacteria;p__Firmicutes;c__Clostridia;o__Oscillospirales;f__Oscillospiraceae;g__Flavonifractor	2	1	29	1	28	2	genus	1	96.67%	93.33%	1
d__Bacteria;p__Firmicutes;c__Clostridia;o__Oscillospirales;f__Ruminococcaceae;g__DTU089	9	8	19	11	18	12	genus	1	63.33%	60.00%	1
d__Bacteria;p__Actinobacteriota;c__Coriobacteriia;o__Coriobacteriales;f__Atopobiaceae;g__Olsenella	6	7	10	20	11	19	genus	-1	33.33%	36.67%	1
d__Bacteria;p__Proteobacteria;c__Gammaproteobacteria;o__Cardiobacteriales;f__Cardiobacteriaceae;g__Cardiobacterium	1	0	1	29	0	30	genus	1	3.33%	0.00%	1
d__Bacteria;p__Firmicutes;c__Clostridia;o__Peptostreptococcales-Tissierellales;f__Peptostreptococcales-Tissierellales;g__Murdochiella	3	2	3	27	2	28	genus	1	10.00%	6.67%	1
d__Bacteria;p__Firmicutes;c__Bacilli;o__Bacillales;f__Bacillaceae;g__Anoxybacillus	0	1	0	30	1	29	genus	-1	0.00%	3.33%	1
d__Bacteria;p__Proteobacteria;c__Gammaproteobacteria;o__Burkholderiales;f__Alcaligenaceae;g__Achromobacter	1	0	1	29	0	30	genus	1	3.33%	0.00%	1
d__Bacteria;p__Firmicutes;c__Bacilli;o__Erysipelotrichales;f__Erysipelotrichaceae;g__Faecalibaculum	2	1	2	28	1	29	genus	1	6.67%	3.33%	1
d__Bacteria;p__Actinobacteriota;c__Actinobacteria;o__Micrococcales;f__Microbacteriaceae;g__Leucobacter	1	0	1	29	0	30	genus	1	3.33%	0.00%	1
d__Bacteria;p__Proteobacteria;c__Alphaproteobacteria;o__Rhizobiales;f__Rhizobiaceae;g__Allorhizobium-Neorhizobium-Pararhizobium-Rhizobium	2	1	2	28	1	29	genus	1	6.67%	3.33%	1
d__Bacteria;p__Firmicutes;c__Bacilli;o__Bacillales;f__Bacillaceae;g__Oceanobacillus	0	1	0	30	1	29	genus	-1	0.00%	3.33%	1
d__Bacteria;p__Proteobacteria;c__Gammaproteobacteria;o__Pseudomonadales;f__Moraxellaceae;g__Acinetobacter;s__Acinetobacter_lwoffii	1	0	1	29	0	30	species	1	3.33%	0.00%	1
d__Bacteria;p__Firmicutes;c__Clostridia;o__Lachnospirales;f__Lachnospiraceae;g__Blautia;s__Blautia_glucerasea	2	1	2	28	1	29	species	1	6.67%	3.33%	1
d__Bacteria;p__Fusobacteriota;c__Fusobacteriia;o__Fusobacteriales;f__Fusobacteriaceae;g__Fusobacterium;s__Fusobacterium_gonidiaformans	1	0	1	29	0	30	species	1	3.33%	0.00%	1
d__Bacteria;p__Proteobacteria;c__Gammaproteobacteria;o__Burkholderiales;f__Sutterellaceae;g__Sutterella;s__Sutterella_sp.	1	0	1	29	0	30	species	1	3.33%	0.00%	1
d__Bacteria;p__Actinobacteriota;c__Coriobacteriia;o__Coriobacteriales;f__Coriobacteriaceae;g__Collinsella;s__Collinsella_tanakaei	2	1	2	28	1	29	species	1	6.67%	3.33%	1
d__Bacteria;p__Actinobacteriota;c__Coriobacteriia;o__Coriobacteriales;f__Eggerthellaceae;g__Gordonibacter;s__Eggerthella_sp.	0	1	0	30	1	29	species	-1	0.00%	3.33%	1
d__Bacteria;p__Actinobacteriota;c__Actinobacteria;o__Streptomycetales;f__Streptomycetaceae;g__Streptomyces;s__Streptomyces_albidoflavus	1	0	1	29	0	30	species	1	3.33%	0.00%	1
d__Bacteria;p__Firmicutes;c__Clostridia;o__Oscillospirales;f__Oscillospiraceae;g__Intestinimonas;s__Flavonifractor_plautii	3	2	3	27	2	28	species	1	10.00%	6.67%	1
d__Bacteria;p__Proteobacteria;c__Gammaproteobacteria;o__Burkholderiales;f__Rhodocyclaceae;g__Denitratisoma;s__metagenome	1	0	1	29	0	30	species	1	3.33%	0.00%	1
d__Bacteria;p__Firmicutes;c__Clostridia;o__Peptostreptococcales-Tissierellales;f__Peptostreptococcales-Tissierellales;g__Parvimonas;s__Peptostreptococcus_sp.	2	1	2	28	1	29	species	1	6.67%	3.33%	1
d__Bacteria;p__Actinobacteriota;c__Actinobacteria;o__Bifidobacteriales;f__Bifidobacteriaceae;g__Bifidobacterium;s__metagenome	5	6	23	7	24	6	species	-1	76.67%	80.00%	1
d__Bacteria;p__Firmicutes;c__Clostridia;o__Lachnospirales;f__Lachnospiraceae;g__[Eubacterium]_eligens_group;s__[Eubacterium]_eligens	7	6	17	13	16	14	species	1	56.67%	53.33%	1
d__Bacteria;p__Firmicutes;c__Clostridia;o__Oscillospirales;f__Oscillospiraceae;g__UCG-005;s__metagenome	4	5	4	26	5	25	species	-1	13.33%	16.67%	1
d__Bacteria;p__Firmicutes;c__Bacilli;o__Izemoplasmatales;f__Izemoplasmatales;g__Izemoplasmatales;s__gut_metagenome	1	2	1	29	2	28	species	-1	3.33%	6.67%	1
d__Bacteria;p__Firmicutes;c__Bacilli;o__Erysipelotrichales;f__Erysipelotrichaceae;g__Faecalicoccus;s__Faecalicoccus_pleomorphus	2	1	2	28	1	29	species	1	6.67%	3.33%	1
d__Bacteria;p__Firmicutes;c__Clostridia;o__Christensenellales;f__Christensenellaceae;g__Christensenella;s__Christensenella_sp.	1	0	1	29	0	30	species	1	3.33%	0.00%	1
d__Bacteria;p__Firmicutes;c__Negativicutes;o__Veillonellales-Selenomonadales;f__Veillonellaceae;g__Dialister;s__Dialister_micraerophilus	2	1	2	28	1	29	species	1	6.67%	3.33%	1
d__Bacteria;p__Synergistota;c__Synergistia;o__Synergistales;f__Synergistaceae;g__Jonquetella;s__Jonquetella_anthropi	0	1	0	30	1	29	species	-1	0.00%	3.33%	1

d__Bacteria;p__Proteobacteria;c__Gammaproteobacteria;o__Burkholderiales;f__Neisseriaceae;g__Neisseria;s__Neisseria_perflava	1	0	1	29	0	30	species	1	3.33%	0.00%	1
d__Bacteria;p__Actinobacteriota;c__Coriobacteriia;o__Coriobacteriales;f__Coriobacteriales_Incertae_Sedis;g__Raoultibacter;s__Raoultibacter_timonensis	1	2	1	29	2	28	species	-1	3.33%	6.67%	1
d__Bacteria;p__Actinobacteriota;c__Actinobacteria;o__Micrococcales;f__Micrococcaceae;g__Rothia;s__Rothia_sp.	1	2	1	29	2	28	species	-1	3.33%	6.67%	1
d__Bacteria;p__Proteobacteria;c__Alphaproteobacteria;o__Rhizobiales;f__Rhizobiaceae;g__Allorhizobium-Neorhizobium-Pararhizobium-Rhizobium;s__Rhizobium_gei	1	0	1	29	0	30	species	1	3.33%	0.00%	1
d__Bacteria;p__Firmicutes;c__Clostridia;o__Lachnospirales;f__Lachnospiraceae;g__Blautia;s__Ruminococcus_sp.	5	4	6	24	5	25	species	1	20.00%	16.67%	1
d__Bacteria;p__Firmicutes;c__Clostridia;o__Christensenellales;f__Christensenellaceae;g__Christensenellaceae_R-7_group;s__Clostridiales_bacterium	2	1	2	28	1	29	species	1	6.67%	3.33%	1
d__Bacteria;p__Fusobacteriota;c__Fusobacteriia;o__Fusobacteriales;f__Fusobacteriaceae;g__Fusobacterium;s__Fusobacterium_varium	0	1	0	30	1	29	species	-1	0.00%	3.33%	1
d__Bacteria;p__Firmicutes;c__Bacilli;o__Lactobacillales;f__Lactobacillaceae;g__Lactobacillus;s__Lactobacillus_fermentum	2	1	2	28	1	29	species	1	6.67%	3.33%	1
d__Bacteria;p__Firmicutes;c__Clostridia;o__Oscillospirales;f__Butyricicoccaceae;g__Butyricicoccus;s__Intestinibacillus_sp.	3	4	3	27	4	26	species	-1	10.00%	13.33%	1
d__Bacteria;p__Firmicutes;c__Clostridia;o__Peptostreptococcales-Tissierellales;f__Peptostreptococcales-Tissierellales;g__Murdochiella;s__unidentified	1	0	1	29	0	30	species	1	3.33%	0.00%	1
d__Bacteria;p__Synergistota;c__Synergistia;o__Synergistales;f__Synergistaceae;g__Cloacibacillus;s__Cloacibacillus_porcorum	0	1	0	30	1	29	species	-1	0.00%	3.33%	1
d__Bacteria;p__Bacteroidota;c__Bacteroidia;o__Bacteroidales;f__Bacteroidaceae;g__Bacteroides;s__Bacteroides_fragilis	7	6	16	14	15	15	species	1	53.33%	50.00%	1
d__Bacteria;p__Proteobacteria;c__Gammaproteobacteria;o__Burkholderiales;f__Burkholderiaceae;g__Ralstonia;s__Ralstonia_pickettii	0	1	0	30	1	29	species	-1	0.00%	3.33%	1
d__Bacteria;p__Firmicutes;c__Bacilli;o__Lactobacillales;f__Streptococcaceae;g__Lactococcus;s__Lactococcus_lactis	2	1	2	28	1	29	species	1	6.67%	3.33%	1
d__Bacteria;p__Proteobacteria;c__Gammaproteobacteria;o__Burkholderiales;f__Burkholderiaceae;g__Burkholderia-Caballeronia-Paraburkholderia;s__Burkholderia_cepacia	1	0	1	29	0	30	species	1	3.33%	0.00%	1
d__Bacteria;p__Firmicutes;c__Clostridia;o__Clostridia_vadinBB60_group;f__Clostridia_vadinBB60_group;g__Clostridia_vadinBB60_group;s__metagenome	1	0	1	29	0	30	species	1	3.33%	0.00%	1
d__Bacteria;p__Bacteroidota;c__Bacteroidia;o__Bacteroidales;f__Prevotellaceae;g__Prevotella;s__Prevotella_timonensis	5	4	5	25	4	26	species	1	16.67%	13.33%	1
d__Bacteria;p__Bacteroidota;c__Bacteroidia;o__Bacteroidales;f__Bacteroidaceae;g__Bacteroides;s__Bacteroides_coprocola	5	6	8	22	9	21	species	-1	26.67%	30.00%	1
d__Bacteria;p__Firmicutes;c__Clostridia;o__Peptostreptococcales-Tissierellales;f__Peptostreptococcales-Tissierellales;g__Anaerococcus;s__Anaerococcus_prevotii	1	2	1	29	2	28	species	-1	3.33%	6.67%	1
d__Bacteria;p__Actinobacteriota;c__Coriobacteriia;o__Coriobacteriales;f__Coriobacteriaceae;g__Collinsella;s__Collinsella_sp.	2	3	2	28	3	27	species	-1	6.67%	10.00%	1
d__Archaea;p__Thermoplasmatota;c__Thermoplasmata;o__Methanomassiliicoccales;f__Methanomethylophilaceae;g__Candidatus_Methanomethylophilus;s__Candidatus_Methanomethylophilus	1	0	1	29	0	30	species	1	3.33%	0.00%	1
d__Bacteria;p__Desulfobacterota;c__Desulfovibrionia;o__Desulfovibrionales;f__Desulfovibrionaceae;g__Desulfovibrio;s__Desulfovibrio_piger	1	0	1	29	0	30	species	1	3.33%	0.00%	1
d__Bacteria;p__Proteobacteria;c__Gammaproteobacteria;o__Burkholderiales;f__Burkholderiaceae;g__Ralstonia;s__Ralstonia_sp.	1	0	1	29	0	30	species	1	3.33%	0.00%	1
d__Bacteria;p__Proteobacteria;c__Gammaproteobacteria;o__Burkholderiales;f__Burkholderiaceae;g__Cupriavidus;s__Ralstonia_sp.	0	1	0	30	1	29	species	-1	0.00%	3.33%	1
d__Bacteria;p__Firmicutes;c__Clostridia;o__Oscillospirales;f__Ruminococcaceae;g__Incertae_Sedis;s__metagenome	0	1	1	29	2	28	species	-1	3.33%	6.67%	1
d__Bacteria;p__Actinobacteriota;c__Actinobacteria;o__Bifidobacteriales;f__Bifidobacteriaceae;g__Bifidobacterium;s__Bifidobacterium_sp.	1	0	1	29	0	30	species	1	3.33%	0.00%	1
d__Bacteria;p__Firmicutes;c__Clostridia;o__Lachnospirales;f__Lachnospiraceae;g__Butyrivibrio;s__Butyrivibrio_crossotus	1	0	1	29	0	30	species	1	3.33%	0.00%	1
d__Bacteria;p__Bacteroidota;c__Bacteroidia;o__Bacteroidales;f__Tannerellaceae;g__Parabacteroides;s__Parabacteroides_faecis	1	0	1	29	0	30	species	1	3.33%	0.00%	1
d__Bacteria;p__Actinobacteriota;c__Actinobacteria;o__Micrococcales;f__Microbacteriaceae;g__Leucobacter;s__Leucobacter_tardus	1	0	1	29	0	30	species	1	3.33%	0.00%	1
d__Bacteria;p__Actinobacteriota;c__Actinobacteria;o__Actinomycetales;f__Actinomycetaceae;g__Arcanobacterium;s__Arcanobacterium_sp.	2	1	2	28	1	29	species	1	6.67%	3.33%	1
d__Bacteria;p__Fusobacteriota;c__Fusobacteriia;o__Fusobacteriales;f__Fusobacteriaceae;g__Fusobacterium;s__Fusobacterium_necrophorum	0	1	0	30	1	29	species	-1	0.00%	3.33%	1

d__Bacteria;p__Firmicutes;c__Bacilli;o__Lactobacillales;f__Streptococcaceae;g__Streptococcus;s__Streptococcus_anginosus	6	5	6	24	5	25	species	1	20.00%	16.67%	1
d__Bacteria;p__Proteobacteria;c__Gammaproteobacteria;o__Enterobacterales;f__Enterobacteriaceae;g__Escherichia-Shigella;s__Citrobacter_koseri	1	0	1	29	0	30	species	1	3.33%	0.00%	1
d__Bacteria;p__Proteobacteria;c__Gammaproteobacteria;o__Enterobacterales;f__Enterobacteriaceae;g__Escherichia-Shigella;s__Shigella_dysenteriae	8	7	10	20	9	21	species	1	33.33%	30.00%	1
d__Bacteria;p__Actinobacteriota;c__Actinobacteria;o__Corynebacteriales;f__Corynebacteriaceae;g__Corynebacterium;s__Corynebacterium_ureicelerivorans	2	1	2	28	1	29	species	1	6.67%	3.33%	1
d__Bacteria;p__Proteobacteria;c__Alphaproteobacteria;o__Rhizobiales;f__Rhizobiaceae;g__Allorhizobium-Neorhizobium-Pararhizobium-Rhizobium;s__Agrobacterium_radiobacter	1	0	1	29	0	30	species	1	3.33%	0.00%	1
d__Bacteria;p__Firmicutes;c__Negativicutes;o__Veillonellales-Selenomonadales;f__Veillonellaceae;g__Dialister;s__Dialister_succinatiphilus	0	1	1	29	2	28	species	-1	3.33%	6.67%	1
d__Bacteria;p__Proteobacteria;c__Gammaproteobacteria;o__Pseudomonadales;f__Pseudomonadaceae;g__Pseudomonas;s__Pseudomonas_fragi	1	2	1	29	2	28	species	-1	3.33%	6.67%	1
d__Bacteria;p__Bacteroidota;c__Bacteroidia;o__Bacteroidales;f__Bacteroidaceae;g__Bacteroides;s__bacterium_NLAE-zl-P297	6	7	21	9	22	8	species	-1	70.00%	73.33%	1
d__Bacteria;p__Firmicutes;c__Clostridia;o__Lachnospirales;f__Lachnospiraceae;g__[Eubacterium]_eligens_group;s__human_gut	2	1	4	26	3	27	species	1	13.33%	10.00%	1
d__Bacteria;p__Firmicutes;c__Bacilli;o__Bacillales;f__Bacillaceae;g__Oceanobacillus;s__Oceanobacillus_massiliensis	0	1	0	30	1	29	species	-1	0.00%	3.33%	1
d__Bacteria;p__Actinobacteriota;c__Coriobacteriia;o__Coriobacteriales;f__Atopobiaceae;g__Atopobium;s__Atopobium_minutum	3	2	3	27	2	28	species	1	10.00%	6.67%	1
d__Bacteria;p__Firmicutes;c__Bacilli;o__Bacillales;f__Bacillaceae;g__Oceanobacillus;s__Oceanobacillus_sojae	0	1	0	30	1	29	species	-1	0.00%	3.33%	1
d__Bacteria;p__Firmicutes;c__Clostridia;o__Peptostreptococcales-Tissierellales;f__Peptostreptococcales-Tissierellales;g__W5053;s__unidentified_marine	1	0	1	29	0	30	species	1	3.33%	0.00%	1
d__Bacteria;p__Firmicutes;c__Clostridia;o__Oscillospirales;f__Oscillospiraceae;g__Oscillibacter;s__Oscillibacter_valericigenes	0	1	0	30	1	29	species	-1	0.00%	3.33%	1
d__Bacteria;p__Actinobacteriota;c__Actinobacteria;o__Micrococcales;f__Micrococcaceae;g__Rothia;s__Rothia_mucilaginosa	0	1	0	30	1	29	species	-1	0.00%	3.33%	1
d__Bacteria;p__Proteobacteria;c__Gammaproteobacteria;o__Aeromonadales;f__Succinivibrionaceae;g__Succinatimonas;s__Succinatimonas_hippei	1	0	1	29	0	30	species	1	3.33%	0.00%	1
d__Bacteria;p__Bacteroidota;c__Bacteroidia;o__Bacteroidales;f__Barnesiellaceae;g__Barnesiella;s__Barnesiella_viscericola	0	1	0	30	1	29	species	-1	0.00%	3.33%	1
d__Bacteria;p__Actinobacteriota;c__Coriobacteriia;o__Coriobacteriales;f__Eggerthellaceae;g__Eggerthella;s__Eggerthella_timonensis	0	1	0	30	1	29	species	-1	0.00%	3.33%	1
d__Bacteria;p__Firmicutes;c__Bacilli;o__Lactobacillales;f__Lactobacillaceae;g__Lactobacillus;s__Lactobacillus_reuteri	2	1	2	28	1	29	species	1	6.67%	3.33%	1
d__Bacteria;p__Campilobacterota;c__Campylobacteria;o__Campylobacterales;f__Arcobacteraceae;g__Arcobacter;s__Arcobacter_trophiarum	0	1	0	30	1	29	species	-1	0.00%	3.33%	1
d__Archaea;p__Thermoplasmatota;c__Thermoplasmata;o__Methanomassiliicoccales;f__Methanomassiliicoccaceae;g__Methanomassiliicoccus;s__Candidatus_Methanomassiliicoccus	0	1	0	30	1	29	species	-1	0.00%	3.33%	1
d__Archaea;p__Euryarchaeota;c__Methanobacteria;o__Methanobacteriales;f__Methanobacteriaceae;g__Methanobrevibacter;s__Methanobrevibacter_smithii	5	4	5	25	4	26	species	1	16.67%	13.33%	1
d__Bacteria;p__Firmicutes;c__Bacilli;o__Lactobacillales;f__Lactobacillaceae;g__Lactobacillus;s__Lactobacillus_rhamnosus	0	1	0	30	1	29	species	-1	0.00%	3.33%	1
d__Bacteria;p__Bacteroidota;c__Bacteroidia;o__Bacteroidales;f__Bacteroidaceae;g__Bacteroides;s__Bacteroides_finegoldii	5	4	6	24	5	25	species	1	20.00%	16.67%	1
d__Bacteria;p__Actinobacteriota;c__Actinobacteria;o__Corynebacteriales;f__Corynebacteriaceae;g__Corynebacterium;s__Corynebacterium_pyruviciproducens	0	1	0	30	1	29	species	-1	0.00%	3.33%	1
d__Bacteria;p__Firmicutes;c__Bacilli;o__Erysipelotrichales;f__Erysipelotrichaceae;g__Catenisphaera;s__Chlamydia_trachomatis	2	1	2	28	1	29	species	1	6.67%	3.33%	1
d__Bacteria;p__Fusobacteriota;c__Fusobacteriia;o__Fusobacteriales;f__Fusobacteriaceae;g__Fusobacterium;s__Fusobacterium_necrogenes	1	0	1	29	0	30	species	1	3.33%	0.00%	1
d__Bacteria;p__Firmicutes;c__Clostridia;o__Oscillospirales;f__[Eubacterium]_coprostanoligenes_group;g__[Eubacterium]_coprostanoligenes_group;s__Clostridiales_bacterium	0	1	0	30	1	29	species	-1	0.00%	3.33%	1
d__Archaea;p__Thermoplasmatota;c__Thermoplasmata;o__Methanomassiliicoccales;f__Methanomethylophilaceae;s__methanogenic_archaeon	1	0	1	29	0	30	species	1	3.33%	0.00%	1
d__Bacteria;p__Actinobacteriota;c__Actinobacteria;o__Actinomycetales;f__Actinomycetaceae;g__Varibaculum;s__Varibaculum_sp.	1	0	1	29	0	30	species	1	3.33%	0.00%	1
d__Bacteria;p__Firmicutes;c__Bacilli;o__Paenibacillales;f__Paenibacillaceae;g__Paenibacillus;s__Paenibacillus_sp.	0	1	0	30	1	29	species	-1	0.00%	3.33%	1

d__Bacteria;p__Firmicutes;c__Clostridia;o__Oscillospirales;f__Ruminococcaceae;g__Incertae_Sedis;s__Clostridium_sp.	1	0	1	29	0	30	species	1	3.33%	0.00%	1
d__Bacteria;p__Actinobacteriota;c__Actinobacteria;o__Micrococcales;f__Micrococcaceae;g__Kocuria;s__Rothia_kristinae	0	1	0	30	1	29	species	-1	0.00%	3.33%	1
d__Bacteria;p__Actinobacteriota;c__Actinobacteria;o__Actinomycetales;f__Actinomycetaceae;g__Actinomyces;s__Actinomyces_bouchesdurhonensis	0	1	0	30	1	29	species	-1	0.00%	3.33%	1
d__Bacteria;p__Firmicutes;c__Clostridia;o__Peptostreptococcales-Tissierellales;f__Peptostreptococcaceae;g__Paeniclostridium;s__Paeniclostridium_ghonii	1	0	1	29	0	30	species	1	3.33%	0.00%	1
d__Archaea;p__Euryarchaeota;c__Methanobacteria;o__Methanobacteriales;f__Methanobacteriaceae;g__Methanobrevibacter;s__Methanobrevibacter_cuticularis	1	0	1	29	0	30	species	1	3.33%	0.00%	1
d__Bacteria;p__Firmicutes;c__Clostridia;o__Peptostreptococcales-Tissierellales;f__Peptostreptococcales-Tissierellales;g__Murdochiella;s__Murdochiella_asaccharolytica	3	2	3	27	2	28	species	1	10.00%	6.67%	1
d__Bacteria;p__Bacteroidota;c__Bacteroidia;o__Bacteroidales;f__Prevotellaceae;g__Prevotella;s__Prevotella_bergensis	0	1	0	30	1	29	species	-1	0.00%	3.33%	1
d__Bacteria;p__Bacteroidota;c__Bacteroidia;o__Bacteroidales;f__Bacteroidaceae;g__Bacteroides;s__human_gut	1	2	1	29	2	28	species	-1	3.33%	6.67%	1
d__Bacteria;p__Firmicutes;c__Bacilli;o__Erysipelotrichales;f__Erysipelatoclostridiaceae;g__Erysipelatoclostridiaceae;s__Firmicutes_bacterium	0	1	0	30	1	29	species	-1	0.00%	3.33%	1
d__Bacteria;p__Firmicutes;c__Clostridia;o__Clostridiales;f__Clostridiaceae;g__Clostridium_sensu_stricto_1;s__Clostridium_butyricum	1	2	1	29	2	28	species	-1	3.33%	6.67%	1
d__Bacteria;p__Fusobacteriota;c__Fusobacteriia;o__Fusobacteriales;f__Fusobacteriaceae;g__Fusobacterium;s__Fusobacterium_mortiferum	1	0	1	29	0	30	species	1	3.33%	0.00%	1
d__Bacteria;p__Firmicutes;c__Bacilli;o__Lactobacillales;f__Lactobacillaceae;g__Lactobacillus;s__Lactobacillus_plantarum	1	0	1	29	0	30	species	1	3.33%	0.00%	1
d__Bacteria;p__Actinobacteriota;c__Coriobacteriia;o__Coriobacteriales;f__Eggerthellaceae;g__Slackia;s__Slackia_exigua	1	0	1	29	0	30	species	1	3.33%	0.00%	1
d__Bacteria;p__Proteobacteria;c__Gammaproteobacteria;o__Burkholderiales;f__Sutterellaceae;g__Sutterella;s__metagenome	1	0	1	29	0	30	species	1	3.33%	0.00%	1
d__Bacteria;p__Proteobacteria;c__Gammaproteobacteria;o__Pasteurellales;f__Pasteurellaceae;g__Haemophilus;s__Haemophilus_sputorum	1	0	1	29	0	30	species	1	3.33%	0.00%	1
d__Archaea;p__Euryarchaeota;c__Methanobacteria;o__Methanobacteriales;f__Methanobacteriaceae;g__Methanosphaera;s__Methanosphaera_stadtmanae	1	0	1	29	0	30	species	1	3.33%	0.00%	1
d__Bacteria;p__Firmicutes;c__Bacilli;o__Lactobacillales;f__Lactobacillaceae;g__Lactobacillus;s__Lactobacillus_gasseri	1	2	1	29	2	28	species	-1	3.33%	6.67%	1
d__Bacteria;p__Firmicutes;c__Bacilli;o__Lactobacillales;f__Leuconostocaceae;g__Weissella;s__Weissella_viridescens	1	0	1	29	0	30	species	1	3.33%	0.00%	1
d__Bacteria;p__Firmicutes;c__Clostridia;o__Peptostreptococcales-Tissierellales;f__Peptostreptococcales-Tissierellales;g__Parvimonas;s__Parvimonas_micra	3	2	3	27	2	28	species	1	10.00%	6.67%	1
d__Bacteria;p__Firmicutes;c__Negativicutes;o__Veillonellales-Selenomonadales;f__Veillonellaceae;g__Anaeroglobus;s__Anaeroglobus_geminatus	0	1	0	30	1	29	species	-1	0.00%	3.33%	1
d__Bacteria;p__Firmicutes;c__Clostridia;o__Oscillospirales;f__Oscillospiraceae;g__UCG-005;s__gut_metagenome	0	1	0	30	1	29	species	-1	0.00%	3.33%	1
d__Bacteria;p__Firmicutes;c__Clostridia;o__Oscillospirales;f__[Eubacterium]_coprostanoligenes_group;g__[Eubacterium]_coprostanoligenes_group;s__unidentified	6	5	8	22	7	23	species	1	26.67%	23.33%	1
d__Bacteria;p__Firmicutes;c__Clostridia;o__Clostridia;f__Hungateiclostridiaceae;g__Fastidiosipila;s__Clostridiales_bacterium	1	0	1	29	0	30	species	1	3.33%	0.00%	1
d__Bacteria;p__Actinobacteriota;c__Actinobacteria;o__Actinomycetales;f__Actinomycetaceae;g__Actinomyces;s__Schaalia_cardiffensis	1	0	1	29	0	30	species	1	3.33%	0.00%	1
d__Bacteria;p__Firmicutes;c__Negativicutes;o__Veillonellales-Selenomonadales;f__Veillonellaceae;g__Dialister;s__Veillonellaceae_bacterium	0	1	0	30	1	29	species	-1	0.00%	3.33%	1
d__Bacteria;p__Firmicutes;c__Negativicutes;o__Veillonellales-Selenomonadales;f__Veillonellaceae;g__Negativicoccus;s__Negativicoccus_succinicivorans	0	1	0	30	1	29	species	-1	0.00%	3.33%	1
d__Bacteria;p__Proteobacteria;c__Alphaproteobacteria;o__Sphingomonadales;f__Sphingomonadaceae;g__Sphingomonas;s__Sphingomonas_prati	0	1	0	30	1	29	species	-1	0.00%	3.33%	1
d__Bacteria;p__Firmicutes;c__Clostridia;o__Lachnospirales;f__Lachnospiraceae;g__Howardella;s__Catonella_sp.	1	0	1	29	0	30	species	1	3.33%	0.00%	1
d__Bacteria;p__Firmicutes;c__Clostridia;o__Christensenellales;f__Christensenellaceae;g__Christensenellaceae;s__Christensenella_sp.	1	2	1	29	2	28	species	-1	3.33%	6.67%	1
d__Bacteria;p__Bacteroidota;c__Bacteroidia;o__Bacteroidales;f__Bacteroidaceae;g__Bacteroides;s__Bacteroides_cellulosilyticus	7	6	15	15	14	16	species	1	50.00%	46.67%	1
d__Bacteria;p__Firmicutes;c__Bacilli;o__Bacillales;f__Planococcaceae;g__Lysinibacillus;s__Lysinibacillus_sphaericus	1	0	1	29	0	30	species	1	3.33%	0.00%	1

d__Bacteria;p__Firmicutes;c__Bacilli;o__Bacillales;f__Planococcaceae;g__Lysinibacillus;s__Bacillus_sp.	1	0	1	29	0	30	species	1	3.33%	0.00%	1
d__Bacteria;p__Firmicutes;c__Clostridia;o__Peptostreptococcales-Tissierellales;f__Peptostreptococcales-Tissierellales;g__Anaerococcus;s__Anaerococcus_sp.	0	1	0	30	1	29	species	-1	0.00%	3.33%	1
d__Bacteria;p__Bacteroidota;c__Bacteroidia;o__Bacteroidales;f__Tannerellaceae;g__Parabacteroides;s__Blattella_germanica	1	0	1	29	0	30	species	1	3.33%	0.00%	1
d__Bacteria;p__Firmicutes;c__Bacilli;o__Lactobacillales;f__Aerococcaceae;g__Aerococcus;s__Aerococcus_christensenii	0	1	0	30	1	29	species	-1	0.00%	3.33%	1
d__Bacteria;p__Firmicutes;c__Clostridia;o__Peptostreptococcales-Tissierellales;f__Anaerovoracaceae;g__S5-A14a;s__Peptostreptococcaceae_bacterium	1	0	1	29	0	30	species	1	3.33%	0.00%	1
d__Bacteria;p__Bacteroidota;c__Bacteroidia;o__Bacteroidales;f__Bacteroidaceae;g__Bacteroides;s__Bacteroides_uniformis	1	2	28	2	29	1	species	-1	93.33%	96.67%	1
d__Bacteria;p__Firmicutes;c__Bacilli;o__Lactobacillales;f__Streptococcaceae;g__Streptococcus;s__Streptococcus_gordonii	1	0	1	29	0	30	species	1	3.33%	0.00%	1
d__Bacteria;p__Proteobacteria;c__Gammaproteobacteria;o__Alteromonadales;f__Alteromonadaceae;g__Rheinheimera;s__gamma_proteobacterium	0	1	0	30	1	29	species	-1	0.00%	3.33%	1
d__Bacteria;p__Proteobacteria;c__Gammaproteobacteria;o__Pseudomonadales;f__Moraxellaceae;g__Acinetobacter;s__Acinetobacter_guillouiae	0	1	0	30	1	29	species	-1	0.00%	3.33%	1
d__Bacteria;p__Actinobacteriota;c__Actinobacteria;o__Corynebacteriales;f__Corynebacteriaceae;g__Corynebacterium;s__Corynebacterium_sundsvallense	1	0	1	29	0	30	species	1	3.33%	0.00%	1
d__Bacteria;p__Actinobacteriota;c__Actinobacteria;o__Bifidobacteriales;f__Bifidobacteriaceae;g__Alloscardovia;s__Alloscardovia_omnicolens	0	1	0	30	1	29	species	-1	0.00%	3.33%	1
d__Bacteria;p__Actinobacteriota;c__Actinobacteria;o__Corynebacteriales;f__Corynebacteriaceae;g__Corynebacterium;s__Corynebacterium_variabile	0	1	0	30	1	29	species	-1	0.00%	3.33%	1
d__Bacteria;p__Firmicutes;c__Clostridia;o__Oscillospirales;f__Oscillospiraceae;g__Intestinimonas;s__Intestinimonas_timonensis	3	2	3	27	2	28	species	1	10.00%	6.67%	1
d__Bacteria;p__Proteobacteria;c__Gammaproteobacteria;o__Enterobacterales;f__Yersiniaceae;g__Serratia;s__Serratia_fonticola	1	2	1	29	2	28	species	-1	3.33%	6.67%	1
d__Bacteria;p__Bacteroidota;c__Bacteroidia;o__Bacteroidales;f__Porphyromonadaceae;g__Porphyromonas;s__Porphyromonas_uenonis	0	1	0	30	1	29	species	-1	0.00%	3.33%	1
d__Bacteria;p__Firmicutes;c__Bacilli;o__Lactobacillales;f__Lactobacillaceae;g__Lactobacillus;s__Lactobacillus_colini	2	1	2	28	1	29	species	1	6.67%	3.33%	1
d__Bacteria;p__Firmicutes;c__Clostridia;o__Oscillospirales;f__Oscillospiraceae;s__Intestinimonas_massiliensis	0	1	0	30	1	29	species	-1	0.00%	3.33%	1
d__Bacteria;p__Firmicutes;c__Clostridia;o__Lachnospirales;f__Lachnospiraceae;g__Lachnoclostridium;s__Mordavella_sp.	4	5	4	26	5	25	species	-1	13.33%	16.67%	1
d__Bacteria;p__Firmicutes;c__Bacilli;o__Lactobacillales;f__Lactobacillaceae;g__Lactobacillus;s__Lactobacillus_sakei	3	2	3	27	2	28	species	1	10.00%	6.67%	1
d__Bacteria;p__Proteobacteria;c__Gammaproteobacteria;o__Pseudomonadales;f__Pseudomonadaceae;g__Pseudomonas;s__Pseudomonas_sp.	1	0	1	29	0	30	species	1	3.33%	0.00%	1
d__Bacteria;p__Desulfobacterota;c__Desulfovibrionia;o__Desulfovibrionales;f__Desulfovibrionaceae;g__Desulfovibrio;s__Desulfovibrio_fairfieldensis	3	2	3	27	2	28	species	1	10.00%	6.67%	1
d__Bacteria;p__Firmicutes;c__Clostridia;o__Lachnospirales;f__Lachnospiraceae;g__[Eubacterium]_fissicatena_group;s__Faecalicatena_fissicatena	1	0	1	29	0	30	species	1	3.33%	0.00%	1
d__Bacteria;p__Firmicutes;c__Bacilli;o__Erysipelotrichales;f__Erysipelotrichaceae;g__Solobacterium;s__Solobacterium_moorei	3	2	3	27	2	28	species	1	10.00%	6.67%	1
d__Bacteria;p__Bacteroidota;c__Bacteroidia;o__Bacteroidales;f__Tannerellaceae;g__Parabacteroides;s__Monocercomonoides_sp.	2	1	2	28	1	29	species	1	6.67%	3.33%	1
d__Bacteria;p__Firmicutes;c__Clostridia;o__Clostridiales;f__Clostridiaceae;g__Clostridium_sensu_stricto_1;s__Ruminococcus_sp.	2	1	3	27	2	28	species	1	10.00%	6.67%	1
d__Bacteria;p__Firmicutes;c__Clostridia;o__Lachnospirales;f__Lachnospiraceae;g__[Ruminococcus]_gauvreauii_group;s__bacterium_ic1296	1	0	1	29	0	30	species	1	3.33%	0.00%	1
d__Bacteria;p__Firmicutes;c__Bacilli;o__Bacillales;f__Bacillaceae;g__Oceanobacillus;s__bacterium_K29	0	1	0	30	1	29	species	-1	0.00%	3.33%	1
d__Bacteria;p__Actinobacteriota;c__Coriobacteriia;o__Coriobacteriales;f__Coriobacteriaceae;g__Enorma;s__Collinsella_massiliensis	1	0	1	29	0	30	species	1	3.33%	0.00%	1
d__Bacteria;p__Firmicutes;c__Bacilli;o__Bacillales;f__Bacillaceae;g__Bacillus;s__Bacillus_simplex	0	1	0	30	1	29	species	-1	0.00%	3.33%	1
d__Bacteria;p__Bacteroidota;c__Bacteroidia;o__Bacteroidales;f__Porphyromonadaceae;g__Porphyromonas;s__Porphyromonas_asaccharolytica	4	3	4	26	3	27	species	1	13.33%	10.00%	1
d__Bacteria;p__Proteobacteria;c__Gammaproteobacteria;o__Aeromonadales;f__Aeromonadaceae;g__Aeromonas;s__Aeromonas_hydrophila	1	2	1	29	2	28	species	-1	3.33%	6.67%	1

d__Bacteria;p__Firmicutes;c__Bacilli;o__Lactobacillales;f__Streptococcaceae;g__Streptococcus;s__Streptococcus_agalactiae	1	0	1	29	0	30	species	1	3.33%	0.00%	1
d__Bacteria;p__Firmicutes;c__Bacilli;o__Lactobacillales;f__Aerococcaceae;g__Eremococcus;s__Eremococcus_coleocola	1	0	1	29	0	30	species	1	3.33%	0.00%	1
d__Bacteria;p__Firmicutes;c__Clostridia;o__Lachnospirales;f__Lachnospiraceae;s__Ruminococcus_sp.	1	0	1	29	0	30	species	1	3.33%	0.00%	1
d__Bacteria;p__Bacteroidota;c__Bacteroidia;o__Bacteroidales;f__Muribaculaceae;g__Muribaculaceae;s__Porphyromonadaceae_bacterium	0	1	0	30	1	29	species	-1	0.00%	3.33%	1
d__Bacteria;p__Proteobacteria;c__Gammaproteobacteria;o__Pasteurellales;f__Pasteurellaceae;g__Actinobacillus;s__Haemophilus_parahaemolyticus	1	0	1	29	0	30	species	1	3.33%	0.00%	1
d__Bacteria;p__Proteobacteria;c__Gammaproteobacteria;o__Xanthomonadales;f__Xanthomonadaceae;g__Stenotrophomonas;s__gamma_proteobacterium	1	0	1	29	0	30	species	1	3.33%	0.00%	1
d__Bacteria;p__Bacteroidota;c__Bacteroidia;o__Bacteroidales;f__Bacteroidaceae;g__Bacteroides;s__bacterium_NLAE-zl-C233	11	10	16	14	15	15	species	1	53.33%	50.00%	1
d__Archaea;p__Thermoplasmatota;c__Thermoplasmata;o__Methanomassiliicoccales;f__Methanomassiliicoccaceae;g__Methanomassiliicoccus;s__Methanomassiliicoccus_luminyensis	1	0	1	29	0	30	species	1	3.33%	0.00%	1
d__Bacteria;p__Proteobacteria;c__Gammaproteobacteria;o__Enterobacterales;f__Morganellaceae;g__Providencia;s__Providencia_vermicola	0	1	0	30	1	29	species	-1	0.00%	3.33%	1
d__Bacteria;p__Proteobacteria;c__Gammaproteobacteria;o__Pseudomonadales;f__Pseudomonadaceae;g__Pseudomonas;s__bacterium_enrichment	2	1	2	28	1	29	species	1	6.67%	3.33%	1
d__Bacteria;p__Proteobacteria;c__Alphaproteobacteria;o__Sphingomonadales;f__Sphingomonadaceae;g__Sphingobium;s__bacterium_YCG12	1	0	1	29	0	30	species	1	3.33%	0.00%	1
d__Bacteria;p__Proteobacteria;c__Gammaproteobacteria;o__Pseudomonadales;f__Moraxellaceae;g__Acinetobacter;s__Acinetobacter_bereziniae	0	1	0	30	1	29	species	-1	0.00%	3.33%	1
d__Bacteria;p__Bacteroidota;c__Bacteroidia;o__Bacteroidales;f__Bacteroidaceae;g__Bacteroides;s__Mogibacterium_sp.	0	1	0	30	1	29	species	-1	0.00%	3.33%	1
d__Bacteria;p__Firmicutes;c__Bacilli;o__Lactobacillales;f__Lactobacillaceae;g__Lactobacillus;s__Lactobacillus_iners	1	0	1	29	0	30	species	1	3.33%	0.00%	1
d__Bacteria;p__Proteobacteria;c__Gammaproteobacteria;o__Pseudomonadales;f__Pseudomonadaceae;g__Pseudomonas;s__Pseudomonas_guangdongensis	0	1	0	30	1	29	species	-1	0.00%	3.33%	1
d__Bacteria;p__Firmicutes;c__Bacilli;o__Lactobacillales;f__Streptococcaceae;g__Streptococcus;s__Streptococcus_peroris	4	5	6	24	7	23	species	-1	20.00%	23.33%	1
d__Bacteria;p__Proteobacteria;c__Gammaproteobacteria;o__Pseudomonadales;f__Pseudomonadaceae;g__Pseudomonas;s__Pseudomonas_chlororaphis	0	1	0	30	1	29	species	-1	0.00%	3.33%	1
d__Bacteria;p__Actinobacteriota;c__Actinobacteria;o__Corynebacteriales;f__Corynebacteriaceae;g__Corynebacterium;s__Corynebacterium_amycolatum	1	0	1	29	0	30	species	1	3.33%	0.00%	1
d__Bacteria;p__Bacteroidota;c__Bacteroidia;o__Bacteroidales;f__Bacteroidaceae;g__Bacteroides;s__Bacteroides_sp.	4	3	4	26	3	27	species	1	13.33%	10.00%	1
d__Bacteria;p__Firmicutes;c__Clostridia;o__Lachnospirales;f__Lachnospiraceae;g__Blautia;s__Blautia_sp.	8	7	9	21	8	22	species	1	30.00%	26.67%	1
d__Bacteria;p__Firmicutes;c__Clostridia;o__Oscillospirales;f__Ruminococcaceae;g__Angelakisella;s__Ruminococcus_sp.	2	3	2	28	3	27	species	-1	6.67%	10.00%	1
d__Bacteria;p__Proteobacteria;c__Gammaproteobacteria;o__Pseudomonadales;f__Pseudomonadaceae;g__Pseudomonas;s__metagenome	1	0	1	29	0	30	species	1	3.33%	0.00%	1
d__Bacteria;p__Proteobacteria;c__Alphaproteobacteria;o__Rhodospirillales;s__Azospirillum_sp.	1	2	1	29	2	28	species	-1	3.33%	6.67%	1
d__Bacteria;p__Firmicutes;c__Clostridia;o__Peptostreptococcales-Tissierellales;f__Anaerovoracaceae;g__Mogibacterium;s__Mogibacterium_sp.	1	0	1	29	0	30	species	1	3.33%	0.00%	1
d__Bacteria;p__Firmicutes;c__Bacilli;o__Lactobacillales;f__Leuconostocaceae;g__Leuconostoc;s__Leuconostoc_gelidum	1	0	1	29	0	30	species	1	3.33%	0.00%	1
d__Bacteria;p__Actinobacteriota;c__Coriobacteriia;o__Coriobacteriales;f__Eggerthellaceae;g__Enteroscipio;s__Enteroscipio_rubneri	1	0	1	29	0	30	species	1	3.33%	0.00%	1
d__Bacteria;p__Actinobacteriota;c__Actinobacteria;o__Bifidobacteriales;f__Bifidobacteriaceae;g__Bifidobacterium;s__Bifidobacterium_pseudolongum	1	0	1	29	0	30	species	1	3.33%	0.00%	1
d__Bacteria;p__Firmicutes;c__Bacilli;o__Bacillales;f__Planococcaceae;g__Psychrobacillus;s__Psychrobacillus_psychrodurans	0	1	0	30	1	29	species	-1	0.00%	3.33%	1
d__Bacteria;p__Proteobacteria;c__Gammaproteobacteria;o__Burkholderiales;f__Sutterellaceae;g__Sutterella;s__Burkholderia_sp.	1	0	1	29	0	30	species	1	3.33%	0.00%	1
d__Bacteria;p__Actinobacteriota;c__Actinobacteria;o__Actinomycetales;f__Actinomycetaceae;g__Actinomyces;s__Winkia_neuii	2	1	2	28	1	29	species	1	6.67%	3.33%	1
d__Bacteria;p__Proteobacteria;c__Gammaproteobacteria;o__Pseudomonadales;f__Pseudomonadaceae;g__Pseudomonas;s__Pseudomonas_japonica	1	0	1	29	0	30	species	1	3.33%	0.00%	1

d__Bacteria;p__Bacteroidota;c__Bacteroidia;o__Bacteroidales;f__Rikenellaceae;g__Alistipes;s__Alistipes_shahii	6	7	14	16	15	15	species	-1	46.67%	50.00%	1
d__Bacteria;p__Proteobacteria;c__Gammaproteobacteria;o__Enterobacterales;f__Enterobacteriaceae;g__Escherichia-Shigella;s__Shigella_flexneri	9	10	12	18	13	17	species	-1	40.00%	43.33%	1
d__Bacteria;p__Firmicutes;c__Clostridia;o__Lachnospirales;f__Lachnospiraceae;s__Lachnospiraceae_bacterium	2	3	3	27	4	26	species	-1	10.00%	13.33%	1
d__Bacteria;p__Firmicutes;c__Clostridia;o__Oscillospirales;f__Oscillospiraceae;s__Clostridium_sp.	4	5	22	8	23	7	species	-1	73.33%	76.67%	1
d__Bacteria;p__Proteobacteria;c__Gammaproteobacteria;o__Pasteurellales;f__Pasteurellaceae;g__Aggregatibacter;s__Aggregatibacter_aphrophilus	1	0	1	29	0	30	species	1	3.33%	0.00%	1
d__Bacteria;p__Bacteroidota;c__Bacteroidia;o__Bacteroidales;f__Rikenellaceae;g__Alistipes;s__Alistipes_indistinctus	8	7	13	17	12	18	species	1	43.33%	40.00%	1
d__Bacteria;p__Bacteroidota;c__Bacteroidia;o__Bacteroidales;f__Bacteroidaceae;g__Bacteroides;s__Bacteroides_stercorirosoris	0	1	0	30	1	29	species	-1	0.00%	3.33%	1
d__Bacteria;p__Proteobacteria;c__Gammaproteobacteria;o__Burkholderiales;f__Comamonadaceae;g__Aquabacterium;s__Aquabacterium_parvum	0	1	0	30	1	29	species	-1	0.00%	3.33%	1
d__Bacteria;p__Actinobacteriota;c__Actinobacteria;o__Bifidobacteriales;f__Bifidobacteriaceae;g__Bifidobacterium;s__Bifidobacterium_indicum	1	0	1	29	0	30	species	1	3.33%	0.00%	1
d__Bacteria;p__Firmicutes;c__Bacilli;o__Lactobacillales;f__Lactobacillaceae;g__Lactobacillus;s__Lactobacillus_mucosae	1	2	1	29	2	28	species	-1	3.33%	6.67%	1
d__Bacteria;p__Proteobacteria;c__Alphaproteobacteria;o__Rhodospirillales;s__gut_metagenome	4	5	9	21	10	20	species	-1	30.00%	33.33%	1
d__Bacteria;p__Firmicutes;c__Clostridia;o__Oscillospirales;f__Ruminococcaceae;g__Faecalibacterium;s__Faecalibacterium_prausnitzii	7	6	22	8	21	9	species	1	73.33%	70.00%	1
d__Bacteria;p__Firmicutes;c__Bacilli;o__Erysipelotrichales;f__Erysipelatoclostridiaceae;g__Erysipelatoclostridium;s__Clostridiales_bacterium	0	1	0	30	1	29	species	-1	0.00%	3.33%	1
d__Bacteria;p__Firmicutes;c__Negativicutes;o__Veillonellales-Selenomonadales;f__Veillonellaceae;g__Veillonella;s__Veillonella_parvula	2	1	2	28	1	29	species	1	6.67%	3.33%	1
d__Bacteria;p__Actinobacteriota;c__Actinobacteria;o__Bifidobacteriales;f__Bifidobacteriaceae;g__Gardnerella;s__Gardnerella_vaginalis	0	1	0	30	1	29	species	-1	0.00%	3.33%	1
d__Bacteria;p__Proteobacteria;c__Gammaproteobacteria;o__Burkholderiales;f__Comamonadaceae;g__Comamonas;s__Comamonas_kerstersii	1	2	1	29	2	28	species	-1	3.33%	6.67%	1
d__Bacteria;p__Firmicutes;c__Clostridia;o__Oscillospirales;f__UCG-010;g__UCG-010;s__metagenome	6	5	8	22	7	23	species	1	26.67%	23.33%	1
d__Bacteria;p__Firmicutes;c__Bacilli;o__Lactobacillales;f__Lactobacillaceae;g__Lactobacillus;s__Lactobacillus_casei	0	1	0	30	1	29	species	-1	0.00%	3.33%	1
d__Bacteria;p__Bacteroidota;c__Bacteroidia;o__Bacteroidales;f__Bacteroidaceae;g__Bacteroides;s__Bacteroides_nordii	8	7	8	22	7	23	species	1	26.67%	23.33%	1
d__Bacteria;p__Proteobacteria;c__Gammaproteobacteria;o__Burkholderiales;f__Burkholderiaceae;g__Burkholderia-Caballeronia-Paraburkholderia;s__Burkholderia_mallei	1	0	1	29	0	30	species	1	3.33%	0.00%	1
d__Bacteria;p__Fusobacteriota;c__Fusobacteriia;o__Fusobacteriales;f__Fusobacteriaceae;g__Fusobacterium;s__rumen_bacterium	0	1	0	30	1	29	species	-1	0.00%	3.33%	1
d__Bacteria;p__Firmicutes;c__Bacilli;o__Lactobacillales;f__Streptococcaceae;g__Lactococcus;s__Lactococcus_garvieae	1	2	1	29	2	28	species	-1	3.33%	6.67%	1
d__Bacteria;p__Firmicutes;c__Clostridia;o__Lachnospirales;f__Lachnospiraceae;g__Anaerostipes;s__Anaerostipes_butyraticus	1	0	1	29	0	30	species	1	3.33%	0.00%	1
d__Bacteria;p__Actinobacteriota;c__Actinobacteria;o__Corynebacteriales;f__Corynebacteriaceae;g__Corynebacterium;s__Corynebacterium_genitalium	1	0	1	29	0	30	species	1	3.33%	0.00%	1
d__Bacteria;p__Proteobacteria;c__Gammaproteobacteria;o__Burkholderiales;f__Comamonadaceae;g__Comamonas;s__bacterium_SM2-6	0	1	0	30	1	29	species	-1	0.00%	3.33%	1
d__Bacteria;p__Firmicutes;c__Clostridia;o__Oscillospirales;f__Butyricicoccaceae;g__Butyricicoccus;s__Agathobaculum_butyriciproducens	1	0	1	29	0	30	species	1	3.33%	0.00%	1
d__Bacteria;p__Firmicutes;c__Negativicutes;o__Veillonellales-Selenomonadales;f__Veillonellaceae;g__Negativicoccus;s__Negativicoccus_massiliensis	1	0	1	29	0	30	species	1	3.33%	0.00%	1
d__Bacteria;p__Proteobacteria;c__Gammaproteobacteria;o__Xanthomonadales;f__Xanthomonadaceae;g__Stenotrophomonas;s__Stenotrophomonas_sp.	1	0	1	29	0	30	species	1	3.33%	0.00%	1
d__Bacteria;p__Firmicutes;c__Clostridia;o__Oscillospirales;f__Ruminococcaceae;g__Candidatus_Soleaferrea;s__Candidatus_Soleaferrea	1	0	1	29	0	30	species	1	3.33%	0.00%	1
d__Bacteria;p__Actinobacteriota;c__Actinobacteria;o__Corynebacteriales;f__Corynebacteriaceae;g__Corynebacterium;s__Corynebacterium_riegelii	1	0	1	29	0	30	species	1	3.33%	0.00%	1
d__Bacteria;p__Bacteroidota;c__Bacteroidia;o__Bacteroidales;f__Prevotellaceae;g__Alloprevotella;s__gut_metagenome	0	1	0	30	1	29	species	-1	0.00%	3.33%	1

d__Bacteria;p__Bacteroidota;c__Bacteroidia;o__Bacteroidales;f__Bacteroidaceae;g__Bacteroides;s__Bacteroides_eggerthii	3	2	10	20	9	21	species	1	33.33%	30.00%	1
d__Bacteria;p__Proteobacteria;c__Gammaproteobacteria;o__Pseudomonadales;f__Pseudomonadaceae;g__Pseudomonas;s__Pseudomonas_otitidis	0	1	0	30	1	29	species	-1	0.00%	3.33%	1
d__Bacteria;p__Proteobacteria;c__Gammaproteobacteria;o__Pseudomonadales;f__Moraxellaceae;g__Acinetobacter;s__Acinetobacter_sp.	0	1	0	30	1	29	species	-1	0.00%	3.33%	1
d__Bacteria;p__Firmicutes;c__Bacilli;o__Lactobacillales;f__Leuconostocaceae;g__Weissella;s__Weissella_paramesenteroides	0	1	0	30	1	29	species	-1	0.00%	3.33%	1
d__Bacteria;p__Campilobacterota;c__Campylobacteria;o__Campylobacterales;f__Campylobacteraceae;g__Campylobacter;s__Campylobacter_concisus	1	0	1	29	0	30	species	1	3.33%	0.00%	1
d__Bacteria;p__Proteobacteria;c__Alphaproteobacteria;o__Sphingomonadales;f__Sphingomonadaceae;g__Sphingomonas;s__Sphingomonas_panacis	1	0	1	29	0	30	species	1	3.33%	0.00%	1
d__Bacteria;p__Proteobacteria;c__Alphaproteobacteria;o__Sphingomonadales;f__Sphingomonadaceae;g__Sphingobium;s__Sphingobium_yanoikuyae	1	2	1	29	2	28	species	-1	3.33%	6.67%	1
d__Bacteria;p__Firmicutes;c__Clostridia;o__Lachnospirales;f__Lachnospiraceae;g__Blautia;s__Blautia_hansenii	7	6	11	19	10	20	species	1	36.67%	33.33%	1
d__Bacteria;p__Proteobacteria;c__Gammaproteobacteria;o__Pseudomonadales;f__Moraxellaceae;g__Acinetobacter;s__Acinetobacter_baumannii	0	1	0	30	1	29	species	-1	0.00%	3.33%	1
d__Bacteria;p__Fusobacteriota;c__Fusobacteriia;o__Fusobacteriales;f__Fusobacteriaceae;g__Fusobacterium;s__bacterium_NLAE-zl-G149	0	1	0	30	1	29	species	-1	0.00%	3.33%	1
d__Bacteria;p__Bacteroidota;c__Bacteroidia;o__Bacteroidales;f__Bacteroidaceae;g__Bacteroides;s__bacterium_NLAE-zl-P803	1	0	30	0	29	1	species	1	100.0%	96.67%	1
d__Bacteria;p__Bacteroidota;c__Bacteroidia;o__Bacteroidales;f__Bacteroidaceae;g__Bacteroides;s__bacterium_New	2	1	2	28	1	29	species	1	6.67%	3.33%	1
d__Bacteria;p__Proteobacteria;c__Gammaproteobacteria;o__Xanthomonadales;f__Xanthomonadaceae;g__Pseudoxanthomonas;s__Pseudoxanthomonas_mexicana	1	0	1	29	0	30	species	1	3.33%	0.00%	1
d__Bacteria;p__Bacteroidota;c__Bacteroidia;o__Flavobacteriales;f__Flavobacteriaceae;g__Flavobacterium;s__Flavobacteriia_bacterium	0	1	0	30	1	29	species	-1	0.00%	3.33%	1
d__Bacteria;p__Proteobacteria;c__Gammaproteobacteria;o__Enterobacterales;f__Enterobacteriaceae;g__Escherichia-Shigella;s__Escherichia_albertii	0	1	0	30	1	29	species	-1	0.00%	3.33%	1
d__Bacteria;p__Firmicutes;c__Clostridia;o__Oscillospirales;f__Ruminococcaceae;g__UBA1819;s__Ruminococcaceae_bacterium	0	1	0	30	1	29	species	-1	0.00%	3.33%	1
d__Bacteria;p__Firmicutes;c__Clostridia;o__Oscillospirales;f__Ruminococcaceae;s__metagenome	4	5	24	6	25	5	species	-1	80.00%	83.33%	1
d__Bacteria;p__Firmicutes;c__Bacilli;o__Bacillales;f__Planococcaceae;g__Lysinibacillus;s__Lysinibacillus_sp.	0	1	0	30	1	29	species	-1	0.00%	3.33%	1
d__Bacteria;p__Firmicutes;c__Bacilli;o__Bacillales;f__Bacillaceae;g__Bacillus;s__Bacillus_clausii	0	1	0	30	1	29	species	-1	0.00%	3.33%	1
d__Bacteria;p__Proteobacteria;c__Alphaproteobacteria;o__Caulobacterales;f__Caulobacteraceae;g__Brevundimonas;s__Alphaproteobacteria_bacterium	0	1	0	30	1	29	species	-1	0.00%	3.33%	1
d__Bacteria;p__Firmicutes;c__Bacilli;o__Bacillales;f__Bacillaceae;g__Oceanobacillus;s__Oceanobacillus_oncorhynchi	0	1	0	30	1	29	species	-1	0.00%	3.33%	1
d__Bacteria;p__Firmicutes;c__Clostridia;o__Lachnospirales;f__Lachnospiraceae;g__Lachnoclostridium;s__bacterium_NLAE-zl-H75	1	0	1	29	0	30	species	1	3.33%	0.00%	1
d__Bacteria;p__Proteobacteria;c__Gammaproteobacteria;o__Pseudomonadales;f__Moraxellaceae;g__Acinetobacter;s__Acinetobacter_junii	0	1	0	30	1	29	species	-1	0.00%	3.33%	1
d__Bacteria;p__Firmicutes;c__Bacilli;o__Lactobacillales;f__Aerococcaceae;g__Ignavigranum;s__Ignavigranum_ruoffiae	1	0	1	29	0	30	species	1	3.33%	0.00%	1
d__Bacteria;p__Proteobacteria;c__Gammaproteobacteria;o__Burkholderiales;f__Sutterellaceae;g__Parasutterella;s__Parasutterella_secunda	2	3	2	28	3	27	species	-1	6.67%	10.00%	1
d__Bacteria;p__Proteobacteria;c__Gammaproteobacteria;o__Burkholderiales;f__Sutterellaceae;g__Sutterella;s__Sutterella_parvirubra	1	0	1	29	0	30	species	1	3.33%	0.00%	1
d__Bacteria;p__Firmicutes;c__Bacilli;o__Bacillales;f__Bacillaceae;g__Oceanobacillus;s__Oceanobacillus_profundus	0	1	0	30	1	29	species	-1	0.00%	3.33%	1
d__Bacteria;p__Firmicutes;c__Negativicutes;o__Acidaminococcales;f__Acidaminococcaceae;g__Acidaminococcus;s__Acidaminococcus_fermentans	1	0	1	29	0	30	species	1	3.33%	0.00%	1
d__Bacteria;p__Firmicutes;c__Bacilli;o__Bacillales;f__Planococcaceae;g__[Renibacterium]_salmoninarum_group;s__Renibacterium_salmoninarum	0	1	0	30	1	29	species	-1	0.00%	3.33%	1
d__Bacteria;p__Firmicutes;c__Bacilli;o__Lactobacillales;f__Streptococcaceae;g__Streptococcus;s__Streptococcus_infantarius	0	1	0	30	1	29	species	-1	0.00%	3.33%	1
d__Bacteria;p__Proteobacteria;c__Gammaproteobacteria;o__Burkholderiales;f__Sutterellaceae;g__Sutterella;s__gut_metagenome	3	4	4	26	5	25	species	-1	13.33%	16.67%	1

d__Bacteria;p__Firmicutes;c__Clostridia;o__Clostridiales;f__Clostridiaceae;g__Clostridium_sensu_stricto_1;s__Clostridium_tertium	0	1	0	30	1	29	species	-1	0.00%	3.33%	1
d__Bacteria;p__Bacteroidota;c__Bacteroidia;o__Bacteroidales;f__Porphyromonadaceae;g__Porphyromonas;s__Porphyromonas_bennonis	1	0	1	29	0	30	species	1	3.33%	0.00%	1
d__Bacteria;p__Bacteroidota;c__Bacteroidia;o__Bacteroidales;f__Barnesiellaceae;g__Barnesiella;s__Barnesiella_intestinihominis	1	0	1	29	0	30	species	1	3.33%	0.00%	1
d__Bacteria;p__Firmicutes;c__Clostridia;o__Eubacteriales;f__Garciellaceae;g__Irregularibacter;s__Irregularibacter_muris	0	1	0	30	1	29	species	-1	0.00%	3.33%	1
d__Bacteria;p__Firmicutes;c__Clostridia;o__Lachnospirales;f__Lachnospiraceae;g__[Eubacterium]_fissicatena_group;s__Clostridium_sp.	2	1	2	28	1	29	species	1	6.67%	3.33%	1
d__Bacteria;p__Firmicutes;c__Clostridia;o__Lachnospirales;f__Lachnospiraceae;g__Roseburia;s__Roseburia_hominis	6	7	7	23	8	22	species	-1	23.33%	26.67%	1
d__Bacteria;p__Actinobacteriota;c__Actinobacteria;o__Streptomycetales;f__Streptomycetaceae;g__Streptomyces;s__Streptomyces_atrovirens	1	0	1	29	0	30	species	1	3.33%	0.00%	1
d__Bacteria;p__Fusobacteriota;c__Fusobacteriia;o__Fusobacteriales;f__Fusobacteriaceae;g__Fusobacterium;s__Fusobacterium_ulcerans	0	1	0	30	1	29	species	-1	0.00%	3.33%	1
d__Bacteria;p__Firmicutes;c__Clostridia;o__Peptostreptococcales-Tissierellales;f__Anaerovoracaceae;g__[Eubacterium]_nodatum_group;s__Ihubacter_massiliensis	5	6	5	25	6	24	species	-1	16.67%	20.00%	1
d__Bacteria;p__Firmicutes;c__Clostridia;o__Lachnospirales;f__Lachnospiraceae;g__Lachnoclostridium;s__unidentified	1	2	1	29	2	28	species	-1	3.33%	6.67%	1
d__Bacteria;p__Actinobacteriota;c__Coriobacteriia;o__Coriobacteriales;f__Atopobiaceae;g__Atopobium;s__Olsenella_sp.	1	0	1	29	0	30	species	1	3.33%	0.00%	1
d__Bacteria;p__Proteobacteria;c__Gammaproteobacteria;o__Pseudomonadales;f__Pseudomonadaceae;g__Pseudomonas;s__Pseudomonas_mosselii	2	1	2	28	1	29	species	1	6.67%	3.33%	1
d__Bacteria;p__Bacteroidota;c__Bacteroidia;o__Bacteroidales;f__Bacteroidaceae;g__Bacteroides;s__Bacteroides_caecimuris	7	6	19	11	18	12	species	1	63.33%	60.00%	1
d__Bacteria;p__Bacteroidota;c__Bacteroidia;o__Bacteroidales;f__Rikenellaceae;g__Alistipes;s__Alistipes_sp.	8	7	15	15	14	16	species	1	50.00%	46.67%	1
d__Bacteria;p__Firmicutes;c__Bacilli;o__Lactobacillales;f__Leuconostocaceae;g__Leuconostoc;s__Leuconostoc_carnosum	1	0	1	29	0	30	species	1	3.33%	0.00%	1
d__Bacteria;p__Firmicutes;c__Clostridia;o__Lachnospirales;f__Lachnospiraceae;g__Blautia;s__Blautia_producta	0	1	0	30	1	29	species	-1	0.00%	3.33%	1
d__Bacteria;p__Firmicutes;c__Clostridia;o__Clostridia_UCG-014;f__Clostridia_UCG-014;g__Clostridia_UCG-014;s__gut_metagenome	5	4	13	17	12	18	species	1	43.33%	40.00%	1
d__Bacteria;p__Proteobacteria;c__Gammaproteobacteria;o__Burkholderiales;f__Burkholderiaceae;g__Ralstonia;s__Ralstonia_solanacearum	0	1	0	30	1	29	species	-1	0.00%	3.33%	1
d__Bacteria;p__Campilobacterota;c__Campylobacteria;o__Campylobacterales;f__Campylobacteraceae;g__Campylobacter;s__Campylobacter_ureolyticus	2	3	2	28	3	27	species	-1	6.67%	10.00%	1
d__Bacteria;p__Firmicutes;c__Clostridia;o__Oscillospirales;f__Oscillospiraceae;g__UCG-005;s__human_gut	4	3	4	26	3	27	species	1	13.33%	10.00%	1
d__Bacteria;p__Firmicutes;c__Clostridia;o__Peptostreptococcales-Tissierellales;f__Peptostreptococcales-Tissierellales;g__Anaerococcus;s__Anaerococcus_octavius	2	1	2	28	1	29	species	1	6.67%	3.33%	1
d__Bacteria;p__Firmicutes;c__Clostridia;o__Clostridiales;f__Clostridiaceae;g__Clostridium_sensu_stricto_1;s__metagenome	4	3	4	26	3	27	species	1	13.33%	10.00%	1
d__Bacteria;p__Firmicutes;c__Clostridia;o__Peptostreptococcales-Tissierellales;f__Peptostreptococcales-Tissierellales;g__Peptoniphilus;s__Peptoniphilus_lacrimalis	4	5	4	26	5	25	species	-1	13.33%	16.67%	1
d__Bacteria;p__Firmicutes;c__Bacilli;o__Lactobacillales;f__Lactobacillaceae;g__Lactobacillus;s__metagenome	1	0	1	29	0	30	species	1	3.33%	0.00%	1
d__Bacteria;p__Proteobacteria;c__Gammaproteobacteria;o__Pseudomonadales;f__Pseudomonadaceae;g__Pseudomonas;s__iron-reducing_bacterium	2	1	2	28	1	29	species	1	6.67%	3.33%	1
d__Bacteria;p__Bacteroidota;c__Bacteroidia;o__Bacteroidales;f__Marinifilaceae;g__Butyricimonas;s__Butyricimonas_sp.	1	0	1	29	0	30	species	1	3.33%	0.00%	1
d__Bacteria;p__Firmicutes;c__Bacilli;o__Erysipelotrichales;f__Erysipelotrichaceae;g__Faecalitalea;s__Faecalitalea_cylindroides	3	2	4	26	3	27	species	1	13.33%	10.00%	1
d__Bacteria;p__Proteobacteria;c__Gammaproteobacteria;o__Enterobacterales;f__Enterobacteriaceae;g__Raoultella;s__Raoultella_planticola	0	1	0	30	1	29	species	-1	0.00%	3.33%	1
d__Bacteria;p__Firmicutes;c__Clostridia;o__Peptostreptococcales-Tissierellales;f__Peptostreptococcales-Tissierellales;g__Anaerococcus;s__Anaerococcus_lactolyticus	1	2	1	29	2	28	species	-1	3.33%	6.67%	1
d__Archaea;p__Thermoplasmatota	2	2	2	28	2	28	phylum	0	6.67%	6.67%	0
d__Archaea;p__Crenarchaeota	1	1	1	29	1	29	phylum	0	3.33%	3.33%	0

d__Bacteria;p__Patescibacteria	5	5	7	23	7	23	phylum	0	23.33%	23.33%	0
d__Archaea;p__Crenarchaeota;c__Nitrososphaeria	1	1	1	29	1	29	class	0	3.33%	3.33%	0
d__Bacteria;p__Proteobacteria;c__Alphaproteobacteria	7	7	14	16	14	16	class	0	46.67%	46.67%	0
d__Archaea;p__Thermoplasmatota;c__Thermoplasmata	2	2	2	28	2	28	class	0	6.67%	6.67%	0
d__Bacteria;p__Bacteroidota;c__Bacteroidia;o__Flavobacteriales	6	6	7	23	7	23	order	0	23.33%	23.33%	0
d__Bacteria;p__Patescibacteria;c__Saccharimonadia;o__Saccharimonadales	3	3	6	24	6	24	order	0	20.00%	20.00%	0
d__Bacteria;p__Actinobacteriota;c__Actinobacteria;o__Micrococcales	4	4	4	26	4	26	order	0	13.33%	13.33%	0
d__Bacteria;p__Firmicutes;c__Clostridia;o__Eubacteriales	5	5	7	23	7	23	order	0	23.33%	23.33%	0
d__Bacteria;p__Proteobacteria;c__Alphaproteobacteria;o__Caulobacterales	1	1	1	29	1	29	order	0	3.33%	3.33%	0
d__Archaea;p__Crenarchaeota;c__Nitrososphaeria;o__Nitrosopumilales	1	1	1	29	1	29	order	0	3.33%	3.33%	0
d__Archaea;p__Thermoplasmatota;c__Thermoplasmata;o__Methanomassiliicoccales	2	2	2	28	2	28	order	0	6.67%	6.67%	0
d__Bacteria;p__Actinobacteriota;c__Actinobacteria;o__Bifidobacteriales	1	1	29	1	29	1	order	0	96.67%	96.67%	0
d__Bacteria;p__Firmicutes;c__Clostridia;o__Christensenellales	1	1	29	1	29	1	order	0	96.67%	96.67%	0
d__Bacteria;p__Actinobacteriota;c__Actinobacteria;o__Bifidobacteriales;f__Bifidobacteriaceae	1	1	29	1	29	1	family	0	96.67%	96.67%	0
d__Archaea;p__Crenarchaeota;c__Nitrososphaeria;o__Nitrosopumilales;f__Nitrosopumilaceae	1	1	1	29	1	29	family	0	3.33%	3.33%	0
d__Archaea;p__Thermoplasmatota;c__Thermoplasmata;o__Methanomassiliicoccales;f__Methanomassiliicoccaceae	1	1	1	29	1	29	family	0	3.33%	3.33%	0
d__Bacteria;p__Proteobacteria;c__Gammaproteobacteria;o__Aeromonadales;f__Succinivibrionaceae	1	1	1	29	1	29	family	0	3.33%	3.33%	0
d__Bacteria;p__Bacteroidota;c__Bacteroidia;o__Bacteroidales;f__Tannerellaceae	1	1	29	1	29	1	family	0	96.67%	96.67%	0
d__Bacteria;p__Firmicutes;c__Clostridia;o__Christensenellales;f__Christensenellaceae	1	1	29	1	29	1	family	0	96.67%	96.67%	0
d__Bacteria;p__Firmicutes;c__Clostridia;o__Eubacteriales;f__Anaerofustaceae	3	3	3	27	3	27	family	0	10.00%	10.00%	0
d__Bacteria;p__Proteobacteria;c__Alphaproteobacteria;o__Caulobacterales;f__Caulobacteraceae	1	1	1	29	1	29	family	0	3.33%	3.33%	0
d__Bacteria;p__Bacteroidota;c__Bacteroidia;o__Bacteroidales;f__Porphyromonadaceae	4	4	4	26	4	26	family	0	13.33%	13.33%	0
d__Bacteria;p__Proteobacteria;c__Gammaproteobacteria;o__Burkholderiales;f__Sutterellaceae	1	1	29	1	29	1	family	0	96.67%	96.67%	0
d__Archaea;p__Thermoplasmatota;c__Thermoplasmata;o__Methanomassiliicoccales;f__Methanomethylophilaceae	1	1	1	29	1	29	family	0	3.33%	3.33%	0
d__Bacteria;p__Bacteroidota;c__Bacteroidia;o__Flavobacteriales;f__Flavobacteriaceae	6	6	7	23	7	23	family	0	23.33%	23.33%	0
d__Bacteria;p__Firmicutes;c__Clostridia;o__Oscillospirales;f__UCG-011	4	4	4	26	4	26	family	0	13.33%	13.33%	0
d__Bacteria;p__Firmicutes;c__Clostridia;o__Peptostreptococcales-Tissierellales;f__Peptostreptococcales-Tissierellales;g__Ezakiella	3	3	3	27	3	27	genus	0	10.00%	10.00%	0
d__Bacteria;p__Firmicutes;c__Negativicutes;o__Veillonellales-Selenomonadales;f__Veillonellaceae;g__Allisonella	7	7	8	22	8	22	genus	0	26.67%	26.67%	0
d__Bacteria;p__Firmicutes;c__Clostridia;o__Peptostreptococcales-Tissierellales;f__Anaerovoracaceae;g__Family_XIII_AD3011_group	3	3	27	3	27	3	genus	0	90.00%	90.00%	0
d__Bacteria;p__Firmicutes;c__Clostridia;o__Lachnospirales;f__Lachnospiraceae;g__Agathobacter	1	1	29	1	29	1	genus	0	96.67%	96.67%	0
d__Bacteria;p__Firmicutes;c__Clostridia;o__Oscillospirales;f__Oscillospiraceae;g__UCG-007	3	3	3	27	3	27	genus	0	10.00%	10.00%	0

d__Bacteria;p__Firmicutes;c__Clostridia;o__Lachnospirales;f__Lachnospiraceae;g__Lachnospiraceae_FE2018_group	1	1	1	29	1	29	genus	0	3.33%	3.33%	0
d__Bacteria;p__Firmicutes;c__Clostridia;o__Oscillospirales;f__Oscillospiraceae;g__Colidextribacter	1	1	29	1	29	1	genus	0	96.67%	96.67%	0
d__Bacteria;p__Proteobacteria;c__Gammaproteobacteria;o__Pasteurellales;f__Pasteurellaceae;g__Haemophilus	6	6	22	8	22	8	genus	0	73.33%	73.33%	0
d__Bacteria;p__Firmicutes;c__Clostridia;o__Lachnospirales;f__Lachnospiraceae;g__Lachnospiraceae_NK4A136_group	3	3	24	6	24	6	genus	0	80.00%	80.00%	0
d__Bacteria;p__Proteobacteria;c__Gammaproteobacteria;o__Pseudomonadales;f__Pseudomonadaceae;g__Pseudomonas	7	7	8	22	8	22	genus	0	26.67%	26.67%	0
d__Bacteria;p__Desulfobacterota;c__Desulfovibrionia;o__Desulfovibrionales;f__Desulfovibrionaceae;g__Desulfovibrio	6	6	11	19	11	19	genus	0	36.67%	36.67%	0
d__Bacteria;p__Proteobacteria;c__Alphaproteobacteria;o__Sphingomonadales;f__Sphingomonadaceae;g__Sphingomonas	1	1	1	29	1	29	genus	0	3.33%	3.33%	0
d__Bacteria;p__Firmicutes;c__Bacilli;o__Lactobacillales;f__Leuconostocaceae;g__Weissella	1	1	1	29	1	29	genus	0	3.33%	3.33%	0
d__Bacteria;p__Firmicutes;c__Clostridia;o__Lachnospirales;f__Lachnospiraceae;g__Lachnospiraceae_ND3007_group	4	4	25	5	25	5	genus	0	83.33%	83.33%	0
d__Bacteria;p__Firmicutes;c__Clostridia;o__Peptostreptococcales-Tissierellales;f__Anaerovoracaceae;g__[Eubacterium]_brachy_group	5	5	15	15	15	15	genus	0	50.00%	50.00%	0
d__Bacteria;p__Firmicutes;c__Clostridia;o__Peptostreptococcales-Tissierellales;f__Peptostreptococcales-Tissierellales;g__Anaerosalibacter	1	1	1	29	1	29	genus	0	3.33%	3.33%	0
d__Bacteria;p__Actinobacteriota;c__Actinobacteria;o__Actinomycetales;f__Actinomycetaceae;g__Varibaculum	5	5	5	25	5	25	genus	0	16.67%	16.67%	0
d__Bacteria;p__Firmicutes;c__Negativicutes;o__Veillonellales-Selenomonadales;f__Veillonellaceae;g__Dialister	8	8	20	10	20	10	genus	0	66.67%	66.67%	0
d__Bacteria;p__Firmicutes;c__Clostridia;o__Lachnospirales;f__Lachnospiraceae;g__[Eubacterium]_hallii_group	1	1	29	1	29	1	genus	0	96.67%	96.67%	0
d__Bacteria;p__Bacteroidota;c__Bacteroidia;o__Bacteroidales;f__Tannerellaceae;g__Parabacteroides	1	1	29	1	29	1	genus	0	96.67%	96.67%	0
d__Bacteria;p__Actinobacteriota;c__Coriobacteriia;o__Coriobacteriales;f__Eggerthellaceae;g__Senegalimassilia	8	8	8	22	8	22	genus	0	26.67%	26.67%	0
d__Bacteria;p__Firmicutes;c__Clostridia;o__Lachnospirales;f__Lachnospiraceae;g__Tuzzerella	3	3	3	27	3	27	genus	0	10.00%	10.00%	0
d__Bacteria;p__Bacteroidota;c__Bacteroidia;o__Bacteroidales;f__Prevotellaceae;g__Prevotellaceae_NK3B31_group	6	6	7	23	7	23	genus	0	23.33%	23.33%	0
d__Bacteria;p__Firmicutes;c__Bacilli;o__Erysipelotrichales;f__Erysipelatoclostridiaceae;g__Erysipelotrichaceae_UCG-003	5	5	22	8	22	8	genus	0	73.33%	73.33%	0
d__Bacteria;p__Firmicutes;c__Clostridia;o__Peptostreptococcales-Tissierellales;f__Peptostreptococcaceae;g__Intestinibacter	3	3	26	4	26	4	genus	0	86.67%	86.67%	0
d__Bacteria;p__Actinobacteriota;c__Coriobacteriia;o__Coriobacteriales;f__Eggerthellaceae;g__Enterorhabdus	7	7	7	23	7	23	genus	0	23.33%	23.33%	0
d__Archaea;p__Thermoplasmatota;c__Thermoplasmata;o__Methanomassiliicoccales;f__Methanomassiliicoccaceae;g__Methanomassiliicoccus	1	1	1	29	1	29	genus	0	3.33%	3.33%	0
d__Bacteria;p__Actinobacteriota;c__Coriobacteriia;o__Coriobacteriales;f__Coriobacteriales_Incertae_Sedis;g__Raoultibacter	1	1	1	29	1	29	genus	0	3.33%	3.33%	0
d__Bacteria;p__Firmicutes;c__Clostridia;o__Clostridiales;f__Clostridiaceae;g__Clostridium_sensu_stricto_13	1	1	1	29	1	29	genus	0	3.33%	3.33%	0
d__Bacteria;p__Firmicutes;c__Clostridia;o__Lachnospirales;f__Lachnospiraceae;g__Lachnospiraceae_UCG-001	5	5	19	11	19	11	genus	0	63.33%	63.33%	0
d__Bacteria;p__Bacteroidota;c__Bacteroidia;o__Bacteroidales;f__Rikenellaceae;g__Rikenellaceae_RC9_gut_group	4	4	4	26	4	26	genus	0	13.33%	13.33%	0
d__Bacteria;p__Firmicutes;c__Clostridia;o__Peptostreptococcales-Tissierellales;f__Peptostreptococcales-Tissierellales;g__Peptoniphilus	6	6	6	24	6	24	genus	0	20.00%	20.00%	0
d__Bacteria;p__Firmicutes;c__Bacilli;o__Lactobacillales;f__Leuconostocaceae;g__Leuconostoc	3	3	3	27	3	27	genus	0	10.00%	10.00%	0
d__Archaea;p__Euryarchaeota;c__Methanobacteria;o__Methanobacteriales;f__Methanobacteriaceae;g__Methanosphaera	2	2	2	28	2	28	genus	0	6.67%	6.67%	0
d__Bacteria;p__Proteobacteria;c__Gammaproteobacteria;o__Xanthomonadales;f__Xanthomonadaceae;g__Stenotrophomonas	2	2	2	28	2	28	genus	0	6.67%	6.67%	0
d__Bacteria;p__Firmicutes;c__Clostridia;o__Eubacteriales;f__Eubacteriaceae;g__Eubacterium	2	2	4	26	4	26	genus	0	13.33%	13.33%	0

d__Bacteria;p__Firmicutes;c__Bacilli;o__Lactobacillales;f__Aerococcaceae;g__Facklamia	1	1	1	29	1	29	genus	0	3.33%	3.33%	0
d__Archaea;p__Crenarchaeota;c__Nitrososphaeria;o__Nitrosopumilales;f__Nitrosopumilaceae;g__Candidatus_Nitrosotenuis	1	1	1	29	1	29	genus	0	3.33%	3.33%	0
d__Bacteria;p__Proteobacteria;c__Gammaproteobacteria;o__Burkholderiales;f__Sutterellaceae;g__Sutterella	7	7	22	8	22	8	genus	0	73.33%	73.33%	0
d__Bacteria;p__Firmicutes;c__Clostridia;o__Oscillospirales;f__Ruminococcaceae;g__Subdoligranulum	3	3	27	3	27	3	genus	0	90.00%	90.00%	0
d__Bacteria;p__Firmicutes;c__Clostridia;o__Christensenellales;f__Christensenellaceae;g__Christensenellaceae	1	1	1	29	1	29	genus	0	3.33%	3.33%	0
d__Bacteria;p__Firmicutes;c__Clostridia;o__Lachnospirales;f__Lachnospiraceae;g__Shuttleworthia	1	1	1	29	1	29	genus	0	3.33%	3.33%	0
d__Bacteria;p__Actinobacteriota;c__Actinobacteria;o__Actinomycetales;f__Actinomycetaceae;g__Arcanobacterium	1	1	1	29	1	29	genus	0	3.33%	3.33%	0
d__Bacteria;p__Firmicutes;c__Clostridia;o__Lachnospirales;f__Lachnospiraceae;g__Dorea	1	1	29	1	29	1	genus	0	96.67%	96.67%	0
d__Bacteria;p__Firmicutes;c__Clostridia;o__Lachnospirales;f__Lachnospiraceae;g__[Ruminococcus]_torques_group	1	1	29	1	29	1	genus	0	96.67%	96.67%	0
d__Bacteria;p__Bacteroidota;c__Bacteroidia;o__Bacteroidales;f__Porphyromonadaceae;g__Porphyromonas	4	4	4	26	4	26	genus	0	13.33%	13.33%	0
d__Bacteria;p__Firmicutes;c__Negativicutes;o__Veillonellales-Selenomonadales;f__Veillonellaceae;g__Dialister;s__Dialister_pneumosintes	1	1	1	29	1	29	species	0	3.33%	3.33%	0
d__Bacteria;p__Firmicutes;c__Bacilli;o__Staphylococcales;f__Staphylococcaceae;g__Staphylococcus;s__Staphylococcus_saprophyticus	1	1	1	29	1	29	species	0	3.33%	3.33%	0
d__Bacteria;p__Proteobacteria;c__Gammaproteobacteria;o__Enterobacterales;f__Enterobacteriaceae;g__Enterobacter;s__Enterobacter_cloacae	4	4	5	25	5	25	species	0	16.67%	16.67%	0
d__Bacteria;p__Bacteroidota;c__Bacteroidia;o__Flavobacteriales;f__Flavobacteriaceae;s__gut_metagenome	6	6	7	23	7	23	species	0	23.33%	23.33%	0
d__Bacteria;p__Firmicutes;c__Bacilli;o__Erysipelotrichales;f__Erysipelotrichaceae;g__Faecalitalea;s__Absiella_argi	1	1	1	29	1	29	species	0	3.33%	3.33%	0
d__Bacteria;p__Proteobacteria;c__Gammaproteobacteria;o__Pseudomonadales;f__Moraxellaceae;g__Acinetobacter;s__Acinetobacter_johnsonii	2	2	3	27	3	27	species	0	10.00%	10.00%	0
d__Bacteria;p__Firmicutes;c__Clostridia;o__Lachnospirales;f__Lachnospiraceae;g__Lachnospiraceae_UCG-001;s__metagenome	1	1	1	29	1	29	species	0	3.33%	3.33%	0
d__Bacteria;p__Actinobacteriota;c__Coriobacteriia;o__Coriobacteriales;f__Coriobacteriaceae;g__Collinsella;s__Collinsella_intestinalis	1	1	1	29	1	29	species	0	3.33%	3.33%	0
d__Bacteria;p__Firmicutes;c__Clostridia;o__Lachnospirales;f__Lachnospiraceae;g__[Ruminococcus]_torques_group;s__Ruminococcus_lactaris	6	6	15	15	15	15	species	0	50.00%	50.00%	0
d__Bacteria;p__Proteobacteria;c__Gammaproteobacteria;o__Pseudomonadales;f__Pseudomonadaceae;g__Pseudomonas;s__Pseudomonas_fluorescens	1	1	1	29	1	29	species	0	3.33%	3.33%	0
d__Bacteria;p__Firmicutes;c__Clostridia;o__Oscillospirales;f__Oscillospiraceae;g__Oscillibacter;s__Oscillibacter_sp.	2	2	28	2	28	2	species	0	93.33%	93.33%	0
d__Bacteria;p__Firmicutes;c__Clostridia;o__Lachnospirales;f__Lachnospiraceae;g__Blautia;s__bacterium_NLAE-zl-C378	1	1	1	29	1	29	species	0	3.33%	3.33%	0
d__Bacteria;p__Firmicutes;c__Clostridia;o__Oscillospirales;f__Ruminococcaceae;g__Paludicola;s__Ruminococcaceae_bacterium	1	1	1	29	1	29	species	0	3.33%	3.33%	0
d__Bacteria;p__Firmicutes;c__Bacilli;o__Lactobacillales;f__Streptococcaceae;g__Streptococcus;s__Streptococcus_sanguinis	3	3	3	27	3	27	species	0	10.00%	10.00%	0
d__Bacteria;p__Verrucomicrobiota;c__Lentisphaeria;o__Victivallales;f__vadinBE97;g__vadinBE97;s__Bacteroidales_bacterium	1	1	1	29	1	29	species	0	3.33%	3.33%	0
d__Bacteria;p__Firmicutes;c__Bacilli;o__Bacillales;f__Bacillaceae;g__Bacillus;s__Bacillus_circulans	1	1	1	29	1	29	species	0	3.33%	3.33%	0
d__Bacteria;p__Firmicutes;c__Clostridia;o__Peptococcales;f__Peptococcaceae;s__unidentified	1	1	1	29	1	29	species	0	3.33%	3.33%	0
d__Bacteria;p__Firmicutes;c__Clostridia;o__Oscillospirales;f__Ruminococcaceae;g__[Eubacterium]_siraeum_group;s__[Eubacterium]_siraeum	5	5	5	25	5	25	species	0	16.67%	16.67%	0
d__Bacteria;p__Firmicutes;c__Bacilli;o__Bacillales;f__Bacillaceae;g__Bacillus;s__Bacillus_sp.	1	1	1	29	1	29	species	0	3.33%	3.33%	0
d__Bacteria;p__Bacteroidota;c__Bacteroidia;o__Bacteroidales;f__Bacteroidaceae;g__Bacteroides;s__bacterium_NLAE-zl-P177	1	1	1	29	1	29	species	0	3.33%	3.33%	0
d__Bacteria;p__Firmicutes;c__Bacilli;o__Lactobacillales;f__Streptococcaceae;g__Streptococcus;s__Streptococcus_gallolyticus	1	1	1	29	1	29	species	0	3.33%	3.33%	0

d__Bacteria;p__Firmicutes;c__Bacilli;o__Lactobacillales;f__Streptococcaceae;g__Streptococcus;s__Streptococcus_lutetiensis	1	1	1	29	1	29	species	0	3.33%	3.33%	0
d__Bacteria;p__Firmicutes;c__Clostridia;o__Clostridiales;f__Clostridiaceae;g__Clostridium_sensu_stricto_13;s__Clostridium_sp.	1	1	1	29	1	29	species	0	3.33%	3.33%	0
d__Bacteria;p__Proteobacteria;c__Gammaproteobacteria;o__Enterobacterales;f__Yersiniaceae;g__Serratia;s__Serratia_sp.	1	1	1	29	1	29	species	0	3.33%	3.33%	0
d__Bacteria;p__Firmicutes;c__Clostridia;o__Eubacteriales;f__Anaerofustaceae;g__Anaerofustis;s__Anaerofustis_stercorihominis	2	2	2	28	2	28	species	0	6.67%	6.67%	0
d__Bacteria;p__Firmicutes;c__Clostridia;o__Peptostreptococcales-Tissierellales;f__Peptostreptococcales-Tissierellales;g__Peptoniphilus;s__Peptoniphilaceae_bacterium	1	1	1	29	1	29	species	0	3.33%	3.33%	0
d__Bacteria;p__Actinobacteriota;c__Actinobacteria;o__Corynebacteriales;f__Corynebacteriaceae;g__Corynebacterium;s__Corynebacterium_striatum	1	1	1	29	1	29	species	0	3.33%	3.33%	0
d__Bacteria;p__Bacteroidota;c__Bacteroidia;o__Bacteroidales;s__gut_metagenome	2	2	2	28	2	28	species	0	6.67%	6.67%	0
d__Bacteria;p__Firmicutes;c__Bacilli;o__Erysipelotrichales;f__Erysipelatoclostridiaceae;g__Erysipelatoclostridium;s__bacterium_ic1391	2	2	2	28	2	28	species	0	6.67%	6.67%	0
d__Bacteria;p__Firmicutes;c__Bacilli;o__Erysipelotrichales;f__Erysipelatoclostridiaceae;g__Asteroleplasma;s__metagenome	1	1	1	29	1	29	species	0	3.33%	3.33%	0
d__Bacteria;p__Proteobacteria;c__Gammaproteobacteria;o__Burkholderiales;f__Rhodocyclaceae;g__Dechloromonas;s__Dechloromonas_sp.	1	1	1	29	1	29	species	0	3.33%	3.33%	0
d__Bacteria;p__Firmicutes;c__Clostridia;o__Peptostreptococcales-Tissierellales;f__Anaerovoracaceae;g__Mogibacterium;s__Mogibacterium_pumilum	2	2	2	28	2	28	species	0	6.67%	6.67%	0
d__Bacteria;p__Firmicutes;c__Clostridia;o__Lachnospirales;f__Lachnospiraceae;g__[Ruminococcus]_torques_group;s__Clostridium_sp.	3	3	4	26	4	26	species	0	13.33%	13.33%	0
d__Bacteria;p__Firmicutes;c__Clostridia;o__Peptostreptococcales-Tissierellales;f__Peptostreptococcales-Tissierellales;g__Peptoniphilus;s__Peptoniphilus_koenoeneniae	2	2	2	28	2	28	species	0	6.67%	6.67%	0
d__Bacteria;p__Firmicutes;c__Clostridia;o__Peptostreptococcales-Tissierellales;f__Peptostreptococcaceae;g__Paeniclostridium;s__Paeniclostridium_sordellii	1	1	1	29	1	29	species	0	3.33%	3.33%	0
d__Bacteria;p__Bacteroidota;c__Bacteroidia;o__Bacteroidales;f__Porphyromonadaceae;g__Porphyromonas;s__Bacteroidales_bacterium	1	1	1	29	1	29	species	0	3.33%	3.33%	0
d__Bacteria;p__Firmicutes;c__Clostridia;o__Christensenellales;f__Christensenellaceae;g__Christensenella;s__Christensenella_minuta	3	3	3	27	3	27	species	0	10.00%	10.00%	0
d__Bacteria;p__Firmicutes;c__Clostridia;o__Lachnospirales;f__Lachnospiraceae;s__metagenome	4	4	8	22	8	22	species	0	26.67%	26.67%	0
d__Bacteria;p__Proteobacteria;c__Gammaproteobacteria;o__Pseudomonadales;f__Pseudomonadaceae;g__Pseudomonas;s__Pseudomonas_putida	2	2	2	28	2	28	species	0	6.67%	6.67%	0
d__Bacteria;p__Actinobacteriota;c__Coriobacteriia;o__Coriobacteriales;f__Eggerthellaceae;g__DNF00809;s__Gordonibacter_sp.	1	1	1	29	1	29	species	0	3.33%	3.33%	0
d__Bacteria;p__Actinobacteriota;c__Actinobacteria;o__Corynebacteriales;f__Corynebacteriaceae;g__Corynebacterium;s__Corynebacterium_stationis	1	1	1	29	1	29	species	0	3.33%	3.33%	0
d__Bacteria;p__Actinobacteriota;c__Actinobacteria;o__Corynebacteriales;f__Corynebacteriaceae;g__Corynebacterium;s__Corynebacterium_sp.	1	1	1	29	1	29	species	0	3.33%	3.33%	0
d__Bacteria;p__Firmicutes;c__Bacilli;o__Lactobacillales;f__Aerococcaceae;g__Facklamia;s__Facklamia_hominis	1	1	1	29	1	29	species	0	3.33%	3.33%	0
d__Bacteria;p__Actinobacteriota;c__Coriobacteriia;o__Coriobacteriales;f__Coriobacteriaceae;g__Collinsella;s__[Collinsella]_massiliensis	2	2	2	28	2	28	species	0	6.67%	6.67%	0
d__Bacteria;p__Firmicutes;c__Clostridia;o__Oscillospirales;f__Oscillospiraceae;s__metagenome	1	1	1	29	1	29	species	0	3.33%	3.33%	0
d__Bacteria;p__Firmicutes;c__Clostridia;o__Peptostreptococcales-Tissierellales;f__Anaerovoracaceae;g__Family_XIII_AD3011_group;s__gut_metagenome	6	6	16	14	16	14	species	0	53.33%	53.33%	0
d__Bacteria;p__Bacteroidota;c__Bacteroidia;o__Bacteroidales;f__Bacteroidaceae;g__Bacteroides;s__metagenome	1	1	1	29	1	29	species	0	3.33%	3.33%	0
d__Bacteria;p__Bacteroidota;c__Bacteroidia;o__Bacteroidales;f__Porphyromonadaceae;g__Porphyromonas;s__Porphyromonas_somerae	2	2	2	28	2	28	species	0	6.67%	6.67%	0
d__Bacteria;p__Firmicutes;c__Clostridia;o__Lachnospirales;f__Lachnospiraceae;g__Lachnospiraceae_ND3007_group;s__metagenome	5	5	8	22	8	22	species	0	26.67%	26.67%	0
d__Bacteria;p__Firmicutes;c__Clostridia;o__Oscillospirales;f__Ruminococcaceae;g__Ruminococcus;s__gut_metagenome	2	2	2	28	2	28	species	0	6.67%	6.67%	0
d__Bacteria;p__Firmicutes;c__Bacilli;o__Lactobacillales;f__Leuconostocaceae;g__Leuconostoc;s__endosymbiont_of	1	1	1	29	1	29	species	0	3.33%	3.33%	0
d__Bacteria;p__Firmicutes;c__Clostridia;o__Monoglobales;f__Monoglobaceae;g__Monoglobus;s__Monoglobus_pectinilyticus	7	7	7	23	7	23	species	0	23.33%	23.33%	0

d__Bacteria;p__Firmicutes;c__Negativicutes;o__Veillonellales-Selenomonadales;f__Veillonellaceae;g__Dialister;s__Dialister_sp.	3	3	3	27	3	27	species	0	10.00%	10.00%	0
d__Bacteria;p__Firmicutes;c__Bacilli;o__Lactobacillales;f__Lactobacillaceae;g__Lactobacillus;s__Lactobacillus_johnsonii	2	2	2	28	2	28	species	0	6.67%	6.67%	0
d__Bacteria;p__Proteobacteria;c__Gammaproteobacteria;o__Pseudomonadales;f__Moraxellaceae;g__Acinetobacter;s__bacterium_MCF24(2011)	1	1	1	29	1	29	species	0	3.33%	3.33%	0
d__Bacteria;p__Firmicutes;c__Clostridia;o__Oscillospirales;f__Oscillospiraceae;g__Colidextribacter;s__Clostridiales_bacterium	8	8	13	17	13	17	species	0	43.33%	43.33%	0
d__Bacteria;p__Firmicutes;c__Bacilli;o__Staphylococcales;f__Staphylococcaceae;g__Staphylococcus;s__Staphylococcus_lugdunensis	2	2	2	28	2	28	species	0	6.67%	6.67%	0
d__Bacteria;p__Verrucomicrobiota;c__Lentisphaeria;o__Victivallales;f__Victivallaceae;g__Victivallis;s__Victivallales_bacterium	9	9	10	20	10	20	species	0	33.33%	33.33%	0
d__Bacteria;p__Firmicutes;c__Clostridia;o__Lachnospirales;f__Lachnospiraceae;g__[Ruminococcus]_gauvreauii_group;s__unidentified	2	2	2	28	2	28	species	0	6.67%	6.67%	0
d__Bacteria;p__Actinobacteriota;c__Coriobacteriia;o__Coriobacteriales;f__Eggerthellaceae;g__Slackia;s__Slackia_sp.	2	2	2	28	2	28	species	0	6.67%	6.67%	0
d__Bacteria;p__Firmicutes;c__Clostridia;o__Peptostreptococcales-Tissierellales;f__Peptostreptococcales-Tissierellales;g__Anaerosalibacter;s__Anaerosalibacter_sp.	1	1	1	29	1	29	species	0	3.33%	3.33%	0
d__Bacteria;p__Verrucomicrobiota;c__Verrucomicrobiae;o__Verrucomicrobiales;f__Akkermansiaceae;g__Akkermansia;s__Akkermansia_muciniphila	6	6	12	18	12	18	species	0	40.00%	40.00%	0
d__Bacteria;p__Actinobacteriota;c__Actinobacteria;o__Bifidobacteriales;f__Bifidobacteriaceae;g__Bifidobacterium;s__Bifidobacterium_longum	1	1	1	29	1	29	species	0	3.33%	3.33%	0
d__Bacteria;p__Bacteroidota;c__Bacteroidia;o__Bacteroidales;f__Porphyromonadaceae;g__Porphyromonas;s__Porphyromonas_sp.	1	1	1	29	1	29	species	0	3.33%	3.33%	0
d__Bacteria;p__Firmicutes;c__Bacilli;o__Erysipelotrichales;f__Erysipelatoclostridiaceae;g__Erysipelatoclostridium;s__Massiliomicrobiota_timonensis	4	4	4	26	4	26	species	0	13.33%	13.33%	0
d__Bacteria;p__Firmicutes;c__Clostridia;o__Eubacteriales;f__Eubacteriaceae;g__Eubacterium;s__Eubacterium_limosum	1	1	1	29	1	29	species	0	3.33%	3.33%	0
d__Bacteria;p__Proteobacteria;c__Gammaproteobacteria;o__Pseudomonadales;f__Pseudomonadaceae;g__Pseudomonas;s__Pseudomonas_aeruginosa	1	1	2	28	2	28	species	0	6.67%	6.67%	0
d__Bacteria;p__Bacteroidota;c__Bacteroidia;o__Bacteroidales;f__Bacteroidaceae;g__Bacteroides;s__Bacteroides_massiliensis	6	6	9	21	9	21	species	0	30.00%	30.00%	0
d__Bacteria;p__Firmicutes;c__Clostridia;o__Oscillospirales;f__Butyricicoccaceae;g__Butyricicoccus;s__Agathobaculum_desmolans	1	1	2	28	2	28	species	0	6.67%	6.67%	0
d__Bacteria;p__Actinobacteriota;c__Actinobacteria;o__Actinomycetales;f__Actinomycetaceae;g__Actinomyces;s__Schaalia_odontolytica	6	6	8	22	8	22	species	0	26.67%	26.67%	0
d__Bacteria;p__Proteobacteria;c__Gammaproteobacteria;o__Xanthomonadales;f__Xanthomonadaceae;g__Stenotrophomonas;s__Stenotrophomonas_acidaminiphila	1	1	1	29	1	29	species	0	3.33%	3.33%	0
d__Bacteria;p__Firmicutes;c__Negativicutes;o__Acidaminococcales;f__Acidaminococcaceae;g__Phascolarctobacterium;s__gut_metagenome	4	4	4	26	4	26	species	0	13.33%	13.33%	0
d__Bacteria;p__Firmicutes;c__Clostridia;o__Oscillospirales;f__Ruminococcaceae;g__Anaerotruncus;s__Anaerotruncus_colihominis	8	8	14	16	14	16	species	0	46.67%	46.67%	0
d__Bacteria;p__Firmicutes;c__Clostridia;o__Peptococcales;f__Peptococcaceae;g__Peptococcus;s__Peptococcus_simiae	1	1	1	29	1	29	species	0	3.33%	3.33%	0
d__Bacteria;p__Firmicutes;c__Clostridia;o__Lachnospirales;f__Lachnospiraceae;g__Oribacterium;s__Oribacterium_parvum	1	1	1	29	1	29	species	0	3.33%	3.33%	0
d__Bacteria;p__Actinobacteriota;c__Actinobacteria;o__Corynebacteriales;f__Corynebacteriaceae;g__Corynebacterium;s__Corynebacterium_tuberculostearicum	3	3	3	27	3	27	species	0	10.00%	10.00%	0
d__Bacteria;p__Firmicutes;c__Clostridia;o__Clostridiales;f__Clostridiaceae;g__Clostridium_sensu_stricto_1;s__human_gut	3	3	4	26	4	26	species	0	13.33%	13.33%	0
d__Bacteria;p__Proteobacteria;c__Gammaproteobacteria;o__Enterobacterales;f__Enterobacteriaceae;g__Escherichia-Shigella;s__metagenome	1	1	1	29	1	29	species	0	3.33%	3.33%	0
d__Bacteria;p__Firmicutes;c__Bacilli;o__Lactobacillales;f__Lactobacillaceae;g__Lactobacillus;s__Lactobacillus_paracasei	1	1	1	29	1	29	species	0	3.33%	3.33%	0
d__Bacteria;p__Actinobacteriota;c__Actinobacteria;o__Bifidobacteriales;f__Bifidobacteriaceae;g__Bifidobacterium;s__Bifidobacterium_breve	5	5	25	5	25	5	species	0	83.33%	83.33%	0
d__Bacteria;p__Firmicutes;c__Negativicutes;o__Veillonellales-Selenomonadales;f__Veillonellaceae;g__Megasphaera;s__Megasphaera_micronuciformis	1	1	1	29	1	29	species	0	3.33%	3.33%	0
d__Bacteria;p__Firmicutes;c__Clostridia;o__Christensenellales;f__Christensenellaceae;g__Christensenellaceae_R-7_group;s__gut_metagenome	5	5	6	24	6	24	species	0	20.00%	20.00%	0
d__Bacteria;p__Actinobacteriota;c__Coriobacteriia;o__Coriobacteriales;f__Atopobiaceae;g__Olsenella;s__Olsenella_sp.	6	6	6	24	6	24	species	0	20.00%	20.00%	0

d__Bacteria;p__Firmicutes;c__Bacilli;o__Lactobacillales;f__Streptococcaceae;g__Streptococcus;s__Streptococcus_intermedius	1	1	1	29	1	29	species	0	3.33%	3.33%	0

Table S5. Prevalence table. Taxa observed in CWF sample but not in the paired F one and *vice versa*, in at least one individual. For each taxa are listed the taxonomic path (Taxa), the number of times it is observed exclusively in F samples (Exclusively in F), the number of times it is observed exclusively in CWF samples (Exclusively in CWF), the number of times it is observed in F samples (Raw F count), the number of times it is not observed in F samples (Not Observed in F), the number of times it is observed in CWF samples (Raw CWF count), the number of times it is not observed in CWF samples (Not Observed in CWF), the taxonomic rank (rank), the prevalence score (Score), the F prevalence (F prev perc), the CWF prevalence (CWF prev perc) and the absolute score value (Abn Score).

Table S6

ASV	F Network	CWF Network	Same Cluster	Taxonomy	Label	Hub
3b9509681943192007160d8caa1c0a08	Selective Yellow 2	Vivid Sky Blue	0	d_Bacteria; p_Proteobacteria; c_Gammaproteobacteria; o_Enterobacterales; f_Enterobacteriaceae; g_Citrobacter; s_bacterium_enrichment	3b9509	YES
3a06ed38de6b4163f8c71696eac6aa93	Red 2	Vivid Sky Blue	0	d_Bacteria; p_Proteobacteria; c_Gammaproteobacteria; o_Enterobacterales; f_Enterobacteriaceae; g_Citrobacter; s_Enterobacter_sp.	3a06ed	YES
bc46d697b5bf9dd0272a24bb21f13e55	Selective Yellow 2	Turquoise Blue	0	d_Bacteria; p_Bacteroidota; c_Bacteroidia; o_Bacteroidales; f_Prevotellaceae; g_Prevotella; s_uncultured_bacterium	bc46d6	YES
e7afc0299cd3a34080385c25cbeab35a	Heat Wave 2	Chlorophol green	0	d_Bacteria; p_Proteobacteria; c_Gammaproteobacteria; o_Enterobacterales; f_Enterobacteriaceae; g_Citrobacter; s_Citrobacter_freundii	e7afc0	YES
d6696fe1c5a16fb1bcb60d8cc4d1bf27	Heat Wave	Heat Wave 2	0	d_Bacteria; p_Firmicutes; c_Clostridia; o_Christensenellales; f_Christensenellaceae; g_Christensenellaceae_R-7_group; s_uncultured_bacterium	d6696f	YES
e811e72ec4c27b6c5781f86a7d256ee2	Selective Yellow 2	Mango Green	0	d_Bacteria; p_Firmicutes; c_Clostridia; o_Oscillospirales; f_Ruminococcaceae; g_Subdoligranulum	e811e7	YES
74b16132da59630e015fc31a464267b0	Red	Red	1	d_Bacteria; p_Bacteroidota; c_Bacteroidia; o_Bacteroidales; f_Bacteroidaceae; g_Bacteroides; s_Bacteroides_vulgatus	74b161	NO
9322571266f0e07ac6fb234d9ed41772	Coquelicot	Coquelicot	1	d_Bacteria; p_Proteobacteria; c_Gammaproteobacteria; o_Enterobacterales; f_Enterobacteriaceae; g_Escherichia-Shigella; s_Escherichia_coli	932257	NO
90a3db47094334e4b6240705204bd2e6	Chlorophol green	Chlorophol green	1	d_Bacteria; p_Bacteroidota; c_Bacteroidia; o_Bacteroidales; f_Bacteroidaceae; g_Bacteroides; s_Bacteroides_stercoris	90a3db	NO
ee2f52f913c478a06a0f4ce109980af6	Coquelicot	Coquelicot	1	d_Bacteria; p_Proteobacteria; c_Gammaproteobacteria; o_Enterobacterales; f_Enterobacteriaceae; g_Escherichia-Shigella; s_Shigella_boydii	ee2f52	NO
ad4447b81a1ecf31fd0af7c109566e74	Red	Red	1	d_Bacteria; p_Bacteroidota; c_Bacteroidia; o_Bacteroidales; f_Bacteroidaceae; g_Bacteroides; s_Bacteroides_vulgatus	ad4447	NO
78a5bcdc75d7ec7529096398800f8295	Red	Red	1	d_Bacteria; p_Bacteroidota; c_Bacteroidia; o_Bacteroidales; f_Bacteroidaceae; g_Bacteroides; s_Bacteroides_vulgatus	78a5bc	NO
3d21d37294087c56afc9f5cc3d1197e0	Coquelicot	Coquelicot	1	d_Bacteria; p_Proteobacteria; c_Gammaproteobacteria; o_Enterobacterales; f_Enterobacteriaceae; g_Escherichia-Shigella; s_Shigella_boydii	3d21d3	NO
3a17923daf0704b18833fba7df9d1c8f	Chlorophol green	Chlorophol green	1	d_Bacteria; p_Bacteroidota; c_Bacteroidia; o_Bacteroidales; f_Bacteroidaceae; g_Bacteroides; s_Bacteroides_stercoris	3a1792	NO
4044409c6bba3ee5d1a4c5a419772926	Chlorophol green	Chlorophol green	1	d_Bacteria; p_Bacteroidota; c_Bacteroidia; o_Bacteroidales; f_Bacteroidaceae; g_Bacteroides; s_Bacteroides_stercoris	404440	NO
d2f5b2493e42f03e164c34cba3ef4e1d	Cadmium Yellow	Cadmium Yellow	1	d_Bacteria; p_Bacteroidota; c_Bacteroidia; o_Bacteroidales; f_Bacteroidaceae; g_Bacteroides; s_Bacteroides_coprocola	d2f5b2	NO
8faf71af226cae8b032c867e9518a1ae	Electric Lime	Electric Lime	1	d_Bacteria; p_Bacteroidota; c_Bacteroidia; o_Bacteroidales; f_Bacteroidaceae; g_Bacteroides; s_Bacteroides_dorei	8faf71	NO
52227e936f84e83b80eacfcf87a8d142	Coquelicot	Coquelicot	1	d_Bacteria; p_Proteobacteria; c_Gammaproteobacteria; o_Enterobacterales; f_Enterobacteriaceae; g_Escherichia-Shigella; s_uncultured_organism	52227e	NO
cd5abe311ecbecf5c5084b5cf1d04a04	Red	Red	1	d_Bacteria; p_Bacteroidota; c_Bacteroidia; o_Bacteroidales; f_Bacteroidaceae; g_Bacteroides; s_bacterium_NLAE-zl-P803	cd5abe	NO
5dd573a403d44760227df66d62310c60	Cadmium Yellow	Cadmium Yellow	1	d_Bacteria; p_Bacteroidota; c_Bacteroidia; o_Bacteroidales; f_Bacteroidaceae; g_Bacteroides; s_Bacteroides_coprocola	5dd573	NO
f1473d3554fa43791905ff0bd9e7c8a0	Cadmium Yellow	Cadmium Yellow	1	d_Bacteria; p_Bacteroidota; c_Bacteroidia; o_Bacteroidales; f_Bacteroidaceae; g_Bacteroides; s_Bacteroides_coprocola	f1473d	NO
5c56a951a85ad9e9c05d687b6e631104	Electric Lime	Electric Lime	1	d_Bacteria; p_Bacteroidota; c_Bacteroidia; o_Bacteroidales; f_Bacteroidaceae; g_Bacteroides; s_Bacteroides_dorei	5c56a9	NO
6baa4bd3c20c243be76011e62d27c979	Electric Lime	Electric Lime	1	d_Bacteria; p_Bacteroidota; c_Bacteroidia; o_Bacteroidales; f_Bacteroidaceae; g_Bacteroides; s_Bacteroides_dorei	6baa4b	NO
8cb125559ba68d4bcfa2d8f2770fb999	Red	Red	1	d_Bacteria; p_Bacteroidota; c_Bacteroidia; o_Bacteroidales; f_Bacteroidaceae; g_Bacteroides; s_bacterium_NLAE-zl-P803	8cb125	NO
e7348210d250c9d06fa8dcc1aa514e58	Coquelicot	Coquelicot	1	d_Bacteria; p_Proteobacteria; c_Gammaproteobacteria; o_Enterobacterales; f_Enterobacteriaceae; g_Escherichia-Shigella; s_uncultured_bacterium	e73482	NO
564bf8dc0d28e4f0a1123df4d523cdbc	Chlorophol green	Chlorophol green	1	d_Bacteria; p_Bacteroidota; c_Bacteroidia; o_Bacteroidales; f_Bacteroidaceae; g_Bacteroides; s_uncultured_organism	564bf8	NO
fef2e52a0448a912ef2d28e4802edb98	Mango Green	Mango Green	1	d_Bacteria; p_Firmicutes; c_Clostridia; o_Oscillospirales; f_Ruminococcaceae; g_Faecalibacterium	fef2e5	NO
4b2251d3bd38ffaa9adead5940da2306	Eletric Green 2	Eletric Green 2	1	d_Bacteria; p_Firmicutes; c_Clostridia; o_Oscillospirales; f_Ruminococcaceae; g_Faecalibacterium; s_uncultured_bacterium	4b2251	NO
b43cd38e895cf5b33c8b52c7b7a28cd8	Coquelicot	Coquelicot	1	d_Bacteria; p_Proteobacteria; c_Gammaproteobacteria; o_Enterobacterales; f_Enterobacteriaceae; g_Escherichia-Shigella; s_Escherichia_coli	b43cd3	NO
4eb8a1472dfdf0e253b849765673b89b	Chlorophol green	Chlorophol green	1	d_Bacteria; p_Bacteroidota; c_Bacteroidia; o_Bacteroidales; f_Bacteroidaceae; g_Bacteroides; s_uncultured_organism	4eb8a1	NO
631b8fb8ddc13811cd017580b397e5e7	Red	Red	1	d_Bacteria; p_Bacteroidota; c_Bacteroidia; o_Bacteroidales; f_Bacteroidaceae; g_Bacteroides; s_Bacteroides_vulgatus	631b8f	NO
0c46222949d9fd52ab7c130967747a18	Mango Green	Mango Green	1	d_Bacteria; p_Firmicutes; c_Clostridia; o_Oscillospirales; f_Ruminococcaceae; g_Faecalibacterium; s_uncultured_organism	0c4622	NO
1e3cff0ef354282d5b17eb81c4b7da3b	Eletric Green 2	Eletric Green 2	1	d_Bacteria; p_Firmicutes; c_Clostridia; o_Oscillospirales; f_Ruminococcaceae; g_Faecalibacterium	1e3cff	NO
0289a27c44c7519efca2d96630806672	Mango Green	Mango Green	1	d_Bacteria; p_Firmicutes; c_Clostridia; o_Oscillospirales; f_Ruminococcaceae; g_Faecalibacterium; s_uncultured_organism	0289a2	NO
c65a06ca1cfeeaeb8cb21d530bf4516e	Selective Yellow	Selective Yellow	1	d_Bacteria; p_Firmicutes; c_Clostridia; o_Lachnospirales; f_Lachnospiraceae; g_Blautia; s_uncultured_bacterium	c65a06	NO
075cbccb96a6982955b1d25db991ee68	Eletric Green 2	Eletric Green 2	1	d_Bacteria; p_Firmicutes; c_Clostridia; o_Oscillospirales; f_Ruminococcaceae; g_Faecalibacterium; s_uncultured_bacterium	075cbc	NO
15ec42308d4a3557a15bdfcf3594a649	Red	Red	1	d_Bacteria; p_Bacteroidota; c_Bacteroidia; o_Bacteroidales; f_Bacteroidaceae; g_Bacteroides; s_bacterium_NLAE-zl-P803	15ec42	NO
432fef7ca53502cf6a93c0e5e8bba528	Chlorophol green	Chlorophol green	1	d_Bacteria; p_Bacteroidota; c_Bacteroidia; o_Bacteroidales; f_Bacteroidaceae; g_Bacteroides; s_Bacteroides_stercoris	432fef	NO
11d5dfba4cb9134640c629b10c182a58	Coquelicot	Coquelicot	1	d_Bacteria; p_Proteobacteria; c_Gammaproteobacteria; o_Enterobacterales; f_Enterobacteriaceae; g_Escherichia-Shigella; s_uncultured_bacterium	11d5df	NO
f1b93ff44e2421792d7a4455a53fbf67	Cadmium Yellow	Cadmium Yellow	1	d_Bacteria; p_Bacteroidota; c_Bacteroidia; o_Bacteroidales; f_Bacteroidaceae; g_Bacteroides; s_uncultured_organism	f1b93f	NO
0779bf96772607010e4821b624122318	Selective Yellow	Selective Yellow	1	d_Bacteria; p_Firmicutes; c_Clostridia; o_Lachnospirales; f_Lachnospiraceae; g_Blautia; s_uncultured_bacterium	0779bf	NO
886afc97121766ab50cd337e3b580430	Electric Lime	Electric Lime	1	d_Bacteria; p_Bacteroidota; c_Bacteroidia; o_Bacteroidales; f_Bacteroidaceae; g_Bacteroides; s_bacterium_NLAE-zl-P803	886afc	NO
78fed3eff8a67348952ec5b7a8103545	Selective Yellow	Selective Yellow	1	d_Bacteria; p_Firmicutes; c_Clostridia; o_Lachnospirales; f_Lachnospiraceae; g_Blautia; s_uncultured_bacterium	78fed3	NO
c2fc1393f91bdc5e7203cd9ad0e84aff	Selective Yellow	Selective Yellow	1	d_Bacteria; p_Firmicutes; c_Clostridia; o_Lachnospirales; f_Lachnospiraceae; g_Blautia; s_uncultured_bacterium	c2fc13	NO
469307ea322792ff93eb9ee40681ad4b	Coquelicot	Coquelicot	1	d_Bacteria; p_Proteobacteria; c_Gammaproteobacteria; o_Enterobacterales; f_Enterobacteriaceae; g_Escherichia-Shigella; s_uncultured_Shigella	469307	NO
dfbb3c545d8374d258f6c682d7e66524	Coquelicot	Coquelicot	1	d_Bacteria; p_Proteobacteria; c_Gammaproteobacteria; o_Enterobacterales; f_Enterobacteriaceae; g_Escherichia-Shigella; s_Escherichia_coli	dfbb3c	NO
bbe816e9978c4ac77b868872dc32ae6d	Cadmium Yellow	Cadmium Yellow	1	d_Bacteria; p_Bacteroidota; c_Bacteroidia; o_Bacteroidales; f_Bacteroidaceae; g_Bacteroides; s_uncultured_organism	bbe816	NO
02bffb18b3db057cdba59e1fe7d05a2a	Electric Lime	Electric Lime	1	d_Bacteria; p_Bacteroidota; c_Bacteroidia; o_Bacteroidales; f_Bacteroidaceae; g_Bacteroides; s_bacterium_NLAE-zl-P803	02bffb	NO
df74a6d3905ea1bb8155ab7ae89b85c8	Red	Red	1	d_Bacteria; p_Bacteroidota; c_Bacteroidia; o_Bacteroidales; f_Bacteroidaceae; g_Bacteroides; s_Bacteroides_vulgatus	df74a6	NO
952160bacbf3ad6bd1c34900dbc21950	Red	Red	1	d_Bacteria; p_Bacteroidota; c_Bacteroidia; o_Bacteroidales; f_Bacteroidaceae; g_Bacteroides; s_bacterium_NLAE-zl-P803	952160	NO
6c71e7a9b7ae5130dfdd8a4910a9f285	Selective Yellow	Selective Yellow	1	d_Bacteria; p_Firmicutes; c_Clostridia; o_Lachnospirales; f_Lachnospiraceae; g_Blautia; s_uncultured_bacterium	6c71e7	NO
d390bf9159e65a45f899930fe4d6015a	Selective Yellow	Selective Yellow	1	d_Bacteria; p_Firmicutes; c_Clostridia; o_Lachnospirales; f_Lachnospiraceae; g_Blautia; s_uncultured_bacterium	d390bf	NO
1f74b1cc28a00d85e0906a01f21aca8c	Heat Wave	Heat Wave	1	d_Bacteria; p_Firmicutes; c_Clostridia; o_Oscillospirales; f_Ruminococcaceae; g_[Eubacterium]_siraeum_group	1f74b1	NO
06df8be5640469d72961dcb4034e34fc	Heat Wave	Heat Wave	1	d_Bacteria; p_Firmicutes; c_Clostridia; o_Oscillospirales; f_Ruminococcaceae; g_[Eubacterium]_siraeum_group	06df8b	NO
d393c8dd110309b0f95e8a402781fc04	Cadmium Yellow	Cadmium Yellow	1	d_Bacteria; p_Bacteroidota; c_Bacteroidia; o_Bacteroidales; f_Bacteroidaceae; g_Bacteroides; s_Bacteroides_coprocola	d393c8	NO
ab67c806f8970d2971e449f4f9c6ac4b	Heat Wave	Heat Wave	1	d_Bacteria; p_Firmicutes; c_Clostridia; o_Oscillospirales; f_Ruminococcaceae; g_[Eubacterium]_siraeum_group	ab67c8	NO

e5680be6b3411f3554b5caeb219d3de3	Mango Green	Mango Green	✔	1	d_Bacteria; p_Firmicutes; c_Clostridia; o_Oscillospirales; f_Ruminococcaceae; g_Faecalibacterium; s_uncultured_Faecalibacterium	e5680b	NO
c79db8ce91d6022140e3e34d94914cf4	Eletric Green 2	Eletric Green 2	✔	1	d_Bacteria; p_Firmicutes; c_Clostridia; o_Oscillospirales; f_Ruminococcaceae; g_Faecalibacterium; s_uncultured_bacterium	c79db8	NO
83ea1b468fda37dc6bf7ad1eec6dba54	Electric Lime	Electric Lime	✔	1	d_Bacteria; p_Bacteroidota; c_Bacteroidia; o_Bacteroidales; f_Bacteroidaceae; g_Bacteroides; s_Bacteroides_dorei	83ea1b	NO
db72d16355ce1ee0276ea85a4fc9a34d	Red	Red	✔	1	d_Bacteria; p_Bacteroidota; c_Bacteroidia; o_Bacteroidales; f_Bacteroidaceae; g_Bacteroides; s_bacterium_NLAE-zl-P803	db72d1	NO
4af8b443e6a1d5b9b0c18429a2f0deb3	Heat Wave	Heat Wave	✔	1	d_Bacteria; p_Verrucomicrobiota; c_Verrucomicrobiae; o_Verrucomicrobiales; f_Akkermansiaceae; g_Akkermansia; s_Akkermansia_muciniphila	4af8b4	NO
c000b56fb89322e0bd092d9eae3e30c1	Cadmium Yellow	Cadmium Yellow	✔	1	d_Bacteria; p_Bacteroidota; c_Bacteroidia; o_Bacteroidales; f_Bacteroidaceae; g_Bacteroides; s_uncultured_organism	c000b5	NO
46c27a4941071dfa2a9fa1766b2f8818	Eletric Green 2	Eletric Green 2	✔	1	d_Bacteria; p_Firmicutes; c_Clostridia; o_Oscillospirales; f_Ruminococcaceae; g_Faecalibacterium; s_uncultured_bacterium	46c27a	NO
c55064ec1a4450c1ce6914c2c72e4c75	Mango Green	Mango Green	✔	1	d_Bacteria; p_Firmicutes; c_Clostridia; o_Oscillospirales; f_Ruminococcaceae; g_Faecalibacterium; s_uncultured_Faecalibacterium	c55064	NO
75135f3dc277a122aa4ab5d5e2db3249	Electric Lime	Electric Lime	✔	1	d_Bacteria; p_Bacteroidota; c_Bacteroidia; o_Bacteroidales; f_Bacteroidaceae; g_Bacteroides; s_bacterium_NLAE-zl-P803	75135f	NO
85b1283e416fa09e6e5a745f23a5a7c4	Selective Yellow	Selective Yellow	✔	1	d_Bacteria; p_Firmicutes; c_Clostridia; o_Lachnospirales; f_Lachnospiraceae; g_Blautia; s_uncultured_bacterium	85b128	NO
adf529d8f2c86fbf5a666396a8022245	Mango Green	Mango Green	✔	1	d_Bacteria; p_Firmicutes; c_Clostridia; o_Oscillospirales; f_Ruminococcaceae; g_Faecalibacterium	adf529	NO
3d36ec8469287e8b5fa771858cbb7bd2	Heat Wave	Heat Wave	✔	1	d_Bacteria; p_Verrucomicrobiota; c_Verrucomicrobiae; o_Verrucomicrobiales; f_Akkermansiaceae; g_Akkermansia; s_uncultured_bacterium	3d36ec	NO
98d83c81b2ffcf6e574f423659615d6f	Heat Wave	Heat Wave	✔	1	d_Bacteria; p_Verrucomicrobiota; c_Verrucomicrobiae; o_Verrucomicrobiales; f_Akkermansiaceae; g_Akkermansia; s_uncultured_bacterium	98d83c	NO
ea0b90112df5108351acf5bc1975526b	Heat Wave	Heat Wave	✔	1	d_Bacteria; p_Actinobacteriota; c_Coriobacteriia; o_Coriobacteriales; f_Coriobacteriaceae; g_Collinsella	ea0b90	NO
7ac25bbcd12a002ecc95143b2c46a3c3	Selective Yellow	Vivid Sky Blue	✖	0	d_Bacteria; p_Proteobacteria; c_Gammaproteobacteria; o_Enterobacterales; f_Enterobacteriaceae; g_Citrobacter; s_Citrobacter_freundii	7ac25b	NO
f91e45acc04434992132c666852e7ae1	Selective Yellow	Vivid Sky Blue	✖	0	d_Bacteria; p_Proteobacteria; c_Gammaproteobacteria; o_Enterobacterales; f_Enterobacteriaceae; g_Citrobacter; s_Citrobacter_freundii	f91e45	NO
f269b5b89c5a84fcb78ba0934dfe66d2	Electric Green	Vivid Sky Blue	✖	0	d_Bacteria; p_Proteobacteria; c_Gammaproteobacteria; o_Enterobacterales; f_Enterobacteriaceae; g_Citrobacter; s_Citrobacter_freundii	f269b5	NO
89f21f3fe07eed34f9338b842b56dc26	Eletric Green 2	Cadmium Yellow	✖	0	d_Bacteria; p_Firmicutes; c_Clostridia; o_Lachnospirales; f_Lachnospiraceae; g_Roseburia; s_uncultured_organism	89f21f	NO
39d7726992584a551cb6cbdeea243831	Heat Wave	Turquoise Blue	✖	0	d_Bacteria; p_Bacteroidota; c_Bacteroidia; o_Bacteroidales; f_Bacteroidaceae; g_Bacteroides; s_uncultured_bacterium	39d772	NO
f015db673662aef0ae152f0e046632b4	Electric Green	Selective Yellow	✖	0	d_Bacteria; p_Firmicutes; c_Clostridia; o_Oscillospirales; f_Ruminococcaceae; g_CAG-352; s_uncultured_bacterium	f015db	NO
9b81a828ae141e7c1dca8eac3968a983	Eletric Green 2	Cadmium Yellow	✖	0	d_Bacteria; p_Firmicutes; c_Clostridia; o_Lachnospirales; f_Lachnospiraceae; g_Roseburia; s_uncultured_organism	9b81a8	NO
c64285d96aa72e927f0a15f17a1df620	Eletric Green 2	Cadmium Yellow	✖	0	d_Bacteria; p_Firmicutes; c_Clostridia; o_Lachnospirales; f_Lachnospiraceae; g_Roseburia; s_uncultured_organism	c64285	NO
0cbcb8defbfb27518aca96a1a4108ae6	Eletric Green 2	Vivid Sky Blue	✖	0	d_Bacteria; p_Firmicutes; c_Clostridia; o_Lachnospirales; f_Lachnospiraceae; g_Agathobacter; s_uncultured_bacterium	0cbcb8	NO
a7c4721759bccb5ad12c5dd38f478a71	Heat Wave	Turquoise Blue	✖	0	d_Bacteria; p_Bacteroidota; c_Bacteroidia; o_Bacteroidales; f_Bacteroidaceae; g_Bacteroides; s_uncultured_bacterium	a7c472	NO
15ce42924cf1bc2268895d83cbfc7c24	Heat Wave	Turquoise Blue	✖	0	d_Bacteria; p_Bacteroidota; c_Bacteroidia; o_Bacteroidales; f_Bacteroidaceae; g_Bacteroides; s_uncultured_bacterium	15ce42	NO
84519eecee5956be7ffbf6727762b70e	Eletric Green 2	Vivid Sky Blue	✖	0	d_Bacteria; p_Firmicutes; c_Clostridia; o_Lachnospirales; f_Lachnospiraceae; g_Agathobacter; s_uncultured_bacterium	84519e	NO
b0adcd52c98958d3cbece16871f5b250	Eletric Green 2	Vivid Sky Blue	✖	0	d_Bacteria; p_Firmicutes; c_Clostridia; o_Lachnospirales; f_Lachnospiraceae; g_Agathobacter	b0adcd	NO
5179f86d4a49574565ca12ca528c57d9	Electric Green	Selective Yellow	✖	0	d_Bacteria; p_Firmicutes; c_Clostridia; o_Oscillospirales; f_Ruminococcaceae; g_Ruminococcus; s_uncultured_bacterium	5179f8	NO
0c0609cebf40dff9ea4ff8bcb106b473	Medium Spring Green	Vivid Cerulean	✖	0	d_Bacteria; p_Firmicutes; c_Clostridia; o_Oscillospirales; f_Ruminococcaceae; g_Faecalibacterium; s_uncultured_bacterium	0c0609	NO
73528828fe210ceb7cf95703608d42dd	Selective Yellow	Turquoise Blue	✖	0	d_Bacteria; p_Bacteroidota; c_Bacteroidia; o_Bacteroidales; f_Prevotellaceae; g_Prevotella; s_uncultured_bacterium	735288	NO
230e8e9de43fea3ea471511d61300bd1	Electric Green	Selective Yellow	✖	0	d_Bacteria; p_Firmicutes; c_Clostridia; o_Oscillospirales; f_Ruminococcaceae; g_CAG-352; s_uncultured_bacterium	230e8e	NO
1627f429742b67c5bfe8cea414a379cc	Heat Wave	Chlorophol green	✖	0	d_Bacteria; p_Bacteroidota; c_Bacteroidia; o_Bacteroidales; f_Bacteroidaceae; g_Bacteroides; s_Bacteroides_plebeius	1627f4	NO
7ff41a0c5db28f7226c6f66cf7bb7306	Selective Yellow	Turquoise Blue	✖	0	d_Bacteria; p_Firmicutes; c_Clostridia; o_Oscillospirales; f_Ruminococcaceae; g_Ruminococcus; s_uncultured_bacterium	7ff41a	NO
be393e379eb6c5d45885f42b54b03a2c	Medium Spring Green	Vivid Cerulean	✖	0	d_Bacteria; p_Firmicutes; c_Clostridia; o_Oscillospirales; f_Ruminococcaceae; g_Faecalibacterium; s_uncultured_bacterium	be393e	NO
66bdbda32f534458b6bd1906ced6f4d6	Medium Spring Green	Vivid Cerulean	✖	0	d_Bacteria; p_Firmicutes; c_Clostridia; o_Oscillospirales; f_Ruminococcaceae; g_Faecalibacterium; s_uncultured_bacterium	66bdbd	NO
8381f2e7a23b32ab72281130c8ee6e73	Selective Yellow	Heat Wave	✖	0	d_Bacteria; p_Firmicutes; c_Clostridia; o_Christensenellales; f_Christensenellaceae; g_Christensenellaceae_R-7_group; s_uncultured_bacterium	8381f2	NO
a1bd492b921a3de58fa3a95cb9fad2a7	Selective Yellow	Turquoise Blue	✖	0	d_Bacteria; p_Bacteroidota; c_Bacteroidia; o_Bacteroidales; f_Prevotellaceae; g_Prevotella; s_uncultured_bacterium	a1bd49	NO
12c7b7343aad2136294c893c1efbfcb5	Selective Yellow	Turquoise Blue	✖	0	d_Bacteria; p_Firmicutes; c_Clostridia; o_Oscillospirales; f_Ruminococcaceae; g_Ruminococcus; s_uncultured_bacterium	12c7b7	NO
36c38b1013d71f4caa81e09f1baef6b5	Heat Wave	Chlorophol green	✖	0	d_Bacteria; p_Bacteroidota; c_Bacteroidia; o_Bacteroidales; f_Bacteroidaceae; g_Bacteroides; s_Bacteroides_plebeius	36c38b	NO
4984848158c17a955793848c63501cc1	Selective Yellow	Turquoise Blue	✖	0	d_Bacteria; p_Firmicutes; c_Clostridia; o_Oscillospirales; f_Ruminococcaceae; g_Ruminococcus; s_uncultured_bacterium	498484	NO
f09dd363e1e851de185ea83ebab6f4d9	Selective Yellow	Heat Wave	✖	0	d_Bacteria; p_Firmicutes; c_Clostridia; o_Christensenellales; f_Christensenellaceae; g_Christensenellaceae_R-7_group; s_uncultured_organism	f09dd3	NO
7c5a476937acc559fb6794f91be37305	Heat Wave	Cadmium Yellow	✖	0	d_Bacteria; p_Firmicutes; c_Clostridia; o_Clostridiales; f_Clostridiaceae; g_Clostridium_sensu_stricto_1; s_uncultured_bacterium	7c5a47	NO
b44152431e2ec094e06c7447dd3df5d9	Electric Green	Selective Yellow	✖	0	d_Bacteria; p_Firmicutes; c_Clostridia; o_Oscillospirales; f_Ruminococcaceae; g_Ruminococcus; s_uncultured_bacterium	b44152	NO
396f60e46e6c47df40aa8b718db4d5f3	Guppie Green	Brandeis Blue	✖	0	d_Bacteria; p_Bacteroidota; c_Bacteroidia; o_Bacteroidales; f_Bacteroidaceae; g_Bacteroides; s_Bacteroides_uniformis	396f60	NO
74f3bad0d4ba0afc7a0ba4bd5ac8f952	Medium Spring Green	Turquoise Blue	✖	0	d_Bacteria; p_Firmicutes; c_Clostridia; o_Oscillospirales; f_Ruminococcaceae; g_Subdoligranulum; s_uncultured_bacterium	74f3ba	NO
8d89f4772a004c5af4b487ae48abe074	Selective Yellow	Cadmium Yellow	✖	0	d_Bacteria; p_Firmicutes; c_Clostridia; o_Christensenellales; f_Christensenellaceae; g_Christensenellaceae_R-7_group; s_uncultured_bacterium	8d89f4	NO
32858b664f767689be429ecaae5fd82f	Medium Spring Green	Vivid Cerulean	✖	0	d_Bacteria; p_Firmicutes; c_Clostridia; o_Oscillospirales; f_Ruminococcaceae; g_Faecalibacterium; s_Faecalibacterium_prausnitzii	32858b	NO
46acc27e3e42b4043a25a0359dd18415	Electric Green	Selective Yellow	✖	0	d_Bacteria; p_Firmicutes; c_Clostridia; o_Oscillospirales; f_Ruminococcaceae; g_Ruminococcus; s_uncultured_bacterium	46acc2	NO
360747578156fe1a57a22ff03e852507	Guppie Green	Brandeis Blue	✖	0	d_Bacteria; p_Bacteroidota; c_Bacteroidia; o_Bacteroidales; f_Bacteroidaceae; g_Bacteroides; s_Bacteroides_uniformis	360747	NO
49f600f55bc3336fb862a2cf89eca81b	Guppie Green	Brandeis Blue	✖	0	d_Bacteria; p_Bacteroidota; c_Bacteroidia; o_Bacteroidales; f_Bacteroidaceae; g_Bacteroides; s_Bacteroides_uniformis	49f600	NO
f25868bf7a5261fea93a5b86cc500f98	Heat Wave	Selective Yellow	✖	0	d_Bacteria; p_Firmicutes; c_Clostridia; o_Lachnospirales; f_Lachnospiraceae; g_Blautia; s_uncultured_bacterium	f25868	NO

Table S6. List of nodes represented in the F and CWF microbial networks and their attributes. For each node the following attributes are listed: 1) ASV: asv name; 2) F Network: cluster colour in F network; 3) CWF Network: cluster colour in CWF network: 4) Same Cluster: logical attribute indicating if the nodes are grouped in the

same clusters in the two networks; 5) Taxonomy: ASV taxonomic classification; 6) Label: ASV label shortcut; 7) Hub: attribute indicating if the ASV is an hub (YES) or not (NO).
